# A short treatise concerning a musical approach for the interpretation of gene expression data

**DOI:** 10.1038/srep15281

**Published:** 2015-10-16

**Authors:** Martin S. Staege

**Affiliations:** 1 Department of Paediatrics, Martin Luther University Halle-Wittenberg.

## Abstract

Recent technical developments allow the genome-wide and near-complete analysis of gene expression in a given sample, e.g. by usage of high-density DNA microarrays or next generation sequencing. The generated data structure is usually multi-dimensional and requires extensive processing not only for analysis but also for presentation of the results. Today, such data are usually presented graphically, e.g. in the form of heat maps. In the present paper, we propose an alternative form of analysis and presentation which is based on the transformation of gene expression data into sounds that are characterized by their frequency (pitch) and tone duration. Using DNA microarray data from a panel of neuroblastoma and Ewing sarcoma cell lines as well as from Hodgkin’s lymphoma cell lines and normal B cells, we demonstrate that this Gene Expression Music Algorithm (GEMusicA) can be used for discrimination between samples with different biology and for the characterization of differentially expressed genes.

Gene expression data can be used (i) for classification of biological samples, *e.g.* for classification of tumours, (ii) for the identification of target structures, *e.g.* for the identification of tumour-specific transcripts, and (iii) for biological studies, *e.g.* for the identification of pathways that drive tumour cell proliferation or cell death^1^. Today, the genome wide analysis of the complete transcriptome of individual samples is possible, e.g. by usage of DNA microarrays. A common part for all these applications is the identification of differentially expressed genes, *i.e.* the identification of gene specific probes or probe sets that show different signal intensities in different samples. Independent of the algorithms used for the identification of these probe sets, the result is a list of probes or probe sets that have some information content and that need further interpretation.

Frequently, microarray data are presented by graphical methods, *e.g*. in the form of two-dimensional heat-maps. Heat-maps transform the signal intensities into different colours whereby different colour schemes can be used. People with dyschromatopsia might have difficulties to interpret such heat-maps if red and green are used for presentation of high and low signal intensities. Similarly, black-and-white prints from originally coloured heat-maps can completely lose the validity. These examples demonstrate that the visual presentation of microarray data has some limits.

Here, we describe an alternative way for data presentation that is based on the transformation of signal intensities into sounds. At first, the developed algorithm was tested on a data set (Gene Expression Omnibus accession number GSE1824) consisting of microarray data from four cell lines that were initially established as neuroblastoma cell lines^2^. This data set was used because the gene expression profile of all samples is well characterized and the cell lines have a similar phenotype but significant differences in their gene expression profile. One of these cell lines (SK-N-MC) was identified as being not derived from neuroblastoma. In contrast, this cell line clearly belongs to the Ewing sarcoma family^2^. Neuroblastomas and Ewing sarcomas are both members of the family of so-called small round blue cell tumours. These tumours have a similar histological appearance but require different treatment. At the molecular level, neuroblastoma cells and Ewing sarcoma cells show several specific alterations. For example, Ewing sarcomas express tumour-specific TET-ETS fusion transcripts^3^. In addition, the gene expression profile of these tumours is highly different and microarray analysis can be used for identification of Ewing sarcoma samples^2^,^4^. The second data set that was used in the present investigation was derived from Hodgkin’s lymphoma (HL) cell lines. Despite the proposed origin of HL cells from lymphocytes, the gene expression profile of HL cells is characterized by absence of typical lineage markers. Nevertheless, the gene expression profile discriminates HL cells from other normal and hematopoietic cells^5^,^6^. The HL data set was based on Affymetrix Human Exon arrays with several hundreds of thousands of probe sets and was used for the evaluation of the Gene Expression Music Algorithm (see below) on such large data sets (the used data set contains 1,411,399 probe sets).

## Results and Discussion

**Figure 1:**
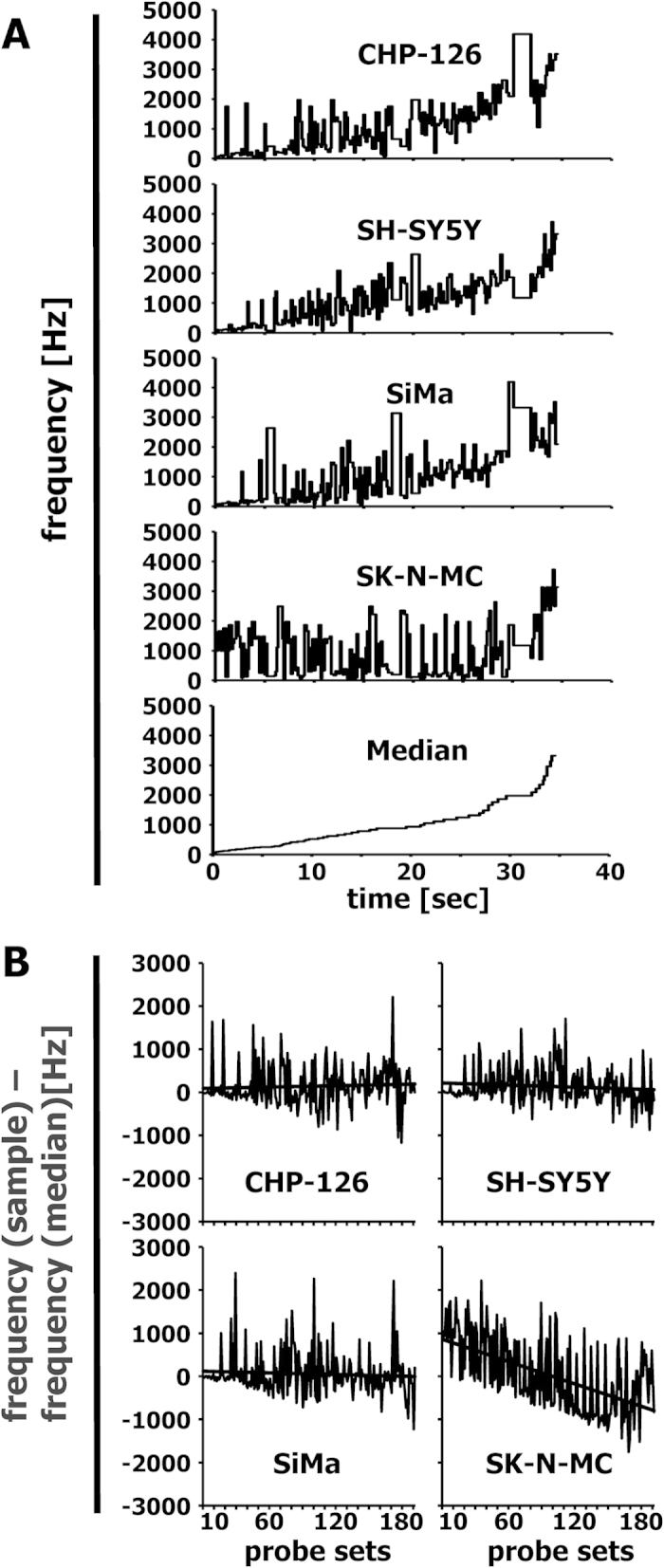
Frequency courses for the musically interpreted microarray data from “neuroblastoma” cell lines. DNA microarray data from 4 cell lines that were initially established as neuroblastoma cell lines (GSE1824) were transformed into melodies as described in the Methods section by using the following parameters: minimal frequency: 27.5; number of different frequencies: 88; number of tone steps per octave: 12; minimal duration: 1/8; number of tones: 192. (**A**) Presented are frequencies of individual cell lines and the frequency of the median signal intensity as a function of time. (**B**) Presented are the absolute differences between the frequencies from individual samples and the frequency of the median of the signal intensities. The straight line represents the linear trend line.

**Figure 2:**
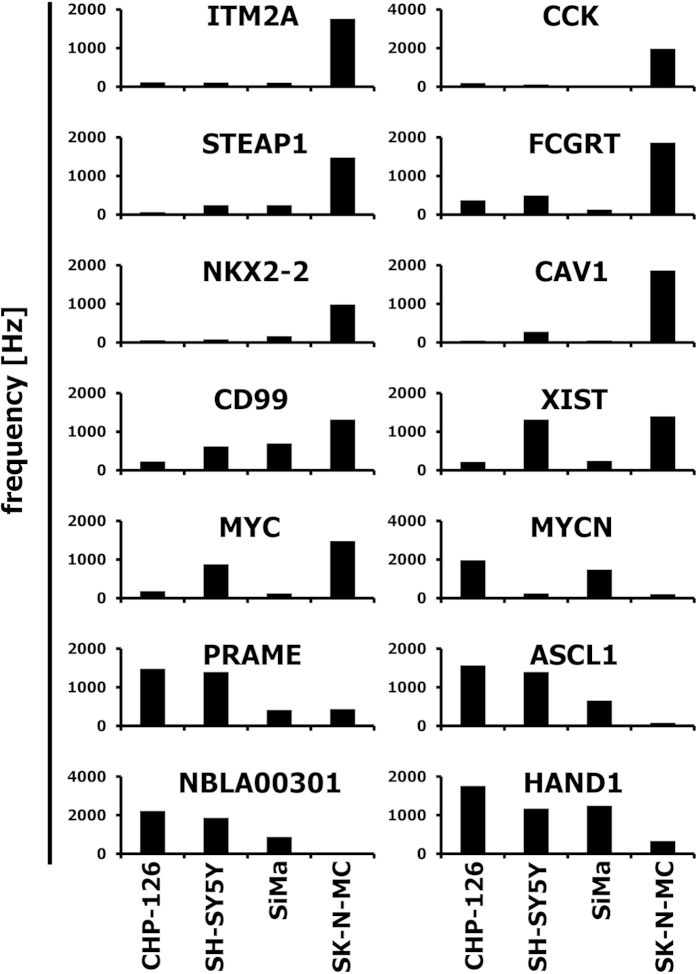
Examples of individual sounds representing probe sets with specificity for differentially expressed genes in the “neuroblastoma” data set. The transformation of the microarray data into sounds was performed as described in the legend to Fig. 1. Presented are frequencies of the musically interpreted signal intensities of the indicated genes (probe sets) in the individual cell lines.

As described in the Methods section, the Gene Expression Music Algorithm (GEMusicA) transforms signal intensities from DNA microarray data into sounds with a specific frequency and duration. The present implementation (see below) allows the choice of the number of transformed probe sets and the choice of the frequency range used for the transformation. In general, it is possible to transform complete data sets into sounds. However, probe sets with low variability can be omitted in order to shorten the length of the melodies. We used 10% of the probe sets with highest variances and transformed these probe sets into sounds. The resulting frequencies are presented in Supplementary Table S1. The resulting melodies are available as MP3 audio files 1CHP2228, 2SHSY5Y2228, 3SIMA2228, 4SKNMC2228, and 5MedSRBCT2228 from the International Music Score Library Project (IMSLP) “Tumour Music” (http://imslp.org/). The frequency courses of the individual melodies are presented in Supplementary Fig. S1. Especially at the beginning of the melodies, the differences between the neuroblastoma cell lines and the Ewing sarcoma cell line SK-N-MC are obvious. Using the median of the frequencies for a probe set in all samples as a reference baseline, we combined the melodies individually with the melody from the median and displayed both melodies separately on two channels. The resulting stereo files are available as IMSLP MP3 files 6CHP2228st, 7SHSY5Y2228st, 8SIMA2228st, and 9SKNMC2228st. The simultaneous presence of the reference melody from the median and the individual melodies increases the audibility of differences between the true neuroblastoma cell lines and the outlier SK-N-MC. The highest differences between the melodies are clustered at the beginning of the melodies. Therefore, we further reduced the number of probe sets. We used the 192 (=12 semitone steps × 4 principal lengths × 4 cell lines) probe sets with highest variance for the transformation of the neuroblastoma data set into sounds (Fig. 1). The corresponding probe sets are presented in Supplementary Table S2. The melodies are available as IMSLP MP3 files 10CHP192, 11SHSY5Y192, 12SIMA192, 13SKNMC192, and 14MedSRBCT192. Stereo versions with the median as reference base line are available as IMSLP MP3 files 15CHP192st, 16SHSY5Y192st, 17SIMA192st, and 18SKNMC192st. All cell lines showed individual frequency peaks (Fig. 1A). Probe sets with high frequencies are perceived in the background of lower frequencies and can be used for the generation of separate melodies (see below). Only 18, 22, and 18 probe sets from CHP-126 cells, SH-SY5Y cells, and SiMa cells, respectively, have frequencies that are two times (one octave) higher than the median frequencies. In contrast, 52 probe sets from SK-N-MC cells have these high frequencies. The probe sets with high frequencies are marked in bold face in Supplementary Table S2. True neuroblastoma cell lines showed frequencies with an average increase over time whereas the Ewing sarcoma cell line SK-N-MC showed such increase only at the end of the melody (Fig. 1A). The difference between the median and SK-N-MC cells decreases over time (Fig. 1B). Whereas at the beginning of the melody the frequencies from SK-N-MC cells are higher than the median frequency, these frequencies are lower at the end of the melody. Importantly, the 192 variance-filtered probe sets include highly specific genes for the investigated samples (Fig. 2). For instance, the Ewing sarcoma specific genes^2^,^7^,^8^,^9^ cholecystokinin (CCK), integral membrane protein 2A (ITM2A), six transmembrane epithelial antigen of the prostate 1 (STEAP1), Fc fragment of IgG receptor transporter alpha (FCGRT), caveolin 1 (CAV1), CD99, and NK2 homeobox 2 (NKX2-2) were represented by sounds with high frequencies in SK-N-MC cells and low frequencies in the true neuroblastoma cells (Fig. 2). *Vice versa*, the genes heart and neural crest derivatives expressed 1 (HAND1) and NBLA00301 (ref. 10) were represented by sounds with high frequencies in neuroblastoma cells and low frequencies in SK-N-MC cells. The X inactive specific transcript XIST^11^ was represented by high frequencies only in the samples of female patients (SH-SY5Y cells and SK-N-MC cells). The mutually exclusive expression of the v-myc avian myelocytomatosis viral oncogene homolog (MYC) and the neuroblastoma derived v-myc avian myelocytomatosis viral oncogene homolog (MYCN) results in sounds with mutually exclusive high frequencies (Fig. 2). Individual differences in the expression pattern were also visible. Quantitative RT-PCR validated the differential expression of the analysed genes in the different cell lines (Supplementary Fig. S2). High expression of ITM2A seems to be a specific feature of Ewing sarcoma cells as indicated by conventional PCR in other cell lines from Ewing sarcoma (Supplementary Fig. S2).

**Figure 3:**
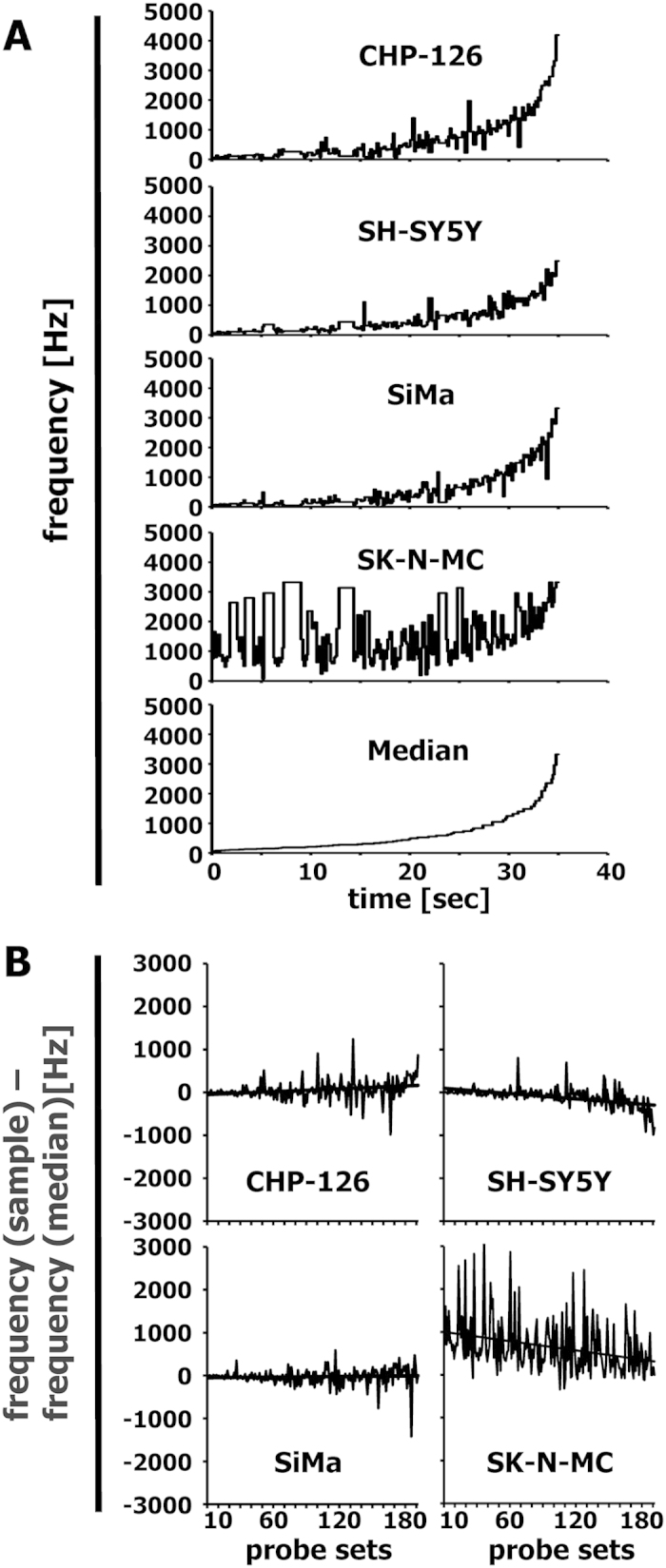
Musical interpretation of Ewing sarcoma-specific probe sets from “neuroblastoma” cell lines. DNA microarray data from a panel of Ewing sarcoma biopsies and neuroblastoma biopsies (GSE1825) were used for the identification of Ewing sarcoma specific probe sets. To this end, MAFilter was used for filtering probe sets with maximal ratios between the median in Ewing sarcomas and the 85^th^ percentile in neuroblastoma samples. All probe sets with a fold change >3 were considered Ewing sarcoma-specific. DNA microarray data from these 376 probe sets in an independent data set of 4 cell lines that were initially established as neuroblastoma cell lines (GSE1824) were transformed into melodies by using the same parameters as in Fig. 1. (**A**) Presented are frequencies of the individual cell lines and the frequency of the median signal intensity as a function of time. (**B**) Presented are absolute differences between the frequencies from individual samples and the frequency of the median of the signal intensities. The straight line represents the linear trend line.

**Figure 4:**
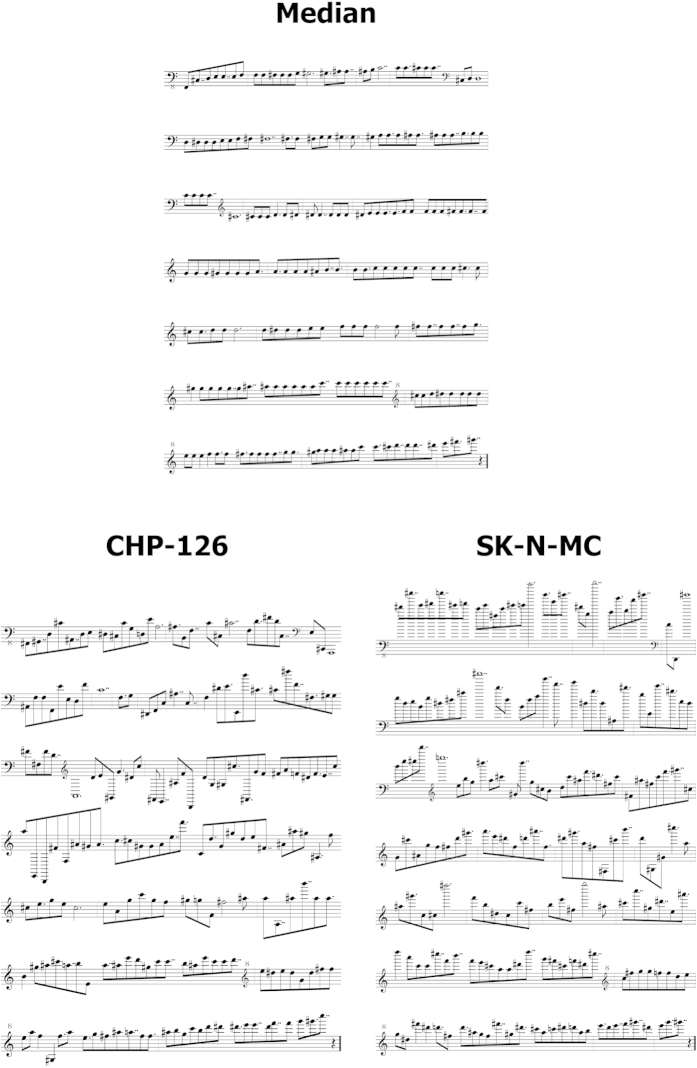
Musical interpretation of Ewing sarcoma-specific probe sets from “neuroblastoma” cell lines. The transformation of the microarray data into sounds was performed as described in the legend to Fig. 3. Presented are the resulting musical scores from CHP-126 cells and SK-N-MC cells and the median signal intensity. Clefs were optimized for the score from the median and the same clefs were used in all other scores. Music scores were generated with capella studio version 5.1-06 (capella Software GmbH, Söhrewald, Germany).

In our original publication describing the gene expression in the 4 cells lines^2^ we demonstrated that SK-N-MC cells can be characterized as Ewing sarcoma cell line on the basis of Ewing sarcoma specific genes which were all expressed in this cell line. Figure 3 demonstrates similar results for the musically interpreted gene expression data. In this case we pre-filtered the data for probe sets with high signal intensity in Ewing sarcoma samples in comparison to neuroblastoma samples^2^ by using MAFilter^6^. The probe sets filtered by MAFilter are presented as Supplementary Information. Again, we used the 192 probe sets with highest variance for the transformation into sounds. The corresponding probe sets are presented in Supplementary Table S3. As expected, the frequencies of Ewing sarcoma specific genes in SK-N-MC cells were all above the median (Fig. 3). The frequency course for cell line SK-N-MC showed several peaks in contrast to the frequencies from true neuroblastoma cell lines that increased only at the end of the melody (Fig. 3A). The melodies are available as IMSLP MP3 files 19CHPEFTspec192, 20SHSY5YEFTspec192, 21SIMAEFTspec192, 22SKNMCEFTspec192, and 23MedEFTspec192. Stereo versions with the median as reference base line are available as IMSLP MP3 files 24CHPEFTspec192st, 25SHSY5YEFTspec192st, 26SIMAEFTspec192st, and 27SKNMCEFTspec192st. As described in detail in the Methods section, we used the keyboard of a standard piano-forte as basis for transformation. Therefore it is possible to present the corresponding melodies in the form of a musical score. Figure 4 shows the scores of the Ewing sarcoma specific probe sets for one of the neuroblastoma cell lines (CHP-126) and the Ewing sarcoma cell line (SK-N-MC). The higher frequencies in the Ewing sarcoma cell line are clearly visible in the scores. An interesting feature of the used algorithm is the fact that this algorithm transforms the signal intensities, which are log-transformed, into keys which also represent log-transformed frequencies. By filtering probe sets with highest variance this algorithm over-represents probe sets with higher signal intensities. This effect is desired because *prima facie* genes with higher expression are more likely to have a high impact on the phenotype of a cell.

**Figure 5:**
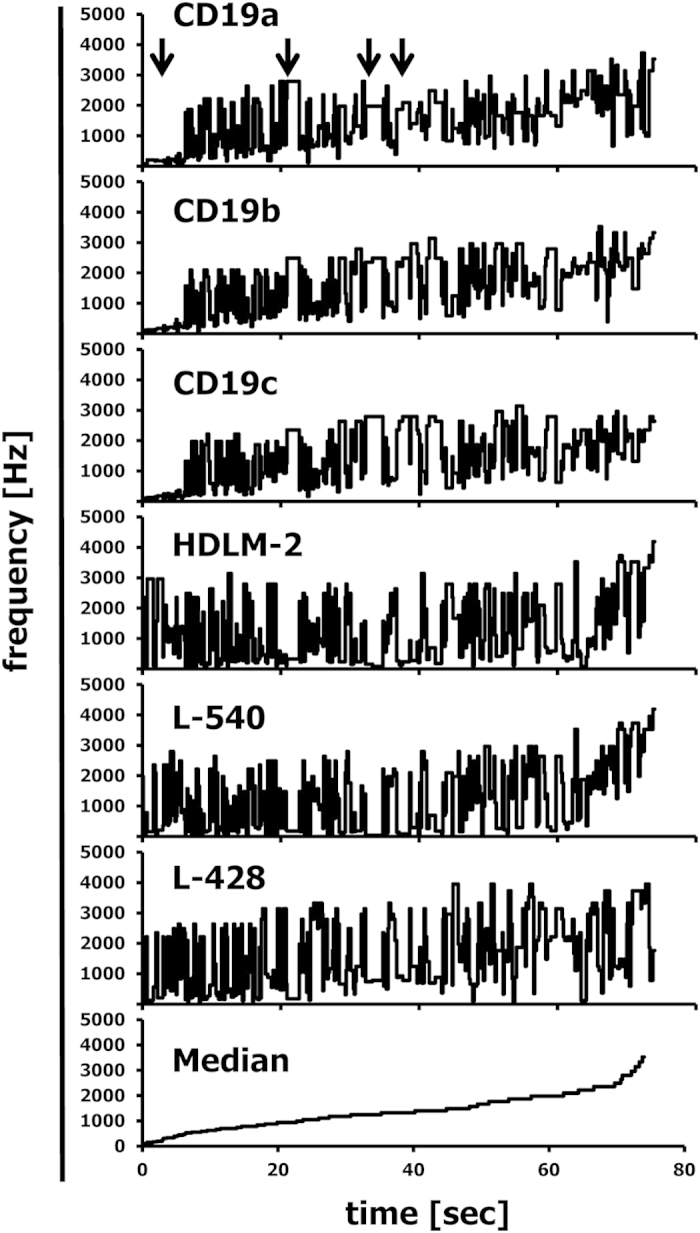
Musical interpretation of differentially expressed probe sets from Hodgkin’s lymphoma cell lines and normal B cells. Affymetrix Human Exon 1.0ST microarray data (extended exon level) from 3 Hodgkin’s lymphoma cell lines^12^ and three CD19-positive B cell samples^13^ were transformed into melodies by using the following parameters: minimal frequency: 27.5; number of different frequencies: 88 (keys); number of tone steps per octave: 12; minimal duration: 1/8; number of tones: 288. Presented are the frequencies of the individual cell lines and the frequency of the median signal intensity as a function of time.

We further tested GEMusicA with a larger dataset derived from Affymetrix Human Exon 1.0ST microarrays (extended exon level with 807,038 probe sets. For an analysis with the complete 1,411,399 probe sets of this array type see the supplementary GEMusicAR.r script). For these experiments we combined data from three Hodgkin’s lymphoma (HL) cell lines^12^ and three samples from normal CD19-positive B cells^13^. We used the 288 (=12 semitone steps × 4 principal lengths × 6 samples) probe sets with highest variance for the transformation of the HL data set into sounds (Fig. 5). The corresponding probe sets are presented in Supplementary Table S4. The melodies are available as IMSLP MP3 files 28CD19aEx288, 29CD19bEx288, 30CD19cEx288, 31HDLM2Ex288, 32L428Ex288, 33L540Ex288, and 34MedHLEx288. Again, we generated stereo versions with the median as reference base line. These versions are available as IMSLP MP3 files 35CD19aEx288st, 36CD19bEx288st, 37CD19cEx288st, 38HDLM2Ex288st, 39L428Ex288st, and 40L540Ex288st. The melodies of the CD19-positive B cells show several characteristic motifs (marked with arrows in Fig. 5) which are absent in the HL samples. It is well-known that HL cells are characterized by the absence of typical B cell markers. In addition, the three HL cell lines are highly heterogeneous^14^,^15^. This heterogeneity is also present in the sound-transformed data. Nevertheless, the differences between the melodies from normal B cells and HL cell lines are obvious and especially pronounced at the beginning of the melodies (see Supplementary Fig. S3 for the first 8 seconds). The heterogeneity is not a consequence of the large number of probe sets used as evidenced by the fact that the same behaviour is present if the arrays were analysed at the core gene level (22,011 probe sets; see Supplementary Fig. S4, Supplementary Table S5, and the corresponding IMSLP MP3 files).

**Figure 6:**
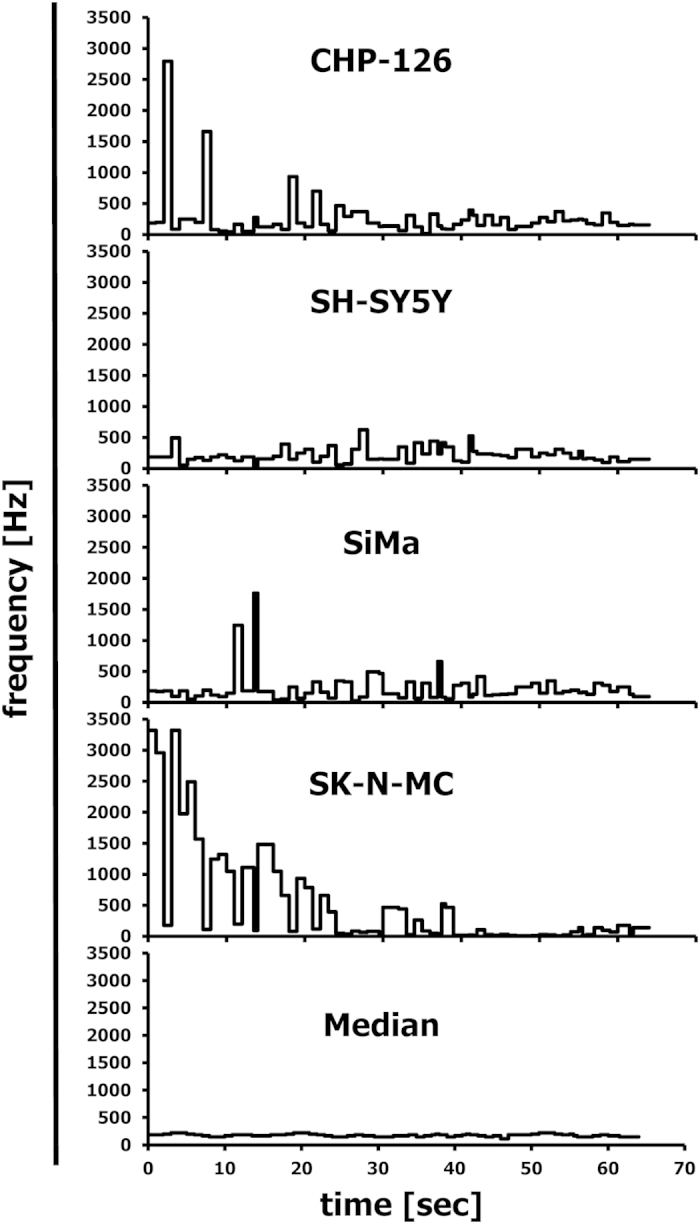
Musical interpretation of top differentially expressed probe sets from “neuroblastoma” cell lines (Song of Joy). DNA microarray data from 4 cell lines that were initially established as neuroblastoma cell lines (GSE1824) were transformed into melodies by using the following parameters: minimal frequency: 27.5; number of different frequencies: 88; number of tone steps per octave: 12; minimal duration: 1/8; number of tones: 63. Probe sets were sorted ascending according to the calculated frequencies from the median signal intensities. Thereafter, Beethoven’s “Song of Joy” from the 9^th^ Symphony was used for re-calibration of the frequencies. Presented are the frequencies of the individual cell lines and the frequency of the median signal intensity as a function of time.

Despite filtering and re-sorting of the probe sets, the resulting melodies in the presented examples are quite abstract and the recall-value is difficult to predict. It seems likely that familiarity with such melodies is achieved faster if dissonances from known melodies are heard. Therefore, we asked whether it is possible to use more conventional melodies for re-calibration of the transformed microarray data. As a first template, we used L. van Beethoven’s “Song of Joy” from the 9^th^ symphony and rescaled the neuroblastoma cell line frequencies as described in the Methods section. The first 63 notes from the melody that include the complete theme were used. The corresponding probe set information is presented in Supplementary Table S6. The melodies are available as IMSLP MP3 files 54CHPSoJ, 55SIMASoJ, 56SHSY5YSoJ, 57SKNMCSoJ, and 58MedSRBCTSoJ. Stereo files with the median (original melody) as reference baseline are available as IMSLP MP3 files 59CHPSoJst, 60SIMASoJst, 61SHSY5YSoJst, and 62SKNMCSoJst. After this transformation the cell line-specific gene expression profiles were still present and the higher divergence of SK-N-MC cells from the median was also evident (Fig. 6). Similar results were obtained with other templates, *e.g.* Wagner’s “Ride of the Valkyries” (Supplementary Fig. 5. The corresponding 86 probe set data from this theme are presented in Supplementary Table S7). The melodies are available as IMSLP MP3 files 63CHPValkyrie, 64SIMAValkyrie, 65SHSY5YValkyrie, 66SKNMCValkyrie, and 67MedSRBCTValkyrie. Stereo files with the median (original melody) as reference baseline are available as IMSLP MP3 files 68CHPValkyriest, 69SIMAValkyriest, 70SHSY5YValkyriest, and 71SKNMCValkyriest. The GEMusicAR.r script (see Supplementary Information) includes additional melody models.

The presented algorithm allows the transformation of gene expression data into sounds. In the present paper we demonstrated only one principle transformation code. This code can easily be changed, *e.g.* by using alternative scales (quarter-tone scale, whole-tone scale) or changing the tune of the “instrument” (*i.e.* by changing the frequency range). In addition, it is not necessary that high differences in the signal intensity are transformed into high differences in the frequency. One alternative possibility might be based on the clock of keys that takes into account that the human ear perceives some intervals as dissonant intervals and others as not. Furthermore, it is possible to play different melodies from different samples at the same time point. In some of the supplementary audio files, the median is played together with one melody. The median can be exchanged by one single sample or a set of other samples. Especially in this case, dissonances are more interesting than simple frequency differences. The Supplementary audio files can be used for the free combination of the included samples, *e.g.* by using ACID Xpress (see Methods section).

What is the practical use of GEMusicA? Aside from the fact that GEMusicA can be considered as a new tool for the composition of musical pieces (in principle it is possible to generate an individual melody from each person who donated some RNA for the generation of a microarray data set) and in addition to the possibility that people with visual impairments can “hear” gene expression, GEMusicA can be used for the analysis of differential gene expression. All probe sets of the 192 variance filtered probe sets form the neuroblastoma/Ewing sarcoma data set (Fig. 1) have frequencies above 987 Hz in at least one sample (see Supplementary Table S2). 85, 48, 32, and 27 of these probe sets have frequencies above 987 Hz in exactly one, two, three, or four cell lines, respectively. From the 32 probe sets that have high pitches in three samples, only 5 have high pitches in SK-N-MC cells (Supplementary Fig. S6). The remaining 27 probe sets represent a neuroblastoma specific signature (in this data set). Interestingly, only 20 probe sets of the 192 variance filtered probe sets have a duration of 0.25 or longer. Seven of these 20 probe sets are high pitched (with a frequency above 987 Hz) only in SK-N-MC cells and 2 only in SiMa cells. All other probe sets are high pitched in at least two samples (see Supplementary Table S2). Obviously, the specific gene expression signature of the SK-N-MC sample is present in the high-pitched notes with long duration which are likely to be noticed by the human ear more easily.

**Figure 7:**
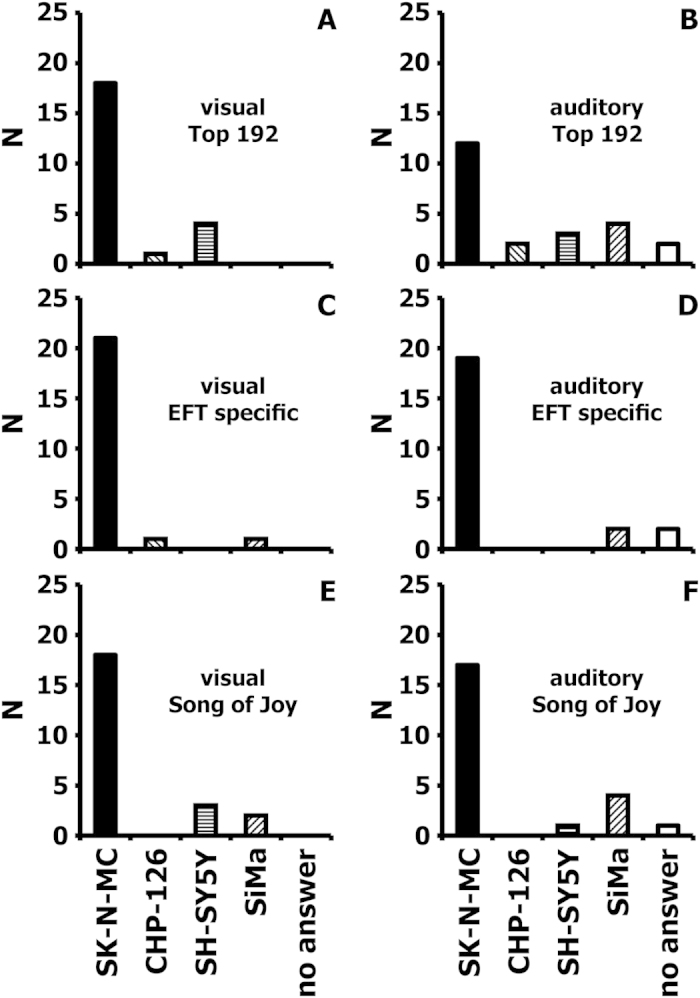
Comparison between the visual and auditory identification of outliers in the “neuroblastoma” data set. Transformation of microarray data into sounds was performed as described in the legends to Figs 1,3 and 6. 23 scientists were asked to identify the outlier among the 4 samples. The following material was presented to the individuals: (**A)** The cell line specific time courses from Fig. 1A; (**B**) The melodies 13SKNMC192, 10CHP192, 11SHSY5Y192, and 12SIMA192 that are based on the frequencies from Fig. 1A; (**C**) The cell line specific time courses from Fig. 3A; (**D**) The melodies 19CHPEFTspec, 20SHSY5YEFTspec, 21SIMAEFTspec, and 22SKNMCEFTspec that are based on the frequencies from Fig. 3A. (**E**) The cell line specific time courses from Fig. 6; (**F**) The melodies 55SHSY5YSoJ, 56SIMASoJ, 57SKNMCSoJ, 54CHPEFTSoJ that are based on the frequencies from Fig. 6. For A, B, and C the individuals had to draw the decision after approximately 2 minutes. For D, E, and F each melody was presented one times and, if requested, one second time. If no cell line was identified, “no answer” could be chosen by the interviewed persons. Presented are the numbers of interviewed persons that voted for the indicated cell lines. The original answers are presented as Supplementary Table S8. The melodies are presented as Supplementary Audio Files.

We interviewed 23 scientists (master students, PhD students, Postdocs) and asked them to identify the outlier in the neuroblastoma/Ewing sarcoma derived diagrams (Figs 1A, 3A and 6) or the corresponding melodies (these melodies are included in the Supplementary Information). The results are presented in Fig. 7. Using the 192 probe sets with highest variance from Fig. 1A, most individuals recognized SK-N-MC cells as outlier on the basis of the diagrams (Fig. 7A) whereas only half of them were able to identify the outlier on the basis of the sounds (Fig. 7B). However, this difference is statistically not significant (p > 0.2; McNemar test) and the difference is not visible after pre-filtering the data (Fig. 7C *versus* Fig. 7D) or after re-calibrating the data by using Beethoven’s “Song of Joy” (Fig. 7E *versus* Fig. 7F). In these cases the graphical versions (Fig. 7C,E) and the sound versions (Fig. 7D,F) allowed identification of the outlier with similar precision. In the past, DNA and protein sequence information has been successfully converted into music with the idea of identifying audible patterns^16^,^17^. In contrast to linear sequence data that can be transformed into melodies, GEMusicA was developed for the comparative analysis of multidimensional gene expression data from multiple samples. The example of the neuroblastoma data set demonstrates that it is possible to use the transformed data for filtering of differentially expressed genes without prior knowledge of a possible classification. Similarly, the probe sets displayed in the melodies from the HL data set contain several cell line or cell type-specific probe sets that are re-transformed in cell type specific frequency courses. The addition of the graphical presentation of the frequencies or the music scores to the sounds can further increase the perceptibility of the specific features of the melodies/gene expression profiles. The normalization by using well-known model melodies (e.g. the “Song of Joy” or the “Ride of the Valkyries”) can probably facilitate the audibility of differential gene expression even for investigators with “low musicality”. Finding the optimal model melody that gives the best results requires further investigations and might be individually different. The musical approach to discriminate gene expression patterns is not necessarily better than the visual approach. However, music may have recreational or educational values that appeal also to the non-specialist and might complement more formal presentations.

## Methods

### Gene expression analysis

Microarray data from Hodgkin’s lymphoma, neuroblastoma and Ewing sarcoma cell lines as well as biopsies were generated as described^2^,^12^. CEL files are available at the Gene Expression Omnibus (GEO) data base (neuroblastoma/Ewing sarcoma data: accession numbers GSE1824 and GSE1825; Hodgkin’s lymphoma cell lines: accession number: GSE47686). Additional CEL files from CD19-positive B cells^13^ were down-loaded from GSE20200. Primary microarray data were analysed by using Expression Console 1.3.1.187 (Affymetrix, Santa Clara, CA, USA) and Microarray Suite 5.0 algorithm (neuroblastoma/Ewing sarcoma data) or Robust Microarray Algorithm (RMA; Hodgkin’s lymphoma/B cell data). All primary data were log2 transformed with Expression Console. If data were pre-filtered for differentially expressed genes, MAFilter was used^6^. Details about polymerase chain reaction (RT-PCR) and quantitative^18^,^19^,^20^,^21^ RT-PCR are provided in the Supplementary Information.

### Gene Expression Music Algorithm (GEMusicA)

Supplementary Fig. S7 gives a short overview on the complete procedure. For each probe set of a sample GEMusicA transforms the signal intensity into a single tone with a specific frequency. High frequencies represent high signal intensities and *vice versa*. The algorithm was initially developed using Microsoft Excel 2010 in combination with a PERL (ArrayMusic.pl) script for transformation of text files into sounds. An Open Office template including the data from Fig. 3 is included in the Supplementary Information. In addition, the ArrayMusic.pl script is included in the Supplementary Information. All IMSLP audio files were generated with this script. An R script (GEMusicAR.r) for the GEMusicA algorithm is included (together with sample data) as Supplementary Information. This script can be used for the automatic generation of (i) audio examples and (ii) TeX template files for music scores. The R script includes instructions for usage (see Supplementary Information).

In general, signal intensities form microarray experiments can be transformed into several different sounds. For simplicity, we used the frequencies produced by conventional key-board instruments as basis for the transformation process in the present paper. Pressing a key on a keyboard instrument produces a tone with a certain frequency. This frequency can vary on different music instruments according to the tuning of the individual instrument. For instance, the 49^th^ key on a standard piano-forte produces a tone with the frequency of 440 Hz but especially on historical instruments frequencies between approximately 415 Hz and 445 Hz can be heard after pressing this key (which is not necessarily the 44^th^ key on all instruments: for small harpsichords the corresponding key is usually the 34^th^ key). On a standard piano-forte the 37^th^ key produces a tone with the frequency of 220 Hz and the 61^st^ key produces a tone with the frequency 880 Hz. The ratio between 220 and 440 as well as the ratio between 440 and 880 is 1:2. With the exception of these simple intervals (octaves) all other frequencies have to be calculated by approximation. If not otherwise stated, the lowest frequency was set to 27.5 Hz (which represents the lowest key on a standard piano-forte). In classical European music, the octave is divided into 12 semi-tone steps but other divisions are also possible (*e.g.* quarter-tone music or whole-tone scales). If not stated otherwise, the octave was divided into 12 identical semi-tone steps. In GEMusicA, for the calculation of the virtual key *key(ps*_*i*_*,s*_*k*_) for a given probe set *ps*_*i*_ from a sample *s*_*k*_, the maximal signal intensity *SImax(s*_*k*_) of the probe sets from this sample was determined and the number *N* of desired different frequencies (the number of virtual keys) was set arbitrarily. For a full standard piano-forte keyboard *N* is 88. Thereafter, *key(ps*_*i*_*,s*_*k*_) was calculated by dividing the signal intensity by *(SImax(s_k_)/N)* and rounding up to the next whole number. With *fmin* = minimal frequency and *step* = number of tone steps per octave, the frequencies *f(key(ps*_*i*_, *s*_*k*_)) of all probe sets *ps*_*i*_ and all samples *s*_*k*_ were calculated as
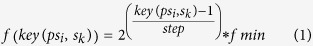


In principle, all probe sets from a given microarray experiment can be transformed into frequencies/keys by GEMusicA. For the intended acoustical presentation the following points have to be considered: 1) the minimal duration of a single tone has to be long enough to allow perception. On the other hand, modern microarrays contain thousands to millions of probe sets; *e.g.*, the neuroblastoma data set that was used in the present paper consists of 22,283 probe sets. With duration of a single tone of 1/10 second, the resulting melody would have a length of approx. 37 minutes. It seems unlikely that differences in the gene expression between different samples can be perceived under these conditions. 2) The informative value is not the same for all probe sets. Probe sets with low variability of the signal intensities are less informative than probe sets with higher variable signal intensities. Therefore, it is reasonable to filter only probe sets with some variability for the transformation into music. Based on these considerations, it seems desirable to adjust the length of the generated tones to the information content. In the present paper, GEMusicA was used to adjust the length of a tone to the variance of the corresponding probe set. For length adaptation, we applied a metrical system that is also used in classical European music. This system is based on the division of a semibreve (whole note) into 2 minims (half notes), 4 crotchets (quarter notes), 8 quavers (eight notes), 16 semiquavers (sixteenth note), 32 demisemiquavers (thirty-second notes) or 64 hemidemisemiquavers (sixty-fourth notes). In the present paper only these notes and dotted version of these notes (one or two dots, increasing the length of a tone by 50% and 75%, respectively) were used. The basic unit for all calculations is the whole note which is transformed into a sound with a length of one second. For calculation of the lengths of the *N* sounds with maximal variance, the variance *s*^*2*^*(ps*_*i*_) of the frequency for each of the *N*+1 probe set with highest variance, the maximal variance *s*^*2*^*max,* and the minimal variance *s*^*2*^*min* for the analysed probe sets were calculated. Thereafter, *s*^*2*^*(ps*_*i*_) was divided by the product of *(s*^*2*^*max-s*^*2*^*min*) and the chosen minimal length. This value was rounded up to the next whole number in order to obtain *val(ps*_*i*_) and the number of minimal-length-units *unit(ps*_*i*_) (*e.g.* hemidemisemiquavers) was calculated as
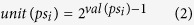


For calculation of dots, *s*^*2*^*(ps*_*i*_) was divided by the product of *s*^*2*^*max/3* and the chosen minimal length. This value was rounded up to the next whole number in order to obtain *da(ps*_*i*_). Thereafter, *dot.a(ps*_*i*_) and *dot.b(ps*_*i*_) were calculated as
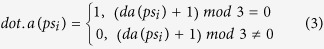
and
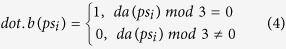
Finally, the complete length of the tone was calculated as
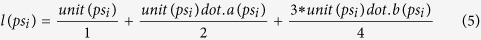


The order of the probe sets in the original data table is not (or not stringently) based on biological or physical parameters. Therefore, after transformation into sounds, differentially expressed genes can be expected to be still arbitrarily spread over the total length of the resulting melody. A universal sort criterion that is independent of knowledge about the data structure is the median. Therefore, the median was used in this paper as the single sort criterion. We calculated the median of the signal intensities of each probe set and transformed these medians into frequencies as described above. Thereafter, we used these new values and sorted all probe sets ascending according to this parameter. With this algorithm probe sets with high expression in outliers compared to the median are preferentially placed on top of the list, and probe sets with low expression in outliers compared to the median are preferentially placed on the bottom of the list.

Despite the adaptation of the length of single tones to the variability, the length of the complete melodies is still high if microarrays with high numbers of probe sets were used. As indicated above, the number of probe sets with high information content can be reduced by filtering for probe sets with high variability. In the present paper we used the variances of the calculated frequencies for selecting probe sets with highest variance.

In some experiments, the frequency-transformed data were re-normalized by using a known melody as reference. For fitting melodies to the reference melody, the number of used probe sets was adjusted to the number of tones in the model. The required number of tones with highest variances was filtered. The signal intensities of these probe sets were transformed into frequencies as described above. After sorting the calculated frequencies ascending according to the median, the new frequencies *f(ps*_*i*_*,med)*_*new*_for the median were set according to the reference melody. Thereafter, the new frequencies *f(ps*_*i*_*,s*_*k*_)_*new*_for the individual samples were calculated from the old *f(ps*_*i*_*,s*_*k*_)_*old*_ as
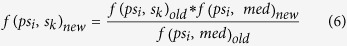


In the present paper L. van Beethoven’s “Song of Joy” from Op. 125 and R. Wagner’s “Ride of the Valkyries” were used as models. The tones for the “Song of Joy” were abstracted from the transcription for piano solo by F. Liszt, Kalmus K09228 edition, Belwin Mills Publishing Corp., Miami, FL, USA, page 196, bars 5–20. The tones for the “Ride of the Valkyries” were abstracted from the trombone part of the Philharmonia pocket score edition No. 123, Wiener Philharmonischer Verlag, Vienna, Austria, pages 25–34, bars 58–75. The duration of the tones was set according to the original reference melody. A PERL script (see below) was used for generation of sounds where a sound of length-unit one has approximately a length of one second. For the model-fitted melodies, the lengths of crotchets were set as one and the other values were calculated accordingly. The R script GEMusicAR.r contains additional melody models.

Example music scores were generated with capella studio version 5.1–06 (capella Software GmbH, Söhrewald, Germany). A short PERL script (compiled with ActivePerl 5.16.3; ActiveState Software Inc., Vancouver, BC, Canada) was used for generation of wavesound files (see Supplementary Information). Stereo files were generated with Acid Xpress 7 (Sony Creative Software Inc., Mittleton, WI). In this case, wavesound files were loaded into an ACID Xpress project and the median was displayed only on one channel whereas the individual samples were displayed only on the other channel. Finally, MP3 stereo files were transformed into wavesound files by using Free Audio Converter 5.0.54 (DVDVideoSoft Ltd, UK). The same software was used for generation of supplementary audio files (see supplementary zip archive). Active Perl, Free Audio Converter as well as ACID Xpress are available from the internet. MP3 versions of all music examples in this manuscript have been submitted to the International Music Score Library Project (IMSLP, “Tumour Music”) http://imslp.org/wiki/Tumour_Music_%28Staege,_Martin_Sebastian%29.

## Additional Information

**How to cite this article**: Staege, M. S. A short treatise concerning a musical approach for the interpretation of gene expression data. *Sci. Rep.* **5**, 15281; doi: 10.1038/srep15281 (2015).

## Supplementary Material

Supplementary Information

Supplementary Audio

Supplementary Data
