# Supplementary material for: A short treatise concerning a musical approach for the interpretation of gene expression data
Source: Sci Rep. 2015 Oct 16;5:15281. doi: 10.1038/srep15281 (PMC4607888; doi:10.1038/srep15281)
Supplement: Supplementary Information [file srep15281-s1.pdf]

Supplementary information to:

Staege MS (2015) A short treatise concerning a musical approach for the interpretation of gene expression data.

Content: **Page:**

Supplementary Table S1 (2,228 probe sets)	2
Supplementary Table S2 (192 probe sets)	76
Supplementary Table S3 (Ewing sarcoma-specific probe sets)	85
Supplementary Table S4 (HL versus B cells; probeset level)	91
Supplementary Table S5 (HL versus B cells; gene level)	100
Supplementary Table S6 (Song of Joy)	109
Supplementary Table S7 (Ride of the Valkyries)	112
Supplementary Table S8 (interview results)	116
Supplementary Figure S1	117
Supplementary Figure S2	118
Supplementary Figure S3	119
Supplementary Figure S4	120
Supplementary Figure S5	121
Supplementary Figure S6	122
Supplementary Figure S7	123
Supplementary Information	
A. Pre-filtered probe sets used in Figure 3	124
B. Gene expression analysis	125
C. GEMusicAR: R script for GEMusicA	126
C. ArrayMusic: PERL script used for generation of wavesound files	146
D. Index of Supplementary audio files	147

Supplementary Table S1.

Frequencies of the musically interpreted microarray data from "neuroblastoma" cell lines (2,228 probe sets). DNA microarray data from 4 cell lines that were initially established as neuroblastoma cell lines (GSE1824) were transformed into melodies as described in the Methods section by using the following parameters: minimal frequency: 27.5; number of different frequencies (keys): 88; number of tone steps per octave: 12; minimal duration: 1/8; number of tones: 2,228. Presented are the frequencies of the individual cell lines and the frequency of the median signal intensity as well as the duration of the filtered 2,228 probe sets.

**Probe Set ID**	**Gene Symbol**	**Median**	**CHP-126**	**SH-SY5Y**	**SiMa**	**SK-N-MC**	**Duration**
207397_s_at	HOXD13	41.20344461	43.65352893	41.20344461	36.70809599	523.2511306	0.125
203964_at	NMI	51.9130872	55	48.9994295	48.9994295	440	0.125
219355_at	CXorf57	58.27047019	61.73541266	41.20344461	55	493.8833013	0.125
202820_at	AHR	61.73541266	34.64782887	77.78174593	43.65352893	415.3046976	0.125
205478_at	PPP1R1A	61.73541266	46.24930284	65.40639133	58.27047019	1108.730524	0.1875
209844_at	HOXB13	65.40639133	65.40639133	61.73541266	69.29565774	554.365262	0.125
220994_s_at	STXBP6	65.40639133	415.3046976	69.29565774	65.40639133	61.73541266	0.125
214530_x_at	EPB41	69.29565774	69.29565774	65.40639133	65.40639133	466.1637615	0.125
200951_s_at	CCND2	73.41619198	110	46.24930284	48.9994295	622.2539674	0.125
206070_s_at	EPHA3	73.41619198	61.73541266	77.78174593	58.27047019	659.2551138	0.125
207238_s_at	PTPRC	73.41619198	55	73.41619198	73.41619198	659.2551138	0.125
209656_s_at	TMEM47	73.41619198	51.9130872	103.8261744	43.65352893	987.7666025	0.125
216598_s_at	CCL2	73.41619198	97.998859	622.2539674	55	58.27047019	0.125
219090_at	SLC24A3	73.41619198	65.40639133	61.73541266	87.30705786	587.3295358	0.125
204456_s_at	GAS1	77.78174593	73.41619198	82.40688923	73.41619198	622.2539674	0.125
205872_x_at	PDE4DIP	77.78174593	65.40639133	82.40688923	73.41619198	466.1637615	0.125
206114_at	EPHA4	77.78174593	61.73541266	69.29565774	87.30705786	554.365262	0.125
209655_s_at	TMEM47	77.78174593	82.40688923	77.78174593	77.78174593	493.8833013	0.125
204971_at	CSTA	82.40688923	97.998859	55	69.29565774	415.3046976	0.125
205081_at	CRIP1	82.40688923	61.73541266	77.78174593	87.30705786	1046.502261	0.1875
205629_s_at	CRH	82.40688923	523.2511306	69.29565774	103.8261744	58.27047019	0.125
206135_at	ST18	82.40688923	130.8127827	43.65352893	932.327523	51.9130872	0.125
219825_at	CYP26B1	82.40688923	65.40639133	103.8261744	65.40639133	587.3295358	0.125
203567_s_at	TRIM38	87.30705786	97.998859	82.40688923	82.40688923	493.8833013	0.125
204379_s_at	FGFR3	87.30705786	92.49860568	77.78174593	73.41619198	830.6093952	0.125

213395_at	MLC1	87.30705786	130.8127827	55	55	587.3295358	0.125
206326_at	GRP	92.49860568	92.49860568	92.49860568	92.49860568	1661.21879	0.25
207149_at	CDH12	92.49860568	146.832384	43.65352893	55	554.365262	0.125
207811_at	KRT12	92.49860568	61.73541266	130.8127827	46.24930284	880	0.125
212909_at	LYPD1	92.49860568	97.998859	87.30705786	69.29565774	932.327523	0.125
203324_s_at	CAV2	97.998859	55	184.9972114	32.70319566	698.4564629	0.125
208965_s_at	IFI16	97.998859	58.27047019	73.41619198	123.4708253	493.8833013	0.125
210103_s_at	FOXA2	97.998859	82.40688923	110	830.6093952	65.40639133	0.125
211675_s_at	MDFIC	97.998859	116.5409404	82.40688923	58.27047019	987.7666025	0.125
202458_at	PRSS23	103.8261744	123.4708253	622.2539674	82.40688923	82.40688923	0.125
202628_s_at	SERPINE1	103.8261744	103.8261744	587.3295358	92.49860568	97.998859	0.125
205000_at	DDX3Y	103.8261744	55	69.29565774	466.1637615	146.832384	0.125
205306_x_at	KMO	103.8261744	73.41619198	92.49860568	110	987.7666025	0.125
205372_at	PLAG1	103.8261744	207.6523488	55	55	587.3295358	0.125
208096_s_at	COL21A1	103.8261744	65.40639133	130.8127827	77.78174593	587.3295358	0.125
208966_x_at	IFI16	103.8261744	36.70809599	138.5913155	73.41619198	523.2511306	0.125
209360_s_at	LOC100506403 /// RUNX1	103.8261744	110	87.30705786	493.8833013	97.998859	0.125
219686_at	STK32B	103.8261744	110	92.49860568	97.998859	523.2511306	0.125
219973_at	ARSJ	103.8261744	130.8127827	440	77.78174593	55	0.125
220445_s_at	CSAG2 /// CSAG3	103.8261744	207.6523488	466.1637615	43.65352893	51.9130872	0.125
201161_s_at	CSDA	110	146.832384	77.78174593	77.78174593	880	0.125
202747_s_at	ITM2A	110	116.5409404	110	103.8261744	1760	0.25
204818_at	HSD17B2	110	87.30705786	65.40639133	138.5913155	739.9888454	0.125
205857_at	SLC18A2	110	130.8127827	87.30705786	698.4564629	48.9994295	0.125
209540_at	IGF1	110	73.41619198	92.49860568	130.8127827	622.2539674	0.125
219360_s_at	TRPM4	110	123.4708253	97.998859	55	783.990872	0.125
220551_at	SLC17A6	110	123.4708253	77.78174593	659.2551138	97.998859	0.125
40284_at	FOXA2	110	65.40639133	87.30705786	880	130.8127827	0.125
205919_at	HBE1	116.5409404	659.2551138	103.8261744	123.4708253	92.49860568	0.125
206812_at	ADRB3	116.5409404	123.4708253	110	82.40688923	739.9888454	0.125
213236_at	SASH1	116.5409404	97.998859	130.8127827	92.49860568	587.3295358	0.125
214479_at	GFRA3	116.5409404	87.30705786	123.4708253	739.9888454	116.5409404	0.125
222001_x_at	LOC728855 /// LOC728875	116.5409404	77.78174593	164.8137785	65.40639133	493.8833013	0.125
202627_s_at	SERPINE1	123.4708253	110	554.365262	97.998859	138.5913155	0.125

206463_s_at	DHRS2	123.4708253	1760	116.5409404	116.5409404	110	0.25
206631_at	PTGER2	123.4708253	55	261.6255653	587.3295358	46.24930284	0.125
206756_at	CHST7	123.4708253	87.30705786	164.8137785	440	69.29565774	0.125
206915_at	NKX2-2	123.4708253	58.27047019	82.40688923	164.8137785	987.7666025	0.125
212097_at	CAV1	123.4708253	48.9994295	277.182631	55	1864.655046	0.25
214460_at	LSAMP	123.4708253	92.49860568	164.8137785	82.40688923	554.365262	0.125
201601_x_at	IFITM1 ///<br>IFITM2	130.8127827	82.40688923	195.997718	82.40688923	698.4564629	0.125
206502_s_at	INSM1	130.8127827	880	58.27047019	261.6255653	61.73541266	0.125
208235_x_at	GAGE12F ///<br>GAGE12G ///<br>GAGE12I ///<br>GAGE4 ///<br>GAGE5 ///<br>GAGE6 ///<br>GAGE7	130.8127827	659.2551138	184.9972114	92.49860568	87.30705786	0.125
218796_at	FERMT1	130.8127827	130.8127827	138.5913155	92.49860568	739.9888454	0.125
218899_s_at	BAALC	130.8127827	123.4708253	73.41619198	587.3295358	138.5913155	0.125
221606_s_at	HMGN5	130.8127827	138.5913155	69.29565774	123.4708253	466.1637615	0.125
201718_s_at	EPB41L2	138.5913155	184.9972114	97.998859	87.30705786	783.990872	0.125
202291_s_at	MGP	138.5913155	82.40688923	698.4564629	87.30705786	220	0.125
204005_s_at	PAWR	138.5913155	195.997718	97.998859	34.64782887	440	0.125
206373_at	ZIC1	138.5913155	61.73541266	622.2539674	55	311.1269837	0.125
206640_x_at	GAGE12B ///<br>GAGE12C ///<br>GAGE12D ///<br>GAGE12E ///<br>GAGE12F ///<br>GAGE12G ///<br>GAGE12H ///<br>GAGE12I ///<br>GAGE2A ///<br>GAGE2B ///<br>GAGE2C ///<br>GAGE4 ///<br>GAGE5 ///	138.5913155	698.4564629	110	92.49860568	164.8137785	0.125

	GAGE6 /// GAGE7						
207398_at	HOXD13	138.5913155	233.0818808	82.40688923	77.78174593	554.365262	0.125
207663_x_at	GAGE3	138.5913155	739.9888454	123.4708253	87.30705786	164.8137785	0.125
218870_at	ARHGAP15	138.5913155	123.4708253	77.78174593	155.5634919	587.3295358	0.125
219371_s_at	KLF2	138.5913155	195.997718	87.30705786	92.49860568	493.8833013	0.125
200660_at	S100A11	146.832384	65.40639133	311.1269837	51.9130872	466.1637615	0.125
201427_s_at	SEPP1	146.832384	174.6141157	61.73541266	130.8127827	1046.502261	0.125
201744_s_at	LUM	146.832384	55	369.9944227	41.20344461	440	0.125
202133_at	WWTR1	146.832384	61.73541266	329.6275569	41.20344461	587.3295358	0.125
202404_s_at	COL1A2	146.832384	138.5913155	146.832384	51.9130872	1244.507935	0.1875
204844_at	ENPEP	146.832384	77.78174593	233.0818808	97.998859	466.1637615	0.125
205858_at	NGFR	146.832384	103.8261744	207.6523488	103.8261744	554.365262	0.125
206247_at	MICB	146.832384	130.8127827	77.78174593	164.8137785	587.3295358	0.125
208334_at	NDST4	146.832384	123.4708253	82.40688923	174.6141157	523.2511306	0.125
209129_at	TRIP6	146.832384	138.5913155	164.8137785	77.78174593	932.327523	0.125
210303_at	MAB21L2	146.832384	587.3295358	55	146.832384	146.832384	0.125
211377_x_at	MYCN	146.832384	415.3046976	48.9994295	466.1637615	51.9130872	0.125
211828_s_at	TNIK	146.832384	493.8833013	277.182631	65.40639133	82.40688923	0.125
219471_at	KIAA0226L	146.832384	97.998859	207.6523488	92.49860568	587.3295358	0.125
201160_s_at	CSDA	155.5634919	174.6141157	38.89087297	130.8127827	1396.912926	0.1875
201189_s_at	ITPR3	155.5634919	87.30705786	277.182631	87.30705786	554.365262	0.125
203881_s_at	DMD	155.5634919	73.41619198	174.6141157	523.2511306	146.832384	0.125
205186_at	DNALI1	155.5634919	77.78174593	311.1269837	391.995436	41.20344461	0.125
205440_s_at	NPY1R	155.5634919	246.9416506	97.998859	61.73541266	783.990872	0.125
205771_s_at	AKAP7	155.5634919	174.6141157	146.832384	77.78174593	739.9888454	0.125
205827_at	CCK	155.5634919	195.997718	116.5409404	41.20344461	1975.533205	0.375
206584_at	LY96	155.5634919	130.8127827	174.6141157	92.49860568	987.7666025	0.125
209278_s_at	TFPI2	155.5634919	55	830.6093952	246.9416506	97.998859	0.125
209541_at	IGF1	155.5634919	123.4708253	184.9972114	82.40688923	783.990872	0.125
217549_at	---	155.5634919	155.5634919	87.30705786	155.5634919	493.8833013	0.125
220340_at	GREB1L	155.5634919	466.1637615	92.49860568	220	103.8261744	0.125
220510_at	RHBG	155.5634919	466.1637615	174.6141157	130.8127827	69.29565774	0.125
201188_s_at	ITPR3	164.8137785	116.5409404	233.0818808	87.30705786	466.1637615	0.125
201288_at	ARHGDIB	164.8137785	87.30705786	329.6275569	58.27047019	493.8833013	0.125
201719_s_at	EPB41L2	164.8137785	130.8127827	73.41619198	220	880	0.125

204049_s_at	PHACTR2	164.8137785	69.29565774	554.365262	138.5913155	195.997718	0.125
205137_x_at	USH1C	164.8137785	523.2511306	123.4708253	146.832384	174.6141157	0.125
205154_at	LRRN2	164.8137785	123.4708253	220	466.1637615	69.29565774	0.125
206332_s_at	IFI16	164.8137785	146.832384	41.20344461	184.9972114	523.2511306	0.125
207739_s_at	GAGE1 /// GAGE12C /// GAGE12D /// GAGE12E /// GAGE12F /// GAGE12G /// GAGE12H /// GAGE12I /// GAGE12J /// GAGE2A /// GAGE2B /// GAGE2C /// GAGE2D /// GAGE2E /// GAGE3 /// GAGE4 /// GAGE5 /// GAGE6 /// GAGE7 /// GAGE8	164.8137785	554.365262	87.30705786	155.5634919	174.6141157	0.125
209183_s_at	C10orf10	164.8137785	65.40639133	277.182631	523.2511306	92.49860568	0.125
210090_at	ARC	164.8137785	493.8833013	116.5409404	130.8127827	195.997718	0.125
212588_at	PTPRC	164.8137785	164.8137785	130.8127827	155.5634919	783.990872	0.125
215990_s_at	BCL6	164.8137785	174.6141157	138.5913155	164.8137785	659.2551138	0.125
218182_s_at	CLDN1	164.8137785	77.78174593	130.8127827	207.6523488	493.8833013	0.125
221215_s_at	RIPK4	164.8137785	110	207.6523488	116.5409404	622.2539674	0.125
202746_at	ITM2A	174.6141157	184.9972114	164.8137785	97.998859	1479.977691	0.21875
202771_at	PIEZO1	174.6141157	65.40639133	369.9944227	82.40688923	523.2511306	0.125
204048_s_at	PHACTR2	174.6141157	51.9130872	554.365262	220	146.832384	0.125
204518_s_at	PPIC	174.6141157	233.0818808	466.1637615	48.9994295	138.5913155	0.125
205227_at	IL1RAP	174.6141157	103.8261744	155.5634919	195.997718	1108.730524	0.1875
205249_at	EGR2	174.6141157	97.998859	246.9416506	130.8127827	659.2551138	0.125

205290_s_at	BMP2	174.6141157	92.49860568	246.9416506	116.5409404	587.3295358	0.125
205618_at	PRRG1	174.6141157	220	130.8127827	87.30705786	523.2511306	0.125
205968_at	KCNS3	174.6141157	329.6275569	82.40688923	1174.659072	87.30705786	0.1875
206176_at	BMP6	174.6141157	233.0818808	138.5913155	116.5409404	493.8833013	0.125
208893_s_at	DUSP6	174.6141157	110	523.2511306	293.6647679	82.40688923	0.125
209292_at	ID4	174.6141157	138.5913155	220	123.4708253	587.3295358	0.125
209542_x_at	IGF1	174.6141157	110	110	261.6255653	880	0.125
209734_at	NCKAP1L	174.6141157	130.8127827	220	130.8127827	1244.507935	0.1875
210274_at	MAGEA8	174.6141157	155.5634919	195.997718	554.365262	58.27047019	0.125
211577_s_at	IGF1	174.6141157	293.6647679	103.8261744	103.8261744	554.365262	0.125
214603_at	MAGEA2 /// MAGEA2B	174.6141157	349.2282314	587.3295358	82.40688923	58.27047019	0.125
221558_s_at	LEF1	174.6141157	82.40688923	174.6141157	174.6141157	587.3295358	0.125
201301_s_at	ANXA4	184.9972114	61.73541266	523.2511306	77.78174593	415.3046976	0.125
202619_s_at	PLOD2	184.9972114	246.9416506	138.5913155	146.832384	698.4564629	0.125
202765_s_at	FBN1	184.9972114	155.5634919	659.2551138	207.6523488	97.998859	0.125
203323_at	CAV2	184.9972114	195.997718	174.6141157	123.4708253	739.9888454	0.125
203786_s_at	TPD52L1	184.9972114	123.4708253	207.6523488	155.5634919	554.365262	0.125
204198_s_at	RUNX3	184.9972114	207.6523488	164.8137785	65.40639133	987.7666025	0.125
204345_at	COL16A1	184.9972114	184.9972114	77.78174593	184.9972114	622.2539674	0.125
204951_at	RHOH	184.9972114	207.6523488	110	164.8137785	698.4564629	0.125
205641_s_at	TRADD	184.9972114	207.6523488	523.2511306	130.8127827	164.8137785	0.125
206645_s_at	NR0B1	184.9972114	233.0818808	77.78174593	138.5913155	783.990872	0.125
206935_at	PCDH8	184.9972114	493.8833013	123.4708253	138.5913155	261.6255653	0.125
207712_at	BAGE	184.9972114	523.2511306	164.8137785	207.6523488	146.832384	0.125
209160_at	AKR1C3	184.9972114	739.9888454	415.3046976	82.40688923	58.27047019	0.125
209220_at	GPC3	184.9972114	184.9972114	523.2511306	174.6141157	123.4708253	0.125
209355_s_at	PPAP2B	184.9972114	82.40688923	391.995436	415.3046976	69.29565774	0.125
209756_s_at	MYCN	184.9972114	698.4564629	61.73541266	554.365262	61.73541266	0.125
210029_at	IDO1	184.9972114	103.8261744	123.4708253	293.6647679	554.365262	0.125
211138_s_at	KMO	184.9972114	246.9416506	138.5913155	103.8261744	1046.502261	0.125
214079_at	DHRS2	184.9972114	1864.655046	146.832384	220	123.4708253	0.25
214453_s_at	IFI44	184.9972114	174.6141157	195.997718	174.6141157	523.2511306	0.125
218468_s_at	GREM1	184.9972114	174.6141157	622.2539674	164.8137785	207.6523488	0.125
219523_s_at	ODZ3	184.9972114	77.78174593	440	466.1637615	73.41619198	0.125
219610_at	RGNEF	184.9972114	659.2551138	349.2282314	97.998859	97.998859	0.125

220136_s_at	CRYBA2	184.9972114	369.9944227	97.998859	587.3295358	58.27047019	0.125
221911_at	ETV1	184.9972114	77.78174593	220	622.2539674	155.5634919	0.125
222108_at	AMIGO2	184.9972114	146.832384	97.998859	987.7666025	246.9416506	0.125
1255_g_at	GUCA1A	195.997718	130.8127827	466.1637615	293.6647679	87.30705786	0.125
200606_at	DSP	195.997718	164.8137785	220	164.8137785	659.2551138	0.125
200952_s_at	CCND2	195.997718	174.6141157	73.41619198	220	698.4564629	0.125
202620_s_at	PLOD2	195.997718	261.6255653	103.8261744	146.832384	698.4564629	0.125
202855_s_at	SLC16A3	195.997718	58.27047019	130.8127827	277.182631	587.3295358	0.125
202992_at	C7	195.997718	97.998859	1046.502261	87.30705786	369.9944227	0.125
203698_s_at	FRZB	195.997718	659.2551138	261.6255653	146.832384	73.41619198	0.125
204197_s_at	RUNX3	195.997718	146.832384	73.41619198	261.6255653	830.6093952	0.125
204249_s_at	LMO2	195.997718	184.9972114	207.6523488	130.8127827	698.4564629	0.125
205126_at	VRK2	195.997718	349.2282314	110	73.41619198	440	0.125
205935_at	FOXF1	195.997718	659.2551138	174.6141157	220	138.5913155	0.125
207086_x_at	GAGE1 /// GAGE12B /// GAGE12C /// GAGE12D /// GAGE12E /// GAGE12F /// GAGE12G /// GAGE12H /// GAGE12I /// GAGE12J /// GAGE2A /// GAGE2B /// GAGE2C /// GAGE2D /// GAGE2E /// GAGE4 /// GAGE5 /// GAGE6 /// GAGE7 /// GAGE8	195.997718	830.6093952	233.0818808	164.8137785	110	0.125
210227_at	DLGAP2	195.997718	523.2511306	164.8137785	220	69.29565774	0.125
210786_s_at	FLI1	195.997718	155.5634919	174.6141157	233.0818808	554.365262	0.125

213479_at	NPTX2	195.997718	61.73541266	880	87.30705786	440	0.125
218469_at	GREM1	195.997718	311.1269837	622.2539674	110	123.4708253	0.125
218642_s_at	CHCHD7	195.997718	130.8127827	277.182631	116.5409404	698.4564629	0.125
219389_at	SUSD4	195.997718	261.6255653	103.8261744	554.365262	138.5913155	0.125
221664_s_at	F11R	195.997718	97.998859	415.3046976	97.998859	391.995436	0.125
221730_at	COL5A2	195.997718	146.832384	261.6255653	155.5634919	622.2539674	0.125
202310_s_at	COL1A1	207.6523488	87.30705786	466.1637615	38.89087297	493.8833013	0.125
202403_s_at	COL1A2	207.6523488	110	261.6255653	164.8137785	1760	0.25
202446_s_at	PLSCR1	207.6523488	184.9972114	233.0818808	195.997718	622.2539674	0.125
202719_s_at	TES	207.6523488	51.9130872	523.2511306	82.40688923	523.2511306	0.125
203638_s_at	FGFR2	207.6523488	130.8127827	311.1269837	554.365262	61.73541266	0.125
204683_at	ICAM2	207.6523488	233.0818808	184.9972114	622.2539674	82.40688923	0.125
205368_at	FAM131B	207.6523488	493.8833013	77.78174593	261.6255653	155.5634919	0.125
208146_s_at	CPVL	207.6523488	195.997718	207.6523488	174.6141157	587.3295358	0.125
208291_s_at	TH	207.6523488	440	103.8261744	659.2551138	38.89087297	0.125
208534_s_at	RASA4 /// RASA4B /// RASA4CP /// UPK3BL	207.6523488	311.1269837	523.2511306	97.998859	130.8127827	0.125
209791_at	PADI2	207.6523488	146.832384	116.5409404	293.6647679	554.365262	0.125
210123_s_at	CHRFAM7A /// CHRNA7	207.6523488	880	311.1269837	130.8127827	73.41619198	0.125
210756_s_at	NOTCH2	207.6523488	116.5409404	622.2539674	87.30705786	349.2282314	0.125
211222_s_at	HAP1	207.6523488	233.0818808	184.9972114	130.8127827	523.2511306	0.125
213005_s_at	KANK1	207.6523488	174.6141157	233.0818808	92.49860568	659.2551138	0.125
219414_at	CLSTN2	207.6523488	97.998859	184.9972114	233.0818808	587.3295358	0.125
219572_at	CADPS2	207.6523488	220	164.8137785	207.6523488	1174.659072	0.1875
220014_at	PRR16	207.6523488	138.5913155	523.2511306	311.1269837	97.998859	0.125
201645_at	TNC	220	110	554.365262	61.73541266	440	0.125
201887_at	IL13RA1	220	195.997718	523.2511306	110	233.0818808	0.125
202007_at	NID1	220	233.0818808	466.1637615	43.65352893	207.6523488	0.125
202201_at	BLVRB	220	164.8137785	277.182631	103.8261744	659.2551138	0.125
202752_x_at	SLC7A8	220	261.6255653	138.5913155	184.9972114	830.6093952	0.125
203065_s_at	CAV1	220	87.30705786	329.6275569	138.5913155	1975.533205	0.375
203704_s_at	RREB1	220	164.8137785	293.6647679	130.8127827	659.2551138	0.125
204513_s_at	ELMO1	220	329.6275569	587.3295358	138.5913155	82.40688923	0.125

204517_at	PPIC	220	261.6255653	587.3295358	195.997718	174.6141157	0.125
205117_at	FGF1	220	277.182631	174.6141157	493.8833013	73.41619198	0.125
206002_at	GPR64	220	220	207.6523488	51.9130872	493.8833013	0.125
206025_s_at	TNFAIP6	220	246.9416506	195.997718	87.30705786	659.2551138	0.125
207084_at	POU3F2	220	261.6255653	87.30705786	184.9972114	554.365262	0.125
208025_s_at	HMGA2	220	233.0818808	195.997718	87.30705786	587.3295358	0.125
208885_at	LCP1	220	123.4708253	246.9416506	184.9972114	783.990872	0.125
209291_at	ID4	220	77.78174593	293.6647679	174.6141157	1396.912926	0.1875
209409_at	GRB10	220	207.6523488	220	220	659.2551138	0.125
210445_at	FABP6	220	783.990872	51.9130872	369.9944227	130.8127827	0.125
211502_s_at	CDK14	220	87.30705786	261.6255653	174.6141157	493.8833013	0.125
212993_at	NACC2	220	87.30705786	277.182631	164.8137785	554.365262	0.125
214604_at	HOXD11	220	261.6255653	164.8137785	146.832384	587.3295358	0.125
214614_at	MNX1	220	587.3295358	184.9972114	246.9416506	164.8137785	0.125
214722_at	NOTCH2NL	220	130.8127827	329.6275569	138.5913155	880	0.125
216222_s_at	MYO10	220	87.30705786	220	207.6523488	554.365262	0.125
217967_s_at	FAM129A	220	155.5634919	311.1269837	138.5913155	659.2551138	0.125
219729_at	PRRX2	220	155.5634919	311.1269837	138.5913155	698.4564629	0.125
219736_at	TRIM36	220	174.6141157	277.182631	92.49860568	554.365262	0.125
220057_at	XAGE1A /// XAGE1B /// XAGE1C /// XAGE1D /// XAGE1E	220	103.8261744	415.3046976	116.5409404	698.4564629	0.125
221173_at	USH1C	220	554.365262	103.8261744	220	233.0818808	0.125
221667_s_at	HSPB8	220	92.49860568	261.6255653	174.6141157	622.2539674	0.125
221966_at	GPR137	220	207.6523488	554.365262	220	207.6523488	0.125
200953_s_at	CCND2	233.0818808	277.182631	164.8137785	195.997718	1396.912926	0.1875
201012_at	ANXA1	233.0818808	61.73541266	246.9416506	207.6523488	1479.977691	0.21875
201331_s_at	STAT6	233.0818808	369.9944227	123.4708253	155.5634919	783.990872	0.125
201438_at	COL6A3	233.0818808	293.6647679	174.6141157	87.30705786	659.2551138	0.125
201909_at	RPS4Y1	233.0818808	207.6523488	155.5634919	1567.981744	246.9416506	0.21875
203140_at	BCL6	233.0818808	233.0818808	220	184.9972114	987.7666025	0.125
203824_at	TSPAN8	233.0818808	82.40688923	830.6093952	174.6141157	311.1269837	0.125
203921_at	CHST2	233.0818808	587.3295358	220	174.6141157	220	0.125
204389_at	MAOA	233.0818808	493.8833013	329.6275569	164.8137785	82.40688923	0.125

204653_at	TFAP2A	233.0818808	207.6523488	184.9972114	261.6255653	587.3295358	0.125
204948_s_at	FST	233.0818808	587.3295358	220	195.997718	246.9416506	0.125
205030_at	FABP7	233.0818808	246.9416506	207.6523488	195.997718	587.3295358	0.125
205379_at	CBR3	233.0818808	82.40688923	195.997718	277.182631	587.3295358	0.125
205547_s_at	TAGLN	233.0818808	174.6141157	1108.730524	138.5913155	277.182631	0.125
205613_at	SYT17	233.0818808	391.995436	493.8833013	130.8127827	103.8261744	0.125
206026_s_at	TNFAIP6	233.0818808	220	233.0818808	155.5634919	622.2539674	0.125
206089_at	NELL1	233.0818808	830.6093952	329.6275569	123.4708253	146.832384	0.125
206315_at	CRLF1	233.0818808	415.3046976	58.27047019	130.8127827	493.8833013	0.125
206461_x_at	MT1H	233.0818808	123.4708253	440	110	466.1637615	0.125
207028_at	MYCNOS	233.0818808	987.7666025	116.5409404	466.1637615	69.29565774	0.125
207074_s_at	SLC18A1	233.0818808	207.6523488	261.6255653	987.7666025	220	0.125
208155_x_at	GAGE1 /// GAGE12C /// GAGE12D /// GAGE12E /// GAGE12F /// GAGE12G /// GAGE12H /// GAGE12I /// GAGE12J /// GAGE2D /// GAGE4 /// GAGE5 /// GAGE6 /// GAGE7	233.0818808	830.6093952	195.997718	116.5409404	277.182631	0.125
208789_at	PTRF	233.0818808	92.49860568	659.2551138	103.8261744	493.8833013	0.125
209869_at	ADRA2A	233.0818808	493.8833013	277.182631	69.29565774	207.6523488	0.125
212667_at	LOC100505813 /// SPARC	233.0818808	311.1269837	523.2511306	110	174.6141157	0.125
214022_s_at	IFITM1	233.0818808	207.6523488	246.9416506	220	830.6093952	0.125
214720_x_at	41527	233.0818808	97.998859	415.3046976	123.4708253	415.3046976	0.125
215311_at	NTRK3	233.0818808	554.365262	207.6523488	246.9416506	123.4708253	0.125
216611_s_at	SLC6A2	233.0818808	554.365262	783.990872	77.78174593	92.49860568	0.125
217621_at	SLC6A2	233.0818808	329.6275569	493.8833013	155.5634919	61.73541266	0.125
219681_s_at	RAB11FIP1	233.0818808	246.9416506	207.6523488	207.6523488	587.3295358	0.125

219895_at	FAM70A	233.0818808	783.990872	82.40688923	164.8137785	329.6275569	0.125
220633_s_at	HP1BP3	233.0818808	195.997718	261.6255653	123.4708253	587.3295358	0.125
201981_at	PAPPA	246.9416506	155.5634919	146.832384	415.3046976	698.4564629	0.125
203126_at	IMPA2	246.9416506	329.6275569	51.9130872	195.997718	622.2539674	0.125
203706_s_at	FZD7	246.9416506	174.6141157	523.2511306	55	349.2282314	0.125
204135_at	FILIP1L	246.9416506	220	659.2551138	261.6255653	97.998859	0.125
204870_s_at	PCSK2	246.9416506	174.6141157	77.78174593	587.3295358	329.6275569	0.125
205542_at	STEAP1	246.9416506	69.29565774	246.9416506	246.9416506	1479.977691	0.21875
205630_at	CRH	246.9416506	1174.659072	246.9416506	261.6255653	195.997718	0.125
205826_at	MYOM2	246.9416506	261.6255653	87.30705786	233.0818808	1244.507935	0.1875
205990_s_at	WNT5A	246.9416506	174.6141157	329.6275569	164.8137785	554.365262	0.125
206973_at	PPFIA2	246.9416506	293.6647679	123.4708253	523.2511306	207.6523488	0.125
208949_s_at	LGALS3	246.9416506	233.0818808	622.2539674	246.9416506	92.49860568	0.125
209120_at	NR2F2	246.9416506	415.3046976	48.9994295	146.832384	440	0.125
209966_x_at	ESRRG	246.9416506	77.78174593	415.3046976	659.2551138	130.8127827	0.125
209975_at	CYP2E1	246.9416506	277.182631	92.49860568	523.2511306	207.6523488	0.125
210881_s_at	IGF2 /// INS-IGF2	246.9416506	261.6255653	246.9416506	932.327523	82.40688923	0.125
211571_s_at	VCAN	246.9416506	329.6275569	466.1637615	48.9994295	184.9972114	0.125
211981_at	COL4A1	246.9416506	103.8261744	587.3295358	92.49860568	587.3295358	0.125
212423_at	ZCCHC24	246.9416506	82.40688923	349.2282314	155.5634919	554.365262	0.125
212489_at	COL5A1	246.9416506	116.5409404	466.1637615	46.24930284	523.2511306	0.125
212670_at	ELN	246.9416506	130.8127827	554.365262	87.30705786	466.1637615	0.125
213921_at	SST	246.9416506	415.3046976	69.29565774	2637.020455	138.5913155	0.75
218035_s_at	RBM47	246.9416506	207.6523488	293.6647679	97.998859	554.365262	0.125
219778_at	ZFPM2	246.9416506	523.2511306	123.4708253	138.5913155	440	0.125
221973_at	LOC100506076 /// LOC100506123	246.9416506	622.2539674	293.6647679	195.997718	207.6523488	0.125
201739_at	SGK1	261.6255653	92.49860568	739.9888454	92.49860568	739.9888454	0.125
202935_s_at	SOX9	261.6255653	466.1637615	369.9944227	184.9972114	87.30705786	0.125
203646_at	FDX1	261.6255653	155.5634919	293.6647679	220	659.2551138	0.125
203697_at	FRZB	261.6255653	830.6093952	311.1269837	220	184.9972114	0.125
204004_at	PAWR	261.6255653	415.3046976	174.6141157	110	830.6093952	0.125
204015_s_at	DUSP4	261.6255653	184.9972114	369.9944227	440	46.24930284	0.125
204779_s_at	HOXB7	261.6255653	261.6255653	277.182631	233.0818808	698.4564629	0.125

205150_s_at	TRIL	261.6255653	277.182631	622.2539674	174.6141157	233.0818808	0.125
205433_at	BCHE	261.6255653	587.3295358	246.9416506	195.997718	261.6255653	0.125
205932_s_at	MSX1	261.6255653	369.9944227	174.6141157	73.41619198	932.327523	0.125
206634_at	SIX3	261.6255653	123.4708253	1174.659072	440	155.5634919	0.1875
207957_s_at	PRKCB	261.6255653	77.78174593	174.6141157	391.995436	830.6093952	0.125
209008_x_at	KRT8	261.6255653	155.5634919	246.9416506	261.6255653	987.7666025	0.125
209529_at	PPAP2C	261.6255653	61.73541266	523.2511306	184.9972114	369.9944227	0.125
209683_at	FAM49A	261.6255653	174.6141157	41.20344461	466.1637615	369.9944227	0.125
210095_s_at	IGFBP3	261.6255653	233.0818808	1396.912926	138.5913155	293.6647679	0.1875
211458_s_at	GABARAPL1 /// GABARAPL3	261.6255653	369.9944227	174.6141157	43.65352893	523.2511306	0.125
211474_s_at	SERPINB6	261.6255653	349.2282314	880	73.41619198	195.997718	0.125
212631_at	STX7	261.6255653	233.0818808	293.6647679	97.998859	698.4564629	0.125
212632_at	STX7	261.6255653	246.9416506	293.6647679	174.6141157	587.3295358	0.125
213620_s_at	ICAM2	261.6255653	391.995436	174.6141157	830.6093952	123.4708253	0.125
213839_at	CLMN	261.6255653	698.4564629	246.9416506	220	277.182631	0.125
215195_at	PRKCA	261.6255653	82.40688923	440	391.995436	164.8137785	0.125
217867_x_at	BACE2	261.6255653	130.8127827	77.78174593	554.365262	622.2539674	0.125
219448_at	TMEM70	261.6255653	277.182631	246.9416506	233.0818808	622.2539674	0.125
220205_at	TPTE	261.6255653	493.8833013	73.41619198	329.6275569	207.6523488	0.125
61734_at	RCN3	261.6255653	246.9416506	277.182631	92.49860568	523.2511306	0.125
201150_s_at	TIMP3	277.182631	220	349.2282314	92.49860568	587.3295358	0.125
203029_s_at	PTPRN2	277.182631	195.997718	415.3046976	415.3046976	69.29565774	0.125
207039_at	CDKN2A	277.182631	246.9416506	261.6255653	261.6255653	739.9888454	0.125
208427_s_at	ELAVL2	277.182631	415.3046976	155.5634919	554.365262	195.997718	0.125
209699_x_at	AKR1C2 /// LOC100653286	277.182631	987.7666025	440	164.8137785	123.4708253	0.125
210353_s_at	SLC6A2	277.182631	466.1637615	830.6093952	164.8137785	46.24930284	0.125
210831_s_at	PTGER3	277.182631	233.0818808	329.6275569	97.998859	1108.730524	0.125
211737_x_at	LOC100287705 /// PTN	277.182631	261.6255653	830.6093952	293.6647679	69.29565774	0.125
212143_s_at	IGFBP3	277.182631	277.182631	880	277.182631	155.5634919	0.125
213791_at	PENK	277.182631	207.6523488	261.6255653	293.6647679	2489.01587	0.5
215715_at	SLC6A2	277.182631	587.3295358	830.6093952	130.8127827	87.30705786	0.125
216973_s_at	HOXB7	277.182631	220	246.9416506	293.6647679	659.2551138	0.125
218959_at	HOXC10	277.182631	293.6647679	233.0818808	207.6523488	659.2551138	0.125

220319_s_at	MYLIP	277.182631	195.997718	369.9944227	103.8261744	554.365262	0.125
221217_s_at	RBFOX1	277.182631	554.365262	207.6523488	349.2282314	174.6141157	0.125
221646_s_at	ZDHHC11	277.182631	830.6093952	246.9416506	311.1269837	207.6523488	0.125
221729_at	COL5A2	277.182631	261.6255653	311.1269837	220	659.2551138	0.125
201667_at	GJA1	293.6647679	184.9972114	440	130.8127827	1396.912926	0.1875
201860_s_at	PLAT	293.6647679	261.6255653	329.6275569	523.2511306	46.24930284	0.125
202283_at	SERPINF1	293.6647679	293.6647679	830.6093952	82.40688923	277.182631	0.125
202609_at	EPS8	293.6647679	261.6255653	311.1269837	41.20344461	493.8833013	0.125
204142_at	ENOSF1	293.6647679	97.998859	391.995436	493.8833013	207.6523488	0.125
205151_s_at	TRIL	293.6647679	391.995436	622.2539674	233.0818808	220	0.125
206745_at	HOXC11	293.6647679	220	220	369.9944227	698.4564629	0.125
206940_s_at	POU4F1	293.6647679	440	195.997718	184.9972114	523.2511306	0.125
207183_at	GPR19	293.6647679	493.8833013	123.4708253	440	184.9972114	0.125
207981_s_at	ESRRG	293.6647679	195.997718	440	659.2551138	184.9972114	0.125
209848_s_at	PMEL	293.6647679	830.6093952	277.182631	184.9972114	293.6647679	0.125
210374_x_at	PTGER3	293.6647679	246.9416506	329.6275569	207.6523488	932.327523	0.125
211456_x_at	MT1P2	293.6647679	184.9972114	659.2551138	195.997718	440	0.125
211653_x_at	AKR1C2 /// LOC100653286	293.6647679	932.327523	391.995436	207.6523488	195.997718	0.125
213438_at	NFASC	293.6647679	440	174.6141157	587.3295358	195.997718	0.125
214043_at	PTPRD	293.6647679	329.6275569	261.6255653	698.4564629	110	0.125
215283_at	LOC339290	293.6647679	659.2551138	293.6647679	293.6647679	261.6255653	0.125
217028_at	CXCR4	293.6647679	739.9888454	523.2511306	103.8261744	155.5634919	0.125
218718_at	PDGFC	293.6647679	293.6647679	293.6647679	207.6523488	659.2551138	0.125
218865_at	41334	293.6647679	130.8127827	587.3295358	622.2539674	146.832384	0.125
219256_s_at	SH3TC1	293.6647679	261.6255653	329.6275569	698.4564629	116.5409404	0.125
219506_at	C1orf54	293.6647679	391.995436	103.8261744	207.6523488	554.365262	0.125
219869_s_at	SLC39A8	293.6647679	261.6255653	329.6275569	174.6141157	587.3295358	0.125
201983_s_at	EGFR	311.1269837	329.6275569	523.2511306	277.182631	43.65352893	0.125
202410_x_at	IGF2 /// INS-IGF2	311.1269837	329.6275569	293.6647679	1396.912926	51.9130872	0.1875
202838_at	FUCA1	311.1269837	174.6141157	466.1637615	207.6523488	659.2551138	0.125
203038_at	PTPRK	311.1269837	233.0818808	155.5634919	415.3046976	932.327523	0.125
203325_s_at	COL5A1	311.1269837	164.8137785	523.2511306	195.997718	493.8833013	0.125
203722_at	ALDH4A1	311.1269837	261.6255653	369.9944227	233.0818808	698.4564629	0.125
203849_s_at	KIF1A	311.1269837	440	220	523.2511306	87.30705786	0.125

204014_at	DUSP4	311.1269837	277.182631	329.6275569	587.3295358	123.4708253	0.125
204137_at	GPR137B	311.1269837	349.2282314	233.0818808	277.182631	698.4564629	0.125
204388_s_at	MAOA	311.1269837	466.1637615	415.3046976	82.40688923	220	0.125
204400_at	EFS	311.1269837	138.5913155	554.365262	233.0818808	415.3046976	0.125
204451_at	FZD1	311.1269837	246.9416506	369.9944227	123.4708253	622.2539674	0.125
205110_s_at	FGF13	311.1269837	987.7666025	155.5634919	277.182631	349.2282314	0.125
205862_at	GREB1	311.1269837	523.2511306	391.995436	233.0818808	110	0.125
206290_s_at	RGS7	311.1269837	349.2282314	261.6255653	587.3295358	87.30705786	0.125
206314_at	ZNF167	311.1269837	41.20344461	493.8833013	493.8833013	184.9972114	0.125
207172_s_at	CDH11	311.1269837	73.41619198	349.2282314	277.182631	739.9888454	0.125
209293_x_at	ID4	311.1269837	293.6647679	311.1269837	261.6255653	783.990872	0.125
209994_s_at	ABCB1 /// ABCB4	311.1269837	220	415.3046976	523.2511306	41.20344461	0.125
210393_at	LGR5	311.1269837	311.1269837	739.9888454	293.6647679	92.49860568	0.125
210467_x_at	MAGEA12	311.1269837	329.6275569	739.9888454	293.6647679	184.9972114	0.125
210832_x_at	PTGER3	311.1269837	233.0818808	415.3046976	220	987.7666025	0.125
213201_s_at	TNNT1	311.1269837	698.4564629	130.8127827	61.73541266	830.6093952	0.125
213725_x_at	XYLT1	311.1269837	277.182631	329.6275569	659.2551138	261.6255653	0.125
213808_at	ADAM23	311.1269837	622.2539674	261.6255653	369.9944227	174.6141157	0.125
213960_at	NTRK3	311.1269837	622.2539674	329.6275569	277.182631	220	0.125
216092_s_at	SLC7A8	311.1269837	329.6275569	293.6647679	174.6141157	659.2551138	0.125
217853_at	TNS3	311.1269837	77.78174593	554.365262	880	164.8137785	0.125
218006_s_at	ZNF22	311.1269837	116.5409404	523.2511306	184.9972114	523.2511306	0.125
218499_at	MST4	311.1269837	195.997718	523.2511306	130.8127827	523.2511306	0.125
218521_s_at	UBE2W	311.1269837	329.6275569	277.182631	311.1269837	659.2551138	0.125
220887_at	C14orf162	311.1269837	587.3295358	155.5634919	391.995436	233.0818808	0.125
221944_at	FLJ42627	311.1269837	523.2511306	466.1637615	207.6523488	174.6141157	0.125
41577_at	PPP1R16B	311.1269837	329.6275569	277.182631	554.365262	92.49860568	0.125
1438_at	EPHB3	329.6275569	311.1269837	329.6275569	293.6647679	830.6093952	0.125
201302_at	ANXA4	329.6275569	110	554.365262	246.9416506	415.3046976	0.125
201481_s_at	PYGB	329.6275569	440	622.2539674	233.0818808	123.4708253	0.125
202052_s_at	RAI14	329.6275569	103.8261744	466.1637615	698.4564629	220	0.125
202157_s_at	CELF2	329.6275569	739.9888454	311.1269837	349.2282314	246.9416506	0.125
203570_at	LOXL1	329.6275569	466.1637615	69.29565774	415.3046976	246.9416506	0.125
203789_s_at	SEMA3C	329.6275569	146.832384	880	41.20344461	739.9888454	0.125
203814_s_at	NQO2	329.6275569	293.6647679	369.9944227	69.29565774	880	0.125
204068_at	STK3	329.6275569	34.64782887	349.2282314	311.1269837	466.1637615	0.125

204457_s_at	GAS1	329.6275569	329.6275569	311.1269837	184.9972114	1108.730524	0.125
205637_s_at	SH3GL3	329.6275569	391.995436	277.182631	155.5634919	987.7666025	0.125
205923_at	RELN	329.6275569	830.6093952	440	207.6523488	220	0.125
206710_s_at	EPB41L3	329.6275569	493.8833013	48.9994295	415.3046976	277.182631	0.125
208792_s_at	CLU	329.6275569	880	466.1637615	220	195.997718	0.125
208917_x_at	NADK	329.6275569	184.9972114	391.995436	261.6255653	587.3295358	0.125
209122_at	LOC100509484 /// PLIN2	329.6275569	146.832384	698.4564629	261.6255653	415.3046976	0.125
209356_x_at	EFEMP2	329.6275569	77.78174593	659.2551138	349.2282314	293.6647679	0.125
209921_at	SLC7A11	329.6275569	391.995436	277.182631	164.8137785	698.4564629	0.125
209993_at	ABCB1	329.6275569	233.0818808	466.1637615	622.2539674	77.78174593	0.125
211504_x_at	ROCK2	329.6275569	329.6275569	329.6275569	783.990872	277.182631	0.125
211896_s_at	DCN	329.6275569	184.9972114	587.3295358	164.8137785	659.2551138	0.125
212203_x_at	IFITM3	329.6275569	146.832384	739.9888454	92.49860568	1244.507935	0.1875
212398_at	RDX	329.6275569	277.182631	369.9944227	103.8261744	783.990872	0.125
212559_at	PRKAR1B	329.6275569	261.6255653	659.2551138	349.2282314	311.1269837	0.125
212805_at	PRUNE2	329.6275569	622.2539674	155.5634919	466.1637615	220	0.125
212859_x_at	LOC100505584 /// MT1E	329.6275569	220	622.2539674	293.6647679	349.2282314	0.125
213721_at	SOX2	329.6275569	103.8261744	174.6141157	587.3295358	987.7666025	0.125
216511_s_at	TCF7L2	329.6275569	391.995436	659.2551138	233.0818808	261.6255653	0.125
218656_s_at	LHFP	329.6275569	391.995436	261.6255653	116.5409404	622.2539674	0.125
219072_at	BCL7C	329.6275569	233.0818808	554.365262	103.8261744	466.1637615	0.125
219132_at	PELI2	329.6275569	207.6523488	87.30705786	493.8833013	493.8833013	0.125
220975_s_at	C1QTNF1	329.6275569	293.6647679	587.3295358	369.9944227	103.8261744	0.125
221310_at	FGF14	329.6275569	246.9416506	587.3295358	415.3046976	195.997718	0.125
221881_s_at	CLIC4	329.6275569	329.6275569	311.1269837	311.1269837	783.990872	0.125
222196_at	LOC389906	329.6275569	622.2539674	391.995436	69.29565774	277.182631	0.125
45288_at	ABHD6	329.6275569	293.6647679	246.9416506	349.2282314	783.990872	0.125
200974_at	ACTA2	349.2282314	311.1269837	932.327523	391.995436	293.6647679	0.125
201289_at	CYR61	349.2282314	311.1269837	554.365262	87.30705786	369.9944227	0.125
201579_at	FAT1	349.2282314	622.2539674	587.3295358	123.4708253	195.997718	0.125
202625_at	LYN	349.2282314	233.0818808	587.3295358	523.2511306	207.6523488	0.125
203131_at	PDGFRA	349.2282314	783.990872	554.365262	207.6523488	155.5634919	0.125
203186_s_at	S100A4	349.2282314	233.0818808	523.2511306	92.49860568	622.2539674	0.125
203619_s_at	FAIM2	349.2282314	523.2511306	233.0818808	698.4564629	97.998859	0.125

203799_at	CD302 /// LY75-CD302	349.2282314	82.40688923	293.6647679	783.990872	415.3046976	0.125
203836_s_at	MAP3K5	349.2282314	329.6275569	277.182631	391.995436	698.4564629	0.125
204042_at	WASF3	349.2282314	493.8833013	739.9888454	207.6523488	246.9416506	0.125
204083_s_at	TPM2	349.2282314	46.24930284	391.995436	293.6647679	622.2539674	0.125
204163_at	EMILIN1	349.2282314	277.182631	739.9888454	82.40688923	440	0.125
204359_at	FLRT2 /// LOC100506718	349.2282314	523.2511306	130.8127827	220	932.327523	0.125
205051_s_at	KIT	349.2282314	195.997718	523.2511306	220	587.3295358	0.125
205173_x_at	CD58	349.2282314	329.6275569	349.2282314	48.9994295	554.365262	0.125
206953_s_at	LPHN2	349.2282314	783.990872	880	138.5913155	155.5634919	0.125
208812_x_at	HLA-C	349.2282314	174.6141157	554.365262	207.6523488	880	0.125
209121_x_at	NR2F2	349.2282314	523.2511306	73.41619198	246.9416506	523.2511306	0.125
209140_x_at	HLA-B	349.2282314	116.5409404	523.2511306	233.0818808	880	0.125
209156_s_at	COL6A2	349.2282314	329.6275569	739.9888454	369.9944227	349.2282314	0.125
209687_at	CXCL12	349.2282314	554.365262	155.5634919	622.2539674	207.6523488	0.125
211006_s_at	KCNB1	349.2282314	554.365262	293.6647679	391.995436	130.8127827	0.125
212448_at	NEDD4L	349.2282314	830.6093952	246.9416506	415.3046976	277.182631	0.125
212459_x_at	SUCLG2	349.2282314	51.9130872	493.8833013	391.995436	311.1269837	0.125
212750_at	PPP1R16B	349.2282314	391.995436	329.6275569	739.9888454	55	0.125
213435_at	SATB2	349.2282314	1046.502261	110	329.6275569	349.2282314	0.125
214671_s_at	ABR	349.2282314	65.40639133	523.2511306	369.9944227	311.1269837	0.125
214945_at	FAM153A /// FAM153B /// FAM153C /// LOC100507387 /// LOC100507427	349.2282314	659.2551138	155.5634919	698.4564629	174.6141157	0.125
215322_at	LONRF1	349.2282314	369.9944227	311.1269837	311.1269837	1174.659072	0.125
215446_s_at	LOX	349.2282314	220	246.9416506	466.1637615	659.2551138	0.125
217456_x_at	HLA-E	349.2282314	233.0818808	369.9944227	311.1269837	698.4564629	0.125
217712_at	---	349.2282314	554.365262	349.2282314	36.70809599	349.2282314	0.125
218025_s_at	ECI2	349.2282314	195.997718	622.2539674	195.997718	830.6093952	0.125
218559_s_at	MAFB	349.2282314	311.1269837	369.9944227	73.41619198	1975.533205	0.25
218625_at	NRN1	349.2282314	233.0818808	493.8833013	246.9416506	1108.730524	0.125
218858_at	DEPTOR	349.2282314	440	261.6255653	659.2551138	261.6255653	0.125

219377_at	FAM59A	349.2282314	369.9944227	698.4564629	97.998859	311.1269837	0.125
219427_at	FAT4	349.2282314	369.9944227	311.1269837	184.9972114	880	0.125
219976_at	HOOK1	349.2282314	293.6647679	277.182631	391.995436	830.6093952	0.125
200872_at	S100A10	369.9944227	277.182631	493.8833013	130.8127827	493.8833013	0.125
201631_s_at	IER3	369.9944227	277.182631	277.182631	1174.659072	523.2511306	0.125
201809_s_at	ENG	369.9944227	415.3046976	523.2511306	69.29565774	349.2282314	0.125
201976_s_at	MYO10	369.9944227	440	329.6275569	246.9416506	1244.507935	0.125
202766_s_at	FBN1	369.9944227	311.1269837	1046.502261	440	195.997718	0.125
202894_at	EPHB4	369.9944227	174.6141157	554.365262	246.9416506	622.2539674	0.125
202972_s_at	FAM13A	369.9944227	349.2282314	329.6275569	783.990872	369.9944227	0.125
203596_s_at	IFIT5	369.9944227	554.365262	277.182631	466.1637615	55	0.125
203729_at	EMP3	369.9944227	293.6647679	440	220	1396.912926	0.1875
203889_at	SCG5	369.9944227	184.9972114	739.9888454	880	51.9130872	0.125
204304_s_at	PROM1	369.9944227	261.6255653	261.6255653	987.7666025	493.8833013	0.125
204326_x_at	MT1X	369.9944227	195.997718	587.3295358	220	622.2539674	0.125
204417_at	GALC	369.9944227	73.41619198	493.8833013	523.2511306	261.6255653	0.125
204464_s_at	EDNRA	369.9944227	261.6255653	493.8833013	195.997718	739.9888454	0.125
204557_s_at	DZIP1	369.9944227	440	349.2282314	369.9944227	48.9994295	0.125
204600_at	EPHB3	369.9944227	415.3046976	349.2282314	293.6647679	783.990872	0.125
204867_at	GCHFR	369.9944227	880	246.9416506	261.6255653	493.8833013	0.125
205165_at	CELSR3	369.9944227	440	440	311.1269837	61.73541266	0.125
205358_at	GRIA2	369.9944227	987.7666025	207.6523488	659.2551138	55	0.125
205375_at	MDFI	369.9944227	184.9972114	466.1637615	293.6647679	830.6093952	0.125
205405_at	SEMA5A	369.9944227	369.9944227	523.2511306	369.9944227	82.40688923	0.125
205722_s_at	GFRA2	369.9944227	783.990872	391.995436	130.8127827	369.9944227	0.125
205850_s_at	GABRB3	369.9944227	493.8833013	277.182631	493.8833013	123.4708253	0.125
206189_at	UNC5C	369.9944227	415.3046976	349.2282314	523.2511306	92.49860568	0.125
207327_at	EYA4	369.9944227	739.9888454	233.0818808	493.8833013	277.182631	0.125
208116_s_at	MAN1A1	369.9944227	415.3046976	349.2282314	466.1637615	73.41619198	0.125
208891_at	DUSP6	369.9944227	146.832384	622.2539674	554.365262	233.0818808	0.125
209167_at	GPM6B	369.9944227	466.1637615	293.6647679	622.2539674	82.40688923	0.125
209170_s_at	GPM6B	369.9944227	493.8833013	277.182631	587.3295358	155.5634919	0.125
209644_x_at	CDKN2A	369.9944227	391.995436	277.182631	349.2282314	1108.730524	0.125
209985_s_at	ASCL1	369.9944227	587.3295358	466.1637615	277.182631	61.73541266	0.125
210816_s_at	CYB561	369.9944227	246.9416506	523.2511306	587.3295358	184.9972114	0.125
210880_s_at	EFS	369.9944227	110	523.2511306	311.1269837	415.3046976	0.125

210964_s_at	GYG2	369.9944227	311.1269837	293.6647679	415.3046976	783.990872	0.125
211767_at	GINS4	369.9944227	311.1269837	233.0818808	440	739.9888454	0.125
211966_at	COL4A2	369.9944227	207.6523488	739.9888454	138.5913155	622.2539674	0.125
212377_s_at	NOTCH2	369.9944227	184.9972114	659.2551138	195.997718	659.2551138	0.125
212817_at	DNAJB5	369.9944227	415.3046976	349.2282314	698.4564629	311.1269837	0.125
214612_x_at	MAGEA6	369.9944227	622.2539674	1108.730524	130.8127827	207.6523488	0.125
215772_x_at	SUCLG2	369.9944227	97.998859	523.2511306	369.9944227	349.2282314	0.125
217677_at	PLEKHA2	369.9944227	293.6647679	233.0818808	493.8833013	622.2539674	0.125
217983_s_at	RNASET2	369.9944227	233.0818808	659.2551138	349.2282314	391.995436	0.125
218847_at	IGF2BP2	369.9944227	391.995436	261.6255653	329.6275569	1046.502261	0.125
219005_at	TMEM59L	369.9944227	466.1637615	349.2282314	391.995436	65.40639133	0.125
219142_at	RASL11B	369.9944227	391.995436	698.4564629	329.6275569	311.1269837	0.125
219196_at	SCG3	369.9944227	523.2511306	261.6255653	554.365262	34.64782887	0.125
219873_at	COLEC11	369.9944227	391.995436	1108.730524	369.9944227	138.5913155	0.125
221586_s_at	E2F5	369.9944227	369.9944227	277.182631	369.9944227	698.4564629	0.125
201185_at	HTRA1	391.995436	293.6647679	830.6093952	493.8833013	155.5634919	0.125
202600_s_at	NRIP1	391.995436	659.2551138	138.5913155	783.990872	220	0.125
202720_at	TES	391.995436	277.182631	523.2511306	246.9416506	622.2539674	0.125
202936_s_at	SOX9	391.995436	622.2539674	493.8833013	195.997718	293.6647679	0.125
203238_s_at	NOTCH3	391.995436	369.9944227	659.2551138	246.9416506	391.995436	0.125
203959_s_at	ZBTB40	391.995436	293.6647679	622.2539674	523.2511306	220	0.125
203998_s_at	SYT1	391.995436	932.327523	369.9944227	415.3046976	277.182631	0.125
204352_at	TRAF5	391.995436	246.9416506	659.2551138	415.3046976	329.6275569	0.125
205331_s_at	REEP2	391.995436	415.3046976	369.9944227	554.365262	110	0.125
205543_at	HSPA4L	391.995436	391.995436	277.182631	739.9888454	369.9944227	0.125
205737_at	KCNQ2	391.995436	622.2539674	440	349.2282314	146.832384	0.125
206481_s_at	LDB2	391.995436	440	246.9416506	349.2282314	783.990872	0.125
206516_at	AMH	391.995436	493.8833013	554.365262	311.1269837	155.5634919	0.125
208107_s_at	LOC81691	391.995436	523.2511306	311.1269837	493.8833013	146.832384	0.125
209466_x_at	LOC100287705 /// PTN	391.995436	369.9944227	880	415.3046976	277.182631	0.125
209605_at	TST	391.995436	349.2282314	493.8833013	440	87.30705786	0.125
209685_s_at	PRKCB	391.995436	261.6255653	220	587.3295358	1318.510228	0.1875
210963_s_at	GYG2	391.995436	329.6275569	246.9416506	466.1637615	783.990872	0.125
211813_x_at	DCN	391.995436	233.0818808	659.2551138	146.832384	830.6093952	0.125
212095_s_at	MTUS1	391.995436	659.2551138	311.1269837	493.8833013	261.6255653	0.125

212415_at	41523	391.995436	587.3295358	261.6255653	587.3295358	174.6141157	0.125
212912_at	RPS6KA2	391.995436	493.8833013	783.990872	329.6275569	110	0.125
213880_at	LGR5	391.995436	440	987.7666025	369.9944227	61.73541266	0.125
213933_at	PTGER3	391.995436	369.9944227	415.3046976	164.8137785	1174.659072	0.125
214053_at	ERBB4	391.995436	311.1269837	329.6275569	880	466.1637615	0.125
214218_s_at	XIST	391.995436	155.5634919	932.327523	184.9972114	783.990872	0.125
214920_at	THSD7A	391.995436	293.6647679	523.2511306	739.9888454	246.9416506	0.125
215145_s_at	CNTNAP2	391.995436	587.3295358	349.2282314	58.27047019	440	0.125
216035_x_at	TCF7L2	391.995436	391.995436	554.365262	116.5409404	369.9944227	0.125
216594_x_at	AKR1C1	391.995436	1046.502261	466.1637615	311.1269837	329.6275569	0.125
217818_s_at	ARPC4	391.995436	311.1269837	739.9888454	493.8833013	233.0818808	0.125
218826_at	SLC35F2	391.995436	311.1269837	523.2511306	207.6523488	698.4564629	0.125
218950_at	ARAP3	391.995436	103.8261744	739.9888454	698.4564629	207.6523488	0.125
219501_at	ENOX1	391.995436	554.365262	293.6647679	493.8833013	164.8137785	0.125
219668_at	GDAP1L1	391.995436	622.2539674	293.6647679	523.2511306	110	0.125
220615_s_at	FAR2	391.995436	138.5913155	440	698.4564629	369.9944227	0.125
220707_s_at	FOXRED2	391.995436	554.365262	369.9944227	391.995436	130.8127827	0.125
200600_at	MSN	415.3046976	523.2511306	349.2282314	261.6255653	1108.730524	0.125
201482_at	QSOX1	415.3046976	415.3046976	739.9888454	369.9944227	391.995436	0.125
201787_at	FBLN1	415.3046976	369.9944227	880	440	220	0.125
201951_at	ALCAM	415.3046976	349.2282314	523.2511306	311.1269837	739.9888454	0.125
202085_at	TJP2	415.3046976	246.9416506	622.2539674	554.365262	311.1269837	0.125
202431_s_at	MYC	415.3046976	184.9972114	880	123.4708253	1479.977691	0.1875
202921_s_at	ANK2	415.3046976	523.2511306	329.6275569	830.6093952	261.6255653	0.125
202995_s_at	FBLN1	415.3046976	329.6275569	739.9888454	349.2282314	466.1637615	0.125
203394_s_at	HES1	415.3046976	277.182631	493.8833013	329.6275569	783.990872	0.125
203424_s_at	IGFBP5	415.3046976	880	554.365262	233.0818808	277.182631	0.125
204139_x_at	MZF1	415.3046976	523.2511306	622.2539674	329.6275569	130.8127827	0.125
204151_x_at	AKR1C1	415.3046976	1396.912926	493.8833013	233.0818808	329.6275569	0.1875
204364_s_at	REEP1	415.3046976	587.3295358	293.6647679	659.2551138	220	0.125
204431_at	TLE2	415.3046976	466.1637615	207.6523488	622.2539674	369.9944227	0.125
204723_at	SCN3B	415.3046976	587.3295358	391.995436	415.3046976	123.4708253	0.125
204851_s_at	DCX	415.3046976	130.8127827	391.995436	1174.659072	440	0.125
205197_s_at	ATP7A	415.3046976	739.9888454	415.3046976	329.6275569	440	0.125
205303_at	KCNJ8	415.3046976	440	369.9944227	554.365262	103.8261744	0.125
205373_at	CTNNA2	415.3046976	587.3295358	329.6275569	523.2511306	174.6141157	0.125

205593_s_at	PDE9A	415.3046976	77.78174593	466.1637615	391.995436	415.3046976	0.125
206013_s_at	ACTL6B	415.3046976	415.3046976	415.3046976	554.365262	55	0.125
206281_at	ADCYAP1	415.3046976	659.2551138	184.9972114	783.990872	261.6255653	0.125
206718_at	LMO1	415.3046976	391.995436	739.9888454	440	138.5913155	0.125
209357_at	CITED2	415.3046976	523.2511306	155.5634919	329.6275569	932.327523	0.125
209505_at	NR2F1	415.3046976	233.0818808	391.995436	1108.730524	391.995436	0.125
209784_s_at	JAG2	415.3046976	554.365262	311.1269837	293.6647679	698.4564629	0.125
210251_s_at	RUFY3	415.3046976	587.3295358	329.6275569	523.2511306	207.6523488	0.125
210302_s_at	MAB21L2	415.3046976	1975.533205	311.1269837	523.2511306	164.8137785	0.25
211028_s_at	KHK	415.3046976	261.6255653	415.3046976	391.995436	739.9888454	0.125
211161_s_at	COL3A1	415.3046976	369.9944227	830.6093952	116.5409404	440	0.125
211958_at	IGFBP5	415.3046976	698.4564629	523.2511306	220	329.6275569	0.125
211964_at	COL4A2	415.3046976	220	932.327523	103.8261744	783.990872	0.125
212093_s_at	MTUS1	415.3046976	783.990872	369.9944227	466.1637615	220	0.125
212488_at	COL5A1	415.3046976	261.6255653	698.4564629	233.0818808	659.2551138	0.125
213169_at	SEMA5A	415.3046976	493.8833013	659.2551138	349.2282314	164.8137785	0.125
213245_at	ADCY1	415.3046976	622.2539674	739.9888454	261.6255653	164.8137785	0.125
214839_at	LINC00599 /// MIR124-1	415.3046976	622.2539674	391.995436	440	164.8137785	0.125
215045_at	CELF3	415.3046976	523.2511306	369.9944227	440	87.30705786	0.125
217790_s_at	SSR3	415.3046976	349.2282314	233.0818808	523.2511306	698.4564629	0.125
218066_at	SLC12A7	415.3046976	349.2282314	391.995436	391.995436	932.327523	0.125
218234_at	ING4	415.3046976	440	622.2539674	415.3046976	174.6141157	0.125
218435_at	DNAJC15	415.3046976	261.6255653	622.2539674	987.7666025	174.6141157	0.125
218694_at	ARMCX1	415.3046976	739.9888454	415.3046976	415.3046976	195.997718	0.125
219271_at	GALNT14	415.3046976	246.9416506	311.1269837	523.2511306	739.9888454	0.125
219528_s_at	BCL11B	415.3046976	440	155.5634919	369.9944227	1108.730524	0.125
219685_at	TMEM35	415.3046976	587.3295358	493.8833013	349.2282314	164.8137785	0.125
220233_at	FBXO17 /// SARS2	415.3046976	65.40639133	440	349.2282314	466.1637615	0.125
221731_x_at	VCAN	415.3046976	659.2551138	783.990872	195.997718	261.6255653	0.125
221972_s_at	SDF4	415.3046976	77.78174593	523.2511306	369.9944227	440	0.125
222133_s_at	PHF20L1	415.3046976	391.995436	349.2282314	415.3046976	987.7666025	0.125
32137_at	JAG2	415.3046976	554.365262	311.1269837	311.1269837	659.2551138	0.125
201286_at	SDC1	440	349.2282314	554.365262	554.365262	73.41619198	0.125
201373_at	PLEC	440	146.832384	493.8833013	391.995436	523.2511306	0.125

201625_s_at	INSIG1	440	523.2511306	329.6275569	391.995436	739.9888454	0.125
202436_s_at	CYP1B1	440	440	415.3046976	277.182631	830.6093952	0.125
202794_at	INPP1	440	329.6275569	554.365262	783.990872	349.2282314	0.125
203643_at	ERF	440	311.1269837	415.3046976	440	783.990872	0.125
203938_s_at	TAF1C	440	466.1637615	523.2511306	415.3046976	92.49860568	0.125
204011_at	SPRY2	440	466.1637615	554.365262	391.995436	82.40688923	0.125
204036_at	LPAR1	440	523.2511306	369.9944227	233.0818808	659.2551138	0.125
204081_at	NRGN	440	440	415.3046976	783.990872	391.995436	0.125
204294_at	AMT	440	466.1637615	739.9888454	415.3046976	329.6275569	0.125
204321_at	NEO1	440	466.1637615	783.990872	311.1269837	391.995436	0.125
204722_at	SCN3B	440	659.2551138	415.3046976	440	103.8261744	0.125
204906_at	RPS6KA2	440	587.3295358	880	329.6275569	261.6255653	0.125
205399_at	DCLK1	440	554.365262	329.6275569	739.9888454	174.6141157	0.125
205515_at	PRSS12	440	587.3295358	493.8833013	369.9944227	92.49860568	0.125
205604_at	HOXD9	440	440	415.3046976	783.990872	415.3046976	0.125
205659_at	HDAC9	440	587.3295358	587.3295358	155.5634919	329.6275569	0.125
206001_at	NPY	440	103.8261744	1046.502261	587.3295358	311.1269837	0.125
206046_at	ADAM23	440	783.990872	415.3046976	466.1637615	369.9944227	0.125
206108_s_at	SRSF6	440	587.3295358	659.2551138	293.6647679	329.6275569	0.125
206163_at	MAB21L1	440	1244.507935	1108.730524	164.8137785	69.29565774	0.1875
206200_s_at	ANXA11	440	73.41619198	554.365262	329.6275569	622.2539674	0.125
206376_at	SLC6A15	440	659.2551138	349.2282314	220	493.8833013	0.125
206450_at	DBH	440	659.2551138	1174.659072	293.6647679	116.5409404	0.125
207463_x_at	PRSS3	440	293.6647679	622.2539674	739.9888454	195.997718	0.125
207980_s_at	CITED2	440	587.3295358	220	329.6275569	1046.502261	0.125
208033_s_at	ZFHX3	440	659.2551138	554.365262	329.6275569	77.78174593	0.125
208581_x_at	MT1X	440	184.9972114	587.3295358	293.6647679	698.4564629	0.125
208816_x_at	ANXA2P2	440	233.0818808	830.6093952	164.8137785	987.7666025	0.125
208892_s_at	DUSP6	440	246.9416506	659.2551138	493.8833013	349.2282314	0.125
209189_at	FOS	440	277.182631	783.990872	440	391.995436	0.125
209325_s_at	RGS16	440	466.1637615	391.995436	1108.730524	329.6275569	0.125
209506_s_at	NR2F1	440	349.2282314	466.1637615	932.327523	415.3046976	0.125
210139_s_at	PMP22	440	880	987.7666025	233.0818808	220	0.125
210150_s_at	LAMA5	440	415.3046976	739.9888454	440	277.182631	0.125
210432_s_at	SCN3A	440	659.2551138	349.2282314	523.2511306	116.5409404	0.125
211530_x_at	HLA-G	440	369.9944227	523.2511306	349.2282314	987.7666025	0.125

212226_s_at	PPAP2B	440	277.182631	622.2539674	739.9888454	233.0818808	0.125
212638_s_at	WWP1	440	349.2282314	415.3046976	466.1637615	783.990872	0.125
212646_at	RFTN1	440	415.3046976	493.8833013	1244.507935	164.8137785	0.125
213411_at	ADAM22	440	440	698.4564629	466.1637615	195.997718	0.125
213676_at	TMEM151B	440	739.9888454	415.3046976	466.1637615	207.6523488	0.125
214247_s_at	DKK3	440	493.8833013	622.2539674	369.9944227	195.997718	0.125
214347_s_at	DDC	440	440	415.3046976	783.990872	92.49860568	0.125
214608_s_at	EYA1	440	622.2539674	391.995436	493.8833013	77.78174593	0.125
218831_s_at	FCGRT	440	369.9944227	493.8833013	130.8127827	1864.655046	0.25
219300_s_at	CNTNAP2	440	554.365262	369.9944227	155.5634919	493.8833013	0.125
219733_s_at	SLC27A5	440	123.4708253	391.995436	466.1637615	587.3295358	0.125
220334_at	RGS17	440	466.1637615	415.3046976	523.2511306	87.30705786	0.125
221047_s_at	MARK1	440	587.3295358	391.995436	493.8833013	184.9972114	0.125
221552_at	ABHD6	440	440	349.2282314	415.3046976	932.327523	0.125
221585_at	CACNG4	440	554.365262	622.2539674	329.6275569	51.9130872	0.125
201315_x_at	IFITM2	466.1637615	329.6275569	622.2539674	220	739.9888454	0.125
201596_x_at	KRT18	466.1637615	261.6255653	659.2551138	311.1269837	1661.21879	0.21875
201615_x_at	CALD1	466.1637615	466.1637615	659.2551138	466.1637615	92.49860568	0.125
201889_at	FAM3C	466.1637615	783.990872	493.8833013	415.3046976	415.3046976	0.125
202443_x_at	NOTCH2	466.1637615	246.9416506	783.990872	207.6523488	830.6093952	0.125
202538_s_at	CHMP2B	466.1637615	523.2511306	415.3046976	246.9416506	659.2551138	0.125
203588_s_at	TFDP2	466.1637615	440	466.1637615	440	880	0.125
203647_s_at	FDX1	466.1637615	293.6647679	554.365262	369.9944227	987.7666025	0.125
203695_s_at	DFNA5	466.1637615	493.8833013	987.7666025	440	293.6647679	0.125
204175_at	ZNF593	466.1637615	369.9944227	523.2511306	349.2282314	739.9888454	0.125
204365_s_at	REEP1	466.1637615	622.2539674	349.2282314	698.4564629	293.6647679	0.125
204440_at	CD83	466.1637615	391.995436	329.6275569	523.2511306	739.9888454	0.125
204529_s_at	TOX	466.1637615	391.995436	554.365262	698.4564629	207.6523488	0.125
204604_at	CDK14	466.1637615	246.9416506	783.990872	233.0818808	932.327523	0.125
205305_at	FGL1	466.1637615	587.3295358	369.9944227	659.2551138	277.182631	0.125
205447_s_at	MAP3K12	466.1637615	493.8833013	466.1637615	466.1637615	97.998859	0.125
206565_x_at	SMA4	466.1637615	293.6647679	415.3046976	523.2511306	698.4564629	0.125
208729_x_at	HLA-B	466.1637615	415.3046976	493.8833013	349.2282314	783.990872	0.125
209012_at	TRIO	466.1637615	587.3295358	830.6093952	369.9944227	293.6647679	0.125
209168_at	GPM6B	466.1637615	659.2551138	349.2282314	587.3295358	220	0.125
209781_s_at	KHDRBS3	466.1637615	830.6093952	493.8833013	415.3046976	116.5409404	0.125

210069_at	CHKB-CPT1B /// CPT1B	466.1637615	659.2551138	493.8833013	440	77.78174593	0.125
211203_s_at	CNTN1	466.1637615	698.4564629	830.6093952	293.6647679	293.6647679	0.125
211685_s_at	NCALD	466.1637615	391.995436	554.365262	783.990872	261.6255653	0.125
211980_at	COL4A1	466.1637615	233.0818808	880	195.997718	932.327523	0.125
212003_at	C1orf144	466.1637615	116.5409404	493.8833013	415.3046976	554.365262	0.125
212396_s_at	EMC1	466.1637615	329.6275569	622.2539674	349.2282314	698.4564629	0.125
212526_at	SPG20	466.1637615	587.3295358	233.0818808	369.9944227	587.3295358	0.125
212665_at	TIPARP	466.1637615	415.3046976	415.3046976	523.2511306	932.327523	0.125
212706_at	RASA4 /// RASA4B	466.1637615	523.2511306	587.3295358	415.3046976	195.997718	0.125
213324_at	SRC	466.1637615	369.9944227	587.3295358	554.365262	155.5634919	0.125
213670_x_at	NSUN5P1	466.1637615	493.8833013	830.6093952	349.2282314	440	0.125
213894_at	THSD7A	466.1637615	349.2282314	587.3295358	932.327523	110	0.125
217492_s_at	PTEN /// PTENP1	466.1637615	493.8833013	739.9888454	440	329.6275569	0.125
217904_s_at	BACE1	466.1637615	415.3046976	659.2551138	523.2511306	87.30705786	0.125
218029_at	FAM65A	466.1637615	466.1637615	523.2511306	493.8833013	97.998859	0.125
218321_x_at	STYXL1	466.1637615	415.3046976	698.4564629	554.365262	277.182631	0.125
218337_at	FAM160B2	466.1637615	415.3046976	739.9888454	523.2511306	329.6275569	0.125
220295_x_at	DEPDC1	466.1637615	174.6141157	391.995436	523.2511306	739.9888454	0.125
220795_s_at	BEGAIN	466.1637615	349.2282314	783.990872	587.3295358	293.6647679	0.125
221824_s_at	41341	466.1637615	659.2551138	493.8833013	415.3046976	184.9972114	0.125
221909_at	RNFT2	466.1637615	493.8833013	440	587.3295358	138.5913155	0.125
221969_at	PAX5	466.1637615	493.8833013	466.1637615	622.2539674	92.49860568	0.125
200787_s_at	PEA15	493.8833013	587.3295358	830.6093952	415.3046976	329.6275569	0.125
200808_s_at	ZYX	493.8833013	698.4564629	659.2551138	369.9944227	261.6255653	0.125
200878_at	EPAS1 /// LOC100652809	493.8833013	369.9944227	659.2551138	1046.502261	311.1269837	0.125
201617_x_at	CALD1	493.8833013	587.3295358	554.365262	415.3046976	123.4708253	0.125
202284_s_at	CDKN1A	493.8833013	523.2511306	466.1637615	659.2551138	146.832384	0.125
202388_at	RGS2	493.8833013	880	466.1637615	523.2511306	369.9944227	0.125
202599_s_at	NRIP1	493.8833013	880	103.8261744	880	277.182631	0.125
202675_at	SDHB	493.8833013	369.9944227	523.2511306	440	783.990872	0.125
203303_at	DYNLT3	493.8833013	195.997718	523.2511306	440	783.990872	0.125
203447_at	PSMD5	493.8833013	97.998859	523.2511306	523.2511306	440	0.125
203595_s_at	IFIT5	493.8833013	659.2551138	391.995436	587.3295358	110	0.125

203607_at	INPP5F	493.8833013	440	830.6093952	587.3295358	293.6647679	0.125
203812_at	SLIT3	493.8833013	554.365262	698.4564629	440	246.9416506	0.125
204053_x_at	PTEN	493.8833013	493.8833013	830.6093952	440	415.3046976	0.125
204099_at	SMARCD3	493.8833013	587.3295358	739.9888454	415.3046976	349.2282314	0.125
204115_at	GNG11	493.8833013	48.9994295	739.9888454	466.1637615	523.2511306	0.125
204820_s_at	BTN3A2 /// BTN3A3	493.8833013	466.1637615	587.3295358	164.8137785	493.8833013	0.125
205003_at	DOCK4	493.8833013	739.9888454	523.2511306	415.3046976	349.2282314	0.125
205938_at	PPM1E	493.8833013	659.2551138	349.2282314	932.327523	207.6523488	0.125
205993_s_at	TBX2	493.8833013	554.365262	622.2539674	440	207.6523488	0.125
206059_at	ZNF91	493.8833013	783.990872	466.1637615	523.2511306	220	0.125
206116_s_at	TPM1	493.8833013	493.8833013	830.6093952	466.1637615	293.6647679	0.125
206374_at	DUSP8	493.8833013	698.4564629	391.995436	554.365262	246.9416506	0.125
206572_x_at	ZNF85	493.8833013	554.365262	698.4564629	440	233.0818808	0.125
208829_at	TAPBP	493.8833013	123.4708253	698.4564629	415.3046976	554.365262	0.125
209007_s_at	C1orf63	493.8833013	415.3046976	659.2551138	261.6255653	554.365262	0.125
209198_s_at	SYT11	493.8833013	698.4564629	391.995436	587.3295358	65.40639133	0.125
209201_x_at	CXCR4	493.8833013	830.6093952	739.9888454	277.182631	293.6647679	0.125
209324_s_at	RGS16	493.8833013	523.2511306	440	1046.502261	311.1269837	0.125
209561_at	THBS3	493.8833013	466.1637615	622.2539674	493.8833013	87.30705786	0.125
209897_s_at	SLIT2	493.8833013	622.2539674	369.9944227	246.9416506	880	0.125
209942_x_at	MAGEA3	493.8833013	830.6093952	1396.912926	155.5634919	293.6647679	0.1875
210015_s_at	MAP2	493.8833013	739.9888454	349.2282314	622.2539674	220	0.125
210514_x_at	HLA-G	493.8833013	440	554.365262	293.6647679	739.9888454	0.125
211038_s_at	CROCCP2	493.8833013	523.2511306	739.9888454	311.1269837	440	0.125
211323_s_at	ITPR1	493.8833013	587.3295358	493.8833013	466.1637615	155.5634919	0.125
212062_at	ATP9A	493.8833013	659.2551138	369.9944227	698.4564629	97.998859	0.125
212253_x_at	DST /// LOC100652766	493.8833013	493.8833013	466.1637615	554.365262	82.40688923	0.125
212698_s_at	41527	493.8833013	123.4708253	739.9888454	329.6275569	830.6093952	0.125
212732_at	MEG3	493.8833013	783.990872	61.73541266	932.327523	311.1269837	0.125
212966_at	HIC2	493.8833013	659.2551138	369.9944227	698.4564629	391.995436	0.125
212969_x_at	EML3	493.8833013	493.8833013	698.4564629	277.182631	493.8833013	0.125
213093_at	PRKCA	493.8833013	195.997718	987.7666025	783.990872	311.1269837	0.125
213847_at	PRPH	493.8833013	1760	261.6255653	830.6093952	293.6647679	0.21875
213923_at	RAP2B	493.8833013	440	554.365262	41.20344461	554.365262	0.125

214109_at	LRBA	493.8833013	830.6093952	440	391.995436	523.2511306	0.125
214717_at	PKI55	493.8833013	739.9888454	466.1637615	493.8833013	233.0818808	0.125
214761_at	ZNF423	493.8833013	587.3295358	1174.659072	415.3046976	369.9944227	0.125
215460_x_at	BRD1	493.8833013	880	493.8833013	493.8833013	440	0.125
215767_at	ZNF804A	493.8833013	1174.659072	415.3046976	587.3295358	155.5634919	0.125
217419_x_at	AGRN	493.8833013	391.995436	622.2539674	329.6275569	739.9888454	0.125
217728_at	S100A6	493.8833013	207.6523488	880	466.1637615	493.8833013	0.125
217751_at	GSTK1	493.8833013	261.6255653	739.9888454	493.8833013	466.1637615	0.125
218005_at	ZNF22	493.8833013	164.8137785	783.990872	293.6647679	783.990872	0.125
218935_at	EHD3	493.8833013	440	659.2551138	554.365262	207.6523488	0.125
218989_x_at	SLC30A5	493.8833013	466.1637615	391.995436	493.8833013	783.990872	0.125
219123_at	ZNF232	493.8833013	830.6093952	523.2511306	415.3046976	440	0.125
219236_at	PAQR6	493.8833013	739.9888454	554.365262	391.995436	110	0.125
220150_s_at	FAM184A	493.8833013	698.4564629	349.2282314	659.2551138	311.1269837	0.125
220979_s_at	ST6GALNAC5	493.8833013	466.1637615	493.8833013	659.2551138	220	0.125
221747_at	TNS1	493.8833013	523.2511306	493.8833013	130.8127827	466.1637615	0.125
221750_at	HMGCS1	493.8833013	523.2511306	415.3046976	466.1637615	830.6093952	0.125
200621_at	CSRP1	523.2511306	261.6255653	932.327523	293.6647679	880	0.125
200799_at	HSPA1A /// HSPA1B	523.2511306	155.5634919	622.2539674	415.3046976	622.2539674	0.125
201153_s_at	MBNL1	523.2511306	415.3046976	523.2511306	493.8833013	932.327523	0.125
201300_s_at	PRNP	523.2511306	466.1637615	311.1269837	554.365262	739.9888454	0.125
201791_s_at	DHCR7	523.2511306	523.2511306	493.8833013	523.2511306	880	0.125
201893_x_at	DCN	523.2511306	311.1269837	783.990872	207.6523488	987.7666025	0.125
202104_s_at	SPG7	523.2511306	587.3295358	783.990872	466.1637615	329.6275569	0.125
202759_s_at	AKAP2 /// PALM2-AKAP2	523.2511306	233.0818808	466.1637615	587.3295358	698.4564629	0.125
202893_at	UNC13B	523.2511306	554.365262	523.2511306	880	493.8833013	0.125
202912_at	ADM	523.2511306	1046.502261	698.4564629	138.5913155	369.9944227	0.125
203404_at	ARMCX2	523.2511306	493.8833013	783.990872	523.2511306	207.6523488	0.125
203476_at	TPBG	523.2511306	261.6255653	1046.502261	233.0818808	1108.730524	0.1875
203802_x_at	NSUN5	523.2511306	523.2511306	987.7666025	493.8833013	466.1637615	0.125
203857_s_at	PDIA5	523.2511306	554.365262	493.8833013	466.1637615	1046.502261	0.125
203928_x_at	MAPT	523.2511306	523.2511306	246.9416506	523.2511306	783.990872	0.125
204239_s_at	NNAT	523.2511306	523.2511306	783.990872	493.8833013	261.6255653	0.125
204343_at	ABCA3	523.2511306	659.2551138	523.2511306	466.1637615	246.9416506	0.125

204471_at	GAP43	523.2511306	698.4564629	391.995436	659.2551138	246.9416506	0.125
204620_s_at	VCAN	523.2511306	783.990872	880	207.6523488	329.6275569	0.125
204773_at	IL11RA	523.2511306	523.2511306	783.990872	349.2282314	523.2511306	0.125
205230_at	RPH3A	523.2511306	554.365262	493.8833013	622.2539674	184.9972114	0.125
205262_at	KCNH2	523.2511306	523.2511306	830.6093952	523.2511306	103.8261744	0.125
205268_s_at	ADD2	523.2511306	622.2539674	415.3046976	698.4564629	207.6523488	0.125
205678_at	AP3B2	523.2511306	587.3295358	554.365262	466.1637615	195.997718	0.125
205721_at	GFRA2	523.2511306	1046.502261	523.2511306	277.182631	523.2511306	0.125
205888_s_at	JAKMIP2	523.2511306	493.8833013	415.3046976	523.2511306	880	0.125
207173_x_at	CDH11	523.2511306	164.8137785	622.2539674	440	1396.912926	0.1875
207401_at	PROX1	523.2511306	523.2511306	466.1637615	659.2551138	233.0818808	0.125
208791_at	CLU	523.2511306	1244.507935	783.990872	82.40688923	329.6275569	0.1875
209250_at	DEGS1	523.2511306	830.6093952	466.1637615	466.1637615	554.365262	0.125
209690_s_at	DOK4	523.2511306	622.2539674	783.990872	415.3046976	103.8261744	0.125
210074_at	CTSL2	523.2511306	783.990872	391.995436	587.3295358	440	0.125
211911_x_at	HLA-B	523.2511306	415.3046976	659.2551138	349.2282314	830.6093952	0.125
212212_s_at	INTS1	523.2511306	391.995436	880	659.2551138	391.995436	0.125
212358_at	CLIP3	523.2511306	739.9888454	622.2539674	440	155.5634919	0.125
212359_s_at	KIAA0913	523.2511306	466.1637615	739.9888454	587.3295358	261.6255653	0.125
212397_at	RDX	523.2511306	369.9944227	698.4564629	220	1396.912926	0.1875
212464_s_at	FN1	523.2511306	391.995436	783.990872	73.41619198	659.2551138	0.125
213280_at	RAP1GAP2	523.2511306	698.4564629	554.365262	466.1637615	65.40639133	0.125
213421_x_at	PRSS3	523.2511306	369.9944227	698.4564629	783.990872	261.6255653	0.125
213439_x_at	RUNDC3A	523.2511306	587.3295358	440	587.3295358	155.5634919	0.125
213460_x_at	NSUN5P2	523.2511306	554.365262	880	440	493.8833013	0.125
213489_at	MAPRE2	523.2511306	587.3295358	466.1637615	932.327523	369.9944227	0.125
213496_at	LPPR4	523.2511306	659.2551138	554.365262	493.8833013	207.6523488	0.125
214100_x_at	NSUN5P1	523.2511306	554.365262	987.7666025	466.1637615	440	0.125
214459_x_at	HLA-C	523.2511306	349.2282314	698.4564629	311.1269837	830.6093952	0.125
214577_at	MAP1B	523.2511306	587.3295358	466.1637615	622.2539674	207.6523488	0.125
214636_at	CALCB	523.2511306	1174.659072	246.9416506	311.1269837	880	0.125
218049_s_at	LOC100506980 /// MRPL13	523.2511306	493.8833013	369.9944227	523.2511306	783.990872	0.125
218170_at	ISOC1	523.2511306	493.8833013	1479.977691	466.1637615	523.2511306	0.1875
218394_at	ROGDI	523.2511306	466.1637615	554.365262	783.990872	233.0818808	0.125
218603_at	HECA	523.2511306	587.3295358	391.995436	466.1637615	987.7666025	0.125

219275_at	PDCD5	523.2511306	523.2511306	391.995436	493.8833013	880	0.125
219338_s_at	LRRC49	523.2511306	466.1637615	554.365262	698.4564629	220	0.125
219410_at	TMEM45A	523.2511306	246.9416506	493.8833013	554.365262	783.990872	0.125
219425_at	SULT4A1	523.2511306	622.2539674	440	659.2551138	174.6141157	0.125
219498_s_at	BCL11A	523.2511306	783.990872	415.3046976	659.2551138	195.997718	0.125
221011_s_at	LBH	523.2511306	466.1637615	554.365262	466.1637615	1864.655046	0.21875
221269_s_at	SH3BGRL3	523.2511306	369.9944227	783.990872	493.8833013	554.365262	0.125
221427_s_at	CCNL2	523.2511306	440	880	466.1637615	587.3295358	0.125
221725_at	WASF2	523.2511306	369.9944227	659.2551138	349.2282314	698.4564629	0.125
222005_s_at	GNG3	523.2511306	739.9888454	369.9944227	932.327523	123.4708253	0.125
31846_at	RHOD	523.2511306	587.3295358	493.8833013	261.6255653	698.4564629	0.125
201005_at	CD9 /// LOC100652804 /// LOC100653288	554.365262	987.7666025	523.2511306	554.365262	391.995436	0.125
201125_s_at	ITGB5	554.365262	523.2511306	349.2282314	622.2539674	880	0.125
201539_s_at	FHL1	554.365262	622.2539674	932.327523	493.8833013	329.6275569	0.125
201580_s_at	TMX4	554.365262	466.1637615	622.2539674	830.6093952	466.1637615	0.125
201616_s_at	CALD1	554.365262	587.3295358	739.9888454	554.365262	103.8261744	0.125
202219_at	SLC6A8	554.365262	587.3295358	739.9888454	311.1269837	523.2511306	0.125
202419_at	KDSR	554.365262	587.3295358	493.8833013	466.1637615	1244.507935	0.125
202626_s_at	LYN	554.365262	349.2282314	987.7666025	880	164.8137785	0.125
202704_at	TOB1	554.365262	880	587.3295358	523.2511306	329.6275569	0.125
202762_at	ROCK2	554.365262	554.365262	493.8833013	1174.659072	466.1637615	0.125
202887_s_at	DDIT4	554.365262	311.1269837	466.1637615	622.2539674	932.327523	0.125
202932_at	YES1	554.365262	659.2551138	493.8833013	103.8261744	587.3295358	0.125
203456_at	PRAF2	554.365262	587.3295358	739.9888454	554.365262	311.1269837	0.125
203511_s_at	TRAPPC3	554.365262	391.995436	587.3295358	466.1637615	830.6093952	0.125
203685_at	BCL2	554.365262	880	622.2539674	466.1637615	329.6275569	0.125
203724_s_at	RUFY3	554.365262	783.990872	415.3046976	698.4564629	92.49860568	0.125
203725_at	GADD45A	554.365262	622.2539674	493.8833013	659.2551138	277.182631	0.125
203813_s_at	SLIT3	554.365262	698.4564629	1046.502261	440	87.30705786	0.125
203957_at	E2F6	554.365262	554.365262	523.2511306	987.7666025	554.365262	0.125
204184_s_at	ADRBK2	554.365262	554.365262	493.8833013	659.2551138	184.9972114	0.125
204454_at	LDOC1	554.365262	523.2511306	587.3295358	698.4564629	207.6523488	0.125
204550_x_at	GSTM1	554.365262	493.8833013	587.3295358	523.2511306	932.327523	0.125

204602_at	DKK1	554.365262	391.995436	1108.730524	739.9888454	69.29565774	0.125
204695_at	CDC25A	554.365262	466.1637615	391.995436	830.6093952	587.3295358	0.125
204953_at	SNAP91	554.365262	739.9888454	554.365262	523.2511306	73.41619198	0.125
204975_at	EMP2	554.365262	587.3295358	523.2511306	174.6141157	554.365262	0.125
205320_at	APC2	554.365262	587.3295358	622.2539674	523.2511306	184.9972114	0.125
205462_s_at	HPCAL1	554.365262	415.3046976	698.4564629	1108.730524	293.6647679	0.125
205522_at	HOXD4	554.365262	659.2551138	466.1637615	987.7666025	391.995436	0.125
205591_at	OLFM1	554.365262	698.4564629	466.1637615	261.6255653	622.2539674	0.125
206401_s_at	MAPT	554.365262	554.365262	261.6255653	523.2511306	830.6093952	0.125
207056_s_at	SLC4A8	554.365262	587.3295358	523.2511306	587.3295358	73.41619198	0.125
209822_s_at	VLDLR	554.365262	659.2551138	523.2511306	554.365262	233.0818808	0.125
210070_s_at	CHKB-CPT1B /// CPT1B	554.365262	659.2551138	587.3295358	466.1637615	164.8137785	0.125
210124_x_at	SEMA4F	554.365262	739.9888454	523.2511306	554.365262	311.1269837	0.125
210762_s_at	DLC1	554.365262	783.990872	391.995436	184.9972114	880	0.125
210794_s_at	MEG3	554.365262	880	77.78174593	1244.507935	329.6275569	0.1875
211162_x_at	SCD	554.365262	554.365262	311.1269837	554.365262	783.990872	0.125
212174_at	AK2	554.365262	440	698.4564629	415.3046976	932.327523	0.125
212192_at	KCTD12	554.365262	1318.510228	932.327523	51.9130872	329.6275569	0.1875
212294_at	GNG12	554.365262	659.2551138	659.2551138	440	220	0.125
212761_at	TCF7L2	554.365262	493.8833013	1108.730524	349.2282314	587.3295358	0.125
212848_s_at	C9orf3 /// LOC100507319	554.365262	587.3295358	493.8833013	698.4564629	164.8137785	0.125
212935_at	MCF2L	554.365262	554.365262	622.2539674	587.3295358	82.40688923	0.125
213273_at	ODZ4	554.365262	587.3295358	659.2551138	493.8833013	220	0.125
213746_s_at	FLNA	554.365262	391.995436	783.990872	415.3046976	698.4564629	0.125
214113_s_at	RBM8A	554.365262	880	466.1637615	587.3295358	523.2511306	0.125
214298_x_at	41523	554.365262	622.2539674	466.1637615	830.6093952	261.6255653	0.125
214811_at	RIMBP2	554.365262	739.9888454	659.2551138	440	184.9972114	0.125
214825_at	FAM155A	554.365262	554.365262	554.365262	698.4564629	246.9416506	0.125
215273_s_at	TADA3	554.365262	554.365262	830.6093952	554.365262	415.3046976	0.125
215771_x_at	RET	554.365262	587.3295358	523.2511306	987.7666025	329.6275569	0.125
217047_s_at	FAM13A	554.365262	554.365262	391.995436	987.7666025	554.365262	0.125
218162_at	OLFML3	554.365262	466.1637615	622.2539674	415.3046976	1396.912926	0.125
218529_at	CD320	554.365262	493.8833013	554.365262	554.365262	880	0.125
218856_at	TNFRSF21	554.365262	659.2551138	554.365262	493.8833013	207.6523488	0.125

219277_s_at	OGDHL	554.365262	466.1637615	622.2539674	880	466.1637615	0.125
219463_at	LAMP5	554.365262	349.2282314	880	932.327523	146.832384	0.125
219489_s_at	NXN	554.365262	698.4564629	587.3295358	164.8137785	493.8833013	0.125
219819_s_at	MRPS28	554.365262	523.2511306	523.2511306	554.365262	932.327523	0.125
219956_at	GALNT6	554.365262	698.4564629	440	1046.502261	349.2282314	0.125
220731_s_at	NECAP2	554.365262	369.9944227	698.4564629	440	698.4564629	0.125
220974_x_at	SFXN3	554.365262	554.365262	659.2551138	554.365262	164.8137785	0.125
221207_s_at	NBEA	554.365262	622.2539674	698.4564629	466.1637615	311.1269837	0.125
221236_s_at	STMN4	554.365262	830.6093952	349.2282314	880	369.9944227	0.125
221489_s_at	SPRY4	554.365262	415.3046976	698.4564629	698.4564629	233.0818808	0.125
221701_s_at	STRA6	554.365262	466.1637615	659.2551138	830.6093952	349.2282314	0.125
221814_at	GPR124	554.365262	415.3046976	698.4564629	369.9944227	783.990872	0.125
221904_at	FAM131A	554.365262	493.8833013	587.3295358	830.6093952	440	0.125
221908_at	RNFT2	554.365262	622.2539674	493.8833013	622.2539674	246.9416506	0.125
36936_at	TSTA3	554.365262	440	587.3295358	493.8833013	880	0.125
40837_at	TLE2	554.365262	659.2551138	293.6647679	880	466.1637615	0.125
200035_at	CTDNEP1	587.3295358	659.2551138	880	493.8833013	523.2511306	0.125
200788_s_at	PEA15	587.3295358	622.2539674	1174.659072	554.365262	523.2511306	0.125
201287_s_at	SDC1	587.3295358	440	783.990872	830.6093952	233.0818808	0.125
201627_s_at	INSIG1	587.3295358	698.4564629	466.1637615	466.1637615	830.6093952	0.125
201790_s_at	DHCR7	587.3295358	493.8833013	587.3295358	587.3295358	932.327523	0.125
202011_at	TJP1	587.3295358	622.2539674	830.6093952	587.3295358	73.41619198	0.125
202036_s_at	SFRP1	587.3295358	830.6093952	493.8833013	659.2551138	103.8261744	0.125
202973_x_at	FAM13A	587.3295358	587.3295358	391.995436	1046.502261	587.3295358	0.125
203037_s_at	MTSS1	587.3295358	698.4564629	349.2282314	1108.730524	523.2511306	0.125
203349_s_at	ETV5	587.3295358	369.9944227	659.2551138	880	493.8833013	0.125
203633_at	CPT1A	587.3295358	587.3295358	554.365262	493.8833013	880	0.125
203790_s_at	HRSP12	587.3295358	523.2511306	523.2511306	659.2551138	1174.659072	0.125
204238_s_at	C6orf108	587.3295358	415.3046976	587.3295358	523.2511306	830.6093952	0.125
204811_s_at	CACNA2D2	587.3295358	466.1637615	830.6093952	739.9888454	293.6647679	0.125
204872_at	TLE4	587.3295358	830.6093952	622.2539674	523.2511306	73.41619198	0.125
204916_at	RAMP1	587.3295358	493.8833013	493.8833013	880	698.4564629	0.125
205381_at	LRRC17	587.3295358	783.990872	587.3295358	138.5913155	523.2511306	0.125
205822_s_at	HMGCS1	587.3295358	659.2551138	369.9944227	493.8833013	987.7666025	0.125
205933_at	SETBP1	587.3295358	659.2551138	466.1637615	698.4564629	61.73541266	0.125
205986_at	AATK	587.3295358	622.2539674	493.8833013	622.2539674	246.9416506	0.125

206045_s_at	NOL4	587.3295358	622.2539674	554.365262	659.2551138	155.5634919	0.125
206196_s_at	RUNDC3A	587.3295358	880	493.8833013	622.2539674	110	0.125
207761_s_at	METTL7A	587.3295358	783.990872	830.6093952	293.6647679	466.1637615	0.125
208650_s_at	CD24	587.3295358	932.327523	622.2539674	523.2511306	293.6647679	0.125
208850_s_at	THY1	587.3295358	523.2511306	932.327523	622.2539674	523.2511306	0.125
209164_s_at	CYB561	587.3295358	391.995436	830.6093952	987.7666025	329.6275569	0.125
209209_s_at	FERMT2	587.3295358	698.4564629	739.9888454	523.2511306	329.6275569	0.125
209286_at	CDC42EP3	587.3295358	830.6093952	783.990872	415.3046976	82.40688923	0.125
209340_at	UAP1	587.3295358	587.3295358	554.365262	554.365262	1396.912926	0.125
209568_s_at	RGL1	587.3295358	493.8833013	622.2539674	523.2511306	987.7666025	0.125
209583_s_at	CD200	587.3295358	739.9888454	587.3295358	554.365262	155.5634919	0.125
209602_s_at	GATA3	587.3295358	830.6093952	739.9888454	466.1637615	97.998859	0.125
209871_s_at	APBA2	587.3295358	554.365262	739.9888454	587.3295358	65.40639133	0.125
210508_s_at	KCNQ2	587.3295358	554.365262	554.365262	880	164.8137785	0.125
210830_s_at	PON2	587.3295358	65.40639133	554.365262	587.3295358	587.3295358	0.125
211467_s_at	NFIB	587.3295358	659.2551138	880	554.365262	369.9944227	0.125
211529_x_at	HLA-G	587.3295358	493.8833013	659.2551138	369.9944227	830.6093952	0.125
211919_s_at	CXCR4	587.3295358	932.327523	830.6093952	293.6647679	391.995436	0.125
212158_at	SDC2	587.3295358	880	698.4564629	415.3046976	466.1637615	0.125
212299_at	NEK9	587.3295358	110	830.6093952	698.4564629	493.8833013	0.125
212609_s_at	AKT3	587.3295358	622.2539674	622.2539674	523.2511306	92.49860568	0.125
212612_at	RCOR1	587.3295358	622.2539674	493.8833013	554.365262	932.327523	0.125
212730_at	SYNM	587.3295358	783.990872	587.3295358	587.3295358	233.0818808	0.125
212956_at	TBC1D9	587.3295358	587.3295358	554.365262	622.2539674	932.327523	0.125
213030_s_at	PLXNA2	587.3295358	493.8833013	622.2539674	830.6093952	146.832384	0.125
213170_at	GPX7	587.3295358	110	622.2539674	554.365262	659.2551138	0.125
213386_at	TMEM246	587.3295358	698.4564629	523.2511306	659.2551138	246.9416506	0.125
213484_at	---	587.3295358	1046.502261	622.2539674	523.2511306	155.5634919	0.125
213666_at	41523	587.3295358	698.4564629	493.8833013	1108.730524	246.9416506	0.125
214432_at	ATP1A3	587.3295358	622.2539674	554.365262	659.2551138	174.6141157	0.125
215440_s_at	BEX4	587.3295358	783.990872	659.2551138	523.2511306	311.1269837	0.125
215706_x_at	ZYX	587.3295358	783.990872	783.990872	391.995436	440	0.125
216086_at	SV2C	587.3295358	659.2551138	523.2511306	659.2551138	233.0818808	0.125
216526_x_at	HLA-C	587.3295358	311.1269837	1046.502261	195.997718	1244.507935	0.1875
216963_s_at	GAP43	587.3295358	739.9888454	440	739.9888454	329.6275569	0.125
217731_s_at	ITM2B	587.3295358	932.327523	554.365262	622.2539674	523.2511306	0.125

217959_s_at	MIR3656 /// TRAPPC4	587.3295358	391.995436	830.6093952	587.3295358	554.365262	0.125
217979_at	TSPAN13	587.3295358	293.6647679	440	783.990872	1396.912926	0.1875
218094_s_at	DBNDD2 /// SYS1 /// SYS1-DBNDD2	587.3295358	493.8833013	391.995436	659.2551138	783.990872	0.125
218962_s_at	TMEM168	587.3295358	61.73541266	554.365262	587.3295358	622.2539674	0.125
219032_x_at	OPN3	587.3295358	277.182631	783.990872	415.3046976	830.6093952	0.125
219438_at	NKAIN1	587.3295358	554.365262	659.2551138	587.3295358	261.6255653	0.125
219558_at	ATP13A3	587.3295358	466.1637615	369.9944227	698.4564629	739.9888454	0.125
221582_at	HIST3H2A	587.3295358	622.2539674	880	523.2511306	293.6647679	0.125
221728_x_at	XIST	587.3295358	220	1318.510228	246.9416506	1396.912926	0.21875
221766_s_at	FAM46A	587.3295358	466.1637615	440	830.6093952	739.9888454	0.125
222155_s_at	SLC52A2	587.3295358	293.6647679	739.9888454	523.2511306	659.2551138	0.125
40850_at	FKBP8	587.3295358	174.6141157	554.365262	587.3295358	587.3295358	0.125
55872_at	ZNF512B	587.3295358	830.6093952	698.4564629	466.1637615	466.1637615	0.125
200701_at	NPC2	622.2539674	587.3295358	1046.502261	277.182631	698.4564629	0.125
200911_s_at	TACC1	622.2539674	523.2511306	466.1637615	698.4564629	1108.730524	0.125
201011_at	RPN1	622.2539674	554.365262	493.8833013	659.2551138	987.7666025	0.125
201341_at	ENC1	622.2539674	587.3295358	659.2551138	622.2539674	110	0.125
201368_at	ZFP36L2	622.2539674	698.4564629	830.6093952	554.365262	391.995436	0.125
201431_s_at	DPYSL3	622.2539674	261.6255653	698.4564629	783.990872	554.365262	0.125
201508_at	IGFBP4	622.2539674	369.9944227	987.7666025	391.995436	1174.659072	0.125
201746_at	TP53	622.2539674	659.2551138	987.7666025	493.8833013	587.3295358	0.125
201876_at	PON2	622.2539674	155.5634919	830.6093952	622.2539674	587.3295358	0.125
202145_at	LY6E	622.2539674	349.2282314	554.365262	659.2551138	1479.977691	0.1875
202242_at	TSPAN7	622.2539674	1174.659072	415.3046976	932.327523	311.1269837	0.125
202562_s_at	C14orf1	622.2539674	622.2539674	493.8833013	622.2539674	987.7666025	0.125
202651_at	LPGAT1	622.2539674	739.9888454	523.2511306	698.4564629	329.6275569	0.125
202712_s_at	CKMT1A /// CKMT1B	622.2539674	698.4564629	523.2511306	932.327523	220	0.125
202802_at	DHPS	622.2539674	415.3046976	698.4564629	783.990872	493.8833013	0.125
202920_at	ANK2	622.2539674	739.9888454	523.2511306	1174.659072	293.6647679	0.125
202946_s_at	BTBD3	622.2539674	739.9888454	554.365262	932.327523	415.3046976	0.125
203274_at	F8A1 /// F8A2 /// F8A3	622.2539674	1046.502261	587.3295358	587.3295358	554.365262	0.125

203466_at	MPV17	622.2539674	622.2539674	880	587.3295358	391.995436	0.125
203710_at	ITPR1	622.2539674	698.4564629	587.3295358	622.2539674	246.9416506	0.125
203880_at	COX17	622.2539674	523.2511306	493.8833013	987.7666025	698.4564629	0.125
203929_s_at	MAPT	622.2539674	783.990872	391.995436	554.365262	739.9888454	0.125
204019_s_at	SH3YL1	622.2539674	880	659.2551138	391.995436	554.365262	0.125
204105_s_at	NRCAM	622.2539674	466.1637615	880	783.990872	261.6255653	0.125
204237_at	GULP1	622.2539674	987.7666025	698.4564629	554.365262	220	0.125
204520_x_at	BRD1	622.2539674	932.327523	659.2551138	587.3295358	554.365262	0.125
204556_s_at	DZIP1	622.2539674	622.2539674	622.2539674	659.2551138	195.997718	0.125
204850_s_at	DCX	622.2539674	311.1269837	698.4564629	1318.510228	523.2511306	0.125
207826_s_at	ID3	622.2539674	523.2511306	739.9888454	440	987.7666025	0.125
208623_s_at	EZR	622.2539674	622.2539674	415.3046976	587.3295358	880	0.125
209013_x_at	TRIO	622.2539674	783.990872	1108.730524	493.8833013	369.9944227	0.125
209030_s_at	CADM1	622.2539674	493.8833013	739.9888454	1108.730524	277.182631	0.125
209045_at	XPNPEP1	622.2539674	587.3295358	659.2551138	987.7666025	554.365262	0.125
209242_at	PEG3	622.2539674	739.9888454	587.3295358	659.2551138	293.6647679	0.125
209287_s_at	CDC42EP3	622.2539674	932.327523	830.6093952	440	220	0.125
209757_s_at	MYCN	622.2539674	1975.533205	246.9416506	1479.977691	207.6523488	0.25
209772_s_at	CD24	622.2539674	1046.502261	698.4564629	554.365262	233.0818808	0.125
209870_s_at	APBA2	622.2539674	622.2539674	587.3295358	783.990872	97.998859	0.125
210022_at	PCGF1	622.2539674	880	622.2539674	622.2539674	466.1637615	0.125
210754_s_at	LYN	622.2539674	440	932.327523	932.327523	311.1269837	0.125
210839_s_at	ENPP2	622.2539674	1244.507935	311.1269837	1396.912926	164.8137785	0.1875
211708_s_at	SCD	622.2539674	622.2539674	493.8833013	622.2539674	932.327523	0.125
212096_s_at	MTUS1	622.2539674	1046.502261	523.2511306	739.9888454	369.9944227	0.125
212149_at	EFR3A	622.2539674	622.2539674	523.2511306	587.3295358	987.7666025	0.125
212169_at	FKBP9	622.2539674	440	880	587.3295358	622.2539674	0.125
212185_x_at	MT2A	622.2539674	466.1637615	987.7666025	369.9944227	830.6093952	0.125
212188_at	KCTD12	622.2539674	1396.912926	1174.659072	103.8261744	311.1269837	0.1875
212190_at	SERPINE2	622.2539674	880	466.1637615	466.1637615	783.990872	0.125
212285_s_at	AGRN	622.2539674	493.8833013	783.990872	311.1269837	830.6093952	0.125
212692_s_at	LRBA	622.2539674	1174.659072	493.8833013	622.2539674	659.2551138	0.125
212724_at	RND3	622.2539674	587.3295358	880	659.2551138	349.2282314	0.125
212736_at	C16orf45	622.2539674	659.2551138	622.2539674	622.2539674	123.4708253	0.125
212767_at	MTG1	622.2539674	554.365262	880	493.8833013	698.4564629	0.125
212936_at	FAM172A	622.2539674	622.2539674	587.3295358	932.327523	466.1637615	0.125

212958_x_at	PAM	622.2539674	523.2511306	739.9888454	932.327523	523.2511306	0.125
212979_s_at	FAM115A /// LOC100294033	622.2539674	880	698.4564629	554.365262	440	0.125
213100_at	UNC5B	622.2539674	587.3295358	830.6093952	698.4564629	415.3046976	0.125
213222_at	PLCB1	622.2539674	783.990872	554.365262	622.2539674	246.9416506	0.125
213358_at	SOGA2	622.2539674	659.2551138	587.3295358	830.6093952	293.6647679	0.125
213417_at	TBX2	622.2539674	698.4564629	622.2539674	587.3295358	87.30705786	0.125
213449_at	POP1	622.2539674	554.365262	329.6275569	698.4564629	880	0.125
213768_s_at	ASCL1	622.2539674	1046.502261	830.6093952	440	138.5913155	0.125
213947_s_at	NUP210	622.2539674	587.3295358	622.2539674	659.2551138	246.9416506	0.125
213977_s_at	CIZ1	622.2539674	830.6093952	739.9888454	523.2511306	466.1637615	0.125
215566_x_at	LYPLA2	622.2539674	440	880	587.3295358	622.2539674	0.125
216449_x_at	HSP90B1	622.2539674	554.365262	554.365262	698.4564629	932.327523	0.125
218353_at	RGS5	622.2539674	1244.507935	2093.004522	293.6647679	220	0.25
218599_at	REC8	622.2539674	277.182631	932.327523	1046.502261	369.9944227	0.125
218862_at	ASB13	622.2539674	554.365262	739.9888454	659.2551138	311.1269837	0.125
218905_at	INTS8	622.2539674	466.1637615	587.3295358	659.2551138	987.7666025	0.125
218974_at	SOBP	622.2539674	659.2551138	415.3046976	932.327523	554.365262	0.125
219288_at	C3orf14	622.2539674	587.3295358	659.2551138	1046.502261	466.1637615	0.125
219779_at	ZFHX4	622.2539674	659.2551138	554.365262	277.182631	1046.502261	0.125
220094_s_at	CCDC90A	622.2539674	554.365262	587.3295358	622.2539674	1046.502261	0.125
220688_s_at	MRTO4	622.2539674	493.8833013	622.2539674	587.3295358	1108.730524	0.125
221214_s_at	NELF	622.2539674	523.2511306	932.327523	739.9888454	493.8833013	0.125
35626_at	SGSH	622.2539674	659.2551138	783.990872	554.365262	369.9944227	0.125
54970_at	ZMIZ2	622.2539674	622.2539674	932.327523	466.1637615	659.2551138	0.125
200616_s_at	MLEC	659.2551138	415.3046976	587.3295358	698.4564629	830.6093952	0.125
200920_s_at	BTG1	659.2551138	932.327523	698.4564629	554.365262	554.365262	0.125
200982_s_at	ANXA6	659.2551138	622.2539674	493.8833013	659.2551138	1244.507935	0.125
200984_s_at	CD59	659.2551138	659.2551138	987.7666025	622.2539674	659.2551138	0.125
201028_s_at	CD99	659.2551138	233.0818808	622.2539674	698.4564629	1318.510228	0.125
201559_s_at	CLIC4	659.2551138	622.2539674	622.2539674	391.995436	1174.659072	0.125
201564_s_at	FSCN1 /// LOC100653350	659.2551138	622.2539674	1174.659072	659.2551138	523.2511306	0.125
201649_at	UBE2L6	659.2551138	783.990872	783.990872	523.2511306	440	0.125
201681_s_at	DLG5	659.2551138	739.9888454	622.2539674	659.2551138	311.1269837	0.125
201830_s_at	NET1	659.2551138	880	493.8833013	739.9888454	554.365262	0.125

202239_at	PARP4	659.2551138	739.9888454	622.2539674	277.182631	659.2551138	0.125
202336_s_at	PAM	659.2551138	466.1637615	932.327523	932.327523	440	0.125
202371_at	TCEAL4	659.2551138	830.6093952	698.4564629	587.3295358	415.3046976	0.125
202409_at	IGF2 /// INS-IGF2	659.2551138	739.9888454	587.3295358	1975.533205	311.1269837	0.21875
202421_at	IGSF3	659.2551138	698.4564629	622.2539674	277.182631	698.4564629	0.125
202501_at	MAPRE2	659.2551138	739.9888454	587.3295358	987.7666025	587.3295358	0.125
202582_s_at	RANBP9	659.2551138	659.2551138	622.2539674	622.2539674	987.7666025	0.125
203066_at	CHST15	659.2551138	1046.502261	440	987.7666025	146.832384	0.125
203069_at	SV2A	659.2551138	783.990872	554.365262	830.6093952	293.6647679	0.125
203440_at	CDH2	659.2551138	739.9888454	698.4564629	587.3295358	164.8137785	0.125
203853_s_at	GAB2	659.2551138	783.990872	587.3295358	698.4564629	220	0.125
204072_s_at	FRY	659.2551138	698.4564629	587.3295358	783.990872	123.4708253	0.125
204092_s_at	AURKA	659.2551138	587.3295358	415.3046976	739.9888454	1046.502261	0.125
204274_at	EBAG9	659.2551138	622.2539674	440	659.2551138	987.7666025	0.125
204396_s_at	GRK5	659.2551138	783.990872	523.2511306	369.9944227	830.6093952	0.125
204510_at	CDC7	659.2551138	1046.502261	622.2539674	466.1637615	698.4564629	0.125
204565_at	ACOT13	659.2551138	466.1637615	554.365262	739.9888454	880	0.125
204566_at	PPM1D	659.2551138	932.327523	622.2539674	659.2551138	440	0.125
204822_at	TTK	659.2551138	554.365262	554.365262	698.4564629	932.327523	0.125
205229_s_at	COCH	659.2551138	369.9944227	554.365262	739.9888454	783.990872	0.125
205573_s_at	SNX7	659.2551138	554.365262	739.9888454	987.7666025	523.2511306	0.125
206857_s_at	FKBP1B	659.2551138	739.9888454	622.2539674	698.4564629	293.6647679	0.125
207410_s_at	TLX2	659.2551138	739.9888454	698.4564629	622.2539674	48.9994295	0.125
207714_s_at	SERPINH1	659.2551138	523.2511306	783.990872	369.9944227	987.7666025	0.125
207747_s_at	DOK4	659.2551138	783.990872	932.327523	554.365262	195.997718	0.125
208178_x_at	TRIO	659.2551138	783.990872	1108.730524	523.2511306	293.6647679	0.125
208453_s_at	XPNPEP1	659.2551138	659.2551138	659.2551138	1046.502261	554.365262	0.125
208666_s_at	ST13	659.2551138	987.7666025	415.3046976	659.2551138	659.2551138	0.125
208951_at	ALDH7A1	659.2551138	587.3295358	466.1637615	698.4564629	932.327523	0.125
209011_at	TRIO	659.2551138	830.6093952	1318.510228	523.2511306	369.9944227	0.125
209051_s_at	RALGDS	659.2551138	698.4564629	739.9888454	622.2539674	349.2282314	0.125
209096_at	UBE2V2	659.2551138	587.3295358	523.2511306	698.4564629	932.327523	0.125
209163_at	CYB561	659.2551138	349.2282314	1174.659072	1318.510228	184.9972114	0.1875
209191_at	TUBB6	659.2551138	622.2539674	493.8833013	698.4564629	1396.912926	0.125
209316_s_at	HBS1L	659.2551138	587.3295358	554.365262	698.4564629	932.327523	0.125

209376_x_at	SCAF11	659.2551138	659.2551138	493.8833013	698.4564629	932.327523	0.125
209462_at	APLP1	659.2551138	659.2551138	622.2539674	880	311.1269837	0.125
209691_s_at	DOK4	659.2551138	830.6093952	932.327523	523.2511306	220	0.125
210788_s_at	DHRS7	659.2551138	659.2551138	622.2539674	1174.659072	329.6275569	0.125
210933_s_at	FSCN1 /// LOC100653350	659.2551138	587.3295358	1108.730524	698.4564629	622.2539674	0.125
211719_x_at	FN1	659.2551138	554.365262	880	77.78174593	783.990872	0.125
211959_at	IGFBP5	659.2551138	1567.981744	1046.502261	220	391.995436	0.1875
212248_at	MTDH	659.2551138	659.2551138	493.8833013	622.2539674	1046.502261	0.125
212309_at	CLASP2	659.2551138	783.990872	587.3295358	739.9888454	391.995436	0.125
212553_at	RPRD2	659.2551138	830.6093952	698.4564629	587.3295358	391.995436	0.125
212593_s_at	MIR4680 /// PDCD4	659.2551138	587.3295358	739.9888454	1046.502261	440	0.125
212655_at	ZCCHC14	659.2551138	739.9888454	783.990872	554.365262	329.6275569	0.125
212675_s_at	CEP68	659.2551138	987.7666025	622.2539674	659.2551138	415.3046976	0.125
212694_s_at	PCCB	659.2551138	622.2539674	622.2539674	659.2551138	987.7666025	0.125
212720_at	PAPOLA	659.2551138	739.9888454	440	554.365262	830.6093952	0.125
212741_at	MAOA	659.2551138	1396.912926	932.327523	440	293.6647679	0.1875
213931_at	ID2 /// ID2B	659.2551138	698.4564629	932.327523	369.9944227	622.2539674	0.125
215913_s_at	GULP1	659.2551138	880	698.4564629	554.365262	261.6255653	0.125
217761_at	ADI1	659.2551138	880	659.2551138	466.1637615	587.3295358	0.125
217870_s_at	CMPK1	659.2551138	659.2551138	622.2539674	622.2539674	1046.502261	0.125
218164_at	SPATA20	659.2551138	659.2551138	880	622.2539674	329.6275569	0.125
218185_s_at	ARMC1	659.2551138	622.2539674	523.2511306	659.2551138	1046.502261	0.125
218330_s_at	NAV2	659.2551138	659.2551138	622.2539674	698.4564629	116.5409404	0.125
218376_s_at	MICAL1	659.2551138	739.9888454	622.2539674	659.2551138	277.182631	0.125
218384_at	CARHSP1	659.2551138	554.365262	698.4564629	587.3295358	1396.912926	0.125
218782_s_at	ATAD2	659.2551138	622.2539674	391.995436	659.2551138	830.6093952	0.125
218953_s_at	PCYOX1L	659.2551138	659.2551138	622.2539674	739.9888454	277.182631	0.125
219649_at	ALG6	659.2551138	554.365262	587.3295358	698.4564629	987.7666025	0.125
220486_x_at	TMEM164	659.2551138	698.4564629	987.7666025	523.2511306	622.2539674	0.125
221748_s_at	TNS1	659.2551138	698.4564629	739.9888454	329.6275569	659.2551138	0.125
221760_at	MAN1A1	659.2551138	739.9888454	554.365262	830.6093952	207.6523488	0.125
221933_at	NLGN4X	659.2551138	932.327523	493.8833013	103.8261744	880	0.125
201152_s_at	MBNL1	698.4564629	587.3295358	739.9888454	587.3295358	1046.502261	0.125
201418_s_at	SOX4	698.4564629	587.3295358	880	493.8833013	830.6093952	0.125

201560_at	CLIC4	698.4564629	698.4564629	698.4564629	523.2511306	1244.507935	0.125
201609_x_at	ICMT	698.4564629	523.2511306	739.9888454	659.2551138	987.7666025	0.125
201968_s_at	PGM1	698.4564629	659.2551138	830.6093952	739.9888454	415.3046976	0.125
202016_at	MEST	698.4564629	659.2551138	739.9888454	783.990872	391.995436	0.125
202096_s_at	TSPO	698.4564629	369.9944227	783.990872	587.3295358	1108.730524	0.125
202111_at	SLC4A2	698.4564629	369.9944227	932.327523	659.2551138	698.4564629	0.125
202117_at	ARHGAP1	698.4564629	830.6093952	987.7666025	587.3295358	587.3295358	0.125
202185_at	PLOD3	698.4564629	698.4564629	1174.659072	698.4564629	698.4564629	0.125
202363_at	SPOCK1	698.4564629	783.990872	554.365262	830.6093952	92.49860568	0.125
202508_s_at	SNAP25	698.4564629	880	554.365262	830.6093952	277.182631	0.125
202706_s_at	UMPS	698.4564629	659.2551138	622.2539674	739.9888454	1046.502261	0.125
203408_s_at	SATB1	698.4564629	783.990872	1046.502261	622.2539674	391.995436	0.125
203411_s_at	LMNA	698.4564629	587.3295358	880	415.3046976	783.990872	0.125
203562_at	FEZ1	698.4564629	659.2551138	698.4564629	880	184.9972114	0.125
204066_s_at	AGAP1	698.4564629	1046.502261	659.2551138	523.2511306	739.9888454	0.125
204224_s_at	GCH1	698.4564629	415.3046976	698.4564629	1174.659072	622.2539674	0.125
204418_x_at	GSTM2	698.4564629	659.2551138	698.4564629	587.3295358	1046.502261	0.125
204480_s_at	C9orf16	698.4564629	659.2551138	987.7666025	739.9888454	554.365262	0.125
204720_s_at	DNAJC6	698.4564629	783.990872	587.3295358	783.990872	391.995436	0.125
205113_at	NEFM	698.4564629	622.2539674	739.9888454	2217.461048	61.73541266	0.375
205184_at	GNG4	698.4564629	987.7666025	659.2551138	698.4564629	261.6255653	0.125
205770_at	GSR	698.4564629	698.4564629	466.1637615	659.2551138	987.7666025	0.125
206767_at	RBMS3	698.4564629	698.4564629	830.6093952	698.4564629	311.1269837	0.125
206858_s_at	HOXC6	698.4564629	311.1269837	554.365262	880	830.6093952	0.125
207076_s_at	ASS1	698.4564629	466.1637615	73.41619198	1479.977691	1046.502261	0.1875
207986_x_at	CYB561	698.4564629	466.1637615	932.327523	1479.977691	184.9972114	0.1875
208079_s_at	AURKA	698.4564629	698.4564629	493.8833013	739.9888454	932.327523	0.125
208711_s_at	CCND1	698.4564629	739.9888454	659.2551138	554.365262	1046.502261	0.125
208786_s_at	MAP1LC3B	698.4564629	880	698.4564629	739.9888454	466.1637615	0.125
209146_at	MSMO1	698.4564629	698.4564629	493.8833013	698.4564629	1174.659072	0.125
209288_s_at	CDC42EP3	698.4564629	987.7666025	880	523.2511306	116.5409404	0.125
209353_s_at	---	698.4564629	783.990872	739.9888454	659.2551138	138.5913155	0.125
209385_s_at	PROSC	698.4564629	659.2551138	622.2539674	698.4564629	1108.730524	0.125
209579_s_at	MBD4	698.4564629	698.4564629	523.2511306	659.2551138	987.7666025	0.125
210298_x_at	FHL1	698.4564629	698.4564629	1174.659072	698.4564629	554.365262	0.125
210396_s_at	BOLA2 ///	698.4564629	523.2511306	932.327523	554.365262	880	0.125

	LOC440354 /// LOC595101						
210609_s_at	TP53I3	698.4564629	932.327523	830.6093952	587.3295358	391.995436	0.125
210986_s_at	TPM1	698.4564629	739.9888454	1174.659072	659.2551138	349.2282314	0.125
211115_x_at	GEMIN2	698.4564629	698.4564629	391.995436	698.4564629	880	0.125
212098_at	MGAT5	698.4564629	830.6093952	932.327523	622.2539674	329.6275569	0.125
212154_at	SDC2	698.4564629	1108.730524	932.327523	466.1637615	523.2511306	0.125
212181_s_at	NUDT4 /// NUDT4P1	698.4564629	587.3295358	830.6093952	523.2511306	987.7666025	0.125
212223_at	IDS	698.4564629	783.990872	698.4564629	659.2551138	233.0818808	0.125
212563_at	BOP1	698.4564629	554.365262	622.2539674	739.9888454	987.7666025	0.125
212757_s_at	CAMK2G	698.4564629	880	587.3295358	783.990872	466.1637615	0.125
213447_at	IPW /// LOC100506948 /// SNORD107 /// SNORD115-13 /// SNORD115-26 /// SNORD115-7 /// SNORD116-28 /// SNRPN	698.4564629	932.327523	554.365262	830.6093952	587.3295358	0.125
213773_x_at	NSUN5	698.4564629	622.2539674	1174.659072	659.2551138	698.4564629	0.125
214693_x_at	LOC100506032 /// NBPF10	698.4564629	554.365262	987.7666025	466.1637615	880	0.125
214789_x_at	SRSF8	698.4564629	659.2551138	739.9888454	830.6093952	415.3046976	0.125
215735_s_at	TSC2	698.4564629	698.4564629	880	698.4564629	391.995436	0.125
217838_s_at	EVL	698.4564629	1108.730524	783.990872	587.3295358	554.365262	0.125
218062_x_at	CDC42EP4	698.4564629	932.327523	659.2551138	739.9888454	466.1637615	0.125
218135_at	ERGIC2	698.4564629	739.9888454	493.8833013	698.4564629	987.7666025	0.125
218191_s_at	LMBRD1	698.4564629	698.4564629	739.9888454	659.2551138	1244.507935	0.125
218312_s_at	ZSCAN18	698.4564629	783.990872	698.4564629	622.2539674	329.6275569	0.125
218641_at	C11orf95	698.4564629	987.7666025	830.6093952	554.365262	587.3295358	0.125
219286_s_at	RBM15	698.4564629	698.4564629	554.365262	659.2551138	987.7666025	0.125
219497_s_at	BCL11A	698.4564629	1108.730524	493.8833013	932.327523	233.0818808	0.125
220261_s_at	ZDHHC4	698.4564629	493.8833013	987.7666025	783.990872	659.2551138	0.125
221482_s_at	ARPP19	698.4564629	830.6093952	415.3046976	783.990872	587.3295358	0.125
221538_s_at	PLXNA1	698.4564629	830.6093952	783.990872	622.2539674	391.995436	0.125

221805_at	NEFL	698.4564629	739.9888454	622.2539674	1318.510228	30.86770633	0.1875
39854_r_at	PNPLA2	698.4564629	659.2551138	1108.730524	783.990872	493.8833013	0.125
40020_at	CELSR3	698.4564629	739.9888454	739.9888454	698.4564629	277.182631	0.125
200611_s_at	WDR1	739.9888454	622.2539674	1046.502261	830.6093952	659.2551138	0.125
200665_s_at	LOC100505813 /// SPARC	739.9888454	622.2539674	1046.502261	261.6255653	880	0.125
200916_at	TAGLN2	739.9888454	659.2551138	1046.502261	698.4564629	739.9888454	0.125
201146_at	NFE2L2	739.9888454	1046.502261	698.4564629	659.2551138	783.990872	0.125
201551_s_at	LAMP1	739.9888454	466.1637615	880	783.990872	659.2551138	0.125
201648_at	JAK1	739.9888454	622.2539674	783.990872	622.2539674	1479.977691	0.125
201753_s_at	ADD3	739.9888454	622.2539674	523.2511306	1318.510228	880	0.125
201955_at	CCNC	739.9888454	739.9888454	659.2551138	739.9888454	1108.730524	0.125
202092_s_at	ARL2BP	739.9888454	880	783.990872	698.4564629	440	0.125
202479_s_at	TRIB2	739.9888454	880	554.365262	987.7666025	622.2539674	0.125
202860_at	DENND4B	739.9888454	830.6093952	932.327523	659.2551138	466.1637615	0.125
202870_s_at	CDC20	739.9888454	622.2539674	698.4564629	739.9888454	1046.502261	0.125
203097_s_at	RAPGEF2	739.9888454	932.327523	523.2511306	932.327523	587.3295358	0.125
203139_at	DAPK1	739.9888454	698.4564629	659.2551138	739.9888454	1174.659072	0.125
203225_s_at	RFK	739.9888454	659.2551138	349.2282314	783.990872	739.9888454	0.125
203417_at	MFAP2	739.9888454	195.997718	1479.977691	587.3295358	880	0.1875
203524_s_at	MPST	739.9888454	739.9888454	880	783.990872	369.9944227	0.125
203764_at	DLGAP5	739.9888454	587.3295358	622.2539674	830.6093952	987.7666025	0.125
204743_at	TAGLN3	739.9888454	698.4564629	739.9888454	932.327523	73.41619198	0.125
205493_s_at	DPYSL4	739.9888454	830.6093952	783.990872	622.2539674	116.5409404	0.125
205879_x_at	RET	739.9888454	739.9888454	698.4564629	1174.659072	311.1269837	0.125
208024_s_at	DGCR6 /// DGCR6L	739.9888454	523.2511306	783.990872	659.2551138	932.327523	0.125
208754_s_at	NAP1L1	739.9888454	987.7666025	698.4564629	698.4564629	587.3295358	0.125
208809_s_at	C6orf62	739.9888454	830.6093952	659.2551138	587.3295358	1046.502261	0.125
208950_s_at	ALDH7A1	739.9888454	659.2551138	415.3046976	783.990872	932.327523	0.125
209094_at	DDAH1	739.9888454	932.327523	932.327523	164.8137785	554.365262	0.125
209361_s_at	PCBP4	739.9888454	783.990872	698.4564629	1174.659072	369.9944227	0.125
209384_at	PROSC	739.9888454	659.2551138	659.2551138	783.990872	1244.507935	0.125
209481_at	SNRK	739.9888454	830.6093952	622.2539674	783.990872	415.3046976	0.125
209524_at	HDGFRP3	739.9888454	830.6093952	698.4564629	783.990872	415.3046976	0.125
209603_at	GATA3	739.9888454	880	830.6093952	587.3295358	195.997718	0.125

209623_at	MCCC2	739.9888454	783.990872	880	698.4564629	466.1637615	0.125
209755_at	NMNAT2	739.9888454	1108.730524	587.3295358	932.327523	87.30705786	0.125
210023_s_at	PCGF1	739.9888454	1108.730524	739.9888454	698.4564629	493.8833013	0.125
210076_x_at	SERBP1	739.9888454	932.327523	493.8833013	659.2551138	830.6093952	0.125
210817_s_at	CALCOCO2	739.9888454	622.2539674	587.3295358	1046.502261	783.990872	0.125
211700_s_at	TRO	739.9888454	739.9888454	698.4564629	739.9888454	277.182631	0.125
212222_at	PSME4	739.9888454	830.6093952	622.2539674	830.6093952	440	0.125
212250_at	MTDH	739.9888454	739.9888454	659.2551138	739.9888454	1244.507935	0.125
212276_at	LPIN1	739.9888454	783.990872	830.6093952	659.2551138	369.9944227	0.125
212412_at	PDLIM5	739.9888454	349.2282314	698.4564629	783.990872	783.990872	0.125
212501_at	CEBPB	739.9888454	739.9888454	466.1637615	739.9888454	1396.912926	0.125
212614_at	ARID5B	739.9888454	369.9944227	1046.502261	830.6093952	659.2551138	0.125
213122_at	TSPYL5	739.9888454	233.0818808	830.6093952	783.990872	659.2551138	0.125
213577_at	SQLE	739.9888454	622.2539674	587.3295358	880	1567.981744	0.125
213842_x_at	NSUN5P1 /// NSUN5P2	739.9888454	739.9888454	1244.507935	659.2551138	698.4564629	0.125
214431_at	GMPS	739.9888454	659.2551138	554.365262	783.990872	1046.502261	0.125
215076_s_at	COL3A1	739.9888454	698.4564629	1567.981744	123.4708253	739.9888454	0.1875
217200_x_at	CYB561	739.9888454	440	1244.507935	1318.510228	440	0.1875
218031_s_at	FOXN3	739.9888454	880	880	554.365262	587.3295358	0.125
218726_at	HJURP	739.9888454	880	440	739.9888454	739.9888454	0.125
218781_at	SMC6	739.9888454	698.4564629	739.9888454	1479.977691	659.2551138	0.125
218829_s_at	CHD7	739.9888454	830.6093952	622.2539674	830.6093952	277.182631	0.125
219041_s_at	REPIN1	739.9888454	880	739.9888454	739.9888454	369.9944227	0.125
220085_at	HELLS	739.9888454	739.9888454	587.3295358	1046.502261	698.4564629	0.125
221965_at	MPHOSPH9	739.9888454	739.9888454	698.4564629	830.6093952	415.3046976	0.125
221989_at	RPL10 /// SNORA70	739.9888454	880	783.990872	698.4564629	415.3046976	0.125
222116_s_at	TBC1D16	739.9888454	698.4564629	739.9888454	987.7666025	466.1637615	0.125
266_s_at	CD24	739.9888454	932.327523	739.9888454	739.9888454	349.2282314	0.125
200697_at	HK1	783.990872	783.990872	783.990872	1046.502261	329.6275569	0.125
201009_s_at	TXNIP	783.990872	1174.659072	880	587.3295358	698.4564629	0.125
201195_s_at	SLC7A5	783.990872	880	659.2551138	1108.730524	622.2539674	0.125
201259_s_at	SYPL1	783.990872	783.990872	830.6093952	587.3295358	1108.730524	0.125
201330_at	RARS	783.990872	783.990872	622.2539674	1108.730524	698.4564629	0.125
201360_at	CST3	783.990872	622.2539674	932.327523	932.327523	440	0.125

201398_s_at	TRAM1	783.990872	783.990872	783.990872	783.990872	1396.912926	0.125
201566_x_at	ID2	783.990872	783.990872	987.7666025	277.182631	698.4564629	0.125
201690_s_at	TPD52	783.990872	659.2551138	440	880	932.327523	0.125
201752_s_at	ADD3	783.990872	659.2551138	587.3295358	1318.510228	932.327523	0.125
201844_s_at	RYBP	783.990872	783.990872	739.9888454	1108.730524	587.3295358	0.125
201845_s_at	RYBP	783.990872	783.990872	739.9888454	932.327523	493.8833013	0.125
201846_s_at	RYBP	783.990872	830.6093952	739.9888454	1108.730524	493.8833013	0.125
201852_x_at	COL3A1	783.990872	783.990872	1567.981744	65.40639133	739.9888454	0.1875
202033_s_at	RB1CC1	783.990872	880	659.2551138	659.2551138	1108.730524	0.125
202037_s_at	SFRP1	783.990872	1174.659072	698.4564629	783.990872	277.182631	0.125
202290_at	PDAP1	783.990872	659.2551138	1046.502261	880	698.4564629	0.125
202468_s_at	CTNNAL1	783.990872	739.9888454	554.365262	987.7666025	783.990872	0.125
202812_at	GAA	783.990872	739.9888454	830.6093952	880	349.2282314	0.125
203006_at	INPP5A	783.990872	739.9888454	739.9888454	987.7666025	523.2511306	0.125
203129_s_at	KIF5C	783.990872	1046.502261	554.365262	1046.502261	43.65352893	0.1875
203413_at	LOC100653018 /// LOC100653255 /// NELL2	783.990872	1318.510228	415.3046976	554.365262	1046.502261	0.125
203432_at	TMPO	783.990872	739.9888454	554.365262	783.990872	1046.502261	0.125
204082_at	PBX3	783.990872	698.4564629	880	830.6093952	391.995436	0.125
204086_at	PRAME	783.990872	1479.977691	1396.912926	415.3046976	440	0.1875
204247_s_at	CDK5	783.990872	830.6093952	1046.502261	698.4564629	554.365262	0.125
204334_at	KLF7	783.990872	987.7666025	698.4564629	880	523.2511306	0.125
204584_at	L1CAM	783.990872	1046.502261	659.2551138	932.327523	466.1637615	0.125
208290_s_at	EIF5	783.990872	880	466.1637615	739.9888454	932.327523	0.125
208651_x_at	CD24	783.990872	987.7666025	987.7666025	659.2551138	293.6647679	0.125
208967_s_at	AK2	783.990872	622.2539674	739.9888454	830.6093952	1046.502261	0.125
208978_at	CRIP2	783.990872	329.6275569	783.990872	698.4564629	1244.507935	0.125
209184_s_at	IRS2	783.990872	523.2511306	932.327523	659.2551138	1479.977691	0.125
210358_x_at	GATA2	783.990872	698.4564629	880	830.6093952	415.3046976	0.125
210495_x_at	FN1	783.990872	659.2551138	1046.502261	155.5634919	932.327523	0.125
210529_s_at	FAM115A /// LOC100294033	783.990872	987.7666025	783.990872	739.9888454	554.365262	0.125
210978_s_at	TAGLN2	783.990872	830.6093952	1108.730524	698.4564629	739.9888454	0.125
211421_s_at	RET	783.990872	698.4564629	830.6093952	1318.510228	220	0.125

212013_at	PXDN	783.990872	783.990872	1396.912926	698.4564629	830.6093952	0.125
212199_at	MRFAP1L1	783.990872	880	739.9888454	830.6093952	493.8833013	0.125
212327_at	LIMCH1	783.990872	659.2551138	1046.502261	880	659.2551138	0.125
212462_at	KAT6B	783.990872	739.9888454	880	783.990872	466.1637615	0.125
212508_at	MOAP1	783.990872	932.327523	698.4564629	880	493.8833013	0.125
212599_at	AUTS2	783.990872	880	1108.730524	698.4564629	622.2539674	0.125
212699_at	SCAMP5	783.990872	987.7666025	622.2539674	1046.502261	349.2282314	0.125
212731_at	ANKRD46	783.990872	698.4564629	466.1637615	932.327523	830.6093952	0.125
212774_at	ZNF238	783.990872	1174.659072	932.327523	659.2551138	293.6647679	0.125
213135_at	TIAM1	783.990872	932.327523	739.9888454	830.6093952	130.8127827	0.125
213943_at	TWIST1	783.990872	554.365262	1244.507935	246.9416506	1108.730524	0.125
214240_at	GAL	783.990872	932.327523	659.2551138	1108.730524	349.2282314	0.125
215116_s_at	DNM1	783.990872	830.6093952	880	783.990872	369.9944227	0.125
215127_s_at	RBMS1	783.990872	932.327523	523.2511306	830.6093952	739.9888454	0.125
216438_s_at	TMSB4X	783.990872	739.9888454	1108.730524	783.990872	440	0.125
217734_s_at	WDR6	783.990872	739.9888454	880	830.6093952	466.1637615	0.125
217783_s_at	YPEL5	783.990872	739.9888454	830.6093952	698.4564629	1661.21879	0.125
217989_at	HSD17B11	783.990872	932.327523	659.2551138	932.327523	554.365262	0.125
218160_at	NDUFA8	783.990872	739.9888454	659.2551138	830.6093952	1108.730524	0.125
218309_at	CAMK2N1	783.990872	293.6647679	987.7666025	1046.502261	622.2539674	0.125
218583_s_at	DCUN1D1	783.990872	698.4564629	587.3295358	830.6093952	1046.502261	0.125
218623_at	HMP19	783.990872	987.7666025	622.2539674	1318.510228	277.182631	0.125
218720_x_at	SEZ6L2	783.990872	830.6093952	739.9888454	932.327523	349.2282314	0.125
219449_s_at	TMEM70	783.990872	739.9888454	783.990872	739.9888454	2489.01587	0.25
219682_s_at	TBX3	783.990872	1174.659072	880	698.4564629	415.3046976	0.125
220651_s_at	MCM10	783.990872	880	739.9888454	1046.502261	466.1637615	0.125
221004_s_at	ITM2C	783.990872	659.2551138	932.327523	587.3295358	1108.730524	0.125
221479_s_at	BNIP3L	783.990872	783.990872	783.990872	659.2551138	1174.659072	0.125
221486_at	ENSA	783.990872	830.6093952	783.990872	698.4564629	440	0.125
222095_s_at	FAM163A	783.990872	783.990872	783.990872	783.990872	130.8127827	0.125
41856_at	UNC5B	783.990872	739.9888454	1046.502261	880	622.2539674	0.125
56197_at	C17orf61-PLSCR3 /// PLSCR3	783.990872	830.6093952	1244.507935	698.4564629	698.4564629	0.125
200729_s_at	ACTR2	830.6093952	1108.730524	622.2539674	783.990872	880	0.125
200737_at	LOC100652805 ///	830.6093952	830.6093952	659.2551138	830.6093952	1108.730524	0.125

	LOC100653302 /// PGK1						
200918_s_at	SRPR	830.6093952	622.2539674	1046.502261	880	739.9888454	0.125
201008_s_at	TXNIP	830.6093952	1108.730524	880	622.2539674	739.9888454	0.125
201215_at	PLS3	830.6093952	830.6093952	830.6093952	311.1269837	1108.730524	0.125
201309_x_at	NREP	830.6093952	1046.502261	880	783.990872	466.1637615	0.125
201562_s_at	SORD	830.6093952	739.9888454	659.2551138	932.327523	1046.502261	0.125
201590_x_at	ANXA2	830.6093952	329.6275569	1760	369.9944227	2217.461048	0.375
201599_at	OAT	830.6093952	830.6093952	880	830.6093952	220	0.125
201666_at	TIMP1	830.6093952	523.2511306	1046.502261	698.4564629	932.327523	0.125
201708_s_at	NIPSNAP1	830.6093952	783.990872	830.6093952	932.327523	523.2511306	0.125
201726_at	ELAVL1	830.6093952	659.2551138	880	698.4564629	1174.659072	0.125
201764_at	TMEM106C	830.6093952	698.4564629	830.6093952	739.9888454	1108.730524	0.125
201849_at	BNIP3	830.6093952	587.3295358	783.990872	880	1174.659072	0.125
201952_at	ALCAM	830.6093952	554.365262	1046.502261	622.2539674	1174.659072	0.125
202260_s_at	STXBP1	830.6093952	1174.659072	739.9888454	880	207.6523488	0.125
202292_x_at	LYPLA2	830.6093952	698.4564629	1108.730524	783.990872	880	0.125
202296_s_at	RER1	830.6093952	698.4564629	987.7666025	698.4564629	1108.730524	0.125
203297_s_at	JARID2	830.6093952	830.6093952	554.365262	830.6093952	987.7666025	0.125
203947_at	CSTF3	830.6093952	783.990872	587.3295358	830.6093952	1318.510228	0.125
204030_s_at	IQCJ-SCHIP1 /// SCHIP1	830.6093952	932.327523	830.6093952	783.990872	207.6523488	0.125
204730_at	RIMS3	830.6093952	1567.981744	830.6093952	783.990872	207.6523488	0.1875
204795_at	PRR3	830.6093952	880	783.990872	932.327523	523.2511306	0.125
205202_at	PCMT1	830.6093952	783.990872	523.2511306	880	932.327523	0.125
205743_at	STAC	830.6093952	783.990872	932.327523	830.6093952	440	0.125
205882_x_at	ADD3	830.6093952	739.9888454	554.365262	1318.510228	880	0.125
208212_s_at	ALK	830.6093952	1108.730524	880	739.9888454	622.2539674	0.125
208626_s_at	VAT1	830.6093952	659.2551138	987.7666025	1046.502261	587.3295358	0.125
208634_s_at	MACF1	830.6093952	987.7666025	1046.502261	622.2539674	698.4564629	0.125
208782_at	FSTL1	830.6093952	659.2551138	1174.659072	329.6275569	987.7666025	0.125
208798_x_at	GOLGA8A	830.6093952	1174.659072	830.6093952	830.6093952	698.4564629	0.125
208846_s_at	VDAC3	830.6093952	739.9888454	659.2551138	932.327523	1244.507935	0.125
208881_x_at	IDI1	830.6093952	830.6093952	587.3295358	880	1108.730524	0.125
209031_at	CADM1	830.6093952	659.2551138	987.7666025	1174.659072	220	0.125
209044_x_at	SF3B4	830.6093952	987.7666025	987.7666025	739.9888454	554.365262	0.125

209050_s_at	RALGDS	830.6093952	932.327523	987.7666025	739.9888454	466.1637615	0.125
209117_at	WBP2	830.6093952	830.6093952	932.327523	783.990872	523.2511306	0.125
209159_s_at	NDRG4	830.6093952	987.7666025	659.2551138	1046.502261	82.40688923	0.125
209257_s_at	SMC3	830.6093952	987.7666025	587.3295358	932.327523	739.9888454	0.125
209271_at	BPTF	830.6093952	1174.659072	830.6093952	783.990872	523.2511306	0.125
209392_at	ENPP2	830.6093952	1567.981744	440	1661.21879	138.5913155	0.21875
209433_s_at	PPAT	830.6093952	739.9888454	523.2511306	932.327523	1046.502261	0.125
209517_s_at	ASH2L	830.6093952	783.990872	783.990872	830.6093952	1244.507935	0.125
210517_s_at	AKAP12	830.6093952	1318.510228	698.4564629	830.6093952	830.6093952	0.125
210633_x_at	KRT10	830.6093952	932.327523	698.4564629	622.2539674	1174.659072	0.125
211985_s_at	CALM1 /// CALM2 /// CALM3	830.6093952	1046.502261	659.2551138	1046.502261	493.8833013	0.125
212012_at	PXDN	830.6093952	830.6093952	1244.507935	739.9888454	830.6093952	0.125
212056_at	KIAA0182	830.6093952	783.990872	1046.502261	830.6093952	391.995436	0.125
212178_s_at	POM121 /// POM121C	830.6093952	1108.730524	932.327523	739.9888454	622.2539674	0.125
212370_x_at	FAM21A /// FAM21B /// FAM21C	830.6093952	932.327523	880	698.4564629	554.365262	0.125
212414_s_at	GLYR1 /// SEPT6	830.6093952	880	739.9888454	1244.507935	349.2282314	0.125
212766_s_at	ISG20L2	830.6093952	783.990872	932.327523	830.6093952	493.8833013	0.125
212877_at	KLC1	830.6093952	987.7666025	659.2551138	932.327523	554.365262	0.125
212904_at	LRRC47	830.6093952	698.4564629	1046.502261	698.4564629	932.327523	0.125
213131_at	OLFM1	830.6093952	1174.659072	554.365262	349.2282314	1174.659072	0.125
213812_s_at	CAMKK2	830.6093952	659.2551138	783.990872	783.990872	1108.730524	0.125
213850_s_at	SCAF11	830.6093952	783.990872	659.2551138	880	1108.730524	0.125
215016_x_at	DST /// LOC100652766	830.6093952	830.6093952	783.990872	987.7666025	440	0.125
216442_x_at	FN1	830.6093952	659.2551138	1108.730524	184.9972114	932.327523	0.125
217923_at	PEF1	830.6093952	493.8833013	932.327523	739.9888454	987.7666025	0.125
218119_at	TIMM23	830.6093952	783.990872	880	1046.502261	622.2539674	0.125
218224_at	PNMA1	830.6093952	1046.502261	783.990872	880	523.2511306	0.125
218678_at	NES	830.6093952	830.6093952	1046.502261	830.6093952	220	0.125
218679_s_at	VPS28	830.6093952	739.9888454	880	659.2551138	1108.730524	0.125
218952_at	PCSK1N	830.6093952	783.990872	830.6093952	932.327523	523.2511306	0.125
219855_at	NUDT11	830.6093952	783.990872	622.2539674	830.6093952	1046.502261	0.125

220155_s_at	BRD9	830.6093952	523.2511306	932.327523	880	783.990872	0.125
221381_s_at	MORF4L1	830.6093952	783.990872	554.365262	880	1046.502261	0.125
221745_at	DCAF7	830.6093952	880	783.990872	880	493.8833013	0.125
221896_s_at	HIGD1A	830.6093952	783.990872	783.990872	1244.507935	880	0.125
32209_at	FAM89B	830.6093952	783.990872	1174.659072	739.9888454	880	0.125
40560_at	TBX2	830.6093952	932.327523	880	783.990872	97.998859	0.125
200607_s_at	RAD21	880	880	739.9888454	880	1244.507935	0.125
200612_s_at	AP2B1	880	932.327523	830.6093952	1174.659072	739.9888454	0.125
200672_x_at	SPTBN1	880	880	1046.502261	880	466.1637615	0.125
200734_s_at	ARF3	880	659.2551138	830.6093952	987.7666025	1046.502261	0.125
200760_s_at	ARL6IP5	880	1174.659072	739.9888454	987.7666025	659.2551138	0.125
200859_x_at	FLNA	880	830.6093952	1244.507935	698.4564629	932.327523	0.125
200919_at	PHC2	880	587.3295358	987.7666025	739.9888454	1046.502261	0.125
200931_s_at	VCL	880	830.6093952	1318.510228	830.6093952	987.7666025	0.125
200958_s_at	SDCBP	880	830.6093952	880	783.990872	1174.659072	0.125
200983_x_at	CD59	880	698.4564629	1174.659072	783.990872	880	0.125
200996_at	ACTR3	880	739.9888454	830.6093952	932.327523	1174.659072	0.125
201019_s_at	EIF1AX	880	830.6093952	830.6093952	830.6093952	1174.659072	0.125
201105_at	LGALS1	880	587.3295358	1975.533205	311.1269837	1244.507935	0.21875
201275_at	FDPS	880	932.327523	739.9888454	880	1396.912926	0.125
201318_s_at	MYL12A /// MYL12B	880	783.990872	987.7666025	739.9888454	1108.730524	0.125
201399_s_at	TRAM1	880	880	830.6093952	880	1661.21879	0.125
201651_s_at	PACSIN2	880	880	523.2511306	880	932.327523	0.125
201694_s_at	EGR1	880	880	830.6093952	932.327523	349.2282314	0.125
201725_at	CDC123	880	880	880	987.7666025	587.3295358	0.125
201738_at	EIF1B	880	1174.659072	783.990872	932.327523	554.365262	0.125
201874_at	MPZL1	880	987.7666025	1046.502261	830.6093952	659.2551138	0.125
202345_s_at	FABP5	880	146.832384	698.4564629	1108.730524	1318.510228	0.1875
202465_at	PCOLCE	880	880	1864.655046	880	466.1637615	0.1875
202548_s_at	ARHGEF7	880	880	987.7666025	880	554.365262	0.125
203259_s_at	HDDC2	880	880	830.6093952	830.6093952	1318.510228	0.125
203318_s_at	ZNF148	880	659.2551138	783.990872	932.327523	1108.730524	0.125
203345_s_at	MTF2	880	830.6093952	587.3295358	987.7666025	932.327523	0.125
203423_at	RBP1	880	82.40688923	1396.912926	1864.655046	523.2511306	0.25
203534_at	LSM1	880	783.990872	783.990872	987.7666025	1174.659072	0.125

203748_x_at	RBMS1	880	1108.730524	739.9888454	1046.502261	739.9888454	0.125
203755_at	BUB1B	880	830.6093952	739.9888454	880	1174.659072	0.125
203917_at	CXADR	880	1396.912926	622.2539674	880	830.6093952	0.125
203999_at	SYT1	880	1661.21879	880	880	554.365262	0.125
204069_at	MEIS1	880	1174.659072	659.2551138	554.365262	1108.730524	0.125
206061_s_at	DICER1	880	987.7666025	587.3295358	932.327523	830.6093952	0.125
206102_at	GINS1	880	830.6093952	587.3295358	932.327523	987.7666025	0.125
207260_at	FEV	880	987.7666025	880	880	77.78174593	0.125
207480_s_at	MEIS2	880	1108.730524	1479.977691	659.2551138	369.9944227	0.1875
207831_x_at	DHPS	880	698.4564629	932.327523	1108.730524	783.990872	0.125
207949_s_at	ICA1	880	880	880	880	103.8261744	0.125
208659_at	CLIC1	880	880	830.6093952	880	1318.510228	0.125
208709_s_at	NRD1	880	830.6093952	880	880	1244.507935	0.125
208753_s_at	NAP1L1	880	1174.659072	830.6093952	880	739.9888454	0.125
208765_s_at	HNRNPR	880	932.327523	783.990872	783.990872	1174.659072	0.125
208864_s_at	TXN	880	932.327523	698.4564629	783.990872	1396.912926	0.125
208972_s_at	ATP5G1	880	659.2551138	698.4564629	987.7666025	1108.730524	0.125
209071_s_at	RGS5	880	1760	2349.318143	415.3046976	261.6255653	0.4375
209289_at	NFIB	880	987.7666025	1108.730524	783.990872	739.9888454	0.125
209560_s_at	DLK1	880	659.2551138	1108.730524	3135.963488	195.997718	0.875
209569_x_at	D4S234E	880	880	830.6093952	1046.502261	622.2539674	0.125
209608_s_at	ACAT2	880	830.6093952	659.2551138	932.327523	1244.507935	0.125
210427_x_at	ANXA2	880	415.3046976	1864.655046	369.9944227	2349.318143	0.4375
210987_x_at	TPM1	880	932.327523	1567.981744	830.6093952	554.365262	0.125
211729_x_at	BLVRA	880	739.9888454	1174.659072	987.7666025	659.2551138	0.125
211772_x_at	CHRNA3	880	1046.502261	830.6093952	880	184.9972114	0.125
212092_at	PEG10	880	932.327523	783.990872	1479.977691	830.6093952	0.125
212094_at	PEG10	880	880	739.9888454	1318.510228	830.6093952	0.125
212461_at	AZIN1	880	1174.659072	830.6093952	880	739.9888454	0.125
212635_at	TNPO1	880	1174.659072	830.6093952	880	783.990872	0.125
212764_at	ZEB1	880	987.7666025	783.990872	932.327523	277.182631	0.125
213503_x_at	ANXA2	880	415.3046976	1661.21879	277.182631	2217.461048	0.375
213939_s_at	RUFY3	880	1108.730524	739.9888454	987.7666025	440	0.125
214023_x_at	TUBB2B	880	1046.502261	783.990872	987.7666025	349.2282314	0.125
214505_s_at	FHL1	880	830.6093952	1396.912926	880	554.365262	0.125
216607_s_at	CYP51A1 ///	880	932.327523	587.3295358	830.6093952	1174.659072	0.125

	LRRD1						
218773_s_at	MSRB2	880	932.327523	1174.659072	830.6093952	493.8833013	0.125
219097_x_at	C19orf42	880	1046.502261	698.4564629	1108.730524	739.9888454	0.125
219170_at	FSD1	880	783.990872	932.327523	987.7666025	138.5913155	0.125
219639_x_at	PARP6	880	1046.502261	830.6093952	932.327523	622.2539674	0.125
221916_at	NEFL	880	987.7666025	739.9888454	1661.21879	97.998859	0.21875
222077_s_at	RACGAP1	880	783.990872	659.2551138	1046.502261	1108.730524	0.125
41386_i_at	KDM6B	880	987.7666025	987.7666025	783.990872	554.365262	0.125
44654_at	G6PC3	880	932.327523	830.6093952	987.7666025	587.3295358	0.125
200603_at	PRKAR1A	932.327523	1174.659072	783.990872	1046.502261	830.6093952	0.125
200653_s_at	CALM1 /// CALM2 /// CALM3	932.327523	987.7666025	880	1046.502261	622.2539674	0.125
200699_at	KDELR2	932.327523	659.2551138	880	1108.730524	987.7666025	0.125
200761_s_at	ARL6IP5	932.327523	1174.659072	659.2551138	1108.730524	739.9888454	0.125
200771_at	LAMC1	932.327523	783.990872	1174.659072	1108.730524	739.9888454	0.125
200782_at	ANXA5	932.327523	987.7666025	830.6093952	554.365262	1396.912926	0.125
200862_at	DHCR24	932.327523	830.6093952	659.2551138	1046.502261	1108.730524	0.125
200891_s_at	SSR1	932.327523	880	783.990872	1046.502261	1174.659072	0.125
200976_s_at	TAX1BP1	932.327523	739.9888454	1108.730524	783.990872	1046.502261	0.125
200998_s_at	CKAP4	932.327523	932.327523	932.327523	932.327523	1396.912926	0.125
201029_s_at	CD99	932.327523	293.6647679	880	987.7666025	1567.981744	0.1875
201064_s_at	PABPC4	932.327523	1174.659072	1046.502261	783.990872	739.9888454	0.125
201118_at	PGD	932.327523	783.990872	987.7666025	880	1396.912926	0.125
201120_s_at	PGRMC1	932.327523	987.7666025	622.2539674	880	987.7666025	0.125
201174_s_at	TERF2IP	932.327523	932.327523	880	932.327523	391.995436	0.125
201313_at	ENO2	932.327523	1244.507935	783.990872	1046.502261	622.2539674	0.125
201464_x_at	JUN	932.327523	932.327523	493.8833013	880	987.7666025	0.125
201642_at	IFNGR2	932.327523	783.990872	1174.659072	987.7666025	830.6093952	0.125
202218_s_at	FADS2	932.327523	783.990872	1046.502261	830.6093952	1174.659072	0.125
202554_s_at	GSTM3	932.327523	830.6093952	830.6093952	1046.502261	1479.977691	0.125
203229_s_at	CLK2	932.327523	1046.502261	1046.502261	783.990872	622.2539674	0.125
203300_x_at	AP1S2	932.327523	1174.659072	932.327523	880	698.4564629	0.125
203773_x_at	BLVRA	932.327523	698.4564629	1244.507935	1108.730524	783.990872	0.125
203825_at	BRD3	932.327523	880	987.7666025	1318.510228	739.9888454	0.125
204235_s_at	GULP1	932.327523	1318.510228	1046.502261	783.990872	329.6275569	0.125
204260_at	CHGB	932.327523	1318.510228	587.3295358	1760	92.49860568	0.21875

204710_s_at	WIPI2	932.327523	622.2539674	1046.502261	880	932.327523	0.125
204825_at	MELK	932.327523	880	698.4564629	987.7666025	1108.730524	0.125
207719_x_at	CEP170	932.327523	1108.730524	1046.502261	830.6093952	739.9888454	0.125
208683_at	CAPN2	932.327523	830.6093952	1479.977691	440	987.7666025	0.125
208694_at	PRKDC	932.327523	830.6093952	783.990872	987.7666025	1174.659072	0.125
208703_s_at	APLP2	932.327523	659.2551138	1108.730524	880	880	0.125
209070_s_at	RGS5	932.327523	1975.533205	2637.020455	440	110	0.75
209197_at	SYT11	932.327523	1318.510228	783.990872	1108.730524	233.0818808	0.1875
209372_x_at	TUBB2A /// TUBB2B	932.327523	880	932.327523	1244.507935	554.365262	0.125
209678_s_at	PRKCI	932.327523	830.6093952	783.990872	987.7666025	1174.659072	0.125
210036_s_at	KCNH2	932.327523	987.7666025	1108.730524	880	466.1637615	0.125
210766_s_at	CSE1L	932.327523	830.6093952	783.990872	987.7666025	1174.659072	0.125
210844_x_at	CTNNA1	932.327523	739.9888454	1046.502261	830.6093952	1108.730524	0.125
211049_at	TLX2	932.327523	1244.507935	932.327523	932.327523	65.40639133	0.1875
212010_s_at	CDV3	932.327523	987.7666025	622.2539674	880	1046.502261	0.125
212036_s_at	PNN	932.327523	1174.659072	554.365262	987.7666025	830.6093952	0.125
212037_at	PNN	932.327523	1174.659072	659.2551138	932.327523	830.6093952	0.125
212041_at	ATP6V0D1	932.327523	932.327523	987.7666025	932.327523	523.2511306	0.125
212100_s_at	POLDIP3	932.327523	932.327523	587.3295358	987.7666025	932.327523	0.125
212163_at	KIDINS220	932.327523	1174.659072	987.7666025	830.6093952	659.2551138	0.125
212166_at	XPO7	932.327523	739.9888454	880	932.327523	1174.659072	0.125
212254_s_at	DST /// LOC100652766	932.327523	932.327523	932.327523	1174.659072	554.365262	0.125
212622_at	TMEM41B	932.327523	1046.502261	698.4564629	830.6093952	1046.502261	0.125
212746_s_at	CEP170	932.327523	1046.502261	987.7666025	830.6093952	622.2539674	0.125
212771_at	FAM171A1	932.327523	987.7666025	880	880	415.3046976	0.125
212843_at	NCAM1	932.327523	880	987.7666025	932.327523	155.5634919	0.125
213029_at	NFIB	932.327523	1108.730524	1396.912926	739.9888454	783.990872	0.125
213587_s_at	ATP6V0E2	932.327523	987.7666025	932.327523	932.327523	587.3295358	0.125
213627_at	MAGED2	932.327523	932.327523	880	1244.507935	622.2539674	0.125
213702_x_at	ASAH1	932.327523	1174.659072	932.327523	880	739.9888454	0.125
215111_s_at	TSC22D1	932.327523	1046.502261	739.9888454	1244.507935	783.990872	0.125
215836_s_at	PCDHGA1 /// PCDHGA10 /// PCDHGA11 ///	932.327523	1046.502261	1108.730524	698.4564629	830.6093952	0.125

	PCDHGA12 /// PCDHGA2 /// PCDHGA3 /// PCDHGA4 /// PCDHGA5 /// PCDHGA6 /// PCDHGA7 /// PCDHGA8 /// PCDHGA9 /// PCDHGB1 /// PCDHGB2 /// PCDHGB3 /// PCDHGB4 /// PCDHGB5 /// PCDHGB6 /// PCDHGB7 /// PCDHGC3 /// PCDHGC4 /// PCDHGC5						
216194_s_at	TBCB	932.327523	987.7666025	830.6093952	1396.912926	698.4564629	0.125
216693_x_at	HDGFRP3	932.327523	1108.730524	830.6093952	987.7666025	369.9944227	0.125
217814_at	CCDC47	932.327523	1108.730524	659.2551138	932.327523	880	0.125
218229_s_at	POGK	932.327523	1108.730524	1108.730524	698.4564629	739.9888454	0.125
218580_x_at	AURKAIP1	932.327523	587.3295358	987.7666025	830.6093952	987.7666025	0.125
220942_x_at	FAM162A	932.327523	830.6093952	830.6093952	987.7666025	1864.655046	0.1875
221853_s_at	NOMO1 /// NOMO2 /// NOMO3	932.327523	932.327523	1108.730524	987.7666025	698.4564629	0.125
36129_at	SGSM2	932.327523	987.7666025	1108.730524	830.6093952	698.4564629	0.125
200000_s_at	PRPF8	987.7666025	1174.659072	1108.730524	880	739.9888454	0.125
200083_at	USP22	987.7666025	1108.730524	1174.659072	440	830.6093952	0.125
200965_s_at	ABLIM1	987.7666025	987.7666025	1108.730524	932.327523	698.4564629	0.125
200999_s_at	CKAP4	987.7666025	987.7666025	932.327523	987.7666025	1479.977691	0.125
201034_at	ADD3	987.7666025	880	659.2551138	1396.912926	1046.502261	0.125
201066_at	CYC1	987.7666025	783.990872	880	1046.502261	1479.977691	0.125
201069_at	MMP2	987.7666025	932.327523	1244.507935	1046.502261	261.6255653	0.125

201291_s_at	TOP2A	987.7666025	783.990872	698.4564629	1174.659072	1479.977691	0.125
201425_at	ALDH2	987.7666025	932.327523	987.7666025	1174.659072	391.995436	0.125
201445_at	CNN3	987.7666025	1108.730524	830.6093952	1046.502261	698.4564629	0.125
201635_s_at	FXR1	987.7666025	880	880	987.7666025	1396.912926	0.125
201760_s_at	WSB2	987.7666025	932.327523	1046.502261	1108.730524	622.2539674	0.125
201841_s_at	HSPB1	987.7666025	830.6093952	1108.730524	783.990872	1244.507935	0.125
201956_s_at	GNPAT	987.7666025	1244.507935	987.7666025	880	880	0.125
202001_s_at	NDUFA6	987.7666025	783.990872	587.3295358	1174.659072	1108.730524	0.125
202220_at	KIAA0907	987.7666025	1174.659072	1244.507935	783.990872	830.6093952	0.125
202313_at	PPP2R2A	987.7666025	880	880	987.7666025	1479.977691	0.125
202395_at	LOC100507699 /// NSF	987.7666025	1108.730524	698.4564629	987.7666025	932.327523	0.125
202520_s_at	MLH1	987.7666025	1046.502261	987.7666025	1108.730524	659.2551138	0.125
202560_s_at	CHTOP	987.7666025	1244.507935	987.7666025	987.7666025	830.6093952	0.125
202824_s_at	TCEB1	987.7666025	783.990872	880	987.7666025	1479.977691	0.125
203485_at	RTN1	987.7666025	1046.502261	932.327523	987.7666025	261.6255653	0.125
203625_x_at	SKP2	987.7666025	880	739.9888454	1174.659072	1046.502261	0.125
203680_at	PRKAR2B	987.7666025	1174.659072	987.7666025	932.327523	523.2511306	0.125
203931_s_at	MRPL12	987.7666025	783.990872	987.7666025	1244.507935	932.327523	0.125
204141_at	TUBB2A	987.7666025	1244.507935	739.9888454	1479.977691	622.2539674	0.125
204465_s_at	INA	987.7666025	1174.659072	830.6093952	1244.507935	329.6275569	0.125
205436_s_at	H2AFX	987.7666025	783.990872	1318.510228	1108.730524	830.6093952	0.125
205812_s_at	TMED9	987.7666025	739.9888454	830.6093952	1108.730524	1108.730524	0.125
207809_s_at	ATP6AP1	987.7666025	1108.730524	987.7666025	932.327523	659.2551138	0.125
207966_s_at	GLG1	987.7666025	783.990872	987.7666025	932.327523	1318.510228	0.125
208853_s_at	CANX	987.7666025	1046.502261	698.4564629	932.327523	1244.507935	0.125
208937_s_at	ID1	987.7666025	880	1567.981744	987.7666025	932.327523	0.125
209526_s_at	HDGFRP3	987.7666025	1174.659072	932.327523	1046.502261	493.8833013	0.125
209987_s_at	ASCL1	987.7666025	1567.981744	1396.912926	659.2551138	82.40688923	0.21875
210428_s_at	HGS	987.7666025	932.327523	1479.977691	987.7666025	880	0.125
211727_s_at	COX11	987.7666025	1046.502261	622.2539674	987.7666025	987.7666025	0.125
211730_s_at	POLR2L	987.7666025	739.9888454	1108.730524	830.6093952	1396.912926	0.125
211929_at	HNRNPA3	987.7666025	1244.507935	880	1108.730524	880	0.125
211936_at	HSPA5	987.7666025	932.327523	932.327523	1046.502261	1318.510228	0.125
211951_at	NOLC1	987.7666025	1108.730524	880	1244.507935	880	0.125
211984_at	CALM1 /// CALM2	987.7666025	1046.502261	880	1174.659072	523.2511306	0.125

	/// CALM3						
212057_at	KIAA0182	987.7666025	932.327523	987.7666025	1046.502261	493.8833013	0.125
212197_x_at	MPRIP	987.7666025	1046.502261	1046.502261	932.327523	659.2551138	0.125
212552_at	HPCAL1	987.7666025	783.990872	1244.507935	2217.461048	554.365262	0.21875
213408_s_at	PI4KA /// PI4KAP1 /// PI4KAP2	987.7666025	932.327523	1108.730524	987.7666025	622.2539674	0.125
213787_s_at	EBP	987.7666025	932.327523	659.2551138	987.7666025	1108.730524	0.125
213846_at	COX7C	987.7666025	880	783.990872	1108.730524	1174.659072	0.125
214268_s_at	MTMR4	987.7666025	1046.502261	932.327523	987.7666025	622.2539674	0.125
214451_at	TFAP2B	987.7666025	783.990872	1108.730524	880	1318.510228	0.125
214512_s_at	SUB1	987.7666025	932.327523	739.9888454	1318.510228	1046.502261	0.125
214771_x_at	MPRIP	987.7666025	1108.730524	987.7666025	932.327523	659.2551138	0.125
215000_s_at	FEZ2	987.7666025	830.6093952	1108.730524	698.4564629	1174.659072	0.125
216396_s_at	EI24	987.7666025	698.4564629	987.7666025	987.7666025	1174.659072	0.125
217755_at	HN1	987.7666025	1244.507935	932.327523	987.7666025	783.990872	0.125
217845_x_at	HIGD1A	987.7666025	880	739.9888454	1318.510228	987.7666025	0.125
217897_at	FXYD6	987.7666025	1046.502261	1046.502261	932.327523	311.1269837	0.125
218027_at	MRPL15	987.7666025	932.327523	783.990872	987.7666025	1396.912926	0.125
218477_at	TMEM14A	987.7666025	987.7666025	987.7666025	1396.912926	830.6093952	0.125
219148_at	PBK	987.7666025	880	622.2539674	1046.502261	1108.730524	0.125
220948_s_at	ATP1A1	987.7666025	987.7666025	932.327523	932.327523	1760	0.125
200655_s_at	CALM1 /// CALM2 /// CALM3	1046.502261	1108.730524	987.7666025	1174.659072	698.4564629	0.125
200769_s_at	MAT2A	1046.502261	1244.507935	932.327523	830.6093952	1108.730524	0.125
200802_at	SARS	1046.502261	1108.730524	880	1396.912926	932.327523	0.125
200923_at	LGALS3BP	1046.502261	1108.730524	1318.510228	932.327523	783.990872	0.125
200941_at	HSBP1	1046.502261	987.7666025	1046.502261	1046.502261	587.3295358	0.125
201010_s_at	TXNIP	1046.502261	1318.510228	1174.659072	622.2539674	830.6093952	0.125
201089_at	ATP6V1B2	1046.502261	987.7666025	987.7666025	1396.912926	1046.502261	0.125
201307_at	41528	1046.502261	1244.507935	830.6093952	1174.659072	830.6093952	0.125
201591_s_at	NISCH	1046.502261	1174.659072	1046.502261	932.327523	554.365262	0.125
201630_s_at	ACP1	1046.502261	1396.912926	987.7666025	1046.502261	880	0.125
201857_at	ZFR	1046.502261	987.7666025	830.6093952	1046.502261	1244.507935	0.125
202084_s_at	SEC14L1	1046.502261	987.7666025	1108.730524	1046.502261	698.4564629	0.125
202089_s_at	SLC39A6	1046.502261	1318.510228	932.327523	1174.659072	698.4564629	0.125

203156_at	AKAP11	1046.502261	1174.659072	932.327523	1108.730524	698.4564629	0.125
204035_at	SCG2	1046.502261	1479.977691	698.4564629	1479.977691	220	0.1875
204338_s_at	RGS4	1046.502261	1174.659072	1567.981744	880	174.6141157	0.1875
205412_at	ACAT1	1046.502261	659.2551138	1046.502261	987.7666025	1174.659072	0.125
208647_at	FDFT1	1046.502261	1046.502261	932.327523	1046.502261	1864.655046	0.125
208706_s_at	EIF5	1046.502261	1046.502261	659.2551138	987.7666025	1174.659072	0.125
208708_x_at	EIF5	1046.502261	1046.502261	554.365262	932.327523	1108.730524	0.125
208742_s_at	SAP18	1046.502261	1046.502261	987.7666025	1046.502261	1396.912926	0.125
208939_at	SEPHS1	1046.502261	1046.502261	987.7666025	1108.730524	587.3295358	0.125
208963_x_at	FADS1 /// MIR1908	1046.502261	1108.730524	739.9888454	987.7666025	1318.510228	0.125
208968_s_at	CIAPIN1	1046.502261	987.7666025	1108.730524	1244.507935	783.990872	0.125
209035_at	MDK	1046.502261	1244.507935	1174.659072	932.327523	554.365262	0.125
209290_s_at	NFIB	1046.502261	1174.659072	1479.977691	932.327523	830.6093952	0.125
209507_at	RPA3	1046.502261	698.4564629	1046.502261	1174.659072	932.327523	0.125
209836_x_at	BOLA2 /// BOLA2B	1046.502261	698.4564629	1046.502261	1174.659072	987.7666025	0.125
209899_s_at	PUF60	1046.502261	932.327523	1046.502261	987.7666025	1318.510228	0.125
209988_s_at	ASCL1	1046.502261	1975.533205	1479.977691	698.4564629	184.9972114	0.25
210024_s_at	UBE2E3	1046.502261	1318.510228	932.327523	739.9888454	1046.502261	0.125
210543_s_at	PRKDC	1046.502261	987.7666025	880	1108.730524	1396.912926	0.125
211452_x_at	LRRFIP1	1046.502261	1046.502261	932.327523	987.7666025	1318.510228	0.125
211587_x_at	CHRNA3	1046.502261	1108.730524	932.327523	1046.502261	130.8127827	0.125
211986_at	AHNAK	1046.502261	987.7666025	987.7666025	233.0818808	1046.502261	0.125
212033_at	RBM25	1046.502261	1244.507935	739.9888454	1046.502261	987.7666025	0.125
212048_s_at	YARS	1046.502261	622.2539674	987.7666025	1108.730524	1046.502261	0.125
212124_at	ZMIZ1	1046.502261	1396.912926	1174.659072	932.327523	440	0.125
212221_x_at	IDS	1046.502261	1108.730524	987.7666025	1174.659072	311.1269837	0.125
212330_at	TFDP1	1046.502261	987.7666025	932.327523	1046.502261	1318.510228	0.125
212607_at	AKT3	1046.502261	1396.912926	932.327523	1046.502261	277.182631	0.125
214352_s_at	KRAS	1046.502261	987.7666025	932.327523	1174.659072	1318.510228	0.125
214730_s_at	GLG1	1046.502261	880	1046.502261	1046.502261	1567.981744	0.125
216032_s_at	ERGIC3	1046.502261	987.7666025	1318.510228	932.327523	1046.502261	0.125
217122_s_at	SLC35E2 /// SLC35E2B	1046.502261	987.7666025	1396.912926	783.990872	1046.502261	0.125
217736_s_at	EIF2AK1	1046.502261	830.6093952	1396.912926	1174.659072	932.327523	0.125

217780_at	WDR83OS	1046.502261	987.7666025	1318.510228	1046.502261	830.6093952	0.125
217811_at	SELT	1046.502261	987.7666025	783.990872	1108.730524	1244.507935	0.125
217869_at	HSD17B12	1046.502261	184.9972114	783.990872	1318.510228	1567.981744	0.1875
217919_s_at	MRPL42	1046.502261	932.327523	880	1174.659072	1479.977691	0.125
218103_at	FTSJ3	1046.502261	1046.502261	1046.502261	1108.730524	587.3295358	0.125
218421_at	CERK	1046.502261	1567.981744	932.327523	1174.659072	783.990872	0.125
219392_x_at	PRR11	1046.502261	1108.730524	739.9888454	1244.507935	987.7666025	0.125
221516_s_at	SMCR7L	1046.502261	987.7666025	659.2551138	1046.502261	1046.502261	0.125
35201_at	HNRNPL	1046.502261	1318.510228	1174.659072	932.327523	932.327523	0.125
200090_at	FNTA	1108.730524	1046.502261	987.7666025	1108.730524	1661.21879	0.125
200722_s_at	CAPRIN1	1108.730524	1108.730524	1108.730524	987.7666025	1396.912926	0.125
200825_s_at	HYOU1	1108.730524	783.990872	1174.659072	1108.730524	1108.730524	0.125
201014_s_at	PAICS	1108.730524	1046.502261	880	1108.730524	1479.977691	0.125
201096_s_at	ARF4	1108.730524	1174.659072	1046.502261	1108.730524	783.990872	0.125
201242_s_at	ATP1B1	1108.730524	1174.659072	1174.659072	987.7666025	207.6523488	0.125
201397_at	PHGDH	1108.730524	880	987.7666025	1318.510228	1108.730524	0.125
201433_s_at	PTDSS1	1108.730524	1108.730524	932.327523	1108.730524	1661.21879	0.125
201533_at	CTNNB1	1108.730524	1479.977691	932.327523	1244.507935	987.7666025	0.125
201653_at	CNIH	1108.730524	987.7666025	783.990872	1318.510228	1318.510228	0.125
201670_s_at	MARCKS	1108.730524	1174.659072	1174.659072	987.7666025	587.3295358	0.125
201682_at	PMPCB	1108.730524	1318.510228	1244.507935	987.7666025	783.990872	0.125
201804_x_at	TBCB	1108.730524	1046.502261	1046.502261	1567.981744	880	0.125
202413_s_at	USP1	1108.730524	1046.502261	830.6093952	1108.730524	1396.912926	0.125
202854_at	HPRT1	1108.730524	1046.502261	783.990872	1244.507935	1244.507935	0.125
203033_x_at	FH	1108.730524	987.7666025	932.327523	1244.507935	1661.21879	0.125
203371_s_at	NDUFB3	1108.730524	1174.659072	830.6093952	1046.502261	1244.507935	0.125
203478_at	NDUFC1	1108.730524	1046.502261	987.7666025	1046.502261	1479.977691	0.125
203662_s_at	TMOD1	1108.730524	1108.730524	1046.502261	1108.730524	116.5409404	0.1875
203781_at	MRPL33	1108.730524	1108.730524	1046.502261	1318.510228	622.2539674	0.125
204131_s_at	FOXO3	1108.730524	1318.510228	1108.730524	1108.730524	880	0.125
204285_s_at	PMAIP1	1108.730524	1244.507935	739.9888454	1046.502261	1046.502261	0.125
204295_at	SURF1	1108.730524	1046.502261	1396.912926	1108.730524	698.4564629	0.125
204339_s_at	RGS4	1108.730524	1174.659072	1661.21879	932.327523	130.8127827	0.1875
204540_at	EEF1A2	1108.730524	1318.510228	987.7666025	1567.981744	195.997718	0.1875
208616_s_at	PTP4A2	1108.730524	932.327523	1108.730524	1046.502261	1396.912926	0.125
208836_at	ATP1B3	1108.730524	1046.502261	1046.502261	1174.659072	1479.977691	0.125

208886_at	H1F0	1108.730524	1864.655046	987.7666025	1108.730524	1046.502261	0.125
208962_s_at	FADS1 /// MIR1908	1108.730524	1174.659072	739.9888454	987.7666025	1318.510228	0.125
209043_at	PAPSS1	1108.730524	987.7666025	1108.730524	1046.502261	1479.977691	0.125
209064_x_at	PAIP1	1108.730524	1046.502261	1244.507935	1174.659072	830.6093952	0.125
209079_x_at	PCDHGA1 /// PCDHGA10 /// PCDHGA11 /// PCDHGA12 /// PCDHGA2 /// PCDHGA3 /// PCDHGA4 /// PCDHGA5 /// PCDHGA6 /// PCDHGA7 /// PCDHGA8 /// PCDHGA9 /// PCDHGB1 /// PCDHGB2 /// PCDHGB3 /// PCDHGB4 /// PCDHGB5 /// PCDHGB6 /// PCDHGB7 /// PCDHGC3 /// PCDHGC4 /// PCDHGC5	1108.730524	1318.510228	1396.912926	830.6093952	880	0.125
209123_at	QDPR	1108.730524	1174.659072	987.7666025	1244.507935	554.365262	0.125
209210_s_at	FERMT2	1108.730524	1108.730524	1396.912926	1046.502261	739.9888454	0.125
209841_s_at	LRRN3	1108.730524	1864.655046	1396.912926	880	220	0.21875
210222_s_at	RTN1	1108.730524	1244.507935	987.7666025	1174.659072	246.9416506	0.125
210950_s_at	FDFT1	1108.730524	1046.502261	1046.502261	1108.730524	2217.461048	0.1875
211759_x_at	TBCB	1108.730524	1108.730524	1046.502261	1567.981744	880	0.125
211783_s_at	MTA1	1108.730524	1244.507935	1174.659072	1046.502261	830.6093952	0.125
211931_s_at	HNRNPA3 /// HNRNPA3P1	1108.730524	1244.507935	783.990872	1108.730524	1108.730524	0.125

211955_at	IPO5	1108.730524	1046.502261	932.327523	1108.730524	1479.977691	0.125
212068_s_at	PRRC2B	1108.730524	1174.659072	1046.502261	1046.502261	739.9888454	0.125
212141_at	MCM4	1108.730524	1046.502261	783.990872	1174.659072	1479.977691	0.125
212251_at	MTDH	1108.730524	1108.730524	932.327523	1174.659072	1760	0.125
213229_at	DICER1	1108.730524	1174.659072	739.9888454	1108.730524	987.7666025	0.125
213360_s_at	POM121 /// POM121C	1108.730524	1244.507935	1244.507935	987.7666025	880	0.125
214042_s_at	RPL22	1108.730524	1108.730524	1046.502261	880	1318.510228	0.125
214170_x_at	FH	1108.730524	1174.659072	739.9888454	1046.502261	1661.21879	0.125
216241_s_at	TCEA1	1108.730524	1046.502261	987.7666025	1174.659072	1567.981744	0.125
218117_at	RBX1	1108.730524	1046.502261	659.2551138	1244.507935	1244.507935	0.125
218391_at	SNF8	1108.730524	1479.977691	1046.502261	1174.659072	1046.502261	0.125
222036_s_at	MCM4	1108.730524	1108.730524	783.990872	1108.730524	1396.912926	0.125
222212_s_at	CERS2	1108.730524	1174.659072	1479.977691	987.7666025	1046.502261	0.125
200078_s_at	ATP6V0B	1174.659072	1046.502261	1046.502261	1479.977691	1318.510228	0.125
200604_s_at	PRKAR1A	1174.659072	1108.730524	1174.659072	1244.507935	783.990872	0.125
200708_at	GOT2	1174.659072	1396.912926	987.7666025	1244.507935	880	0.125
200736_s_at	GPX1	1174.659072	1046.502261	1318.510228	1244.507935	783.990872	0.125
200749_at	RAN	1174.659072	1174.659072	830.6093952	1174.659072	1174.659072	0.125
200768_s_at	MAT2A	1174.659072	1479.977691	1046.502261	1046.502261	1174.659072	0.125
201054_at	HNRNPA0	1174.659072	1479.977691	1046.502261	1244.507935	1108.730524	0.125
201104_x_at	LOC100506032 /// NBPF10 /// NBPF11 /// NBPF12 /// NBPF14 /// NBPF15 /// NBPF16 /// NBPF24 /// NBPF7 /// NBPF8 /// NBPF9	1174.659072	1046.502261	1479.977691	880	1244.507935	0.125
201172_x_at	ATP6V0E1	1174.659072	1174.659072	1108.730524	1046.502261	1479.977691	0.125
201226_at	NDUFB8	1174.659072	1244.507935	1108.730524	1567.981744	987.7666025	0.125
201243_s_at	ATP1B1	1174.659072	1108.730524	1244.507935	1174.659072	311.1269837	0.125
201375_s_at	PPP2CB	1174.659072	1108.730524	880	1108.730524	1479.977691	0.125
201477_s_at	RRM1	1174.659072	1108.730524	698.4564629	1174.659072	1318.510228	0.125

201540_at	FHL1	1174.659072	1174.659072	1567.981744	1174.659072	830.6093952	0.125
201563_at	SORD	1174.659072	1046.502261	932.327523	1244.507935	1318.510228	0.125
201676_x_at	PSMA1	1174.659072	1174.659072	1046.502261	932.327523	1396.912926	0.125
201828_x_at	FAM127A	1174.659072	1174.659072	1318.510228	1174.659072	554.365262	0.125
201864_at	GDI1	1174.659072	1174.659072	1244.507935	1244.507935	622.2539674	0.125
201973_s_at	CCZ1 /// CCZ1B	1174.659072	987.7666025	1479.977691	1174.659072	1108.730524	0.125
202095_s_at	BIRC5	1174.659072	1108.730524	830.6093952	1244.507935	1174.659072	0.125
202517_at	CRMP1	1174.659072	1318.510228	987.7666025	1244.507935	277.182631	0.1875
203000_at	STMN2	1174.659072	880	1864.655046	1479.977691	138.5913155	0.21875
203001_s_at	STMN2	1174.659072	830.6093952	1975.533205	1567.981744	51.9130872	0.25
203661_s_at	TMOD1	1174.659072	1318.510228	1108.730524	1244.507935	246.9416506	0.1875
204913_s_at	SOX11	1174.659072	1864.655046	1479.977691	622.2539674	880	0.1875
205133_s_at	HSPE1	1174.659072	1244.507935	880	1046.502261	1244.507935	0.125
206051_at	ELAVL4	1174.659072	1479.977691	1318.510228	1046.502261	293.6647679	0.1875
206809_s_at	HNRNPA3 /// HNRNPA3P1	1174.659072	1479.977691	783.990872	1174.659072	1174.659072	0.125
208660_at	CS	1174.659072	1318.510228	1108.730524	1108.730524	880	0.125
208661_s_at	TTC3 /// TTC3P1	1174.659072	1244.507935	880	1244.507935	1046.502261	0.125
208845_at	VDAC3	1174.659072	1046.502261	1046.502261	1244.507935	1479.977691	0.125
208964_s_at	FADS1 /// MIR1908	1174.659072	1244.507935	880	1108.730524	1396.912926	0.125
209185_s_at	IRS2	1174.659072	523.2511306	1318.510228	987.7666025	1864.655046	0.1875
209218_at	SQLE	1174.659072	1174.659072	880	1174.659072	1975.533205	0.125
209389_x_at	DBI	1174.659072	1108.730524	987.7666025	1244.507935	1567.981744	0.125
209537_at	EXTL2	1174.659072	1108.730524	1567.981744	1244.507935	987.7666025	0.125
209932_s_at	DUT	1174.659072	1174.659072	1046.502261	1479.977691	1046.502261	0.125
210425_x_at	GOLGA8A /// GOLGA8B /// LOC100508892	1174.659072	1318.510228	1318.510228	1046.502261	830.6093952	0.125
211066_x_at	PCDHGA1 /// PCDHGA10 /// PCDHGA11 /// PCDHGA12 /// PCDHGA2 /// PCDHGA3 /// PCDHGA4 ///	1174.659072	1396.912926	1318.510228	830.6093952	987.7666025	0.125

	PCDHGA5 /// PCDHGA6 /// PCDHGA7 /// PCDHGA8 /// PCDHGA9 /// PCDHGB1 /// PCDHGB2 /// PCDHGB3 /// PCDHGB4 /// PCDHGB5 /// PCDHGB6 /// PCDHGB7 /// PCDHGC3 /// PCDHGC4 /// PCDHGC5						
211070_x_at	DBI	1174.659072	1046.502261	1108.730524	1318.510228	1661.21879	0.125
211746_x_at	PSMA1	1174.659072	1318.510228	1046.502261	932.327523	1479.977691	0.125
211954_s_at	IPO5	1174.659072	1174.659072	987.7666025	1174.659072	1567.981744	0.125
211998_at	H3F3A /// H3F3B	1174.659072	1661.21879	1174.659072	1174.659072	880	0.125
212077_at	CALD1	1174.659072	1174.659072	1479.977691	1108.730524	587.3295358	0.125
212295_s_at	SLC7A1	1174.659072	1244.507935	1108.730524	1244.507935	880	0.125
212296_at	PSMD14	1174.659072	830.6093952	1174.659072	1174.659072	1567.981744	0.125
212928_at	TSPYL4	1174.659072	1244.507935	1046.502261	1174.659072	523.2511306	0.125
213009_s_at	TRIM37	1174.659072	1244.507935	1046.502261	1396.912926	932.327523	0.125
214609_at	PHOX2A	1174.659072	1244.507935	1396.912926	1108.730524	174.6141157	0.1875
217356_s_at	LOC100652805 /// LOC100653302 /// PGK1	1174.659072	1108.730524	987.7666025	1174.659072	1479.977691	0.125
217854_s_at	POLR2E	1174.659072	830.6093952	1174.659072	1174.659072	1318.510228	0.125
218074_at	FAM96B	1174.659072	1174.659072	1244.507935	1108.730524	659.2551138	0.125
218097_s_at	CUEDC2	1174.659072	1108.730524	1174.659072	1244.507935	659.2551138	0.125
218237_s_at	SLC38A1	1174.659072	1396.912926	987.7666025	1396.912926	987.7666025	0.125
218888_s_at	NETO2	1174.659072	1479.977691	1046.502261	1244.507935	587.3295358	0.125
221539_at	EIF4EBP1	1174.659072	987.7666025	1046.502261	1174.659072	1661.21879	0.125
62987_r_at	CACNG4	1174.659072	1244.507935	1567.981744	1046.502261	523.2511306	0.125

200020_at	TARDBP	1244.507935	1108.730524	1396.912926	1046.502261	1661.21879	0.125
200046_at	DAD1	1244.507935	1046.502261	1108.730524	1396.912926	1567.981744	0.125
200086_s_at	COX4I1	1244.507935	1244.507935	1318.510228	1479.977691	1046.502261	0.125
200673_at	LAPTM4A	1244.507935	1174.659072	1661.21879	1174.659072	1318.510228	0.125
200832_s_at	SCD	1244.507935	1318.510228	880	1174.659072	1396.912926	0.125
200871_s_at	PSAP	1244.507935	1174.659072	1661.21879	1318.510228	1046.502261	0.125
201068_s_at	PSMC2	1244.507935	1396.912926	1108.730524	1046.502261	1567.981744	0.125
201256_at	COX7A2L	1244.507935	1567.981744	1244.507935	1174.659072	1174.659072	0.125
201303_at	EIF4A3	1244.507935	1567.981744	1174.659072	1318.510228	987.7666025	0.125
201462_at	SCRN1	1244.507935	1318.510228	1174.659072	1396.912926	830.6093952	0.125
201784_s_at	C11orf58	1244.507935	1318.510228	1108.730524	932.327523	1396.912926	0.125
201923_at	PRDX4	1244.507935	1174.659072	932.327523	1174.659072	1396.912926	0.125
203130_s_at	KIF5C	1244.507935	1661.21879	987.7666025	1396.912926	246.9416506	0.1875
203414_at	MMD	1244.507935	1661.21879	1244.507935	1244.507935	523.2511306	0.1875
203462_x_at	EIF3B	1244.507935	880	1318.510228	1174.659072	1567.981744	0.125
204050_s_at	CLTA	1244.507935	1318.510228	1244.507935	1244.507935	880	0.125
204386_s_at	MRP63	1244.507935	1108.730524	987.7666025	1318.510228	1479.977691	0.125
204805_s_at	H1FX	1244.507935	1046.502261	1244.507935	1567.981744	1108.730524	0.125
205311_at	DDC	1244.507935	1244.507935	1244.507935	2093.004522	61.73541266	0.25
208549_x_at	PTMA	1244.507935	1479.977691	1046.502261	1318.510228	1108.730524	0.125
208667_s_at	ST13	1244.507935	1479.977691	659.2551138	1244.507935	1244.507935	0.125
208723_at	USP11	1244.507935	1244.507935	1244.507935	1174.659072	880	0.125
208787_at	MRPL3	1244.507935	1174.659072	1108.730524	1318.510228	1567.981744	0.125
208852_s_at	CANX	1244.507935	1318.510228	987.7666025	1174.659072	1567.981744	0.125
209066_x_at	UQCRB	1244.507935	1174.659072	932.327523	1244.507935	1479.977691	0.125
209669_s_at	SERBP1	1244.507935	987.7666025	1108.730524	1244.507935	1479.977691	0.125
209840_s_at	LRRN3	1244.507935	1760	1567.981744	932.327523	174.6141157	0.21875
210046_s_at	IDH2	1244.507935	1046.502261	1108.730524	1396.912926	1396.912926	0.125
210142_x_at	FLOT1	1244.507935	1174.659072	1244.507935	1396.912926	830.6093952	0.125
210221_at	CHRNA3	1244.507935	1567.981744	1174.659072	1244.507935	195.997718	0.1875
210547_x_at	ICA1	1244.507935	1174.659072	1318.510228	1244.507935	103.8261744	0.1875
210759_s_at	PSMA1	1244.507935	1318.510228	1174.659072	987.7666025	1479.977691	0.125
212372_at	MYH10	1244.507935	1244.507935	1567.981744	1174.659072	880	0.125
212854_x_at	NBPF10	1244.507935	1108.730524	1567.981744	830.6093952	1396.912926	0.125
213166_x_at	MIR4784 /// MZT2A	1244.507935	1244.507935	880	1244.507935	1244.507935	0.125

213932_x_at	HLA-A	1244.507935	880	1567.981744	932.327523	1975.533205	0.1875
214035_x_at	LOC399491	1244.507935	1318.510228	1396.912926	1108.730524	783.990872	0.125
215780_s_at	SET /// SETP4	1244.507935	1174.659072	932.327523	1318.510228	1318.510228	0.125
218051_s_at	NT5DC2	1244.507935	1244.507935	1244.507935	1396.912926	698.4564629	0.125
218250_s_at	CNOT7	1244.507935	1244.507935	1244.507935	1244.507935	1760	0.125
218357_s_at	TIMM8B	1244.507935	932.327523	1479.977691	1244.507935	1174.659072	0.125
218482_at	ENY2	1244.507935	1108.730524	1244.507935	1174.659072	1864.655046	0.125
218740_s_at	CDK5RAP3	1244.507935	1479.977691	1567.981744	987.7666025	698.4564629	0.125
220138_at	HAND1	1244.507935	1760	1174.659072	1244.507935	329.6275569	0.1875
39729_at	PRDX2	1244.507935	1174.659072	1244.507935	1244.507935	783.990872	0.125
45572_s_at	GGA1	1244.507935	1108.730524	1396.912926	1396.912926	1046.502261	0.125
50277_at	GGA1	1244.507935	1108.730524	1479.977691	1396.912926	1108.730524	0.125
200023_s_at	EIF3F	1318.510228	1567.981744	1661.21879	1046.502261	1108.730524	0.125
200084_at	C11orf58	1318.510228	1396.912926	1046.502261	1174.659072	1396.912926	0.125
200639_s_at	YWHAZ	1318.510228	1318.510228	987.7666025	1244.507935	1567.981744	0.125
200824_at	GSTP1	1318.510228	880	1318.510228	1318.510228	1396.912926	0.125
200942_s_at	HSBP1	1318.510228	1396.912926	1174.659072	1479.977691	830.6093952	0.125
200964_at	UBA1	1318.510228	1174.659072	1318.510228	1244.507935	1661.21879	0.125
201112_s_at	CSE1L	1318.510228	1244.507935	1108.730524	1396.912926	1661.21879	0.125
201168_x_at	ARHGDIA	1318.510228	1244.507935	1479.977691	1396.912926	880	0.125
201416_at	SOX4	1318.510228	1396.912926	1975.533205	830.6093952	1174.659072	0.1875
201417_at	SOX4	1318.510228	1396.912926	1760	1046.502261	1318.510228	0.125
201522_x_at	PAR-SN /// SNORD107 /// SNRPN /// SNURF	1318.510228	1479.977691	1174.659072	1396.912926	1108.730524	0.125
201593_s_at	ZC3H15	1318.510228	1396.912926	880	1318.510228	1318.510228	0.125
201709_s_at	NIPSNAP1	1318.510228	1244.507935	1396.912926	1479.977691	880	0.125
201890_at	RRM2	1318.510228	1396.912926	1046.502261	1479.977691	1244.507935	0.125
202107_s_at	MCM2	1318.510228	1174.659072	1318.510228	1244.507935	1661.21879	0.125
202428_x_at	DBI	1318.510228	1244.507935	1174.659072	1396.912926	1760	0.125
202478_at	TRIB2	1318.510228	1567.981744	622.2539674	1567.981744	1108.730524	0.125
202567_at	SNRPD3	1318.510228	1174.659072	1244.507935	1318.510228	1567.981744	0.125
202596_at	ENSA	1318.510228	1396.912926	1046.502261	1174.659072	1567.981744	0.125
203804_s_at	LUC7L3	1318.510228	1479.977691	1318.510228	1174.659072	932.327523	0.125
204697_s_at	CHGA	1318.510228	1244.507935	1318.510228	2093.004522	146.832384	0.25

205644_s_at	SNRPG	1318.510228	1244.507935	932.327523	1396.912926	1318.510228	0.125
206104_at	ISL1	1318.510228	1396.912926	1760	1174.659072	440	0.1875
207121_s_at	MAPK6	1318.510228	1174.659072	932.327523	1567.981744	1396.912926	0.125
208308_s_at	GPI	1318.510228	1318.510228	1244.507935	1661.21879	1244.507935	0.125
208313_s_at	SF1	1318.510228	1567.981744	1396.912926	1174.659072	1244.507935	0.125
208655_at	CCNI	1318.510228	1760	1244.507935	1318.510228	1244.507935	0.125
208675_s_at	DDOST	1318.510228	1108.730524	1567.981744	1174.659072	1479.977691	0.125
208688_x_at	EIF3B	1318.510228	830.6093952	1318.510228	1318.510228	1479.977691	0.125
208693_s_at	GARS	1318.510228	1108.730524	1174.659072	1567.981744	1318.510228	0.125
208712_at	CCND1	1318.510228	1479.977691	1174.659072	932.327523	1479.977691	0.125
208749_x_at	FLOT1	1318.510228	1244.507935	1318.510228	1396.912926	830.6093952	0.125
208788_at	ELOVL5	1318.510228	1244.507935	932.327523	1396.912926	1244.507935	0.125
208909_at	UQCRFS1	1318.510228	1318.510228	932.327523	1318.510228	1479.977691	0.125
209046_s_at	GABARAPL2	1318.510228	1396.912926	1244.507935	1318.510228	830.6093952	0.125
209226_s_at	TNPO1	1318.510228	1479.977691	1046.502261	1318.510228	1318.510228	0.125
209563_x_at	CALM1 /// CALM2 /// CALM3	1318.510228	1396.912926	1174.659072	1864.655046	1108.730524	0.125
209710_at	GATA2	1318.510228	1318.510228	1567.981744	1244.507935	440	0.1875
211945_s_at	ITGB1	1318.510228	1318.510228	1396.912926	1318.510228	987.7666025	0.125
211963_s_at	ARPC5	1318.510228	1174.659072	1396.912926	1108.730524	1567.981744	0.125
212015_x_at	PTBP1	1318.510228	1244.507935	1244.507935	1396.912926	1760	0.125
212387_at	TCF4	1318.510228	1244.507935	1318.510228	1396.912926	987.7666025	0.125
213655_at	---	1318.510228	1479.977691	1567.981744	1174.659072	1108.730524	0.125
213720_s_at	SMARCA4	1318.510228	1479.977691	1174.659072	1479.977691	1108.730524	0.125
213729_at	PRPF40A	1318.510228	1244.507935	987.7666025	1318.510228	1396.912926	0.125
216215_s_at	RBFOX2	1318.510228	1244.507935	1396.912926	1396.912926	932.327523	0.125
216295_s_at	CLTA	1318.510228	1479.977691	1567.981744	1174.659072	1108.730524	0.125
217725_x_at	SERBP1	1318.510228	1396.912926	987.7666025	1318.510228	1318.510228	0.125
217772_s_at	MTCH2	1318.510228	1174.659072	987.7666025	1396.912926	1479.977691	0.125
218258_at	POLR1D	1318.510228	1396.912926	1244.507935	1396.912926	932.327523	0.125
219791_s_at	NBLA00301	1318.510228	2217.461048	1864.655046	880	48.9994295	0.375
222231_s_at	LRRC59	1318.510228	1396.912926	1174.659072	1567.981744	1244.507935	0.125
200005_at	EIF3D	1396.912926	1244.507935	1396.912926	1396.912926	1661.21879	0.125
200008_s_at	GDI2	1396.912926	1396.912926	1318.510228	1479.977691	1046.502261	0.125
200746_s_at	GNB1	1396.912926	1244.507935	1661.21879	1318.510228	1479.977691	0.125
200803_s_at	TMBIM6	1396.912926	1318.510228	1174.659072	1479.977691	1661.21879	0.125

200960_x_at	CLTA	1396.912926	1567.981744	1318.510228	1318.510228	987.7666025	0.125
200989_at	HIF1A	1396.912926	1396.912926	1661.21879	1396.912926	1174.659072	0.125
201220_x_at	CTBP2	1396.912926	1396.912926	1396.912926	1396.912926	932.327523	0.125
201272_at	AKR1B1	1396.912926	1479.977691	1760	1318.510228	493.8833013	0.1875
201400_at	PSMB3	1396.912926	1174.659072	1244.507935	1396.912926	1567.981744	0.125
201518_at	CBX1	1396.912926	1567.981744	1396.912926	1318.510228	987.7666025	0.125
201669_s_at	MARCKS	1396.912926	1661.21879	1479.977691	1174.659072	783.990872	0.125
201938_at	CDK2AP1	1396.912926	1396.912926	1760	1318.510228	1108.730524	0.125
201999_s_at	DYNLT1	1396.912926	1479.977691	1318.510228	1396.912926	1046.502261	0.125
203832_at	SNRPF	1396.912926	1396.912926	880	1318.510228	1661.21879	0.125
204915_s_at	SOX11	1396.912926	1975.533205	1661.21879	1108.730524	739.9888454	0.1875
205370_x_at	DBT	1396.912926	1108.730524	1244.507935	1661.21879	1567.981744	0.125
207508_at	ATP5G3	1396.912926	1396.912926	1108.730524	1479.977691	1760	0.125
208620_at	PCBP1	1396.912926	1864.655046	1318.510228	1396.912926	1108.730524	0.125
208726_s_at	EIF2S2	1396.912926	1244.507935	1108.730524	1479.977691	1567.981744	0.125
210645_s_at	TTC3 /// TTC3P1	1396.912926	1760	1318.510228	1396.912926	987.7666025	0.125
212099_at	RHOB	1396.912926	1567.981744	1174.659072	1174.659072	1567.981744	0.125
212281_s_at	TMEM97	1396.912926	1479.977691	1046.502261	1318.510228	1479.977691	0.125
212282_at	TMEM97	1396.912926	1567.981744	1046.502261	1244.507935	1479.977691	0.125
212426_s_at	YWHAQ	1396.912926	1661.21879	1244.507935	1479.977691	1318.510228	0.125
213762_x_at	RBMX /// SNORD61	1396.912926	1661.21879	1174.659072	1396.912926	1396.912926	0.125
214096_s_at	SHMT2	1396.912926	1244.507935	1108.730524	1567.981744	1396.912926	0.125
214882_s_at	SRSF2	1396.912926	1567.981744	1479.977691	1174.659072	1174.659072	0.125
215313_x_at	HLA-A	1396.912926	1046.502261	1567.981744	1174.659072	1661.21879	0.125
215952_s_at	OAZ1	1396.912926	1318.510228	1174.659072	1479.977691	1661.21879	0.125
217225_x_at	NOMO1 /// NOMO2 /// NOMO3	1396.912926	1396.912926	1567.981744	1479.977691	1108.730524	0.125
217927_at	SPCS1	1396.912926	1318.510228	1479.977691	1567.981744	1046.502261	0.125
218820_at	C14orf132	1396.912926	1760	1318.510228	1396.912926	466.1637615	0.1875
221483_s_at	ARPP19	1396.912926	1396.912926	1318.510228	1396.912926	987.7666025	0.125
200091_s_at	RPS25	1479.977691	1479.977691	1864.655046	1479.977691	1479.977691	0.125
200624_s_at	MATR3 /// SNHG4	1479.977691	1567.981744	1174.659072	1479.977691	1479.977691	0.125
200644_at	MARCKSL1	1479.977691	1396.912926	1760	1479.977691	1396.912926	0.125

200675_at	CD81	1479.977691	1567.981744	1661.21879	1396.912926	1244.507935	0.125
200738_s_at	LOC100652805 /// LOC100653302 /// PGK1	1479.977691	1396.912926	1318.510228	1479.977691	1760	0.125
200745_s_at	GNB1	1479.977691	1244.507935	1567.981744	1318.510228	1661.21879	0.125
200786_at	PSMB7	1479.977691	1396.912926	1174.659072	1479.977691	1661.21879	0.125
200812_at	CCT7	1479.977691	1479.977691	1174.659072	1479.977691	1760	0.125
200873_s_at	CCT8	1479.977691	1479.977691	987.7666025	1479.977691	1479.977691	0.125
200903_s_at	AHCY	1479.977691	1479.977691	1108.730524	1661.21879	1396.912926	0.125
200943_at	HMGN1	1479.977691	1975.533205	987.7666025	1760	1174.659072	0.125
201103_x_at	LOC100506032 /// NBPF10 /// NBPF11 /// NBPF12 /// NBPF15 /// NBPF16 /// NBPF24 /// NBPF7 /// NBPF8 /// NBPF9	1479.977691	1318.510228	1760	1174.659072	1567.981744	0.125
201290_at	SEC11A	1479.977691	1479.977691	1318.510228	1479.977691	1046.502261	0.125
201555_at	MCM3	1479.977691	1396.912926	1244.507935	1661.21879	1567.981744	0.125
201565_s_at	ID2	1479.977691	1479.977691	1567.981744	659.2551138	1661.21879	0.125
201761_at	MTHFD2	1479.977691	1567.981744	987.7666025	1479.977691	1396.912926	0.125
202391_at	BASP1	1479.977691	1864.655046	1174.659072	1174.659072	2093.004522	0.1875
202858_at	U2AF1	1479.977691	1396.912926	1244.507935	1661.21879	1479.977691	0.125
203113_s_at	EEF1D	1479.977691	1396.912926	1567.981744	1318.510228	1975.533205	0.125
204538_x_at	NPIP	1479.977691	1567.981744	1760	1318.510228	987.7666025	0.125
205711_x_at	ATP5C1	1479.977691	1479.977691	1479.977691	1864.655046	1479.977691	0.125
207507_s_at	ATP5G3	1479.977691	1396.912926	1174.659072	1567.981744	1661.21879	0.125
207657_x_at	TNPO1	1479.977691	1567.981744	1174.659072	1396.912926	1567.981744	0.125
208113_x_at	PABPC3	1479.977691	1567.981744	1661.21879	1244.507935	1318.510228	0.125
208691_at	TFRC	1479.977691	1396.912926	1174.659072	1567.981744	1760	0.125
208821_at	SNRPB	1479.977691	1244.507935	1396.912926	1661.21879	1567.981744	0.125
208833_s_at	ATXN10	1479.977691	1479.977691	1108.730524	1479.977691	1567.981744	0.125
208956_x_at	DUT	1479.977691	1567.981744	1318.510228	1760	1244.507935	0.125

210466_s_at	SERBP1	1479.977691	1318.510228	1244.507935	1661.21879	1567.981744	0.125
210835_s_at	CTBP2	1479.977691	1479.977691	1479.977691	1479.977691	1046.502261	0.125
210891_s_at	GTF2I /// GTF2IP1 /// LOC100093631	1479.977691	1661.21879	1661.21879	1244.507935	1046.502261	0.125
211047_x_at	AP2S1	1479.977691	1318.510228	1864.655046	1479.977691	1479.977691	0.125
212718_at	PAPOLA	1479.977691	1479.977691	1244.507935	1396.912926	1760	0.125
212782_x_at	POLR2J	1479.977691	1567.981744	1567.981744	1396.912926	1108.730524	0.125
216274_s_at	SEC11A	1479.977691	1479.977691	1396.912926	1479.977691	1046.502261	0.125
216515_x_at	---	1479.977691	1975.533205	1567.981744	1396.912926	1396.912926	0.125
217294_s_at	ENO1	1479.977691	1318.510228	1567.981744	1396.912926	2349.318143	0.1875
217982_s_at	MORF4L1	1479.977691	1396.912926	1244.507935	1661.21879	1479.977691	0.125
220147_s_at	FAM60A	1479.977691	1567.981744	1396.912926	1567.981744	1046.502261	0.125
221829_s_at	TNPO1	1479.977691	1661.21879	1174.659072	1396.912926	1479.977691	0.125
221923_s_at	NPM1	1479.977691	1567.981744	1174.659072	1479.977691	1975.533205	0.125
200634_at	PFN1	1567.981744	1661.21879	1975.533205	1567.981744	1318.510228	0.125
200638_s_at	YWHAZ	1567.981744	1567.981744	1244.507935	1396.912926	1661.21879	0.125
200679_x_at	HMGB1	1567.981744	1479.977691	1318.510228	1661.21879	1760	0.125
200681_at	GLO1	1567.981744	1479.977691	1318.510228	1760	2217.461048	0.125
200762_at	DPYSL2	1567.981744	1661.21879	1244.507935	1479.977691	1661.21879	0.125
200792_at	XRCC6	1567.981744	1396.912926	1046.502261	1661.21879	1760	0.125
201071_x_at	SF3B1	1567.981744	1864.655046	1479.977691	1479.977691	1567.981744	0.125
201338_x_at	GTF3A	1567.981744	1396.912926	1108.730524	1661.21879	1661.21879	0.125
201891_s_at	B2M	1567.981744	1396.912926	1479.977691	1567.981744	1864.655046	0.125
202088_at	SLC39A6	1567.981744	1864.655046	1396.912926	1661.21879	987.7666025	0.125
202110_at	COX7B	1567.981744	1396.912926	1046.502261	1661.21879	1864.655046	0.125
202154_x_at	TUBB3	1567.981744	1479.977691	1661.21879	1760	1046.502261	0.125
202899_s_at	SRSF3	1567.981744	1760	1396.912926	1396.912926	1661.21879	0.125
203554_x_at	PTTG1	1567.981744	1479.977691	1396.912926	1864.655046	1567.981744	0.125
207040_s_at	ST13	1567.981744	1864.655046	1046.502261	1479.977691	1479.977691	0.125
207573_x_at	ATP5L	1567.981744	1244.507935	1479.977691	1661.21879	1864.655046	0.125
208639_x_at	PDIA6	1567.981744	1567.981744	1479.977691	1318.510228	1864.655046	0.125
208662_s_at	TTC3 /// TTC3P1	1567.981744	1864.655046	1244.507935	1661.21879	1396.912926	0.125
209014_at	MAGED1	1567.981744	1396.912926	1760	1396.912926	1661.21879	0.125
209503_s_at	PSMC5	1567.981744	1760	1396.912926	1760	1108.730524	0.125
211270_x_at	PTBP1	1567.981744	1479.977691	1396.912926	1567.981744	1864.655046	0.125

212071_s_at	SPTBN1	1567.981744	1661.21879	1567.981744	1479.977691	830.6093952	0.125
213048_s_at	---	1567.981744	1396.912926	1396.912926	1661.21879	1760	0.125
213366_x_at	ATP5C1	1567.981744	1567.981744	1318.510228	1760	1567.981744	0.125
213612_x_at	LOC100506032 /// NBPF10 /// NBPF11 /// NBPF12 /// NBPF15 /// NBPF16 /// NBPF24 /// NBPF7 /// NBPF8 /// NBPF9	1567.981744	1396.912926	1760	1174.659072	1661.21879	0.125
216384_x_at	LOC100506248 /// LOC728026 /// MIR1244-1 /// MIR1244-2 /// MIR1244-3 /// PTMA	1567.981744	1174.659072	1760	1661.21879	1479.977691	0.125
217773_s_at	NDUFA4	1567.981744	1108.730524	1396.912926	1760	1975.533205	0.125
217848_s_at	PPA1	1567.981744	1567.981744	1396.912926	1864.655046	1567.981744	0.125
220864_s_at	NDUFA13	1567.981744	1318.510228	1479.977691	1760	1661.21879	0.125
221891_x_at	HSPA8 /// SNORD14C /// SNORD14D	1567.981744	1661.21879	1396.912926	1567.981744	1864.655046	0.125
200006_at	PARK7	1661.21879	1318.510228	1661.21879	1479.977691	1975.533205	0.125
200009_at	GDI2	1661.21879	1661.21879	1567.981744	1864.655046	1396.912926	0.125
200033_at	DDX5	1661.21879	1975.533205	1661.21879	1661.21879	1479.977691	0.125
200627_at	LOC100506732 /// PTGES3	1661.21879	1760	1396.912926	1567.981744	1864.655046	0.125
200652_at	SSR2	1661.21879	1661.21879	1864.655046	1567.981744	1046.502261	0.125
200844_s_at	PRDX6	1661.21879	1760	1661.21879	1567.981744	1318.510228	0.125
200853_at	H2AFZ	1661.21879	1760	1396.912926	1760	1661.21879	0.125
200876_s_at	PSMB1	1661.21879	1567.981744	1396.912926	1760	1760	0.125
200886_s_at	PGAM1	1661.21879	1479.977691	1396.912926	1760	1864.655046	0.125
202961_s_at	ATP5J2	1661.21879	1318.510228	1760	1567.981744	1567.981744	0.125
203752_s_at	JUND	1661.21879	1864.655046	1479.977691	1661.21879	1479.977691	0.125

204337_at	RGS4	1661.21879	1864.655046	2093.004522	1479.977691	220	0.25
204914_s_at	SOX11	1661.21879	2489.01587	1975.533205	1396.912926	1108.730524	0.1875
207332_s_at	TFRC	1661.21879	1661.21879	1174.659072	1661.21879	2093.004522	0.125
208029_s_at	LAPTM4B	1661.21879	1479.977691	1318.510228	1760	1864.655046	0.125
208073_x_at	TTC3 /// TTC3P1	1661.21879	1975.533205	1396.912926	1760	1396.912926	0.125
208641_s_at	RAC1	1661.21879	987.7666025	1864.655046	1567.981744	1567.981744	0.125
208643_s_at	XRCC5	1661.21879	2093.004522	1661.21879	1661.21879	1396.912926	0.125
208746_x_at	ATP5L	1661.21879	1318.510228	1479.977691	1661.21879	1975.533205	0.125
208775_at	XPO1	1661.21879	1864.655046	1244.507935	1567.981744	1661.21879	0.125
208780_x_at	VAPA	1661.21879	1661.21879	1174.659072	1661.21879	1864.655046	0.125
208805_at	KIAA0391 /// PSMA6	1661.21879	1567.981744	1479.977691	1760	1864.655046	0.125
208870_x_at	ATP5C1	1661.21879	1661.21879	1479.977691	1975.533205	1661.21879	0.125
210453_x_at	ATP5L	1661.21879	1318.510228	1479.977691	1661.21879	1975.533205	0.125
210470_x_at	NONO	1661.21879	1864.655046	1661.21879	1396.912926	1567.981744	0.125
210927_x_at	JTB	1661.21879	1760	1864.655046	1479.977691	1567.981744	0.125
211716_x_at	ARHGDIA	1661.21879	1661.21879	1567.981744	1760	1108.730524	0.125
211762_s_at	KPNA2	1661.21879	1760	1318.510228	1864.655046	1479.977691	0.125
212279_at	TMEM97	1661.21879	1661.21879	1174.659072	1567.981744	1661.21879	0.125
212386_at	TCF4	1661.21879	1661.21879	1661.21879	1760	1318.510228	0.125
214870_x_at	LOC100288332 /// LOC100506193 /// LOC642778 /// LOC642799 /// NPIP /// PKD1P1	1661.21879	1760	1864.655046	1479.977691	880	0.125
215230_x_at	EIF3C /// EIF3CL	1661.21879	1661.21879	1567.981744	1864.655046	1318.510228	0.125
217491_x_at	COX7C	1661.21879	1479.977691	1174.659072	1760	1975.533205	0.125
221501_x_at	LOC100288332 /// LOC642778 /// LOC642799 /// PKD1P1	1661.21879	1760	1864.655046	1479.977691	987.7666025	0.125
200723_s_at	CAPRIN1	1760	1760	1661.21879	1567.981744	2217.461048	0.125
200748_s_at	FTH1	1760	1864.655046	1661.21879	1174.659072	1661.21879	0.125
200779_at	ATF4	1760	1864.655046	1174.659072	1864.655046	1567.981744	0.125

200818_at	ATP5O	1760	1661.21879	1244.507935	1864.655046	1760	0.125
200910_at	CCT3	1760	2093.004522	1396.912926	1661.21879	1760	0.125
201128_s_at	ACLY	1760	1661.21879	1661.21879	1864.655046	2093.004522	0.125
201231_s_at	ENO1	1760	1479.977691	1864.655046	1661.21879	2637.020455	0.1875
201310_s_at	NREP	1760	1864.655046	1661.21879	1661.21879	987.7666025	0.125
201592_at	EIF3H	1760	1864.655046	1567.981744	1661.21879	1975.533205	0.125
201946_s_at	CCT2	1760	1760	1396.912926	1661.21879	2093.004522	0.125
201947_s_at	CCT2	1760	1661.21879	1661.21879	1760	2093.004522	0.125
205347_s_at	TMSB15A /// TMSB15B	1760	1760	1661.21879	1760	1108.730524	0.125
206445_s_at	PRMT1	1760	1479.977691	1661.21879	1760	1975.533205	0.125
208640_at	RAC1	1760	1318.510228	1864.655046	1760	1661.21879	0.125
208656_s_at	CCNI	1760	2217.461048	1760	1760	1661.21879	0.125
208705_s_at	EIF5	1760	1760	1244.507935	1661.21879	1760	0.125
208739_x_at	SUMO2	1760	2093.004522	1567.981744	1975.533205	1567.981744	0.125
211600_at	PTPRO	1760	2093.004522	1975.533205	1567.981744	1244.507935	0.125
211623_s_at	FBL	1760	1661.21879	1760	1760	2093.004522	0.125
211628_x_at	FTH1P5	1760	1864.655046	1661.21879	1396.912926	1760	0.125
211933_s_at	HNRNPA3 /// HNRNPA3P1	1760	1864.655046	1174.659072	1661.21879	1760	0.125
212233_at	MAP1B	1760	2217.461048	1567.981744	1864.655046	698.4564629	0.21875
212509_s_at	MXRA7	1760	2489.01587	1661.21879	1760	1108.730524	0.1875
213101_s_at	ACTR3	1760	1661.21879	1661.21879	1661.21879	2217.461048	0.125
216231_s_at	B2M	1760	1567.981744	1661.21879	1661.21879	2217.461048	0.125
218041_x_at	SLC38A2	1760	1661.21879	1760	1396.912926	2093.004522	0.125
218332_at	BEX1	1760	1864.655046	1661.21879	1864.655046	830.6093952	0.1875
221434_s_at	SLIRP	1760	1567.981744	1174.659072	1864.655046	1975.533205	0.125
221952_x_at	TRMT5	1760	1479.977691	1760	1661.21879	1975.533205	0.125
200043_at	ERH	1864.655046	1661.21879	1760	2093.004522	1864.655046	0.125
200662_s_at	TOMM20	1864.655046	2093.004522	1661.21879	1864.655046	1396.912926	0.125
200713_s_at	MAPRE1	1864.655046	1661.21879	1318.510228	1864.655046	1864.655046	0.125
200840_at	KARS	1864.655046	1760	1864.655046	2093.004522	1661.21879	0.125
200978_at	MDH1	1864.655046	1864.655046	1318.510228	1864.655046	1760	0.125
201134_x_at	COX7C	1864.655046	1760	1244.507935	1975.533205	2093.004522	0.125
201251_at	PKM	1864.655046	1479.977691	1864.655046	1760	1975.533205	0.125
201426_s_at	VIM	1864.655046	2349.318143	2637.020455	1479.977691	1318.510228	0.21875

201568_at	UQCRQ	1864.655046	1567.981744	1760	2093.004522	1760	0.125
201742_x_at	SRSF1	1864.655046	2093.004522	1661.21879	1661.21879	1864.655046	0.125
202503_s_at	KIAA0101	1864.655046	1864.655046	1318.510228	1760	1975.533205	0.125
202589_at	TYMS	1864.655046	1975.533205	1760	1864.655046	1479.977691	0.125
202698_x_at	COX4I1	1864.655046	1864.655046	1760	2217.461048	1479.977691	0.125
207009_at	PHOX2B	1864.655046	2637.020455	1760	1864.655046	110	0.4375
208905_at	CYCS	1864.655046	1760	1479.977691	1975.533205	1975.533205	0.125
209604_s_at	GATA3	1864.655046	2217.461048	2093.004522	1661.21879	369.9944227	0.25
210434_x_at	JTB	1864.655046	1975.533205	1975.533205	1567.981744	1760	0.125
214359_s_at	HSP90AB1	1864.655046	1864.655046	1975.533205	1864.655046	1479.977691	0.125
217719_at	EIF3L	1864.655046	2093.004522	1864.655046	1864.655046	1661.21879	0.125
217724_at	SERBP1	1864.655046	1864.655046	1479.977691	1864.655046	1975.533205	0.125
217753_s_at	RPS26	1864.655046	1864.655046	1864.655046	1864.655046	1479.977691	0.125
200036_s_at	RPL10A	1975.533205	2093.004522	2217.461048	1864.655046	1864.655046	0.125
200631_s_at	SET	1975.533205	2093.004522	1479.977691	1864.655046	2093.004522	0.125
200645_at	GABARAP	1975.533205	1975.533205	1975.533205	1864.655046	1174.659072	0.125
200657_at	SLC25A5	1975.533205	1975.533205	1661.21879	2093.004522	1864.655046	0.125
200773_x_at	PTMA	1975.533205	2349.318143	1975.533205	1864.655046	1864.655046	0.125
200790_at	ODC1	1975.533205	2093.004522	1864.655046	4186.009045	1864.655046	0.5
201241_at	DDX1	1975.533205	4186.009045	1174.659072	3322.437581	1174.659072	1.75
201268_at	NME1-NME2 /// NME2	1975.533205	2093.004522	1661.21879	2093.004522	1661.21879	0.125
201754_at	COX6C	1975.533205	1864.655046	1479.977691	2093.004522	2217.461048	0.125
208680_at	PRDX1	1975.533205	2093.004522	1760	1975.533205	2349.318143	0.125
208697_s_at	EIF3E	1975.533205	1975.533205	1760	1975.533205	2489.01587	0.125
209771_x_at	CD24	1975.533205	2093.004522	1975.533205	1864.655046	739.9888454	0.1875
210231_x_at	SET	1975.533205	1864.655046	1760	1975.533205	2217.461048	0.125
210532_s_at	C14orf2	1975.533205	1975.533205	1479.977691	1975.533205	1864.655046	0.125
211071_s_at	MLLT11	1975.533205	2637.020455	1864.655046	1975.533205	1396.912926	0.1875
211999_at	H3F3A /// H3F3B	1975.533205	2489.01587	1661.21879	2093.004522	1760	0.125
213175_s_at	SNRPB	1975.533205	1661.21879	1760	2093.004522	2093.004522	0.125
213699_s_at	YWHAQ	1975.533205	2217.461048	1661.21879	1975.533205	1864.655046	0.125
214687_x_at	ALDOA	1975.533205	1975.533205	1760	1975.533205	2217.461048	0.125
214853_s_at	SHC1	1975.533205	2093.004522	2093.004522	1760	1318.510228	0.125
41220_at	41526	1975.533205	1864.655046	1975.533205	1975.533205	1318.510228	0.125
200057_s_at	NONO	2093.004522	2349.318143	2217.461048	1975.533205	1864.655046	0.125

200068_s_at	CANX	2093.004522	2093.004522	1760	1975.533205	2489.01587	0.125
200077_s_at	OAZ1	2093.004522	1975.533205	1975.533205	2093.004522	2349.318143	0.125
200610_s_at	NCL	2093.004522	2093.004522	1479.977691	1975.533205	2093.004522	0.125
200754_x_at	SRSF2	2093.004522	2217.461048	2217.461048	1864.655046	1661.21879	0.125
200822_x_at	TPI1	2093.004522	1864.655046	1975.533205	2217.461048	2217.461048	0.125
200912_s_at	EIF4A2 /// MIR1248 /// SNORA4 /// SNORA63 /// SNORA81 /// SNORD2	2093.004522	2217.461048	1567.981744	1975.533205	2093.004522	0.125
205967_at	HIST1H4A /// HIST1H4B /// HIST1H4C /// HIST1H4D /// HIST1H4E /// HIST1H4F /// HIST1H4H /// HIST1H4I /// HIST1H4J /// HIST1H4K /// HIST1H4L /// HIST2H4A /// HIST2H4B /// HIST4H4	2093.004522	1864.655046	1396.912926	3135.963488	2217.461048	0.21875
208718_at	DDX17	2093.004522	2093.004522	1567.981744	1975.533205	1975.533205	0.125
209069_s_at	H3F3A /// H3F3B	2093.004522	2489.01587	1864.655046	2217.461048	1661.21879	0.125
209118_s_at	TUBA1A	2093.004522	2637.020455	1864.655046	2349.318143	1661.21879	0.125
210211_s_at	HSP90AA1	2093.004522	2217.461048	1661.21879	1975.533205	2093.004522	0.125
211939_x_at	BTF3	2093.004522	2489.01587	2093.004522	2093.004522	1864.655046	0.125
212130_x_at	EIF1	2093.004522	2217.461048	1760	2489.01587	1975.533205	0.125
212227_x_at	EIF1	2093.004522	2093.004522	1661.21879	2489.01587	1975.533205	0.125
212826_s_at	SLC25A6	2093.004522	1046.502261	1975.533205	2217.461048	2217.461048	0.1875
213476_x_at	TUBB3	2093.004522	1864.655046	2093.004522	2217.461048	1479.977691	0.125
213687_s_at	RPL35A	2093.004522	2217.461048	1864.655046	1975.533205	2489.01587	0.125
216342_x_at	---	2093.004522	1864.655046	2093.004522	1760	2217.461048	0.125

217807_s_at	GLTSCR2	2093.004522	2217.461048	1975.533205	1975.533205	1318.510228	0.125
221691_x_at	NPM1	2093.004522	2217.461048	1661.21879	2093.004522	1975.533205	0.125
200017_at	RPS27A	2217.461048	2217.461048	2093.004522	1760	2217.461048	0.125
200080_s_at	H3F3A /// H3F3AP4 /// H3F3B	2217.461048	2349.318143	2489.01587	1975.533205	1760	0.125
200630_x_at	SET	2217.461048	2093.004522	1864.655046	2217.461048	2489.01587	0.125
200647_x_at	EIF3C /// EIF3CL	2217.461048	2093.004522	2349.318143	2349.318143	1760	0.125
200705_s_at	EEF1B2 /// SNORA41	2217.461048	2959.955382	1975.533205	2217.461048	2217.461048	0.125
200877_at	CCT4	2217.461048	2217.461048	1760	2093.004522	2217.461048	0.125
201322_at	ATP5B	2217.461048	2093.004522	1975.533205	2349.318143	2349.318143	0.125
204031_s_at	PCBP2	2217.461048	2637.020455	2093.004522	2217.461048	1864.655046	0.125
208517_x_at	BTF3	2217.461048	2349.318143	2217.461048	1975.533205	1760	0.125
208668_x_at	HMGN2	2217.461048	1864.655046	2217.461048	1975.533205	2349.318143	0.125
208687_x_at	HSPA8 /// SNORD14C /// SNORD14D	2217.461048	2217.461048	1975.533205	2093.004522	2489.01587	0.125
208980_s_at	UBC	2217.461048	2093.004522	2093.004522	2093.004522	2637.020455	0.125
210949_s_at	EIF3C /// EIF3CL	2217.461048	1975.533205	2349.318143	2349.318143	1661.21879	0.125
212085_at	SLC25A6	2217.461048	1046.502261	2217.461048	2217.461048	2217.461048	0.1875
214327_x_at	TPT1	2217.461048	2637.020455	1661.21879	1975.533205	2349.318143	0.125
216379_x_at	CD24	2217.461048	2489.01587	2217.461048	1975.533205	698.4564629	0.25
220960_x_at	RPL22	2217.461048	1975.533205	2349.318143	1864.655046	2637.020455	0.125
221475_s_at	RPL15	2217.461048	2217.461048	2349.318143	2093.004522	1864.655046	0.125
200010_at	RPL11	2349.318143	2217.461048	2349.318143	1864.655046	2489.01587	0.125
200650_s_at	LDHA	2349.318143	2349.318143	1975.533205	2349.318143	2959.955382	0.125
200772_x_at	LOC100506248 /// LOC728026 /// MIR1244-1 /// MIR1244-2 /// MIR1244-3 /// PTMA	2349.318143	2959.955382	2349.318143	2349.318143	2217.461048	0.125
200806_s_at	HSPD1	2349.318143	2637.020455	1760	2217.461048	2349.318143	0.125
201577_at	NME1	2349.318143	2349.318143	1760	2349.318143	2217.461048	0.125
202021_x_at	EIF1	2349.318143	2489.01587	1760	2489.01587	2093.004522	0.125

208826_x_at	HINT1	2349.318143	2093.004522	1864.655046	2637.020455	3135.963488	0.1875
211921_x_at	LOC100506248 /// LOC728026 /// MIR1244-1 /// MIR1244-2 /// MIR1244-3 /// PTMA	2349.318143	2959.955382	2349.318143	2217.461048	2093.004522	0.125
211956_s_at	EIF1	2349.318143	2349.318143	1760	2349.318143	2217.461048	0.125
212191_x_at	RPL13 /// SNORD68	2349.318143	2349.318143	2637.020455	2349.318143	2217.461048	0.125
217871_s_at	MIF	2349.318143	1975.533205	2217.461048	2349.318143	2489.01587	0.125
221476_s_at	RPL15	2349.318143	2349.318143	2217.461048	2489.01587	1975.533205	0.125
221775_x_at	RPL22	2349.318143	2093.004522	2637.020455	1975.533205	2959.955382	0.125
AFFX-HSAC07/X00351_5_at	ACTB /// ACTB /// LOC100505829	2349.318143	2349.318143	3322.437581	2217.461048	2349.318143	0.1875
200003_s_at	RPL28	2489.01587	2793.825851	2637.020455	1975.533205	2093.004522	0.125
200030_s_at	SLC25A3	2489.01587	2637.020455	2217.461048	2489.01587	2349.318143	0.125
200063_s_at	NPM1	2489.01587	2489.01587	2093.004522	2349.318143	2489.01587	0.125
200735_x_at	NACA	2489.01587	2637.020455	2489.01587	2217.461048	2349.318143	0.125
200807_s_at	HSPD1	2489.01587	2637.020455	1975.533205	2489.01587	2489.01587	0.125
200823_x_at	RPL29	2489.01587	2217.461048	2637.020455	2793.825851	2217.461048	0.125
200888_s_at	RPL23	2489.01587	2637.020455	2489.01587	2217.461048	2349.318143	0.125
201258_at	RPS16	2489.01587	2349.318143	2637.020455	2349.318143	2093.004522	0.125
211968_s_at	HSP90AA1	2489.01587	2489.01587	1864.655046	2349.318143	2637.020455	0.125
211969_at	HSP90AA1	2489.01587	2489.01587	1975.533205	2349.318143	2349.318143	0.125
212933_x_at	RPL13 /// SNORD68	2489.01587	2489.01587	2489.01587	2489.01587	1864.655046	0.125
213187_x_at	FTL	2489.01587	2793.825851	2217.461048	1760	2793.825851	0.1875
213564_x_at	LDHB	2489.01587	2489.01587	2349.318143	2489.01587	2793.825851	0.125
AFFX-HSAC07/X00351_3_at	ACTB /// ACTB /// LOC100505829	2489.01587	2489.01587	2793.825851	2349.318143	2489.01587	0.125
200022_at	RPL18	2637.020455	2349.318143	2793.825851	2637.020455	2489.01587	0.125
200651_at	GNB2L1 /// LOC100289627	2637.020455	2793.825851	2637.020455	2489.01587	2093.004522	0.125

	/// SNORD95 /// SNORD96A						
200715_x_at	RPL13A /// SNORD32A /// SNORD33 /// SNORD34 /// SNORD35A	2637.020455	2093.004522	2793.825851	2637.020455	2637.020455	0.125
200909_s_at	RPLP2 /// SNORA52	2637.020455	2793.825851	2959.955382	2489.01587	2489.01587	0.125
200936_at	RPL8	2637.020455	2489.01587	2637.020455	2489.01587	3135.963488	0.125
201387_s_at	UCHL1	2637.020455	2637.020455	2489.01587	2217.461048	2637.020455	0.125
207721_x_at	HINT1	2637.020455	2349.318143	2093.004522	2793.825851	2959.955382	0.125
208628_s_at	YBX1	2637.020455	2637.020455	2217.461048	2637.020455	2489.01587	0.125
208635_x_at	NACA	2637.020455	2793.825851	2637.020455	2349.318143	2489.01587	0.125
208768_x_at	RPL22	2637.020455	2093.004522	2793.825851	2217.461048	2959.955382	0.125
211710_x_at	RPL4 /// SNORD16 /// SNORD18A /// SNORD18B /// SNORD18C	2637.020455	2637.020455	2793.825851	2637.020455	2349.318143	0.125
211858_x_at	GNAS	2637.020455	2793.825851	2637.020455	2489.01587	2217.461048	0.125
211943_x_at	TPT1	2637.020455	3135.963488	2489.01587	2489.01587	2637.020455	0.125
212788_x_at	FTL	2637.020455	3135.963488	2217.461048	2093.004522	3135.963488	0.1875
213347_x_at	RPS4X	2637.020455	2637.020455	2959.955382	2349.318143	2637.020455	0.125
213941_x_at	RPS7	2637.020455	2793.825851	2959.955382	2489.01587	2489.01587	0.125
217733_s_at	TMSB10	2637.020455	2959.955382	2637.020455	1661.21879	2489.01587	0.1875
200064_at	HSP90AB1	2793.825851	2793.825851	2349.318143	2959.955382	2637.020455	0.125
200082_s_at	RPS7	2793.825851	2793.825851	2793.825851	2637.020455	2349.318143	0.125
200680_x_at	HMGB1	2793.825851	2489.01587	2489.01587	2959.955382	3135.963488	0.125
200869_at	RPL18A	2793.825851	3135.963488	2793.825851	2793.825851	2637.020455	0.125
210338_s_at	HSPA8 /// SNORD14C /// SNORD14D	2793.825851	2637.020455	2489.01587	2793.825851	3135.963488	0.125
212363_x_at	ACTG1	2793.825851	3520	2637.020455	2637.020455	2793.825851	0.125
212734_x_at	RPL13 /// SNORD68	2793.825851	2637.020455	2959.955382	2793.825851	2489.01587	0.125

212988_x_at	ACTG1	2793.825851	3322.437581	2793.825851	2489.01587	2637.020455	0.125
213969_x_at	RPL29	2793.825851	2793.825851	2959.955382	2793.825851	2489.01587	0.125
214271_x_at	RPL12	2793.825851	2793.825851	2637.020455	2349.318143	2793.825851	0.125
214328_s_at	HSP90AA1	2793.825851	2959.955382	2217.461048	2637.020455	2793.825851	0.125
200018_at	LOC100508408 /// RPS13 /// SNORD14B	2959.955382	2959.955382	2793.825851	2349.318143	2793.825851	0.125
200021_at	CFL1	2959.955382	3135.963488	2959.955382	2793.825851	2637.020455	0.125
200062_s_at	RPL30	2959.955382	2959.955382	2959.955382	2793.825851	3520	0.125
200633_at	UBB	2959.955382	3135.963488	2959.955382	2637.020455	2637.020455	0.125
200741_s_at	RPS27	2959.955382	3520	3135.963488	2793.825851	2637.020455	0.125
200763_s_at	RPLP1	2959.955382	2959.955382	3135.963488	2637.020455	2637.020455	0.125
200780_x_at	GNAS	2959.955382	3135.963488	2793.825851	2793.825851	2489.01587	0.125
200981_x_at	GNAS	2959.955382	3135.963488	2959.955382	2793.825851	2489.01587	0.125
201406_at	RPL36A /// RPL36A-HNRNPH2	2959.955382	3322.437581	2959.955382	2793.825851	2793.825851	0.125
201550_x_at	ACTG1	2959.955382	3520	3135.963488	2637.020455	2637.020455	0.125
211940_x_at	H3F3A /// H3F3AP4 /// H3F3B	2959.955382	3135.963488	2959.955382	2959.955382	2637.020455	0.125
211970_x_at	ACTG1	2959.955382	3520	3135.963488	2637.020455	2637.020455	0.125
211995_x_at	ACTG1	2959.955382	3520	2959.955382	2637.020455	2793.825851	0.125
211997_x_at	H3F3A /// H3F3B	2959.955382	3520	2793.825851	3135.963488	2349.318143	0.1875
212273_x_at	GNAS	2959.955382	3322.437581	3135.963488	2793.825851	2637.020455	0.125
212537_x_at	RPL17 /// RPL17-C18ORF32	2959.955382	2959.955382	2959.955382	2637.020455	3135.963488	0.125
214938_x_at	HMGB1	2959.955382	2959.955382	2093.004522	2959.955382	3135.963488	0.125
AFFX-HSAC07/X00351_M_at	ACTB /// ACTB /// LOC100505829	2959.955382	2959.955382	3729.310092	2793.825851	2793.825851	0.125
200717_x_at	RPL7	3135.963488	3322.437581	2637.020455	2959.955382	3729.310092	0.125
200801_x_at	ACTB /// LOC100505829	3135.963488	2959.955382	3520	3135.963488	3135.963488	0.125
200809_x_at	RPL12	3135.963488	3322.437581	2959.955382	2793.825851	3322.437581	0.125
200963_x_at	RPL31	3135.963488	2489.01587	3322.437581	2959.955382	3135.963488	0.125

201665_x_at	RPS17 /// RPS17L	3135.963488	2959.955382	2959.955382	3135.963488	3322.437581	0.125
208645_s_at	RPS14	3135.963488	3135.963488	3322.437581	2959.955382	2959.955382	0.125
208755_x_at	H3F3A /// H3F3AP4 /// H3F3B	3135.963488	3135.963488	3135.963488	2959.955382	2637.020455	0.125
211983_x_at	ACTG1	3135.963488	3520	3135.963488	2637.020455	2959.955382	0.125
213214_x_at	ACTG1	3135.963488	3520	3135.963488	2637.020455	2793.825851	0.125
214548_x_at	GNAS	3135.963488	3135.963488	3135.963488	3135.963488	2793.825851	0.125
216520_s_at	TPT1	3135.963488	3135.963488	3135.963488	2793.825851	2793.825851	0.125
221607_x_at	ACTG1	3135.963488	3729.310092	3135.963488	2793.825851	2959.955382	0.125
200817_x_at	RPS10	3322.437581	3135.963488	3135.963488	3322.437581	3520	0.125
200819_s_at	RPS15	3322.437581	3322.437581	3322.437581	3135.963488	2959.955382	0.125
200933_x_at	RPS4X	3322.437581	3135.963488	3729.310092	2959.955382	3322.437581	0.125
202029_x_at	RPL38	3322.437581	3322.437581	3135.963488	3520	2489.01587	0.125
207783_x_at	HUWE1	3322.437581	3520	3322.437581	2093.004522	3135.963488	0.1875
211296_x_at	UBC	3322.437581	3135.963488	2959.955382	3322.437581	3520	0.125
211714_x_at	TUBB	3322.437581	2959.955382	3135.963488	3520	2959.955382	0.125
211927_x_at	EEF1G /// MIR3654	3322.437581	3322.437581	3322.437581	2959.955382	3322.437581	0.125
213356_x_at	HNRNPA1 /// HNRNPA1L2 /// HNRNPA1P10 /// LOC728643	3322.437581	3520	2959.955382	3135.963488	3135.963488	0.125
213614_x_at	EEF1A1 /// LOC100653236	3322.437581	3520	3135.963488	3135.963488	3520	0.125
213828_x_at	H3F3A /// H3F3AP4 /// H3F3B	3322.437581	3322.437581	3520	3135.963488	2793.825851	0.125
213867_x_at	ACTB /// LOC100505829	3322.437581	2959.955382	3729.310092	3135.963488	3135.963488	0.125
213890_x_at	RPS16	3322.437581	3322.437581	3322.437581	3322.437581	2959.955382	0.125
200095_x_at	RPS10	3520	3520	3135.963488	3520	3951.06641	0.125
201049_s_at	RPS18	3520	3135.963488	3729.310092	3322.437581	3520	0.125
201217_x_at	RNU86 /// RPL3 /// SNORD83B	3520	3322.437581	3135.963488	3520	3520	0.125

211072_x_at	LOC100288366 /// TUBA1B	3520	3322.437581	3322.437581	3520	3951.06641	0.125
211073_x_at	RNU86 /// RPL3 /// SNORD83B	3520	3729.310092	3135.963488	3520	3729.310092	0.125
211378_x_at	LOC100288602 /// PPIA	3520	3322.437581	3520	3520	3951.06641	0.125
211487_x_at	RPS17 /// RPS17L	3520	3520	3322.437581	3135.963488	3520	0.125
211750_x_at	TUBA1C	3520	3322.437581	3520	3520	3951.06641	0.125
211765_x_at	PPIA	3520	3322.437581	3322.437581	3520	3729.310092	0.125
213453_x_at	GAPDH	3520	3322.437581	3322.437581	3520	3729.310092	0.125
213477_x_at	EEF1A1 /// LOC100653236	3520	3520	3322.437581	3135.963488	3520	0.125
215963_x_at	RPL3	3520	3520	3135.963488	3322.437581	3520	0.125
201090_x_at	LOC100288366 /// TUBA1B	3729.310092	3322.437581	3322.437581	3729.310092	3729.310092	0.125
201254_x_at	RPS6	3729.310092	3729.310092	3729.310092	3322.437581	3520	0.125
201293_x_at	LOC100288602 /// PPIA	3729.310092	3520	3729.310092	3520	4186.009045	0.125
201429_s_at	RPL37A	3729.310092	3951.06641	3729.310092	3520	3520	0.125
211058_x_at	LOC100288366 /// TUBA1B	3729.310092	3322.437581	3322.437581	3729.310092	3951.06641	0.125
211720_x_at	RPLP0	3729.310092	3729.310092	3135.963488	3520	3729.310092	0.125
212661_x_at	LOC100288602 /// PPIA	3729.310092	3729.310092	3729.310092	3520	4186.009045	0.125
213646_x_at	LOC100288366 /// TUBA1B	3729.310092	3520	3520	3729.310092	3951.06641	0.125
217398_x_at	GAPDH	3729.310092	3729.310092	3520	3729.310092	3951.06641	0.125
201033_x_at	RPLP0	3951.06641	3951.06641	3520	3951.06641	3951.06641	0.125
201257_x_at	RPS3A /// SNORD73A	3951.06641	3951.06641	3729.310092	3322.437581	3729.310092	0.125
203012_x_at	RPL23A	3951.06641	3729.310092	3951.06641	3951.06641	3520	0.125
208856_x_at	RPLP0	3951.06641	3951.06641	3520	3729.310092	3951.06641	0.125
212639_x_at	LOC100288366 /// TUBA1A /// TUBA1B	3951.06641	3520	3520	3951.06641	4186.009045	0.125

208825_x_at	RPL23A /// SNORD4A	4186.009045	4186.009045	4186.009045	3729.310092	3951.06641	0.125
208834_x_at	RPL23A /// SNORD4A	4186.009045	4186.009045	4186.009045	3729.310092	4186.009045	0.125
212869_x_at	TPT1	4186.009045	4186.009045	3951.06641	3322.437581	3951.06641	0.125
213084_x_at	RPL23A /// SNORD4A	4186.009045	4186.009045	4186.009045	3520	3951.06641	0.125

Supplementary Table S2.

Frequencies of the musically interpreted microarray data from "neuroblastoma" cell lines (192 probe sets). DNA microarray data from 4 cell lines that were initially established as neuroblastoma cell lines (GSE1824) were transformed into melodies as described in the Methods section by using the following parameters: minimal frequency: 27.5; number of different frequencies (keys): 88; number of tone steps per octave: 12; minimal duration: 1/8; number of tones: 192. Presented are the frequencies of the individual cell lines and the frequency of the median signal intensity as well as the duration of the filtered 192 probe sets. Probe sets with frequencies two times higher than the median are marked in bold face. Probe sets with a duration of 0.25 or longer and a frequency above 987 Hz have been marked yellow.

Probe Set ID	Gene Symbol	Median	CHP-126	SH-SY5Y	SiMa	SK-N-MC	Duration
205478_at	PPP1R1A	61.7354127	46.2493028	65.4063913	58.2704702	**1108.7305**	0.125
209656_s_at	TMEM47	73.416192	51.9130872	103.826174	43.6535289	**987.7666**	0.125
205081_at	CRIP1	82.4068892	61.7354127	77.7817459	87.3070579	**1046.5023**	0.125
206326_at	GRP	92.4986057	92.4986057	92.4986057	92.4986057	**1661.2188**	0.21875
211675_s_at	MDFIC	97.998859	116.54094	82.4068892	58.2704702	**987.7666**	0.125
205306_x_at	KMO	103.826174	73.416192	92.4986057	110	**987.7666**	0.125
202747_s_at	ITM2A	110	116.54094	110	103.826174	**1760**	0.21875
206463_s_at	DHRS2	123.470825	**1760**	116.54094	116.54094	110	0.21875
206915_at	NKX2-2	123.470825	58.2704702	82.4068892	164.813779	**987.7666**	0.125
212097_at	CAV1	123.470825	48.9994295	**277.18263**	55	**1864.655**	0.25
201427_s_at	SEPP1	146.832384	174.614116	61.7354127	130.812783	**1046.5023**	0.125
202404_s_at	COL1A2	146.832384	138.591316	146.832384	51.9130872	**1244.5079**	0.125
201160_s_at	CSDA	155.563492	174.614116	38.890873	130.812783	**1396.9129**	0.1875
205827_at	CCK	155.563492	195.997718	116.54094	41.2034446	**1975.5332**	0.25
202746_at	ITM2A	174.614116	184.997211	164.813779	97.998859	**1479.9777**	0.1875
205227_at	IL1RAP	174.614116	103.826174	155.563492	195.997718	**1108.7305**	0.125
205968_at	KCNS3	174.614116	329.627557	82.4068892	**1174.6591**	87.3070579	0.125
209734_at	NCKAP1L	174.614116	130.812783	220	130.812783	**1244.5079**	0.125

211138_s_at	KMO	184.997211	246.941651	138.591316	103.826174	**1046.5023**	0.125
214079_at	DHRS2	184.997211	**1864.655**	146.832384	220	123.470825	0.21875
202992_at	C7	195.997718	97.998859	**1046.5023**	87.3070579	369.994423	0.125
202403_s_at	COL1A2	207.652349	110	261.625565	164.813779	**1760**	0.21875
219572_at	CADPS2	207.652349	220	164.813779	207.652349	**1174.6591**	0.125
203065_s_at	CAV1	220	87.3070579	329.627557	138.591316	**1975.5332**	0.25
209291_at	ID4	220	77.7817459	293.664768	174.614116	**1396.9129**	0.1875
200953_s_at	CCND2	233.081881	277.182631	164.813779	195.997718	**1396.9129**	0.125
201012_at	ANXA1	233.081881	61.7354127	246.941651	207.652349	**1479.9777**	0.1875
201909_at	RPS4Y1	233.081881	207.652349	155.563492	**1567.9817**	246.941651	0.1875
205547_s_at	TAGLN	233.081881	174.614116	**1108.7305**	138.591316	277.182631	0.125
205542_at	STEAP1	246.941651	69.2956577	246.941651	246.941651	**1479.9777**	0.1875
205630_at	CRH	246.941651	**1174.6591**	246.941651	261.625565	195.997718	0.125
205826_at	MYOM2	246.941651	261.625565	87.3070579	233.081881	**1244.5079**	0.125
213921_at	SST	246.941651	415.304698	69.2956577	**2637.0205**	138.591316	0.75
206634_at	SIX3	261.625565	123.470825	**1174.6591**	440	155.563492	0.125
210095_s_at	IGFBP3	261.625565	233.081881	**1396.9129**	138.591316	293.664768	0.125
210831_s_at	PTGER3	277.182631	233.081881	329.627557	97.998859	**1108.7305**	0.125
213791_at	PENK	277.182631	207.652349	261.625565	293.664768	**2489.0159**	0.4375
201667_at	GJA1	293.664768	184.997211	440	130.812783	**1396.9129**	0.125
202410_x_at	IGF2 /// INS-IGF2	311.126984	329.627557	293.664768	**1396.9129**	51.9130872	0.125
212203_x_at	IFITM3	329.627557	146.832384	**739.98885**	92.4986057	**1244.5079**	0.125
218559_s_at	MAFB	349.228231	311.126984	369.994423	73.416192	**1975.5332**	0.25
201976_s_at	MYO10	369.994423	440	329.627557	246.941651	**1244.5079**	0.125
203729_at	EMP3	369.994423	293.664768	440	220	**1396.9129**	0.125
214612_x_at	MAGEA6	369.994423	622.253967	**1108.7305**	130.812783	207.652349	0.125
209685_s_at	PRKCB	391.995436	261.625565	220	587.329536	**1318.5102**	0.125

202431_s_at	MYC	415.304698	184.997211	**880**	123.470825	**1479.9777**	0.1875
204151_x_at	AKR1C1	415.304698	**1396.9129**	493.883301	233.081881	329.627557	0.125
204851_s_at	DCX	415.304698	130.812783	391.995436	**1174.6591**	440	0.125
210302_s_at	MAB21L2	415.304698	**1975.5332**	311.126984	523.251131	164.813779	0.21875
206163_at	MAB21L1	440	**1244.5079**	**1108.7305**	164.813779	69.2956577	0.1875
206450_at	DBH	440	659.255114	**1174.6591**	293.664768	116.54094	0.125
212646_at	RFTN1	440	415.304698	493.883301	**1244.5079**	164.813779	0.125
218831_s_at	FCGRT	440	369.994423	493.883301	130.812783	**1864.655**	0.21875
201596_x_at	KRT18	466.163762	261.625565	659.255114	311.126984	**1661.2188**	0.1875
209942_x_at	MAGEA3	493.883301	830.609395	**1396.9129**	155.563492	293.664768	0.125
213847_at	PRPH	493.883301	**1760**	261.625565	830.609395	293.664768	0.1875
203476_at	TPBG	523.251131	261.625565	1046.50226	233.081881	**1108.7305**	0.125
207173_x_at	CDH11	523.251131	164.813779	622.253967	440	**1396.9129**	0.125
208791_at	CLU	523.251131	**1244.5079**	783.990872	82.4068892	329.627557	0.125
212397_at	RDX	523.251131	369.994423	698.456463	220	**1396.9129**	0.125
214636_at	CALCB	523.251131	**1174.6591**	246.941651	311.126984	880	0.125
218170_at	ISOC1	523.251131	493.883301	**1479.9777**	466.163762	523.251131	0.125
221011_s_at	LBH	523.251131	466.163762	554.365262	466.163762	**1864.655**	0.1875
204602_at	DKK1	554.365262	391.995436	1108.73052	739.988845	69.2956577	0.125
210794_s_at	MEG3	554.365262	880	77.7817459	**1244.5079**	329.627557	0.125
212192_at	KCTD12	554.365262	**1318.5102**	932.327523	51.9130872	329.627557	0.125
218162_at	OLFML3	554.365262	466.163762	622.253967	415.304698	**1396.9129**	0.125
216526_x_at	HLA-C	587.329536	311.126984	1046.50226	195.997718	**1244.5079**	0.125
217979_at	TSPAN13	587.329536	293.664768	440	783.990872	**1396.9129**	0.125
221728_x_at	XIST	587.329536	220	**1318.5102**	246.941651	**1396.9129**	0.1875
202145_at	LY6E	622.253967	349.228231	554.365262	659.255114	**1479.9777**	0.125
209757_s_at	MYCN	622.253967	**1975.5332**	246.941651	**1479.9777**	207.652349	0.25

210839_s_at	ENPP2	622.253967	**1244.5079**	311.126984	**1396.9129**	164.813779	0.1875
212188_at	KCTD12	622.253967	**1396.9129**	1174.65907	103.826174	311.126984	0.1875
218353_at	RGS5	622.253967	**1244.5079**	**2093.0045**	293.664768	220	0.25
201028_s_at	CD99	659.255114	233.081881	622.253967	698.456463	**1318.5102**	0.125
202409_at	IGF2 /// INS-IGF2	659.255114	739.988845	587.329536	**1975.5332**	311.126984	0.1875
209163_at	CYB561	659.255114	349.228231	1174.65907	**1318.5102**	184.997211	0.125
211959_at	IGFBP5	659.255114	**1567.9817**	1046.50226	220	391.995436	0.1875
212741_at	MAOA	659.255114	**1396.9129**	932.327523	440	293.664768	0.125
205113_at	NEFM	698.456463	622.253967	739.988845	**2217.461**	61.7354127	0.25
207076_s_at	ASS1	698.456463	466.163762	73.416192	**1479.9777**	1046.50226	0.1875
207986_x_at	CYB561	698.456463	466.163762	932.327523	**1479.9777**	184.997211	0.125
221805_at	NEFL	698.456463	739.988845	622.253967	1318.51023	30.8677063	0.125
203417_at	MFAP2	739.988845	195.997718	**1479.9777**	587.329536	880	0.125
209755_at	NMNAT2	739.988845	1108.73052	587.329536	932.327523	87.3070579	0.125
213577_at	SQLE	739.988845	622.253967	587.329536	880	**1567.9817**	0.125
215076_s_at	COL3A1	739.988845	698.456463	**1567.9817**	123.470825	739.988845	0.125
217200_x_at	CYB561	739.988845	440	1244.50794	1318.51023	440	0.125
201852_x_at	COL3A1	783.990872	783.990872	1567.98174	65.4063913	739.988845	0.1875
203129_s_at	KIF5C	783.990872	1046.50226	554.365262	1046.50226	43.6535289	0.125
204086_at	PRAME	783.990872	1479.97769	1396.91293	415.304698	440	0.125
211421_s_at	RET	783.990872	698.456463	830.609395	1318.51023	220	0.125
213943_at	TWIST1	783.990872	554.365262	1244.50794	246.941651	1108.73052	0.125
217783_s_at	YPEL5	783.990872	739.988845	830.609395	698.456463	**1661.2188**	0.125
218623_at	HMP19	783.990872	987.766603	622.253967	1318.51023	277.182631	0.125
219449_s_at	TMEM70	783.990872	739.988845	783.990872	739.988845	**2489.0159**	0.25
201590_x_at	ANXA2	830.609395	329.627557	**1760**	369.994423	**2217.461**	0.375
204730_at	RIMS3	830.609395	1567.98174	830.609395	783.990872	207.652349	0.125

209392_at	ENPP2	830.609395	1567.98174	440	1661.21879	138.591316	0.21875
201105_at	LGALS1	880	587.329536	**1975.5332**	311.126984	1244.50794	0.1875
202345_s_at	FABP5	880	146.832384	698.456463	1108.73052	1318.51023	0.125
202465_at	PCOLCE	880	880	**1864.655**	880	466.163762	0.125
203423_at	RBP1	880	82.4068892	1396.91293	**1864.655**	523.251131	0.21875
203999_at	SYT1	880	1661.21879	880	880	554.365262	0.125
207480_s_at	MEIS2	880	1108.73052	1479.97769	659.255114	369.994423	0.125
209071_s_at	RGS5	880	1760	**2349.3181**	415.304698	261.625565	0.375
209560_s_at	DLK1	880	659.255114	1108.73052	**3135.9635**	195.997718	0.875
210427_x_at	ANXA2	880	415.304698	**1864.655**	369.994423	**2349.3181**	0.375
213503_x_at	ANXA2	880	415.304698	1661.21879	277.182631	**2217.461**	0.25
221916_at	NEFL	880	987.766603	739.988845	1661.21879	97.998859	0.1875
201029_s_at	CD99	932.327523	293.664768	880	987.766603	1567.98174	0.125
204260_at	CHGB	932.327523	1318.51023	587.329536	1760	92.4986057	0.1875
209070_s_at	RGS5	932.327523	**1975.5332**	**2637.0205**	440	110	0.75
209197_at	SYT11	932.327523	1318.51023	783.990872	1108.73052	233.081881	0.125
211049_at	TLX2	932.327523	1244.50794	932.327523	932.327523	65.4063913	0.125
220942_x_at	FAM162A	932.327523	830.609395	830.609395	987.766603	1864.65505	0.125
209987_s_at	ASCL1	987.766603	1567.98174	1396.91293	659.255114	82.4068892	0.1875
212552_at	HPCAL1	987.766603	783.990872	1244.50794	**2217.461**	554.365262	0.1875
204035_at	SCG2	1046.50226	1479.97769	698.456463	1479.97769	220	0.1875
204338_s_at	RGS4	1046.50226	1174.65907	1567.98174	880	174.614116	0.125
209988_s_at	ASCL1	1046.50226	1975.53321	1479.97769	698.456463	184.997211	0.21875
211587_x_at	CHRNA3	1046.50226	1108.73052	932.327523	1046.50226	130.812783	0.125
212607_at	AKT3	1046.50226	1396.91293	932.327523	1046.50226	277.182631	0.125
217869_at	HSD17B12	1046.50226	184.997211	783.990872	1318.51023	1567.98174	0.1875
201242_s_at	ATP1B1	1108.73052	1174.65907	1174.65907	987.766603	207.652349	0.125

203662_s_at	TMOD1	1108.73052	1108.73052	1046.50226	1108.73052	116.54094	0.125
204339_s_at	RGS4	1108.73052	1174.65907	1661.21879	932.327523	130.812783	0.1875
204540_at	EEF1A2	1108.73052	1318.51023	987.766603	1567.98174	195.997718	0.125
209841_s_at	LRRN3	1108.73052	1864.65505	1396.91293	880	220	0.1875
210222_s_at	RTN1	1108.73052	1244.50794	987.766603	1174.65907	246.941651	0.125
210950_s_at	FDFT1	1108.73052	1046.50226	1046.50226	1108.73052	2217.46105	0.125
202517_at	CRMP1	1174.65907	1318.51023	987.766603	1244.50794	277.182631	0.125
203000_at	STMN2	1174.65907	880	1864.65505	1479.97769	138.591316	0.21875
203001_s_at	STMN2	1174.65907	830.609395	1975.53321	1567.98174	51.9130872	0.21875
203661_s_at	TMOD1	1174.65907	1318.51023	1108.73052	1244.50794	246.941651	0.125
204913_s_at	SOX11	1174.65907	1864.65505	1479.97769	622.253967	880	0.125
206051_at	ELAVL4	1174.65907	1479.97769	1318.51023	1046.50226	293.664768	0.125
209185_s_at	IRS2	1174.65907	523.251131	1318.51023	987.766603	1864.65505	0.125
209218_at	SQLE	1174.65907	1174.65907	880	1174.65907	1975.53321	0.125
214609_at	PHOX2A	1174.65907	1244.50794	1396.91293	1108.73052	174.614116	0.125
203130_s_at	KIF5C	1244.50794	1661.21879	987.766603	1396.91293	246.941651	0.1875
203414_at	MMD	1244.50794	1661.21879	1244.50794	1244.50794	523.251131	0.125
205311_at	DDC	1244.50794	1244.50794	1244.50794	2093.00452	61.7354127	0.21875
209840_s_at	LRRN3	1244.50794	1760	1567.98174	932.327523	174.614116	0.1875
210221_at	CHRNA3	1244.50794	1567.98174	1174.65907	1244.50794	195.997718	0.125
210547_x_at	ICA1	1244.50794	1174.65907	1318.51023	1244.50794	103.826174	0.125
213932_x_at	HLA-A	1244.50794	880	1567.98174	932.327523	1975.53321	0.125
220138_at	HAND1	1244.50794	1760	1174.65907	1244.50794	329.627557	0.125
201416_at	SOX4	1318.51023	1396.91293	1975.53321	830.609395	1174.65907	0.125
202478_at	TRIB2	1318.51023	1567.98174	622.253967	1567.98174	1108.73052	0.125
204697_s_at	CHGA	1318.51023	1244.50794	1318.51023	2093.00452	146.832384	0.21875
206104_at	ISL1	1318.51023	1396.91293	1760	1174.65907	440	0.125

209710_at	GATA2	1318.51023	1318.51023	1567.98174	1244.50794	440	0.125
219791_s_at	NBLA00301	1318.51023	2217.46105	1864.65505	880	48.9994295	0.375
201272_at	AKR1B1	1396.91293	1479.97769	1760	1318.51023	493.883301	0.125
204915_s_at	SOX11	1396.91293	1975.53321	1661.21879	1108.73052	739.988845	0.125
218820_at	C14orf132	1396.91293	1760	1318.51023	1396.91293	466.163762	0.125
200943_at	HMGN1	1479.97769	1975.53321	987.766603	1760	1174.65907	0.125
201565_s_at	ID2	1479.97769	1479.97769	1567.98174	659.255114	1661.21879	0.125
202391_at	BASP1	1479.97769	1864.65505	1174.65907	1174.65907	2093.00452	0.125
217294_s_at	ENO1	1479.97769	1318.51023	1567.98174	1396.91293	2349.31814	0.125
204337_at	RGS4	1661.21879	1864.65505	2093.00452	1479.97769	220	0.21875
204914_s_at	SOX11	1661.21879	2489.01587	1975.53321	1396.91293	1108.73052	0.1875
201231_s_at	ENO1	1760	1479.97769	1864.65505	1661.21879	2637.02046	0.125
212233_at	MAP1B	1760	2217.46105	1567.98174	1864.65505	698.456463	0.1875
212509_s_at	MXRA7	1760	2489.01587	1661.21879	1760	1108.73052	0.125
218332_at	BEX1	1760	1864.65505	1661.21879	1864.65505	830.609395	0.125
201426_s_at	VIM	1864.65505	2349.31814	2637.02046	1479.97769	1318.51023	0.1875
207009_at	PHOX2B	1864.65505	2637.02046	1760	1864.65505	110	0.4375
209604_s_at	GATA3	1864.65505	2217.46105	2093.00452	1661.21879	369.994423	0.21875
200790_at	ODC1	1975.53321	2093.00452	1864.65505	**4186.009**	1864.65505	0.5
201241_at	DDX1	1975.53321	**4186.009**	1174.65907	3322.43758	1174.65907	1.75
209771_x_at	CD24	1975.53321	2093.00452	1975.53321	1864.65505	739.988845	0.1875
211071_s_at	MLLT11	1975.53321	2637.02046	1864.65505	1975.53321	1396.91293	0.125

205967_at	HIST1H4A /// HIST1H4B /// HIST1H4C /// HIST1H4D /// HIST1H4E /// HIST1H4F /// HIST1H4H /// HIST1H4I /// HIST1H4J /// HIST1H4K /// HIST1H4L /// HIST2H4A /// HIST2H4B /// HIST4H4	2093.00452	1864.65505	1396.91293	3135.96349	2217.46105	0.1875
209118_s_at	TUBA1A	2093.00452	2637.02046	1864.65505	2349.31814	1661.21879	0.125
212826_s_at	SLC25A6	2093.00452	1046.50226	1975.53321	2217.46105	2217.46105	0.125
212085_at	SLC25A6	2217.46105	1046.50226	2217.46105	2217.46105	2217.46105	0.125
216379_x_at	CD24	2217.46105	2489.01587	2217.46105	1975.53321	698.456463	0.21875
208826_x_at	HINT1	2349.31814	2093.00452	1864.65505	2637.02046	3135.96349	0.125
221775_x_at	RPL22	2349.31814	2093.00452	2637.02046	1975.53321	2959.95538	0.125
AFFX-HSAC07/X00351_5_at	ACTB /// ACTB /// LOC100505829	2349.31814	2349.31814	3322.43758	2217.46105	2349.31814	0.125
213187_x_at	FTL	2489.01587	2793.82585	2217.46105	1760	2793.82585	0.125
212788_x_at	FTL	2637.02046	3135.96349	2217.46105	2093.00452	3135.96349	0.125
217733_s_at	TMSB10	2637.02046	2959.95538	2637.02046	1661.21879	2489.01587	0.125
211997_x_at	H3F3A /// H3F3B	2959.95538	3520	2793.82585	3135.96349	2349.31814	0.125
214938_x_at	HMGB1	2959.95538	2959.95538	2093.00452	2959.95538	3135.96349	0.125

AFFX-HSAC07/X00351_M_at	ACTB /// ACTB /// LOC100505829	2959.95538	2959.95538	3729.31009	2793.82585	2793.82585	0.125
200717_x_at	RPL7	3135.96349	3322.43758	2637.02046	2959.95538	3729.31009	0.125
202029_x_at	RPL38	3322.43758	3322.43758	3135.96349	3520	2489.01587	0.125
207783_x_at	HUWE1	3322.43758	3520	3322.43758	2093.00452	3135.96349	0.1875

Supplementary Table S3.

Musical interpretation of Ewing sarcoma-specific probe sets from "neuroblastoma" cell lines. DNA microarray data from a panel of Ewing sarcoma biopsies and neuroblastoma biopsies (GSE1825) were used for the identification of Ewing sarcoma-specific probe sets. To this end, MAFilter was used for filtering probe sets with maximal ratios between the median in Ewing sarcomas and the 85th percentile in neuroblastoma samples. All probe sets with a fold change > 3 were considered Ewing sarcoma-specific. DNA microarray data from these 376 probe sets in an independent data set of 4 cell lines that were initially established as neuroblastoma cell lines (GSE1824) were transformed into melodies by using the same parameters as in Supplementary Table S2.

**Probe Set ID**	**Gene symbol**	**Median**	**CHP-126**	**SH-SY5Y**	**SiMa**	**SK-N-MC**	**Duration**
207397_s_at	HOXD13	43.65352893	46.24930284	41.20344461	36.70809599	739.9888454	0.125
205478_at	PPP1R1A	69.29565774	51.9130872	73.41619198	61.73541266	1661.21879	0.21875
209844_at	HOXB13	73.41619198	73.41619198	69.29565774	77.78174593	783.990872	0.125
200951_s_at	CCND2	82.40688923	138.5913155	48.9994295	51.9130872	932.327523	0.125
209656_s_at	TMEM47	82.40688923	58.27047019	116.5409404	46.24930284	1567.981744	0.21875
219090_at	SLC24A3	82.40688923	73.41619198	69.29565774	97.998859	830.6093952	0.125
204456_s_at	GAS1	87.30705786	82.40688923	92.49860568	82.40688923	880	0.125
205872_x_at	LOC101060353 /// PDE4DIP	87.30705786	77.78174593	92.49860568	82.40688923	659.2551138	0.125
206114_at	EPHA4	87.30705786	69.29565774	77.78174593	97.998859	783.990872	0.125
206866_at	CDH4	92.49860568	130.8127827	69.29565774	69.29565774	466.1637615	0.125
209655_s_at	TMEM47	92.49860568	97.998859	82.40688923	87.30705786	659.2551138	0.125
219825_at	CYP26B1	92.49860568	73.41619198	116.5409404	69.29565774	830.6093952	0.125
213395_at	MLC1	97.998859	164.8137785	58.27047019	58.27047019	783.990872	0.125
206326_at	GRP	103.8261744	110	103.8261744	103.8261744	2637.020455	0.75
212909_at	LYPD1	103.8261744	116.5409404	97.998859	77.78174593	1396.912926	0.1875
203324_s_at	CAV2	116.5409404	61.73541266	220	32.70319566	987.7666025	0.125
205306_x_at	KMO	116.5409404	87.30705786	103.8261744	130.8127827	1479.977691	0.21875
219686_at	STK32B	116.5409404	130.8127827	103.8261744	110	739.9888454	0.125
207373_at	HOXD10	123.4708253	69.29565774	65.40639133	233.0818808	493.8833013	0.125
202747_s_at	ITM2A	130.8127827	138.5913155	123.4708253	123.4708253	2793.825851	0.875
217303_s_at	ADRB3	130.8127827	87.30705786	184.9972114	82.40688923	523.2511306	0.125
219360_s_at	TRPM4	130.8127827	155.5634919	110	61.73541266	1174.659072	0.1875
205066_s_at	ENPP1	138.5913155	184.9972114	110	92.49860568	587.3295358	0.125
206812_at	ADRB3	138.5913155	155.5634919	130.8127827	92.49860568	1046.502261	0.125

206915_at	NKX2-2	138.5913155	65.40639133	92.49860568	207.6523488	1479.977691	0.21875
208060_at	PAX7	138.5913155	164.8137785	116.5409404	110	523.2511306	0.125
207016_s_at	ALDH1A2	146.832384	69.29565774	277.182631	493.8833013	73.41619198	0.125
212097_at	CAV1	146.832384	55	349.2282314	61.73541266	2959.955382	1
214460_at	LSAMP	146.832384	116.5409404	195.997718	92.49860568	783.990872	0.125
205307_s_at	KMO	155.5634919	174.6141157	146.832384	138.5913155	587.3295358	0.125
221606_s_at	HMGN5	155.5634919	174.6141157	77.78174593	146.832384	659.2551138	0.125
221928_at	ACACB	155.5634919	87.30705786	233.0818808	103.8261744	493.8833013	0.125
207398_at	HOXD13	164.8137785	329.6275569	92.49860568	87.30705786	783.990872	0.125
212236_x_at	JUP /// KRT17	164.8137785	164.8137785	184.9972114	123.4708253	622.2539674	0.125
208334_at	NDST4	174.6141157	146.832384	87.30705786	207.6523488	739.9888454	0.125
205440_s_at	NPY1R	184.9972114	349.2282314	110	69.29565774	1108.730524	0.125
205827_at	CCK	184.9972114	261.6255653	130.8127827	43.65352893	3322.437581	1.75
209129_at	TRIP6	184.9972114	174.6141157	195.997718	87.30705786	1396.912926	0.1875
217549_at	---	184.9972114	195.997718	97.998859	195.997718	698.4564629	0.125
57588_at	SLC24A3	184.9972114	77.78174593	184.9972114	174.6141157	493.8833013	0.125
218182_s_at	CLDN1	195.997718	87.30705786	146.832384	261.6255653	698.4564629	0.125
221215_s_at	RIPK4	195.997718	130.8127827	261.6255653	138.5913155	880	0.125
202746_at	ITM2A	207.6523488	233.0818808	195.997718	110	2349.318143	0.4375
205227_at	IL1RAP	207.6523488	130.8127827	184.9972114	246.9416506	1760	0.21875
209734_at	NCKAP1L	207.6523488	174.6141157	277.182631	155.5634919	1975.533205	0.25
204229_at	SLC17A7	220	311.1269837	174.6141157	130.8127827	622.2539674	0.125
206645_s_at	NR0B1	220	329.6275569	82.40688923	164.8137785	1174.659072	0.1875
219937_at	TRHDE	220	82.40688923	164.8137785	277.182631	622.2539674	0.125
203914_x_at	HPGD	233.0818808	587.3295358	220	110	233.0818808	0.125
204198_s_at	RUNX3	233.0818808	277.182631	207.6523488	73.41619198	1479.977691	0.1875
204951_at	RHOH	233.0818808	277.182631	123.4708253	207.6523488	987.7666025	0.125
206935_at	PCDH8	233.0818808	739.9888454	146.832384	164.8137785	329.6275569	0.125
211138_s_at	KMO	233.0818808	349.2282314	164.8137785	116.5409404	1567.981744	0.21875
204197_s_at	RUNX3	246.9416506	184.9972114	82.40688923	349.2282314	1244.507935	0.1875
210375_at	PTGER3	246.9416506	207.6523488	293.6647679	138.5913155	659.2551138	0.125
219908_at	DKK2	246.9416506	207.6523488	311.1269837	138.5913155	587.3295358	0.125
207400_at	NPY5R	261.6255653	369.9944227	155.5634919	207.6523488	587.3295358	0.125
209791_at	PADI2	261.6255653	184.9972114	138.5913155	391.995436	783.990872	0.125
214604_at	HOXD11	261.6255653	369.9944227	207.6523488	174.6141157	830.6093952	0.125
219572_at	CADPS2	261.6255653	293.6647679	195.997718	261.6255653	1760	0.21875

203065_s_at	CAV1	277.182631	110	440	164.8137785	3135.963488	1.5
206002_at	GPR64	277.182631	293.6647679	261.6255653	55	659.2551138	0.125
206025_s_at	TNFAIP6	277.182631	329.6275569	233.0818808	97.998859	987.7666025	0.125
216222_s_at	MYO10	277.182631	103.8261744	261.6255653	261.6255653	783.990872	0.125
200953_s_at	CCND2	293.6647679	391.995436	195.997718	246.9416506	2217.461048	0.375
206026_s_at	TNFAIP6	293.6647679	311.1269837	293.6647679	184.9972114	880	0.125
201331_s_at	STAT6	311.1269837	523.2511306	146.832384	195.997718	1108.730524	0.125
203824_at	TSPAN8	311.1269837	92.49860568	1108.730524	220	415.3046976	0.125
205542_at	STEAP1	311.1269837	82.40688923	311.1269837	329.6275569	2349.318143	0.4375
205990_s_at	WNT5A	311.1269837	233.0818808	440	195.997718	783.990872	0.125
206159_at	GDF10	311.1269837	349.2282314	277.182631	103.8261744	554.365262	0.125
213714_at	CACNB2	311.1269837	61.73541266	277.182631	329.6275569	493.8833013	0.125
43427_at	ACACB	311.1269837	293.6647679	349.2282314	155.5634919	587.3295358	0.125
201981_at	PAPPA	329.6275569	195.997718	174.6141157	587.3295358	1046.502261	0.125
203646_at	FDX1	329.6275569	207.6523488	369.9944227	277.182631	932.327523	0.125
205932_s_at	MSX1	329.6275569	554.365262	207.6523488	82.40688923	1396.912926	0.1875
207957_s_at	PRKCB	329.6275569	92.49860568	207.6523488	523.2511306	1244.507935	0.1875
204230_s_at	SLC17A7	349.2282314	391.995436	329.6275569	293.6647679	739.9888454	0.125
204779_s_at	HOXB7	349.2282314	349.2282314	329.6275569	311.1269837	1046.502261	0.125
212920_at	REST	349.2282314	369.9944227	440	38.89087297	369.9944227	0.125
218959_at	HOXC10	349.2282314	440	293.6647679	261.6255653	932.327523	0.125
61734_at	RCN3	349.2282314	349.2282314	349.2282314	103.8261744	739.9888454	0.125
206745_at	HOXC11	369.9944227	311.1269837	261.6255653	493.8833013	1046.502261	0.125
210374_x_at	PTGER3	369.9944227	349.2282314	415.3046976	261.6255653	1396.912926	0.1875
210831_s_at	PTGER3	369.9944227	329.6275569	415.3046976	116.5409404	1661.21879	0.21875
213552_at	GLCE	369.9944227	523.2511306	261.6255653	174.6141157	554.365262	0.125
202709_at	FMOD	391.995436	880	311.1269837	415.3046976	391.995436	0.125
207172_s_at	CDH11	391.995436	82.40688923	440	349.2282314	1108.730524	0.125
219837_s_at	CYTL1	391.995436	61.73541266	440	329.6275569	466.1637615	0.125
201982_s_at	PAPPA	415.3046976	369.9944227	220	466.1637615	783.990872	0.125
215695_s_at	GYG2	415.3046976	174.6141157	293.6647679	554.365262	698.4564629	0.125
221679_s_at	ABHD6	415.3046976	466.1637615	293.6647679	391.995436	739.9888454	0.125
45288_at	ABHD6	415.3046976	415.3046976	293.6647679	493.8833013	1174.659072	0.125
204457_s_at	GAS1	440	466.1637615	415.3046976	233.0818808	1661.21879	0.1875
215322_at	LONRF1	440	523.2511306	391.995436	415.3046976	1760	0.1875
219427_at	FAT4	440	554.365262	391.995436	233.0818808	1318.510228	0.125

219976_at	HOOK1	440	415.3046976	349.2282314	523.2511306	1244.507935	0.125
49452_at	ACACB	440	415.3046976	493.8833013	164.8137785	698.4564629	0.125
210964_s_at	GYG2	466.1637615	440	369.9944227	554.365262	1174.659072	0.125
201976_s_at	MYO10	493.8833013	659.2551138	415.3046976	329.6275569	1864.655046	0.21875
204867_at	GCHFR	493.8833013	1396.912926	311.1269837	329.6275569	698.4564629	0.1875
205375_at	MDFI	493.8833013	261.6255653	622.2539674	391.995436	1244.507935	0.125
218847_at	IGF2BP2	493.8833013	587.3295358	311.1269837	440	1661.21879	0.1875
202720_at	TES	523.2511306	391.995436	698.4564629	311.1269837	880	0.125
204201_s_at	PTPN13	523.2511306	830.6093952	329.6275569	369.9944227	739.9888454	0.125
205103_at	C1orf61	523.2511306	587.3295358	493.8833013	587.3295358	184.9972114	0.125
206481_s_at	LDB2	523.2511306	659.2551138	311.1269837	466.1637615	1108.730524	0.125
209685_s_at	PRKCB	523.2511306	369.9944227	277.182631	830.6093952	1975.533205	0.21875
210963_s_at	GYG2	523.2511306	466.1637615	311.1269837	659.2551138	1174.659072	0.125
212415_at	41888	523.2511306	932.327523	329.6275569	830.6093952	207.6523488	0.125
221703_at	BRIP1	523.2511306	830.6093952	440	587.3295358	415.3046976	0.125
202431_s_at	MYC	554.365262	233.0818808	1244.507935	146.832384	2217.461048	0.375
205345_at	BARD1	554.365262	698.4564629	466.1637615	466.1637615	880	0.125
212045_at	GLG1	554.365262	554.365262	493.8833013	622.2539674	932.327523	0.125
219528_s_at	BCL11B	554.365262	659.2551138	184.9972114	493.8833013	1661.21879	0.1875
204424_s_at	LMO3	587.3295358	783.990872	783.990872	369.9944227	523.2511306	0.125
205604_at	HOXD9	587.3295358	659.2551138	554.365262	1174.659072	554.365262	0.125
218831_s_at	FCGRT	587.3295358	554.365262	659.2551138	155.5634919	2959.955382	0.75
221552_at	ABHD6	587.3295358	659.2551138	440	587.3295358	1396.912926	0.125
206565_x_at	SMA4	622.2539674	440	523.2511306	739.9888454	987.7666025	0.125
215071_s_at	HIST1H2AC	622.2539674	783.990872	622.2539674	261.6255653	622.2539674	0.125
222067_x_at	HIST1H2BD	622.2539674	1046.502261	659.2551138	587.3295358	523.2511306	0.125
201260_s_at	SYPL1	659.2551138	783.990872	587.3295358	466.1637615	1046.502261	0.125
218665_at	FZD4	659.2551138	698.4564629	698.4564629	369.9944227	739.9888454	0.125
205097_at	SLC26A2	698.4564629	830.6093952	554.365262	622.2539674	987.7666025	0.125
205888_s_at	JAKMIP2	698.4564629	783.990872	523.2511306	739.9888454	1318.510228	0.125
209628_at	NXT2	698.4564629	349.2282314	739.9888454	659.2551138	739.9888454	0.125
221011_s_at	LBH	698.4564629	739.9888454	739.9888454	659.2551138	3135.963488	0.5
222209_s_at	TMEM135	698.4564629	932.327523	493.8833013	587.3295358	932.327523	0.125
205046_at	CENPE	739.9888454	880	554.365262	783.990872	1108.730524	0.125
207173_x_at	CDH11	739.9888454	220	830.6093952	622.2539674	2093.004522	0.21875
209294_x_at	TNFRSF10B	739.9888454	932.327523	493.8833013	698.4564629	830.6093952	0.125

214636_at	CALCB	739.9888454	1975.533205	293.6647679	415.3046976	1244.507935	0.21875
219555_s_at	CENPN	739.9888454	932.327523	587.3295358	932.327523	659.2551138	0.125
202419_at	KDSR	783.990872	932.327523	659.2551138	659.2551138	1864.655046	0.1875
202145_at	LY6E	830.6093952	493.8833013	739.9888454	932.327523	2349.318143	0.25
203790_s_at	HRSP12	830.6093952	830.6093952	698.4564629	932.327523	1760	0.125
203856_at	VRK1	830.6093952	932.327523	523.2511306	830.6093952	1046.502261	0.125
207563_s_at	OGT	830.6093952	1108.730524	622.2539674	698.4564629	1046.502261	0.125
209340_at	UAP1	830.6093952	880	783.990872	783.990872	2217.461048	0.21875
212612_at	RCOR1	830.6093952	987.7666025	659.2551138	739.9888454	1396.912926	0.125
201028_s_at	CD99	932.327523	329.6275569	830.6093952	987.7666025	1975.533205	0.21875
204822_at	TTK	932.327523	880	739.9888454	1046.502261	1396.912926	0.125
208951_at	ALDH7A1	932.327523	932.327523	622.2539674	1046.502261	1318.510228	0.125
212307_s_at	OGT	932.327523	1318.510228	698.4564629	830.6093952	1108.730524	0.125
213931_at	ID2 /// ID2B	932.327523	1174.659072	1318.510228	493.8833013	880	0.125
214290_s_at	HIST2H2AA3 /// HIST2H2AA4	932.327523	830.6093952	1244.507935	987.7666025	880	0.125
218782_s_at	ATAD2	932.327523	1046.502261	523.2511306	932.327523	1244.507935	0.125
201648_at	JAK1	1046.502261	1046.502261	1108.730524	880	2349.318143	0.21875
202870_s_at	CDC20	1046.502261	987.7666025	932.327523	1108.730524	1567.981744	0.125
203764_at	DLGAP5	1046.502261	932.327523	830.6093952	1244.507935	1479.977691	0.125
206316_s_at	KNTC1	1046.502261	1318.510228	698.4564629	1046.502261	1108.730524	0.125
208950_s_at	ALDH7A1	1046.502261	1046.502261	554.365262	1174.659072	1396.912926	0.125
209714_s_at	CDKN3	1046.502261	1046.502261	783.990872	1174.659072	1244.507935	0.125
212501_at	CEBPB	1046.502261	1174.659072	587.3295358	1108.730524	2217.461048	0.21875
201566_x_at	ID2	1108.730524	1318.510228	1396.912926	349.2282314	1046.502261	0.125
213811_x_at	TCF3	1108.730524	1244.507935	1046.502261	1108.730524	1479.977691	0.125
211953_s_at	IPO5	1174.659072	1318.510228	987.7666025	1174.659072	1567.981744	0.125
201562_s_at	SORD	1244.507935	1174.659072	880	1396.912926	1567.981744	0.125
201666_at	TIMP1	1244.507935	783.990872	1479.977691	1046.502261	1396.912926	0.125
203213_at	CDK1	1244.507935	1244.507935	932.327523	1396.912926	1396.912926	0.125
205176_s_at	ITGB3BP	1244.507935	1479.977691	932.327523	1479.977691	1174.659072	0.125
212713_at	MFAP4	1244.507935	1479.977691	1318.510228	880	1318.510228	0.125
203418_at	CCNA2	1318.510228	1318.510228	880	1396.912926	1567.981744	0.125
208796_s_at	CCNG1	1318.510228	1760	1244.507935	1174.659072	1244.507935	0.125
220942_x_at	FAM162A	1318.510228	1396.912926	1108.730524	1479.977691	2959.955382	0.25
201029_s_at	CD99	1396.912926	415.3046976	1244.507935	1479.977691	2489.01587	0.25

202554_s_at	GSTM3	1396.912926	1396.912926	1108.730524	1567.981744	2349.318143	0.1875
219918_s_at	ASPM	1396.912926	1760	1244.507935	1174.659072	1479.977691	0.125
201291_s_at	TOP2A	1479.977691	1318.510228	987.7666025	1760	2349.318143	0.1875
201292_at	TOP2A	1479.977691	1567.981744	1244.507935	1661.21879	1864.655046	0.125
203625_x_at	SKP2	1479.977691	1479.977691	987.7666025	1760	1567.981744	0.125
218883_s_at	MLF1IP	1479.977691	1864.655046	1174.659072	1661.21879	1396.912926	0.125
220948_s_at	ATP1A1	1479.977691	1760	1318.510228	1396.912926	2959.955382	0.21875
201397_at	PHGDH	1567.981744	1479.977691	1396.912926	1975.533205	1661.21879	0.125
214730_s_at	GLG1	1567.981744	1479.977691	1479.977691	1567.981744	2489.01587	0.1875
211955_at	IPO5	1661.21879	1864.655046	1244.507935	1661.21879	2217.461048	0.125
201074_at	SMARCC1	1760	1975.533205	1396.912926	1864.655046	1760	0.125
201563_at	SORD	1760	1760	1318.510228	1975.533205	2093.004522	0.125
211954_s_at	IPO5	1760	2093.004522	1396.912926	1864.655046	2489.01587	0.125
204386_s_at	MRP63	1864.655046	1975.533205	1396.912926	2093.004522	2349.318143	0.125
208771_s_at	LTA4H	1864.655046	2349.318143	1760	1567.981744	1760	0.125
201890_at	RRM2	2093.004522	2489.01587	1479.977691	2349.318143	1975.533205	0.125
203832_at	SNRPF	2093.004522	2489.01587	1174.659072	2093.004522	2637.020455	0.1875
209773_s_at	RRM2	2217.461048	2637.020455	1661.21879	2217.461048	2349.318143	0.125
201565_s_at	ID2	2349.318143	2637.020455	2217.461048	932.327523	2637.020455	0.21875
212739_s_at	NME4	2349.318143	2489.01587	2093.004522	2489.01587	2793.825851	0.125
221923_s_at	NPM1	2349.318143	2793.825851	1661.21879	2349.318143	3322.437581	0.21875
203554_x_at	PTTG1	2489.01587	2793.825851	2093.004522	2959.955382	2489.01587	0.125
200853_at	H2AFZ	2637.020455	3135.963488	1975.533205	2793.825851	2637.020455	0.125
202503_s_at	KIAA0101	2959.955382	3322.437581	1975.533205	2793.825851	3135.963488	0.1875
221691_x_at	NPM1	3322.437581	4186.009045	2489.01587	3322.437581	3322.437581	0.21875

Supplementary Table S4.

Frequencies of the musically interpreted microarray data from HL cell lines and normal B cells (exon level). Affymetrix Human Exon 1.0ST microarray data (extended exon level) from 3 Hodgkin's lymphoma cell lines and three CD19-positive B cell samples (from GSE20200) were transformed into melodies by using the following parameters: minimal frequency: 27.5; number of different frequencies: 88 (keys); number of tone steps per octave: 12; minimal duration: 1/8; number of tones: 288. Presented are the frequencies of the individual samples and the frequency of the median signal intensity as well as the duration of the filtered 288 probe sets.

Probe Set ID	Symbol	Median	CD19a	CD19b	CD19c	HDLM-2	L-540	L-428	Duration
4032703	---	46.24930284	46.24930284	43.65352893	43.65352893	36.70809599	1975.533205	46.24930284	0.125
2876713	IL9	73.41619198	73.41619198	73.41619198	69.29565774	2217.461048	73.41619198	138.5913155	0.1875
3168076	CA9	97.998859	77.78174593	123.4708253	116.5409404	2349.318143	55	77.78174593	0.21875
2903233	---	110	61.73541266	116.5409404	69.29565774	97.998859	783.990872	2217.461048	0.1875
2876712	IL9	146.832384	207.6523488	130.8127827	130.8127827	2959.955382	155.5634919	130.8127827	0.875
2633194	GPR15	164.8137785	195.997718	87.30705786	130.8127827	123.4708253	2349.318143	554.365262	0.21875
2633195	---	164.8137785	164.8137785	123.4708253	155.5634919	82.40688923	2093.004522	587.3295358	0.125
2616135	CCR4	174.6141157	195.997718	146.832384	116.5409404	1567.981744	82.40688923	2349.318143	0.25
2876714	IL9	195.997718	174.6141157	174.6141157	184.9972114	2959.955382	207.6523488	207.6523488	0.75
2752746	NEIL3	246.9416506	155.5634919	123.4708253	110	391.995436	349.2282314	2217.461048	0.125
3470535	SELPLG	293.6647679	195.997718	261.6255653	246.9416506	1318.510228	2349.318143	311.1269837	0.1875
2468704	ID2	311.1269837	92.49860568	130.8127827	130.8127827	2093.004522	1567.981744	698.4564629	0.1875
3959992	IL2RB	311.1269837	97.998859	138.5913155	164.8137785	1396.912926	2349.318143	554.365262	0.21875
2351088	CSF1	329.6275569	277.182631	195.997718	261.6255653	880	369.9944227	2217.461048	0.125
2988745	FSCN1	329.6275569	138.5913155	207.6523488	246.9416506	880	415.3046976	2217.461048	0.125
3312516	MKI67	329.6275569	82.40688923	207.6523488	55	987.7666025	493.8833013	2093.004522	0.125
3470532	SELPLG	329.6275569	233.0818808	220	233.0818808	1318.510228	2793.825851	440	0.4375
3756225	TOP2A	329.6275569	207.6523488	184.9972114	87.30705786	493.8833013	698.4564629	2489.01587	0.21875
2988738	FSCN1	391.995436	146.832384	329.6275569	195.997718	1396.912926	493.8833013	2349.318143	0.1875
3484073	ALOX5AP	391.995436	329.6275569	466.1637615	329.6275569	2349.318143	659.2551138	311.1269837	0.125
3761465	HOXB9	391.995436	97.998859	73.41619198	155.5634919	1396.912926	2093.004522	987.7666025	0.1875
3417063	HSPD1	415.3046976	415.3046976	329.6275569	369.9944227	369.9944227	1046.502261	2637.020455	0.21875
2838615	CCNG1	440	195.997718	233.0818808	184.9972114	1244.507935	2489.01587	830.6093952	0.21875
2988740	FSCN1	440	69.29565774	233.0818808	123.4708253	1396.912926	783.990872	2349.318143	0.21875

3595984	CCNB2	440	164.8137785	207.6523488	164.8137785	1174.659072	880	2489.01587	0.21875
2988743	FSCN1	466.1637615	246.9416506	369.9944227	311.1269837	932.327523	554.365262	2349.318143	0.125
3595268	LOC283663	466.1637615	1760	1567.981744	1318.510228	77.78174593	103.8261744	146.832384	0.125
3484079	ALOX5AP	493.8833013	369.9944227	554.365262	415.3046976	2489.01587	783.990872	391.995436	0.125
2976378	PERP	523.2511306	277.182631	174.6141157	195.997718	932.327523	1567.981744	2093.004522	0.125
3582186	IGHG1	523.2511306	1864.655046	1567.981744	1318.510228	77.78174593	174.6141157	65.40639133	0.125
3197340	---	523.2511306	329.6275569	233.0818808	195.997718	2489.01587	1318.510228	830.6093952	0.21875
3455395	---	523.2511306	1864.655046	2093.004522	1244.507935	97.998859	146.832384	195.997718	0.21875
4042535	---	523.2511306	1244.507935	1760	1975.533205	184.9972114	58.27047019	220	0.1875
2365142	MGST3	554.365262	311.1269837	349.2282314	233.0818808	2349.318143	1174.659072	880	0.125
2593741	HSPD1	554.365262	466.1637615	349.2282314	369.9944227	622.2539674	1174.659072	2637.020455	0.21875
3285837	---	554.365262	987.7666025	1396.912926	1975.533205	55	92.49860568	293.6647679	0.125
3315681	IFITM1	554.365262	349.2282314	349.2282314	293.6647679	2349.318143	2217.461048	880	0.25
3503756	TUBA3C	554.365262	195.997718	87.30705786	220	1244.507935	1661.21879	1975.533205	0.1875
3512954	KIAA0226L	554.365262	1975.533205	1318.510228	1479.977691	233.0818808	123.4708253	69.29565774	0.125
3645643	IL32	554.365262	293.6647679	349.2282314	195.997718	1479.977691	880	2217.461048	0.125
4044836	---	554.365262	1046.502261	1661.21879	1975.533205	155.5634919	277.182631	184.9972114	0.125
3090256	---	554.365262	1760	1567.981744	1396.912926	195.997718	73.41619198	155.5634919	0.125
3636534	HDGFRP3	587.3295358	440	233.0818808	246.9416506	1864.655046	739.9888454	2217.461048	0.1875
3938664	---	587.3295358	2217.461048	2093.004522	1864.655046	110	146.832384	164.8137785	0.4375
4044844	---	587.3295358	1244.507935	1864.655046	2217.461048	195.997718	174.6141157	246.9416506	0.21875
2578042	CXCR4	622.2539674	2217.461048	1396.912926	1318.510228	155.5634919	195.997718	277.182631	0.1875
2679302	ID2	622.2539674	261.6255653	466.1637615	587.3295358	2637.020455	2217.461048	622.2539674	0.4375
2927532	TNFAIP3	622.2539674	1046.502261	587.3295358	391.995436	622.2539674	493.8833013	2637.020455	0.1875
3174850	ANXA1	622.2539674	277.182631	246.9416506	220	1396.912926	2637.020455	1318.510228	0.25
3285860	---	622.2539674	1046.502261	1479.977691	1975.533205	36.70809599	51.9130872	349.2282314	0.125
3597724	---	622.2539674	659.2551138	293.6647679	523.2511306	1864.655046	2093.004522	277.182631	0.125
3285861	---	659.2551138	1046.502261	1567.981744	1864.655046	48.9994295	41.20344461	391.995436	0.125
3938653	IGLL5	659.2551138	2217.461048	2093.004522	1975.533205	146.832384	207.6523488	164.8137785	0.4375
3227201	---	659.2551138	880	739.9888454	587.3295358	349.2282314	329.6275569	2489.01587	0.125
2447075	GLUL	698.4564629	622.2539674	739.9888454	554.365262	2217.461048	1975.533205	174.6141157	0.1875
2563796	---	698.4564629	1864.655046	1864.655046	1396.912926	293.6647679	116.5409404	155.5634919	0.1875
3014739	ARPC1B	698.4564629	369.9944227	415.3046976	233.0818808	1046.502261	1567.981744	2637.020455	0.21875
3260603	SCD	698.4564629	164.8137785	369.9944227	391.995436	1567.981744	1174.659072	2217.461048	0.125
3285839	---	698.4564629	1174.659072	1567.981744	1975.533205	55	61.73541266	391.995436	0.125
3484084	ALOX5AP	698.4564629	587.3295358	783.990872	554.365262	3135.963488	987.7666025	493.8833013	0.4375

3938665	IGLL5	698.4564629	2093.004522	2093.004522	1975.533205	246.9416506	184.9972114	233.0818808	0.375
3962282	OLA1	698.4564629	622.2539674	659.2551138	587.3295358	698.4564629	1318.510228	2637.020455	0.125
4042463	---	698.4564629	1318.510228	1975.533205	2217.461048	233.0818808	349.2282314	293.6647679	0.21875
3091158	---	698.4564629	277.182631	523.2511306	369.9944227	1479.977691	880	2349.318143	0.125
4044869	---	698.4564629	1318.510228	2093.004522	2217.461048	261.6255653	138.5913155	349.2282314	0.25
3014738	ARPC1B	739.9888454	466.1637615	369.9944227	329.6275569	1108.730524	1661.21879	2489.01587	0.1875
3060316	SRI	739.9888454	466.1637615	349.2282314	369.9944227	1046.502261	1396.912926	2489.01587	0.1875
3948008	SAMM50	739.9888454	880	587.3295358	554.365262	587.3295358	830.6093952	2637.020455	0.125
3582276	---	739.9888454	2217.461048	1864.655046	1567.981744	277.182631	311.1269837	123.4708253	0.21875
2365149	MGST3	783.990872	311.1269837	391.995436	220	2793.825851	1479.977691	1567.981744	0.375
2895894	CD83	783.990872	2637.020455	1174.659072	1318.510228	195.997718	65.40639133	523.2511306	0.25
2899172	HIST1H1E	783.990872	554.365262	622.2539674	440	2489.01587	2217.461048	1046.502261	0.21875
3060324	SRI	783.990872	554.365262	523.2511306	415.3046976	1046.502261	1567.981744	2637.020455	0.1875
3315685	IFITM1	783.990872	415.3046976	233.0818808	246.9416506	2217.461048	2217.461048	1396.912926	0.25
3582308	IGHD	783.990872	1975.533205	1567.981744	1318.510228	261.6255653	391.995436	97.998859	0.125
3834513	CD79A	783.990872	2217.461048	1479.977691	1479.977691	369.9944227	391.995436	207.6523488	0.125
3894615	FKBP1A	783.990872	622.2539674	466.1637615	523.2511306	987.7666025	1661.21879	2489.01587	0.125
3938637	IGLL5	783.990872	2217.461048	2093.004522	1864.655046	195.997718	293.6647679	207.6523488	0.375
3938652	---	783.990872	2093.004522	1975.533205	1864.655046	311.1269837	246.9416506	146.832384	0.25
2838619	CCNG1	830.6093952	493.8833013	493.8833013	415.3046976	1318.510228	2349.318143	1864.655046	0.125
3938644	---	830.6093952	1864.655046	1864.655046	1479.977691	415.3046976	369.9944227	195.997718	0.125
4000973	RBBP7	830.6093952	1046.502261	830.6093952	783.990872	739.9888454	523.2511306	2793.825851	0.1875
4013160	LDHB	830.6093952	523.2511306	659.2551138	932.327523	466.1637615	1479.977691	3135.963488	0.4375
2726595	---	830.6093952	659.2551138	2093.004522	1864.655046	233.0818808	41.20344461	987.7666025	0.1875
2726609	---	830.6093952	659.2551138	1864.655046	1975.533205	184.9972114	82.40688923	987.7666025	0.125
2468699	ID2	880	174.6141157	523.2511306	830.6093952	2793.825851	2489.01587	880	0.5
2895892	CD83	880	2637.020455	1396.912926	1567.981744	391.995436	61.73541266	554.365262	0.25
3093276	SNORD13	880	466.1637615	554.365262	659.2551138	2793.825851	2489.01587	1108.730524	0.4375
3285821	---	880	1396.912926	1975.533205	1975.533205	110	41.20344461	523.2511306	0.21875
3446871	LDHB	880	587.3295358	739.9888454	987.7666025	587.3295358	1567.981744	3135.963488	0.375
2578041	CXCR4	932.327523	2793.825851	1864.655046	1760	195.997718	277.182631	466.1637615	0.5
3446892	LDHB	932.327523	698.4564629	880	932.327523	466.1637615	1479.977691	3135.963488	0.375
3766907	DDX5	932.327523	2217.461048	1244.507935	1244.507935	116.5409404	207.6523488	659.2551138	0.125
3938680	---	932.327523	2793.825851	2489.01587	2349.318143	329.6275569	174.6141157	174.6141157	1.75
2888713	---	932.327523	554.365262	466.1637615	369.9944227	2217.461048	1479.977691	1975.533205	0.125
2726608	---	987.7666025	932.327523	2217.461048	2093.004522	220	155.5634919	1046.502261	0.21875

2927530	TNFAIP3	987.7666025	987.7666025	659.2551138	440	987.7666025	932.327523	2793.825851	0.1875
3944892	LGALS1	987.7666025	587.3295358	739.9888454	698.4564629	2489.01587	2489.01587	1318.510228	0.21875
2726589	---	987.7666025	932.327523	2093.004522	1864.655046	246.9416506	92.49860568	1046.502261	0.125
2888711	---	987.7666025	523.2511306	440	391.995436	2349.318143	1661.21879	1975.533205	0.1875
2404162	LAPTM5	1046.502261	2093.004522	1864.655046	1479.977691	659.2551138	155.5634919	391.995436	0.125
2469273	RRM2	1046.502261	130.8127827	659.2551138	164.8137785	1567.981744	1760	2349.318143	0.21875
2563792	---	1046.502261	2217.461048	1760	1760	587.3295358	97.998859	138.5913155	0.21875
2587634	OLA1	1046.502261	880	783.990872	830.6093952	1108.730524	1567.981744	3135.963488	0.21875
3014736	ARPC1B	1046.502261	622.2539674	659.2551138	415.3046976	1479.977691	1975.533205	2793.825851	0.25
3446878	LDHB	1046.502261	698.4564629	987.7666025	1046.502261	493.8833013	1174.659072	3322.437581	0.4375
3446886	LDHB	1046.502261	783.990872	1046.502261	987.7666025	587.3295358	1396.912926	2793.825851	0.125
3476099	CDK2AP1	1046.502261	659.2551138	466.1637615	466.1637615	1661.21879	1567.981744	2489.01587	0.125
3666757	CYB5B	1046.502261	1318.510228	1046.502261	880	880	1046.502261	2959.955382	0.125
3790497	SEC11C	1046.502261	1318.510228	587.3295358	987.7666025	987.7666025	880	3322.437581	0.375
4000968	RBBP7	1046.502261	1244.507935	987.7666025	932.327523	830.6093952	1046.502261	2959.955382	0.125
2404169	LAPTM5	1108.730524	2349.318143	1661.21879	1396.912926	739.9888454	261.6255653	440	0.125
2491297	TMSB10	1108.730524	659.2551138	987.7666025	659.2551138	2489.01587	2637.020455	1318.510228	0.21875
2891348	IRF4	1108.730524	698.4564629	622.2539674	587.3295358	1661.21879	2093.004522	2637.020455	0.1875
2891356	IRF4	1108.730524	830.6093952	622.2539674	1046.502261	987.7666025	1244.507935	2793.825851	0.125
3285830	---	1108.730524	1396.912926	1760	1975.533205	261.6255653	55	830.6093952	0.125
2433841	---	1108.730524	932.327523	880	1174.659072	2793.825851	2489.01587	1046.502261	0.1875
2726581	---	1108.730524	1046.502261	2489.01587	2349.318143	220	73.41619198	1174.659072	0.4375
2888710	---	1108.730524	659.2551138	587.3295358	554.365262	2349.318143	1864.655046	1760	0.125
2536359	41884	1174.659072	1661.21879	880	830.6093952	698.4564629	1479.977691	2793.825851	0.125
2578043	CXCR4	1174.659072	2349.318143	1760	1864.655046	440	587.3295358	783.990872	0.125
2891083	LOC100289627 // SNORD96A // SNORD95 // GNB2L1	1174.659072	1108.730524	987.7666025	698.4564629	2637.020455	2489.01587	1244.507935	0.125
2891355	IRF4	1174.659072	880	698.4564629	987.7666025	1174.659072	1396.912926	3135.963488	0.21875
2900061	HIST1H2BM	1174.659072	261.6255653	1046.502261	311.1269837	1975.533205	1244.507935	2637.020455	0.25
3285854	---	1174.659072	1975.533205	2489.01587	2637.020455	233.0818808	138.5913155	659.2551138	0.875
3294533	PPP3CB	1174.659072	830.6093952	1318.510228	932.327523	2349.318143	2093.004522	311.1269837	0.125

3560612	RNU1-1	1174.659072	987.7666025	987.7666025	1174.659072	2793.825851	2489.01587	1108.730524	0.125
3906968	SERINC3	1174.659072	880	622.2539674	466.1637615	1479.977691	2793.825851	1567.981744	0.1875
2888718	---	1174.659072	739.9888454	415.3046976	466.1637615	2489.01587	1760	2489.01587	0.375
3243464	---	1174.659072	1108.730524	2349.318143	2349.318143	146.832384	146.832384	1108.730524	0.375
3410592	---	1174.659072	1661.21879	2217.461048	2093.004522	493.8833013	783.990872	493.8833013	0.125
3581932	---	1174.659072	1975.533205	1975.533205	1567.981744	261.6255653	783.990872	329.6275569	0.125
2404183	LAPTM5	1244.507935	2349.318143	1975.533205	1318.510228	987.7666025	164.8137785	739.9888454	0.125
2726604	---	1244.507935	1318.510228	2793.825851	2637.020455	233.0818808	184.9972114	1244.507935	0.75
2844235	CANX	1244.507935	932.327523	739.9888454	739.9888454	1479.977691	1661.21879	2959.955382	0.1875
2895891	CD83	1244.507935	2793.825851	1567.981744	1661.21879	415.3046976	246.9416506	932.327523	0.25
3147058	ZNF706	1244.507935	987.7666025	880	932.327523	1567.981744	1396.912926	3135.963488	0.1875
3285824	---	1244.507935	1975.533205	2349.318143	2793.825851	155.5634919	36.70809599	739.9888454	1
3285847	---	1244.507935	1975.533205	2489.01587	2793.825851	87.30705786	38.89087297	783.990872	1.5
3548360	CALM3 // CALM2 // CALM1	1244.507935	880	523.2511306	587.3295358	1567.981744	2217.461048	2349.318143	0.125
3630699	CALML4	1244.507935	2217.461048	2489.01587	1864.655046	698.4564629	739.9888454	587.3295358	0.1875
3052634	---	1244.507935	1567.981744	2093.004522	2093.004522	554.365262	51.9130872	932.327523	0.1875
2404164	LAPTM5	1318.510228	2637.020455	1975.533205	1760	830.6093952	123.4708253	493.8833013	0.25
2434127	HIST2H2BE	1318.510228	698.4564629	932.327523	523.2511306	1760	1975.533205	2489.01587	0.125
2527646	ARPC2	1318.510228	1396.912926	932.327523	659.2551138	1174.659072	1975.533205	3322.437581	0.25
2729855	MT2A	1318.510228	622.2539674	1046.502261	783.990872	2489.01587	1661.21879	2793.825851	0.21875
2844212	CANX	1318.510228	830.6093952	880	698.4564629	1760	1975.533205	2959.955382	0.1875
2844230	CANX	1318.510228	830.6093952	739.9888454	698.4564629	1864.655046	2217.461048	2793.825851	0.21875
3009413	HSPB1	1318.510228	391.995436	739.9888454	739.9888454	2637.020455	2093.004522	2093.004522	0.25
3285831	---	1318.510228	1864.655046	2349.318143	2349.318143	261.6255653	138.5913155	880	0.375
3285855	---	1318.510228	1864.655046	2217.461048	2637.020455	138.5913155	46.24930284	932.327523	0.5
3285857	---	1318.510228	2093.004522	2489.01587	2793.825851	233.0818808	87.30705786	783.990872	1
3395444	HSPA8	1318.510228	1244.507935	1174.659072	1244.507935	932.327523	2093.004522	3135.963488	0.1875
2726601	---	1318.510228	1396.912926	2959.955382	2793.825851	277.182631	293.6647679	1244.507935	0.875
3206071	---	1318.510228	1760	2637.020455	2349.318143	880	739.9888454	698.4564629	0.1875
3285828	---	1318.510228	1864.655046	1864.655046	2217.461048	207.6523488	41.20344461	987.7666025	0.21875
3446490	---	1318.510228	2093.004522	493.8833013	233.0818808	932.327523	1760	1864.655046	0.125
2356125	TXNIP	1396.912926	2093.004522	2217.461048	1864.655046	587.3295358	293.6647679	932.327523	0.125
2442952	GPR161	1396.912926	1174.659072	1567.981744	987.7666025	3135.963488	2793.825851	698.4564629	0.375
2527640	ARPC2	1396.912926	1661.21879	1174.659072	987.7666025	1046.502261	2217.461048	3135.963488	0.1875

2560282	AUP1	1396.912926	880	659.2551138	587.3295358	1975.533205	2489.01587	2217.461048	0.1875
2726606	---	1396.912926	1318.510228	2489.01587	2489.01587	311.1269837	261.6255653	1396.912926	0.375
3259405	CCNJ	1396.912926	2489.01587	3135.963488	2793.825851	698.4564629	698.4564629	698.4564629	0.875
3285823	---	1396.912926	2093.004522	2489.01587	2637.020455	174.6141157	174.6141157	880	0.875
3326722	CD44	1396.912926	2093.004522	1244.507935	1396.912926	2093.004522	103.8261744	369.9944227	0.1875
3453749	TUBA1B	1396.912926	880	987.7666025	987.7666025	2217.461048	1760	2793.825851	0.125
3696722	NONO	1396.912926	1760	1396.912926	1244.507935	739.9888454	698.4564629	3135.963488	0.21875
3884803	ACTR5	1396.912926	2489.01587	2349.318143	1760	932.327523	783.990872	698.4564629	0.125
2888704	---	1396.912926	830.6093952	587.3295358	440	2793.825851	2093.004522	2959.955382	0.875
3326664	---	1396.912926	1864.655046	1244.507935	1479.977691	2349.318143	51.9130872	622.2539674	0.125
2443976	PFN1	1479.977691	1244.507935	1244.507935	932.327523	1661.21879	1975.533205	3951.06641	0.75
2685915	CLDND1	1479.977691	1108.730524	659.2551138	622.2539674	1760	2793.825851	1975.533205	0.1875
2726607	---	1479.977691	1396.912926	2793.825851	2637.020455	391.995436	155.5634919	1396.912926	0.5
2841295	ATP6V0E1	1479.977691	932.327523	554.365262	440	2217.461048	1975.533205	2217.461048	0.1875
3344233	TUBA1B	1479.977691	739.9888454	987.7666025	987.7666025	2349.318143	1975.533205	2637.020455	0.125
3346558	BIRC3	1479.977691	2793.825851	2217.461048	1864.655046	698.4564629	554.365262	1108.730524	0.21875
3453746	TUBA1B	1479.977691	932.327523	1174.659072	1108.730524	2093.004522	1661.21879	3135.963488	0.125
3934254	CSTB	1479.977691	739.9888454	932.327523	783.990872	2489.01587	2217.461048	2793.825851	0.25
2483009	---	1479.977691	1318.510228	1661.21879	1174.659072	2793.825851	2637.020455	830.6093952	0.125
2901831	---	1479.977691	2217.461048	2959.955382	2489.01587	880	880	659.2551138	0.375
3581931	---	1479.977691	2093.004522	1760	1567.981744	97.998859	1174.659072	97.998859	0.1875
4090948	---	1479.977691	1396.912926	1567.981744	880	2793.825851	2489.01587	659.2551138	0.1875
4114368	---	1479.977691	1318.510228	1479.977691	830.6093952	2793.825851	2349.318143	587.3295358	0.1875
2662480	PRRT3	1567.981744	2489.01587	2793.825851	1864.655046	1108.730524	1108.730524	659.2551138	0.1875
3315670	IFITM2	1567.981744	739.9888454	1174.659072	830.6093952	2217.461048	2637.020455	1975.533205	0.125
3453846	TUBA1A	1567.981744	1046.502261	1396.912926	1174.659072	1975.533205	1661.21879	3322.437581	0.1875
3494938	HSPD1P8	1567.981744	1567.981744	1479.977691	1661.21879	369.9944227	1396.912926	3322.437581	0.25
3052636	---	1567.981744	2093.004522	2093.004522	2093.004522	329.6275569	92.49860568	1174.659072	0.21875
3596982	---	1567.981744	2349.318143	2959.955382	2217.461048	932.327523	987.7666025	783.990872	0.21875
2491296	TMSB10	1661.21879	932.327523	1567.981744	987.7666025	2637.020455	2959.955382	1760	0.1875
2539874	YWHAQ	1661.21879	987.7666025	830.6093952	622.2539674	2793.825851	2959.955382	2489.01587	0.5
3192652	EEF1A1	1661.21879	2959.955382	2217.461048	1864.655046	261.6255653	1244.507935	1318.510228	0.21875
3457622	CS	1661.21879	1760	1318.510228	1108.730524	1661.21879	1479.977691	3951.06641	0.4375
4006736	GAPDHP65	1661.21879	587.3295358	1396.912926	1318.510228	2637.020455	2637.020455	1975.533205	0.125
2831333	---	1661.21879	2349.318143	2793.825851	2489.01587	1108.730524	1108.730524	739.9888454	0.1875
2726587	---	1760	1661.21879	2959.955382	2959.955382	415.3046976	261.6255653	1760	0.875

2899173	HIST1H1E	1760	1174.659072	932.327523	739.9888454	2489.01587	2489.01587	2349.318143	0.125
2953943	C6orf132	1760	1108.730524	1760	1479.977691	2489.01587	2349.318143	329.6275569	0.125
3243457	---	1760	1567.981744	2793.825851	2637.020455	523.2511306	220	1760	0.4375
3285851	---	1760	2093.004522	2217.461048	2489.01587	1174.659072	103.8261744	1396.912926	0.1875
3453747	TUBA1B	1760	1046.502261	1396.912926	1318.510228	2489.01587	1975.533205	3729.310092	0.375
3655956	ALDOA	1760	739.9888454	1244.507935	987.7666025	2349.318143	2489.01587	2637.020455	0.1875
3666783	NFAT5	1760	2349.318143	2637.020455	2637.020455	1108.730524	1244.507935	830.6093952	0.125
3581929	---	1760	2217.461048	1975.533205	1864.655046	130.8127827	1479.977691	77.78174593	0.25
4119114	---	1760	1760	1760	1046.502261	3135.963488	2959.955382	880	0.25
2726613	---	1864.655046	1760	2959.955382	3135.963488	659.2551138	293.6647679	1864.655046	0.875
2844217	CANX	1864.655046	1244.507935	1244.507935	1108.730524	2349.318143	2637.020455	3135.963488	0.1875
2901922	TUBB	1864.655046	830.6093952	1396.912926	1244.507935	2489.01587	2349.318143	3135.963488	0.21875
2972849	---	1864.655046	2959.955382	2217.461048	2793.825851	1396.912926	1244.507935	1244.507935	0.125
3092398	TUBB	1864.655046	830.6093952	1396.912926	1318.510228	2489.01587	2349.318143	3322.437581	0.21875
3148369	TAGLN2	1864.655046	1864.655046	1174.659072	880	1661.21879	1975.533205	3135.963488	0.125
3395441	HSPA8	1864.655046	1661.21879	1567.981744	1760	1567.981744	2959.955382	3520	0.1875
3395442	HSPA8	1864.655046	1661.21879	1661.21879	1760	1567.981744	2959.955382	3951.06641	0.375
3413821	TUBA1B	1864.655046	1174.659072	1567.981744	1567.981744	2489.01587	1975.533205	3520	0.1875
3585274	GABRG3	1864.655046	1108.730524	1975.533205	1567.981744	2349.318143	2489.01587	349.2282314	0.125
3992397	GAPDHP67	1864.655046	698.4564629	1567.981744	1479.977691	2793.825851	2637.020455	1975.533205	0.125
4006738	GAPDHP65	1864.655046	783.990872	1864.655046	1661.21879	2793.825851	2959.955382	1760	0.125
2981435	---	1864.655046	1975.533205	1864.655046	1661.21879	415.3046976	329.6275569	2093.004522	0.125
2981447	---	1864.655046	2217.461048	1864.655046	1760	220	391.995436	2093.004522	0.1875
4048799	---	1864.655046	2093.004522	1864.655046	1760	415.3046976	466.1637615	2217.461048	0.125
2325808	TMEM50A	1975.533205	1661.21879	830.6093952	698.4564629	2093.004522	2637.020455	3322.437581	0.4375
2325961	TMEM50A	1975.533205	1661.21879	880	739.9888454	2093.004522	2637.020455	3135.963488	0.25
2439849	TAGLN2	1975.533205	1975.533205	1108.730524	932.327523	1760	2093.004522	3135.963488	0.125
2532044	LOC100506248 // MIR1244-2 // MIR1244-3 // MIR1244-1 // LOC728026	1975.533205	1975.533205	2217.461048	1760	739.9888454	1244.507935	3135.963488	0.125

	// PTMA								
2578044	CXCR4	1975.533205	3135.963488	2489.01587	2349.318143	698.4564629	1396.912926	1479.977691	0.21875
2726586	---	1975.533205	1760	2793.825851	2793.825851	523.2511306	311.1269837	2093.004522	0.5
2726619	---	1975.533205	1661.21879	2793.825851	2793.825851	587.3295358	184.9972114	2217.461048	0.75
2841289	ATP6V0E1	1975.533205	1318.510228	783.990872	622.2539674	2793.825851	2637.020455	3135.963488	0.75
2960915	EEF1A1	1975.533205	2489.01587	1760	1318.510228	987.7666025	2093.004522	3135.963488	0.125
3041180	EEF1A1P6	1975.533205	2793.825851	2093.004522	1046.502261	830.6093952	1760	2793.825851	0.1875
2981438	---	1975.533205	2093.004522	1975.533205	1864.655046	369.9944227	493.8833013	2217.461048	0.1875
2981440	---	1975.533205	2217.461048	1864.655046	1864.655046	440	415.3046976	2093.004522	0.125
2981443	---	1975.533205	2093.004522	1975.533205	1760	391.995436	440	2093.004522	0.125
2981449	---	1975.533205	2349.318143	1975.533205	1864.655046	261.6255653	233.0818808	2093.004522	0.25
4048793	---	1975.533205	2093.004522	1864.655046	1864.655046	415.3046976	440	2217.461048	0.125
4048796	---	1975.533205	2217.461048	1975.533205	1760	369.9944227	466.1637615	2093.004522	0.1875
4048798	---	1975.533205	2093.004522	1864.655046	1760	415.3046976	466.1637615	2217.461048	0.125
4048801	---	1975.533205	2217.461048	1975.533205	1760	329.6275569	440	2093.004522	0.1875
2738235	EEF1A1	2093.004522	3135.963488	2349.318143	1046.502261	932.327523	1864.655046	2793.825851	0.21875
2890162	HNRNPH1	2093.004522	3135.963488	2489.01587	2349.318143	880	1479.977691	1661.21879	0.125
2483119	---	2093.004522	1975.533205	2217.461048	1479.977691	3520	3520	1108.730524	0.375
2981437	---	2093.004522	2217.461048	1975.533205	1975.533205	415.3046976	523.2511306	2217.461048	0.1875
2981442	---	2093.004522	2217.461048	1975.533205	1864.655046	587.3295358	554.365262	2217.461048	0.125
2981450	---	2093.004522	2217.461048	2093.004522	1975.533205	493.8833013	622.2539674	2349.318143	0.1875
3581928	---	2093.004522	2489.01587	2217.461048	2349.318143	87.30705786	1864.655046	123.4708253	0.75
4048797	---	2093.004522	2349.318143	1975.533205	1864.655046	391.995436	349.2282314	2093.004522	0.21875
2849420	EEF1A1	2217.461048	3135.963488	2349.318143	1108.730524	987.7666025	1975.533205	2959.955382	0.21875
2890159	HNRNPH1	2217.461048	3135.963488	2637.020455	2349.318143	739.9888454	1396.912926	1760	0.1875
2960918	EEF1A1	2217.461048	3135.963488	2349.318143	1046.502261	987.7666025	1975.533205	3135.963488	0.25
3181114	LOC441454	2217.461048	2217.461048	2349.318143	1975.533205	783.990872	1567.981744	3322.437581	0.1875
3192648	EEF1A1	2217.461048	3135.963488	2349.318143	1108.730524	1046.502261	2093.004522	2959.955382	0.21875
3215508	LOC441454	2217.461048	2217.461048	2349.318143	1975.533205	659.2551138	1661.21879	3135.963488	0.125
3749823	YWHAZ	2217.461048	2093.004522	1108.730524	1318.510228	1975.533205	2489.01587	3322.437581	0.125
3816400	OAZ1	2217.461048	1760	1174.659072	987.7666025	2349.318143	2489.01587	3135.963488	0.125
4029703	TSPY4	2217.461048	2793.825851	3322.437581	2637.020455	1479.977691	1567.981744	1244.507935	0.1875
4029735	TSPY3	2217.461048	2959.955382	3520	2793.825851	1479.977691	1567.981744	1108.730524	0.375
3836706	---	2217.461048	1661.21879	2217.461048	1975.533205	2959.955382	2959.955382	369.9944227	0.25
2532046	PTMA	2349.318143	2349.318143	2489.01587	1975.533205	659.2551138	1396.912926	3322.437581	0.21875
2908498	HSP90AB1	2349.318143	2637.020455	2093.004522	2349.318143	1046.502261	1661.21879	3729.310092	0.21875

2960930	EEF1A1	2349.318143	2637.020455	1975.533205	739.9888454	1661.21879	2637.020455	2959.955382	0.125
3036926	ACTB	2349.318143	1864.655046	391.995436	1975.533205	2637.020455	2349.318143	2349.318143	0.125
3238820	YWHAZ	2349.318143	2093.004522	1244.507935	1318.510228	2217.461048	2489.01587	3729.310092	0.21875
3335170	MALAT1	2349.318143	2489.01587	2349.318143	2217.461048	987.7666025	2959.955382	830.6093952	0.1875
3827336	RPS27	2349.318143	3520	2793.825851	2093.004522	1864.655046	2217.461048	932.327523	0.1875
4006734	GAPDHP65	2349.318143	659.2551138	1864.655046	1661.21879	2793.825851	2959.955382	3135.963488	0.25
4028843	TSPY1	2349.318143	2959.955382	3322.437581	2793.825851	1479.977691	1760	1244.507935	0.1875
4029681	TSPY3	2349.318143	2959.955382	3322.437581	2637.020455	1479.977691	1760	1244.507935	0.1875
4029927	---	2349.318143	3322.437581	2637.020455	2959.955382	1975.533205	1244.507935	1244.507935	0.1875
4074465	---	2349.318143	2349.318143	2349.318143	1567.981744	3520	3520	1174.659072	0.25
4129546	---	2349.318143	2349.318143	2217.461048	1567.981744	3729.310092	3729.310092	1479.977691	0.375
4134740	---	2349.318143	2349.318143	2093.004522	1396.912926	3520	3520	1244.507935	0.375
2993208	MPP6	2489.01587	3135.963488	3322.437581	2793.825851	1760	1760	1479.977691	0.125
3335183	MALAT1	2489.01587	2793.825851	2489.01587	2349.318143	1760	3322.437581	880	0.1875
3402638	GAPDH	2489.01587	987.7666025	2217.461048	1975.533205	3135.963488	3135.963488	2637.020455	0.125
3405241	PTMA	2489.01587	2489.01587	2489.01587	2093.004522	523.2511306	1479.977691	3322.437581	0.25
4037584	---	2489.01587	2349.318143	2489.01587	2217.461048	3135.963488	2959.955382	587.3295358	0.21875
3402636	GAPDH	2637.020455	1046.502261	2489.01587	2217.461048	3520	3520	2637.020455	0.21875
2539883	YWHAQ	2793.825851	1975.533205	1479.977691	1318.510228	3520	3729.310092	3729.310092	0.875
2825508	PTMA	2793.825851	2793.825851	2793.825851	2349.318143	1046.502261	1975.533205	3729.310092	0.21875
2960912	EEF1A1	2793.825851	3135.963488	2637.020455	1975.533205	1396.912926	2637.020455	3729.310092	0.125
2915390	---	2793.825851	1244.507935	2489.01587	2217.461048	3135.963488	3322.437581	3135.963488	0.125
3041177	EEF1A1	2959.955382	3729.310092	2959.955382	1975.533205	1864.655046	2959.955382	3729.310092	0.125
3192651	EEF1A1	2959.955382	3729.310092	2793.825851	1975.533205	1567.981744	2793.825851	3729.310092	0.21875
3402647	GAPDH	2959.955382	1108.730524	2637.020455	2349.318143	3322.437581	3520	3322.437581	0.21875
3402640	GAPDH	3135.963488	987.7666025	2637.020455	2349.318143	3322.437581	3520	3951.06641	0.5
4097642	---	3135.963488	3135.963488	2959.955382	2349.318143	3729.310092	3951.06641	1864.655046	0.125
2405003	MARCKSL1	3322.437581	3135.963488	3135.963488	2793.825851	3520	3520	783.990872	0.4375
4128085	---	3520	3520	3322.437581	2637.020455	4186.009045	4186.009045	1760	0.25

Supplementary Table S5.

Frequencies of the musically interpreted microarray data from HL cell lines and normal B cells (gene level). Affymetrix Human Exon 1.0ST microarray data (gene level) from 3 Hodgkin's lymphoma cell lines and three CD19-positive B cell samples (from GSE20200) were transformed into melodies by using the following parameters: minimal frequency: 27.5; number of different frequencies: 88 (keys); number of tone steps per octave: 12; minimal duration: 1/8; number of tones: 288. Presented are the frequencies of the individual samples and the frequency of the median signal intensity as well as the duration of the filtered 288 probe sets. The resulting melodies are presented as Supplementary MP3 files 41CD19aGe288, 42CD19bGe288, 43CD19cGe288, 44HDLM2Ge288, 45L428Ge288, 46L540Ge288, and 47MedHLGe288. Stereo versions with the median as reference base line are presented as Supplementary MP3 files 48CD19aGe288st, 49CD19bGe288st, 50CD19cGe288st, 51HDLM2Ge288st, 52L428Ge288st, and 53L540Ge288st.

Probe Set ID	Gene Symbol	Duration	Median	CD19a	CD19b	CD19c	HDLM-2	L-540	L-428
4023242	CT45A2 // CT45A2	0.125	48.9994295	36.70809599	43.65352893	69.29565774	46.24930284	51.9130872	1318.510228
2876707	IL9	0.125	97.998859	87.30705786	97.998859	92.49860568	1318.510228	97.998859	103.8261744
2441220	SH2D1B	0.125	103.8261744	103.8261744	97.998859	92.49860568	103.8261744	103.8261744	1318.510228
4048265	HLA-DRB1	0.125	116.5409404	82.40688923	116.5409404	69.29565774	1396.912926	116.5409404	138.5913155
2633191	GPR15	0.25	123.4708253	130.8127827	97.998859	116.5409404	87.30705786	1864.655046	311.1269837
2335014	CYP4Z1	0.125	130.8127827	130.8127827	138.5913155	103.8261744	1108.730524	123.4708253	1244.507935
2412312	TTC39A	0.125	138.5913155	103.8261744	110	116.5409404	415.3046976	155.5634919	1318.510228
3470523	SELPLG	0.1875	220	146.832384	146.832384	195.997718	698.4564629	1661.21879	246.9416506
2902407	LTA	0.1875	233.0818808	207.6523488	246.9416506	220	1760	195.997718	698.4564629
2908762	RUNX2	0.125	246.9416506	184.9972114	195.997718	207.6523488	293.6647679	932.327523	1318.510228
3174816	ANXA1	0.125	277.182631	116.5409404	130.8127827	103.8261744	554.365262	1318.510228	622.2539674
3834502	CD79A	0.21875	349.2282314	1661.21879	830.6093952	987.7666025	130.8127827	138.5913155	116.5409404
2946194	HIST1H1A // HIST1H1A	0.1875	369.9944227	138.5913155	207.6523488	174.6141157	739.9888454	554.365262	1661.21879
3976341	TIMP1	0.1875	369.9944227	233.0818808	246.9416506	207.6523488	1479.977691	1479.977691	587.3295358
2764192	SEL1L3	0.125	391.995436	622.2539674	1108.730524	1108.730524	164.8137785	207.6523488	82.40688923
3387259	SESN3	0.125	391.995436	1396.912926	783.990872	932.327523	138.5913155	110	220
3417485	NABP2	0.125	391.995436	311.1269837	391.995436	415.3046976	391.995436	349.2282314	1661.21879
3445786	ARHGDIB	0.1875	391.995436	1174.659072	1244.507935	1174.659072	138.5913155	116.5409404	116.5409404

2724472	UBE2K	0.125	415.3046976	466.1637615	369.9944227	349.2282314	233.0818808	698.4564629	1567.981744
2988726	FSCN1	0.75	415.3046976	184.9972114	293.6647679	277.182631	1108.730524	554.365262	2637.020455
3662687	CCL22	0.125	415.3046976	195.997718	932.327523	1318.510228	1108.730524	184.9972114	184.9972114
3484060	ALOX5AP // ALOX5AP	0.21875	440	391.995436	493.8833013	415.3046976	1975.533205	523.2511306	311.1269837
2844226	---	0.125	440	293.6647679	164.8137785	369.9944227	493.8833013	554.365262	1479.977691
3570050	---	0.125	440	369.9944227	82.40688923	155.5634919	440	622.2539674	1396.912926
3819550	---	0.125	466.1637615	659.2551138	493.8833013	466.1637615	311.1269837	233.0818808	1567.981744
3315675	IFITM1	0.1875	493.8833013	277.182631	261.6255653	246.9416506	1567.981744	1396.912926	880
2516455	---	0.1875	493.8833013	1567.981744	880	830.6093952	130.8127827	164.8137785	311.1269837
2775468	---	0.1875	493.8833013	880	587.3295358	391.995436	369.9944227	233.0818808	1864.655046
2457988	ZNF706 // ZNF706	0.1875	523.2511306	369.9944227	311.1269837	415.3046976	622.2539674	739.9888454	1864.655046
3334125	COX8A	0.125	523.2511306	329.6275569	293.6647679	277.182631	880	739.9888454	1567.981744
3980920	---	0.125	523.2511306	622.2539674	349.2282314	220	493.8833013	466.1637615	1567.981744
2563785	---	0.125	554.365262	1174.659072	1046.502261	1046.502261	277.182631	103.8261744	130.8127827
2655773	POLR2H	0.125	554.365262	369.9944227	415.3046976	369.9944227	698.4564629	698.4564629	1760
2948887	---	0.125	587.3295358	1760	493.8833013	622.2539674	739.9888454	466.1637615	622.2539674
2436156	---	0.125	587.3295358	880	415.3046976	523.2511306	261.6255653	622.2539674	1661.21879
2365119	MGST3	0.375	622.2539674	277.182631	329.6275569	277.182631	2217.461048	1108.730524	1174.659072
3060300	SRI	0.25	622.2539674	440	466.1637615	440	783.990872	1046.502261	2349.318143
3253438	RPS24	0.125	622.2539674	1174.659072	1174.659072	987.7666025	130.8127827	220	440
3666732	CYB5B	0.375	622.2539674	830.6093952	622.2539674	659.2551138	415.3046976	523.2511306	2489.01587
3253453	---	0.125	622.2539674	1174.659072	1174.659072	987.7666025	130.8127827	220	440
3453389	---	0.125	622.2539674	466.1637615	440	369.9944227	739.9888454	880	1661.21879
3062665	---	0.21875	659.2551138	554.365262	440	369.9944227	698.4564629	698.4564629	2093.004522
3740126	YWHAE // YWHAE	0.125	659.2551138	932.327523	622.2539674	739.9888454	261.6255653	466.1637615	1661.21879
3944882	LGALS1	0.125	659.2551138	369.9944227	466.1637615	493.8833013	1479.977691	1567.981744	932.327523
2536358	---	0.21875	659.2551138	880	523.2511306	493.8833013	415.3046976	698.4564629	2093.004522
2895841	CD83 // CD83	0.1875	698.4564629	1760	932.327523	1046.502261	329.6275569	207.6523488	554.365262
2981912	EZR	0.125	698.4564629	1567.981744	880	1244.507935	277.182631	329.6275569	554.365262
2436157	---	0.375	698.4564629	987.7666025	698.4564629	739.9888454	415.3046976	587.3295358	2489.01587
2536355	---	0.21875	698.4564629	783.990872	466.1637615	554.365262	523.2511306	1174.659072	2217.461048
3453384	---	0.21875	698.4564629	739.9888454	587.3295358	739.9888454	587.3295358	622.2539674	2349.318143

3555886	---	0.125	698.4564629	1108.730524	698.4564629	830.6093952	233.0818808	466.1637615	1567.981744
2899102	HIST1H3C	0.21875	739.9888454	61.73541266	415.3046976	184.9972114	1396.912926	1244.507935	1479.977691
3014714	ARPC1B	0.125	739.9888454	523.2511306	493.8833013	523.2511306	880	1318.510228	1760
3429312	HSP90B1	0.125	739.9888454	1661.21879	830.6093952	1174.659072	311.1269837	493.8833013	698.4564629
3253447	---	0.125	739.9888454	1479.977691	1479.977691	1108.730524	440	415.3046976	554.365262
2468622	ID2 // ID2	0.5	783.990872	174.6141157	493.8833013	830.6093952	2349.318143	2093.004522	830.6093952
3190659	SET	0.125	783.990872	1244.507935	880	698.4564629	622.2539674	523.2511306	1975.533205
2775485	---	0.125	783.990872	932.327523	987.7666025	739.9888454	246.9416506	369.9944227	1567.981744
3474938	---	0.25	783.990872	415.3046976	220	220	1318.510228	1396.912926	1864.655046
3465409	BTG1	0.125	830.6093952	1760	1244.507935	1108.730524	523.2511306	622.2539674	523.2511306
3580179	HSP90AA1	0.125	830.6093952	1396.912926	830.6093952	987.7666025	130.8127827	311.1269837	1108.730524
2841296	---	0.21875	830.6093952	587.3295358	369.9944227	277.182631	1479.977691	987.7666025	1864.655046
3377895	---	0.1875	830.6093952	739.9888454	932.327523	830.6093952	783.990872	659.2551138	2217.461048
3766926	---	0.125	830.6093952	1567.981744	1046.502261	1046.502261	207.6523488	523.2511306	698.4564629
2356115	TXNIP	0.25	880	1479.977691	1661.21879	1661.21879	349.2282314	110	554.365262
2578028	CXCR4	0.21875	880	1975.533205	1318.510228	1479.977691	293.6647679	440	659.2551138
2843619	HNRNPAB	0.125	880	698.4564629	587.3295358	440	932.327523	932.327523	1760
2902559	CSNK2B	0.125	880	830.6093952	880	880	830.6093952	739.9888454	2093.004522
4083032	---	0.125	880	783.990872	987.7666025	554.365262	1567.981744	1396.912926	349.2282314
2775482	---	0.1875	880	932.327523	987.7666025	830.6093952	329.6275569	466.1637615	1975.533205
2844213	---	0.21875	880	622.2539674	587.3295358	587.3295358	1046.502261	1318.510228	2349.318143
2844247	---	0.125	880	659.2551138	391.995436	587.3295358	1108.730524	987.7666025	1864.655046
3145955	---	0.4375	880	2093.004522	1975.533205	1567.981744	466.1637615	329.6275569	391.995436
3435860	---	0.1875	880	698.4564629	466.1637615	587.3295358	880	932.327523	2093.004522
3556556	OR6J1	0.125	932.327523	830.6093952	698.4564629	523.2511306	1108.730524	880	1864.655046
3838385	CD37	0.21875	932.327523	1479.977691	1864.655046	1567.981744	554.365262	415.3046976	466.1637615
4066723	---	0.125	932.327523	880	987.7666025	554.365262	1661.21879	1396.912926	349.2282314
2831949	---	0.125	932.327523	1661.21879	932.327523	1108.730524	146.832384	622.2539674	1108.730524
2844222	---	0.1875	932.327523	587.3295358	554.365262	554.365262	1174.659072	1479.977691	1864.655046
2844228	---	0.21875	932.327523	622.2539674	554.365262	622.2539674	1174.659072	1244.507935	2217.461048
2844242	---	0.21875	932.327523	622.2539674	587.3295358	783.990872	1046.502261	1479.977691	2217.461048
3453383	---	0.25	932.327523	932.327523	739.9888454	1046.502261	523.2511306	783.990872	2489.01587
3555888	---	0.125	932.327523	1479.977691	1046.502261	932.327523	261.6255653	554.365262	1567.981744
3766918	---	0.125	932.327523	1567.981744	1108.730524	1174.659072	97.998859	369.9944227	830.6093952
2894711	TMEM14B	0.1875	987.7666025	783.990872	622.2539674	698.4564629	1046.502261	1108.730524	2217.461048
3446868	LDHB	0.25	987.7666025	698.4564629	932.327523	1108.730524	466.1637615	1174.659072	2489.01587

2318746	---	0.125	987.7666025	1108.730524	739.9888454	987.7666025	698.4564629	932.327523	2093.004522
2325205	---	0.21875	987.7666025	2093.004522	1567.981744	1318.510228	311.1269837	739.9888454	659.2551138
2436160	---	0.1875	987.7666025	783.990872	659.2551138	698.4564629	1046.502261	1174.659072	2217.461048
2844238	---	0.375	987.7666025	698.4564629	830.6093952	783.990872	1108.730524	1396.912926	2793.825851
2908509	---	0.125	987.7666025	1108.730524	932.327523	1318.510228	184.9972114	246.9416506	1479.977691
3362744	---	0.125	987.7666025	1760	1174.659072	1479.977691	523.2511306	783.990872	783.990872
3377893	---	0.125	987.7666025	783.990872	698.4564629	587.3295358	987.7666025	1244.507935	1864.655046
3570057	---	0.125	987.7666025	659.2551138	440	493.8833013	1244.507935	1244.507935	1760
3819579	---	0.125	987.7666025	1567.981744	1174.659072	1244.507935	233.0818808	440	932.327523
2844203	CANX	0.1875	1046.502261	739.9888454	698.4564629	698.4564629	1244.507935	1318.510228	2217.461048
2888698	LMAN2	0.4375	1046.502261	622.2539674	493.8833013	466.1637615	2217.461048	1479.977691	2217.461048
2900059	HIST1H2BM // HIST1H2BM	0.4375	1046.502261	220	880	329.6275569	1567.981744	1046.502261	2489.01587
3592023	B2M	0.125	1046.502261	1760	1318.510228	1396.912926	783.990872	830.6093952	349.2282314
4058352	---	0.1875	1046.502261	987.7666025	1174.659072	659.2551138	1975.533205	1661.21879	466.1637615
4109262	---	0.1875	1046.502261	880	1174.659072	659.2551138	2093.004522	1567.981744	493.8833013
2536362	---	0.1875	1046.502261	1975.533205	1108.730524	1108.730524	391.995436	880	1661.21879
2766422	---	0.1875	1046.502261	1864.655046	1760	1396.912926	493.8833013	783.990872	698.4564629
2841286	---	0.375	1046.502261	587.3295358	493.8833013	415.3046976	1864.655046	1661.21879	2217.461048
2844241	---	0.375	1046.502261	698.4564629	783.990872	659.2551138	1244.507935	1479.977691	2793.825851
2844246	---	0.125	1046.502261	830.6093952	493.8833013	659.2551138	1244.507935	1174.659072	1975.533205
2949061	---	0.125	1046.502261	1396.912926	1046.502261	1108.730524	554.365262	493.8833013	1975.533205
3309937	---	0.25	1046.502261	2489.01587	1975.533205	1396.912926	698.4564629	659.2551138	830.6093952
3555898	---	0.125	1046.502261	1479.977691	1108.730524	1108.730524	391.995436	698.4564629	1760
3766912	---	0.1875	1046.502261	1864.655046	1046.502261	1174.659072	261.6255653	493.8833013	1174.659072
3766924	---	0.21875	1046.502261	2093.004522	1396.912926	1318.510228	195.997718	587.3295358	830.6093952
2404158	LAPTM5	0.5	1108.730524	2349.318143	1864.655046	1864.655046	659.2551138	146.832384	523.2511306
2435383	S100A10 // S100A10	0.125	1108.730524	739.9888454	880	698.4564629	1479.977691	1975.533205	1479.977691
2491271	TMSB10	0.125	1108.730524	698.4564629	1174.659072	880	1864.655046	2093.004522	1174.659072
2527606	ARPC2	0.25	1108.730524	1244.507935	932.327523	987.7666025	698.4564629	1567.981744	2793.825851
3417309	PA2G4	0.375	1108.730524	830.6093952	1108.730524	932.327523	987.7666025	1046.502261	2959.955382
3476741	UBC	0.1875	1108.730524	622.2539674	1174.659072	1174.659072	1975.533205	1975.533205	932.327523
3708186	RNASEK // RNASEK	0.21875	1108.730524	1046.502261	932.327523	830.6093952	987.7666025	1244.507935	2637.020455

4081344	---	0.125	1108.730524	987.7666025	1244.507935	698.4564629	1864.655046	1479.977691	349.2282314
2775480	---	0.5	1108.730524	1396.912926	1318.510228	1046.502261	293.6647679	523.2511306	2793.825851
2840627	---	0.125	1108.730524	1108.730524	1174.659072	1661.21879	220	932.327523	880
2844229	---	0.375	1108.730524	659.2551138	622.2539674	659.2551138	1567.981744	1864.655046	2489.01587
2908503	---	0.125	1108.730524	1174.659072	1046.502261	1567.981744	277.182631	739.9888454	1479.977691
3309938	---	0.1875	1108.730524	1975.533205	1760	1318.510228	830.6093952	880	587.3295358
3362740	---	0.125	1108.730524	1567.981744	1318.510228	1567.981744	369.9944227	783.990872	987.7666025
3570052	---	0.25	1108.730524	830.6093952	554.365262	415.3046976	1318.510228	1244.507935	2349.318143
4133728	---	0.1875	1174.659072	1046.502261	1244.507935	739.9888454	2093.004522	1661.21879	391.995436
2436147	---	0.125	1174.659072	1567.981744	880	1244.507935	987.7666025	1046.502261	2349.318143
2436155	---	0.21875	1174.659072	1318.510228	783.990872	1046.502261	739.9888454	1174.659072	2489.01587
2904700	---	0.125	1174.659072	1975.533205	1396.912926	1396.912926	659.2551138	932.327523	932.327523
2904703	---	0.25	1174.659072	2349.318143	1661.21879	1864.655046	659.2551138	830.6093952	698.4564629
2908500	---	0.25	1174.659072	1318.510228	1108.730524	1760	246.9416506	622.2539674	2217.461048
3322069	---	0.1875	1174.659072	1760	1396.912926	1108.730524	233.0818808	554.365262	1396.912926
3377465	---	0.1875	1174.659072	1975.533205	1975.533205	1567.981744	739.9888454	783.990872	987.7666025
3377899	---	0.125	1174.659072	698.4564629	880	783.990872	1396.912926	1396.912926	2093.004522
3740531	---	0.125	1174.659072	1479.977691	1046.502261	1174.659072	932.327523	1174.659072	2349.318143
3740532	---	0.125	1174.659072	1174.659072	987.7666025	932.327523	932.327523	1244.507935	2217.461048
3819546	---	0.125	1174.659072	987.7666025	1244.507935	1108.730524	932.327523	1108.730524	2349.318143
3980914	---	0.1875	1174.659072	1479.977691	1396.912926	1174.659072	622.2539674	830.6093952	2349.318143
4118126	---	0.21875	1244.507935	1174.659072	1396.912926	880	2217.461048	1864.655046	493.8833013
2320068	---	0.125	1244.507935	1661.21879	1396.912926	1318.510228	415.3046976	587.3295358	1244.507935
2325198	---	0.1875	1244.507935	2093.004522	1760	1244.507935	659.2551138	1244.507935	622.2539674
2840629	---	0.125	1244.507935	1108.730524	1396.912926	1661.21879	311.1269837	659.2551138	1396.912926
3551689	---	0.21875	1244.507935	1396.912926	2217.461048	2489.01587	880	987.7666025	1108.730524
3740533	---	0.125	1244.507935	1244.507935	1174.659072	987.7666025	1108.730524	1479.977691	2489.01587
3766922	---	0.25	1244.507935	2489.01587	1479.977691	1661.21879	391.995436	932.327523	1046.502261
3831192	---	0.125	1244.507935	1174.659072	1108.730524	1318.510228	1661.21879	932.327523	2349.318143
3980912	---	0.125	1244.507935	1567.981744	1318.510228	1244.507935	698.4564629	830.6093952	2217.461048
2844365	---	0.125	1244.507935	1567.981744	1479.977691	2217.461048	987.7666025	932.327523	987.7666025
2841284	ATP6V0E1	0.25	1318.510228	830.6093952	523.2511306	493.8833013	1864.655046	1760	2217.461048
2325201	---	0.25	1318.510228	2217.461048	2093.004522	1661.21879	391.995436	932.327523	932.327523
2536359	---	0.75	1318.510228	1864.655046	987.7666025	1046.502261	698.4564629	1479.977691	3322.437581
2775476	---	0.5	1318.510228	1479.977691	1479.977691	1318.510228	739.9888454	783.990872	3135.963488
2844219	---	0.125	1318.510228	880	880	1046.502261	1567.981744	1760	2217.461048

2844235	---	0.875	1318.510228	987.7666025	830.6093952	932.327523	1479.977691	1661.21879	3520
2908495	---	0.1875	1318.510228	1318.510228	1318.510228	1975.533205	440	932.327523	1975.533205
3255512	---	0.125	1318.510228	2093.004522	1760	1479.977691	554.365262	1108.730524	1318.510228
3556589	---	0.25	1318.510228	987.7666025	830.6093952	659.2551138	1396.912926	1479.977691	2637.020455
3740534	---	0.1875	1318.510228	1244.507935	1046.502261	987.7666025	1244.507935	1479.977691	2637.020455
3766929	---	0.1875	1318.510228	1864.655046	1479.977691	1567.981744	233.0818808	783.990872	1244.507935
3749744	---	0.125	1318.510228	1396.912926	2217.461048	1864.655046	987.7666025	1046.502261	1318.510228
2539869	YWHAQ	0.1875	1396.912926	987.7666025	739.9888454	739.9888454	1864.655046	1864.655046	2093.004522
2908474	HSP90AB1	0.125	1396.912926	1760	1318.510228	1760	554.365262	987.7666025	1567.981744
3563317	RPS29 // RPS29	0.25	1396.912926	2349.318143	2093.004522	1975.533205	783.990872	987.7666025	783.990872
2325203	---	0.1875	1396.912926	2217.461048	2093.004522	1479.977691	880	1174.659072	783.990872
2766427	---	0.1875	1396.912926	2217.461048	1864.655046	1864.655046	554.365262	1046.502261	1174.659072
2841301	---	0.125	1396.912926	1108.730524	783.990872	659.2551138	1760	1396.912926	1864.655046
2844212	---	1	1396.912926	932.327523	987.7666025	880	1760	1975.533205	3520
2844215	---	0.25	1396.912926	830.6093952	932.327523	880	1975.533205	1975.533205	2489.01587
2908492	---	0.1875	1396.912926	1479.977691	1396.912926	1864.655046	523.2511306	880	1975.533205
2908499	---	0.375	1396.912926	1661.21879	1244.507935	1864.655046	233.0818808	622.2539674	2217.461048
2908501	---	0.25	1396.912926	1479.977691	1244.507935	1864.655046	369.9944227	783.990872	2349.318143
2908505	---	0.125	1396.912926	1760	1318.510228	1760	554.365262	987.7666025	1567.981744
3145960	---	0.125	1396.912926	1864.655046	1174.659072	739.9888454	1396.912926	1108.730524	1975.533205
3416490	---	0.125	1396.912926	1661.21879	1479.977691	1661.21879	349.2282314	739.9888454	1479.977691
2328561	---	0.21875	1396.912926	1318.510228	2349.318143	2349.318143	1174.659072	1318.510228	830.6093952
2993694	---	0.125	1396.912926	1760	1760	2349.318143	987.7666025	1108.730524	1244.507935
4129216	---	0.21875	1479.977691	1396.912926	1661.21879	987.7666025	2349.318143	1975.533205	493.8833013
2436152	---	0.1875	1479.977691	1567.981744	932.327523	1318.510228	1108.730524	1479.977691	2637.020455
2844214	---	0.375	1479.977691	880	880	987.7666025	1975.533205	2217.461048	2637.020455
2844227	---	0.4375	1479.977691	1108.730524	1108.730524	1174.659072	1567.981744	1760	3322.437581
2844230	---	0.875	1479.977691	880	830.6093952	880	1864.655046	2217.461048	3322.437581
3212314	---	0.125	1479.977691	1864.655046	1760	1479.977691	587.3295358	739.9888454	1567.981744
3225207	---	0.125	1479.977691	1396.912926	1396.912926	987.7666025	1661.21879	1318.510228	2489.01587
3377467	---	0.1875	1479.977691	2349.318143	2349.318143	1975.533205	1046.502261	1108.730524	1318.510228
3555896	---	0.21875	1479.977691	1975.533205	1567.981744	1567.981744	783.990872	1046.502261	2637.020455
2832041	---	0.125	1479.977691	1864.655046	1661.21879	2489.01587	1108.730524	1174.659072	1244.507935
3809800	---	0.125	1479.977691	1760	1661.21879	2093.004522	880	987.7666025	1479.977691
4114368	---	0.375	1567.981744	1479.977691	1760	1046.502261	2793.825851	2349.318143	659.2551138

2325200	---	0.1875	1567.981744	2093.004522	2093.004522	1760	622.2539674	1396.912926	1174.659072
2841295	---	0.5	1567.981744	1046.502261	622.2539674	523.2511306	2217.461048	1975.533205	2637.020455
2908491	---	0.1875	1567.981744	1567.981744	1661.21879	1975.533205	698.4564629	1108.730524	2349.318143
3416485	---	0.125	1567.981744	1864.655046	1661.21879	1760	698.4564629	1174.659072	1975.533205
3807490	---	0.125	1567.981744	2217.461048	1975.533205	1479.977691	830.6093952	987.7666025	1760
2320124	---	0.1875	1567.981744	1864.655046	1760	2489.01587	1174.659072	987.7666025	1244.507935
2674278	---	0.125	1567.981744	1864.655046	1760	2489.01587	1046.502261	1108.730524	1479.977691
3551806	---	0.125	1567.981744	1661.21879	1661.21879	2349.318143	880	932.327523	1479.977691
2899171	HIST1H1E // HIST1H1E	0.25	1661.21879	1244.507935	1108.730524	987.7666025	2489.01587	2489.01587	2217.461048
3159040	RPL8	0.375	1661.21879	1479.977691	1244.507935	1244.507935	1760	1661.21879	3322.437581
3395416	SNORD14D // SNORD14C // HSPA8	0.21875	1661.21879	1479.977691	1661.21879	1975.533205	1318.510228	2793.825851	2959.955382
4090948	---	0.375	1661.21879	1567.981744	1864.655046	1108.730524	2793.825851	2489.01587	698.4564629
2436149	---	0.375	1661.21879	1975.533205	1244.507935	1760	1108.730524	1567.981744	3322.437581
2908502	---	0.21875	1661.21879	1864.655046	1567.981744	2349.318143	493.8833013	987.7666025	1975.533205
3212308	---	0.125	1661.21879	1975.533205	1396.912926	1975.533205	1108.730524	1396.912926	2349.318143
3416496	---	0.21875	1661.21879	1864.655046	1567.981744	2093.004522	523.2511306	880	1975.533205
3556588	---	0.375	1661.21879	1396.912926	1108.730524	932.327523	1760	1661.21879	3135.963488
3720121	---	0.125	1661.21879	1567.981744	1318.510228	1108.730524	1975.533205	2637.020455	1760
3867237	---	0.21875	1661.21879	1479.977691	1318.510228	1046.502261	2637.020455	2489.01587	1760
3725876	---	0.1875	1661.21879	2093.004522	1760	2637.020455	1174.659072	1174.659072	1567.981744
3844055	---	0.125	1661.21879	1760	2093.004522	2349.318143	1108.730524	987.7666025	1661.21879
3867223	RPL18	0.1875	1760	1567.981744	1396.912926	1174.659072	2637.020455	2489.01587	1975.533205
2840628	---	0.21875	1760	1760	1975.533205	2217.461048	440	987.7666025	1760
2840631	---	0.1875	1760	1661.21879	1864.655046	2217.461048	493.8833013	1396.912926	2093.004522
2840633	---	0.125	1760	1661.21879	1760	2093.004522	783.990872	1567.981744	2217.461048
3255510	---	0.1875	1760	2489.01587	1864.655046	1975.533205	698.4564629	1567.981744	1396.912926
3364739	---	0.21875	1760	1567.981744	1174.659072	932.327523	2349.318143	2637.020455	1975.533205
3722311	---	0.1875	1760	1244.507935	1174.659072	987.7666025	1975.533205	2349.318143	2349.318143
2674347	---	0.125	1760	2217.461048	1864.655046	2637.020455	1318.510228	1479.977691	1567.981744
2840766	---	0.1875	1760	1864.655046	2637.020455	2349.318143	1318.510228	1396.912926	1396.912926
3096474	---	0.125	1760	2093.004522	1975.533205	2637.020455	1174.659072	1318.510228	1760

3556270	---	0.125	1760	2093.004522	2093.004522	2637.020455	1318.510228	1318.510228	1567.981744
2395490	ENO1 // ENO1	0.125	1864.655046	1108.730524	2093.004522	2489.01587	1479.977691	1567.981744	2093.004522
3416489	---	0.375	1864.655046	2093.004522	1975.533205	1975.533205	329.6275569	659.2551138	1975.533205
3630185	---	0.375	1864.655046	2217.461048	2349.318143	2793.825851	932.327523	1244.507935	1174.659072
3816394	---	0.1875	1864.655046	1567.981744	932.327523	1174.659072	1661.21879	1760	2637.020455
3816399	---	0.25	1864.655046	1479.977691	932.327523	880	2093.004522	1975.533205	2793.825851
2318817	---	0.1875	1864.655046	1760	2959.955382	2489.01587	1479.977691	1661.21879	1975.533205
2950329	HLA-DPA1 // HLA-DPA1	0.125	1975.533205	1760	1864.655046	1661.21879	2637.020455	1760	2793.825851
4119114	---	0.4375	1975.533205	1864.655046	1975.533205	1396.912926	3135.963488	2959.955382	987.7666025
2738919	---	0.125	1975.533205	2489.01587	1864.655046	1864.655046	1760	1975.533205	932.327523
2844217	---	0.75	1975.533205	1396.912926	1479.977691	1396.912926	2349.318143	2637.020455	3729.310092
2908489	---	0.25	1975.533205	1975.533205	1975.533205	2637.020455	783.990872	1567.981744	2637.020455
2908497	---	0.4375	1975.533205	2093.004522	1864.655046	2637.020455	739.9888454	1396.912926	3135.963488
3816398	---	0.125	1975.533205	1661.21879	932.327523	1318.510228	1975.533205	1975.533205	2489.01587
3275204	---	0.1875	1975.533205	1975.533205	2093.004522	2637.020455	1108.730524	1244.507935	2093.004522
3275206	---	0.21875	1975.533205	2217.461048	1975.533205	2637.020455	1108.730524	1046.502261	2093.004522
3551822	---	0.125	1975.533205	2217.461048	2093.004522	2959.955382	1661.21879	1760	1864.655046
3740566	---	0.1875	1975.533205	2349.318143	2217.461048	3135.963488	1661.21879	1661.21879	1661.21879
3773932	ACTG1 // ACTG1	0.125	2093.004522	1396.912926	1760	2093.004522	1975.533205	2793.825851	2349.318143
7385515	MALAT1	0.21875	2093.004522	2093.004522	2093.004522	2349.318143	830.6093952	2217.461048	1046.502261
2775498	---	0.1875	2093.004522	1975.533205	2959.955382	2349.318143	1479.977691	1760	1479.977691
3319311	---	0.125	2093.004522	1661.21879	1661.21879	1864.655046	2093.004522	2637.020455	2793.825851
3319313	---	0.125	2093.004522	1760	1567.981744	1864.655046	2093.004522	2793.825851	2637.020455
3371639	---	0.125	2093.004522	2793.825851	2217.461048	1760	1975.533205	1864.655046	1318.510228
3842154	---	0.375	2093.004522	2349.318143	2217.461048	2349.318143	587.3295358	1046.502261	2093.004522
2536418	---	0.125	2093.004522	2489.01587	2349.318143	2959.955382	1479.977691	1661.21879	1975.533205
3392916	---	0.125	2093.004522	2349.318143	2217.461048	2793.825851	1396.912926	1567.981744	2093.004522
2439842	TAGLN2	0.75	2217.461048	2217.461048	1318.510228	1174.659072	1760	2093.004522	3729.310092
3322775	LDHA	0.1875	2217.461048	1108.730524	2217.461048	2217.461048	1864.655046	2489.01587	2793.825851
2841289	---	1.75	2217.461048	1396.912926	880	783.990872	2793.825851	2637.020455	3520
3416498	---	0.1875	2217.461048	2349.318143	2349.318143	2489.01587	1174.659072	1567.981744	2793.825851
2840760	---	0.125	2217.461048	2489.01587	2793.825851	2793.825851	1760	1760	1760

3275348	---	0.125	2217.461048	2489.01587	2349.318143	2793.825851	1396.912926	1567.981744	2217.461048
3275350	---	0.125	2217.461048	2489.01587	2349.318143	2793.825851	1567.981744	1567.981744	2349.318143
3699858	---	0.125	2217.461048	2349.318143	2349.318143	2959.955382	1661.21879	1760	1975.533205
3416492	---	0.25	2349.318143	2637.020455	2489.01587	2959.955382	1174.659072	1479.977691	2349.318143
3722308	---	0.1875	2349.318143	1975.533205	1567.981744	1567.981744	2637.020455	2793.825851	2793.825851
2831979	---	0.1875	2349.318143	2637.020455	2489.01587	3322.437581	1760	1864.655046	2349.318143
2844323	---	0.125	2349.318143	2637.020455	2637.020455	3135.963488	1760	1975.533205	2217.461048
3809794	---	0.125	2349.318143	2349.318143	2489.01587	3135.963488	1975.533205	1864.655046	1975.533205
4037669	---	0.125	2489.01587	1975.533205	2093.004522	2093.004522	2489.01587	2489.01587	3322.437581
3472096	---	0.125	2489.01587	2637.020455	2793.825851	2959.955382	1567.981744	2093.004522	2489.01587
3816400	---	0.5	2489.01587	1975.533205	1396.912926	1318.510228	2349.318143	2489.01587	3729.310092
3842146	---	0.125	2489.01587	2959.955382	2637.020455	2637.020455	1567.981744	2093.004522	1975.533205
3842151	---	0.125	2489.01587	2793.825851	2489.01587	2637.020455	1396.912926	2217.461048	2637.020455
2536452	---	0.125	2489.01587	2637.020455	2793.825851	3322.437581	1864.655046	2093.004522	2349.318143
2840758	---	0.21875	2489.01587	2489.01587	3135.963488	3135.963488	1975.533205	1975.533205	1567.981744
3475126	---	0.125	2489.01587	2793.825851	2637.020455	3322.437581	1975.533205	2093.004522	2349.318143
3556280	---	0.1875	2489.01587	2637.020455	2637.020455	3135.963488	1661.21879	1864.655046	2637.020455
3725938	---	0.125	2489.01587	2793.825851	2637.020455	3322.437581	1975.533205	2093.004522	2349.318143
3740592	---	0.125	2489.01587	2637.020455	2637.020455	3322.437581	1975.533205	2093.004522	2349.318143
3819693	---	0.125	2489.01587	2637.020455	2637.020455	3135.963488	1760	1864.655046	2489.01587
4037595	---	0.125	2637.020455	2093.004522	2217.461048	2217.461048	3135.963488	2637.020455	3322.437581
4074465	---	0.4375	2637.020455	2489.01587	2793.825851	2093.004522	3520	3520	1318.510228
4129546	---	0.4375	2637.020455	2637.020455	2637.020455	2093.004522	3729.310092	3729.310092	1661.21879
4134740	---	0.4375	2637.020455	2637.020455	2489.01587	1864.655046	3520	3520	1479.977691
2908498	---	1.75	2637.020455	2959.955382	2489.01587	3322.437581	1046.502261	1661.21879	4186.009045
3630187	---	0.75	2637.020455	3135.963488	3322.437581	3729.310092	1318.510228	1864.655046	1975.533205
3816396	---	0.1875	2637.020455	2217.461048	1567.981744	1760	2489.01587	2489.01587	3135.963488
3036924	ACTB	0.4375	2793.825851	2093.004522	739.9888454	2959.955382	2637.020455	2489.01587	2959.955382
3416491	---	0.875	2793.825851	2959.955382	2637.020455	3322.437581	783.990872	1567.981744	2959.955382
3475052	---	0.125	2793.825851	2959.955382	2959.955382	3729.310092	2349.318143	2217.461048	2959.955382
4037583	---	0.21875	2959.955382	2637.020455	2959.955382	3322.437581	2793.825851	2959.955382	1479.977691
3842152	---	0.125	2959.955382	3135.963488	3322.437581	3135.963488	1975.533205	2349.318143	2959.955382
4097642	---	0.21875	3520	3520	3520	3135.963488	3729.310092	3951.06641	2093.004522
3453474	---	0.125	3729.310092	3729.310092	3729.310092	4186.009045	2959.955382	3135.963488	4186.009045
4091084	---	0.1875	3951.06641	3951.06641	3951.06641	3520	4186.009045	4186.009045	2637.020455
4128085	---	0.4375	3951.06641	3951.06641	3951.06641	3520	4186.009045	4186.009045	1975.533205

Supplementary Table S6.

Musical interpretation of top differentially expressed probe sets from "neuroblastoma" cell lines (Song of Joy). DNA microarray data from 4 cell lines that were initially established as neuroblastoma cell lines (GSE1824) were transformed into melodies by using the following parameters: minimal frequency: 27.5; number of different frequencies: 88 (keys); number of tone steps per octave: 12; minimal duration: 1/8; number of tones: 63. Probe sets were sorted ascending according to the calculated frequencies from the median signal intensities. Thereafter, Beethoven's "Song of Joy" from the 9th Symphony was used for re-calibration of the frequencies. Presented are the frequencies of the individual samples and the frequency of the median signal intensity as well as the duration of the filtered 63 probe sets. The durations were re-calibrated to Beethoven's original melody (crotchet = 1).

Probe Set ID	Gene Symbol	Median	CHP-126	SH-SY5Y	SiMa	SK-N-MC	Duration (Original)
206326_at	GRP	184.9972114	184.9972114	184.9972114	184.9972114	3322.437581	1
202747_s_at	ITM2A	184.9972114	195.997718	184.9972114	174.6141157	2959.955382	1
206463_s_at	DHRS2	195.997718	2793.825851	184.9972114	184.9972114	174.6141157	1
212097_at	CAV1	220	87.30705786	493.8833013	97.998859	3322.437581	1
201160_s_at	CSDA	220	246.9416506	55	184.9972114	1975.533205	1
205827_at	CCK	195.997718	246.9416506	146.832384	51.9130872	2489.01587	1
202746_at	ITM2A	184.9972114	195.997718	174.6141157	103.8261744	1567.981744	1
214079_at	DHRS2	164.8137785	1661.21879	130.8127827	195.997718	110	1
202403_s_at	COL1A2	146.832384	77.78174593	184.9972114	116.5409404	1244.507935	1
203065_s_at	CAV1	146.832384	58.27047019	220	92.49860568	1318.510228	1
201012_at	ANXA1	164.8137785	43.65352893	174.6141157	146.832384	1046.502261	1
201909_at	RPS4Y1	184.9972114	164.8137785	123.4708253	1244.507935	195.997718	1
205542_at	STEAP1	184.9972114	51.9130872	184.9972114	184.9972114	1108.730524	1.5
213921_at	SST	164.8137785	277.182631	46.24930284	1760	92.49860568	0.5
213791_at	PENK	164.8137785	123.4708253	155.5634919	174.6141157	1479.977691	2
218559_s_at	MAFB	184.9972114	164.8137785	195.997718	38.89087297	1046.502261	1
202431_s_at	MYC	184.9972114	82.40688923	391.995436	55	659.2551138	1
210302_s_at	MAB21L2	195.997718	932.327523	146.832384	246.9416506	77.78174593	1
218831_s_at	FCGRT	220	184.9972114	246.9416506	65.40639133	932.327523	1
201596_x_at	KRT18	220	123.4708253	311.1269837	146.832384	783.990872	1
213847_at	PRPH	195.997718	698.4564629	103.8261744	329.6275569	116.5409404	1
221011_s_at	LBH	184.9972114	164.8137785	195.997718	164.8137785	659.2551138	1
221728_x_at	XIST	164.8137785	61.73541266	369.9944227	69.29565774	391.995436	1

209757_s_at	MYCN	146.832384	466.1637615	58.27047019	349.2282314	48.9994295	1
210839_s_at	ENPP2	146.832384	293.6647679	73.41619198	329.6275569	38.89087297	1
212188_at	KCTD12	164.8137785	369.9944227	311.1269837	27.5	82.40688923	1
218353_at	RGS5	184.9972114	369.9944227	622.2539674	87.30705786	65.40639133	1
202409_at	IGF2 /// INS-IGF2	164.8137785	184.9972114	146.832384	493.8833013	77.78174593	1.5
205113_at	NEFM	146.832384	130.8127827	155.5634919	466.1637615	12.9782718	0.5
219449_s_at	TMEM70	146.832384	138.5913155	146.832384	138.5913155	466.1637615	2
201590_x_at	ANXA2	164.8137785	65.40639133	349.2282314	73.41619198	440	1
209392_at	ENPP2	164.8137785	311.1269837	87.30705786	329.6275569	27.5	1
201105_at	LGALS1	184.9972114	123.4708253	415.3046976	65.40639133	261.6255653	1
203423_at	RBP1	146.832384	13.75	233.0818808	311.1269837	87.30705786	1
209071_s_at	RGS5	164.8137785	329.6275569	440	77.78174593	48.9994295	1
209560_s_at	DLK1	184.9972114	138.5913155	233.0818808	659.2551138	41.20344461	0.5
210427_x_at	ANXA2	195.997718	92.49860568	415.3046976	82.40688923	523.2511306	0.5
213503_x_at	ANXA2	184.9972114	87.30705786	349.2282314	58.27047019	466.1637615	1
221916_at	NEFL	146.832384	164.8137785	123.4708253	277.182631	16.35159783	1
204260_at	CHGB	164.8137785	233.0818808	103.8261744	311.1269837	16.35159783	1
209070_s_at	RGS5	184.9972114	391.995436	523.2511306	87.30705786	21.82676446	0.5
209987_s_at	ASCL1	195.997718	311.1269837	277.182631	130.8127827	16.35159783	0.5
212552_at	HPCAL1	184.9972114	146.832384	233.0818808	415.3046976	103.8261744	1
209988_s_at	ASCL1	164.8137785	311.1269837	233.0818808	110	29.13523509	1
204339_s_at	RGS4	146.832384	155.5634919	220	123.4708253	17.32391444	1
209841_s_at	LRRN3	164.8137785	277.182631	207.6523488	130.8127827	32.70319566	1
203000_at	STMN2	110	82.40688923	174.6141157	138.5913155	12.9782718	1
203001_s_at	STMN2	184.9972114	130.8127827	311.1269837	246.9416506	8.175798916	2
205311_at	DDC	184.9972114	184.9972114	184.9972114	311.1269837	9.177023997	1
209840_s_at	LRRN3	195.997718	277.182631	246.9416506	146.832384	27.5	1
204697_s_at	CHGA	220	207.6523488	220	349.2282314	24.49971475	1
219791_s_at	NBLA00301	220	369.9944227	311.1269837	146.832384	8.175798916	1
204337_at	RGS4	195.997718	220	246.9416506	174.6141157	25.9565436	1
212233_at	MAP1B	184.9972114	233.0818808	164.8137785	195.997718	73.41619198	1
201426_s_at	VIM	195.997718	246.9416506	277.182631	155.5634919	138.5913155	0.5
207009_at	PHOX2B	164.8137785	233.0818808	155.5634919	164.8137785	9.722718241	0.5
209604_s_at	GATA3	146.832384	174.6141157	164.8137785	130.8127827	29.13523509	1
200790_at	ODC1	146.832384	155.5634919	138.5913155	311.1269837	138.5913155	1

201241_at	DDX1	164.8137785	349.2282314	97.998859	277.182631	97.998859	1
209771_x_at	CD24	184.9972114	195.997718	184.9972114	174.6141157	69.29565774	1
205967_at	HIST1H4A /// HIST1H4B /// HIST1H4C /// HIST1H4D /// HIST1H4E /// HIST1H4F /// HIST1H4H /// HIST1H4I /// HIST1H4J /// HIST1H4K /// HIST1H4L /// HIST2H4A /// HIST2H4B /// HIST4H4	164.8137785	146.832384	110	246.9416506	174.6141157	1.5
216379_x_at	CD24	146.832384	164.8137785	146.832384	130.8127827	46.24930284	0.5
207783_x_at	HUWE1	146.832384	155.5634919	146.832384	92.49860568	138.5913155	2

Supplementary Table S7.

Musical interpretation of top differentially expressed probe sets from "neuroblastoma" cell lines (Ride of the Valkyries). DNA microarray data from 4 cell lines that were initially established as neuroblastoma cell lines were transformed into melodies by using the following parameters: minimal frequency: 27.5; number of different frequencies: 88; number of tone steps per octave: 12; minimal duration: 1/8; number of tones: 86. Probe sets were sorted ascending according to the calculated frequencies from the median signal intensities. Thereafter, Wagner's "Ride of the Valkyries" was used for re-calibration of the frequencies. Presented are the frequencies of the individual samples and the frequency of the median signal intensity as well as the duration of the filtered 86 probe sets. The durations were recalibrated to Wagner's original melody (crotchet = 1).

Probe Set ID	Gene Symbol	Median	CHP-126	SH-SY5Y	SiMa	SK-N-MC	Duration (Original)
206326_at	GRP	92.49860568	92.49860568	92.49860568	92.49860568	1661.21879	0.5
202747_s_at	ITM2A	123.4708253	130.8127827	123.4708253	116.5409404	1975.533205	0.75
206463_s_at	DHRS2	92.49860568	1318.510228	87.30705786	87.30705786	82.40688923	0.25
212097_at	CAV1	123.4708253	48.9994295	277.182631	55	1864.655046	0.5
201160_s_at	CSDA	155.5634919	174.6141157	38.89087297	130.8127827	1396.912926	1.5
205827_at	CCK	123.4708253	155.5634919	92.49860568	32.70319566	1567.981744	1.5
202746_at	ITM2A	155.5634919	164.8137785	146.832384	87.30705786	1318.510228	0.75
214079_at	DHRS2	123.4708253	1244.507935	97.998859	146.832384	82.40688923	0.25
202403_s_at	COL1A2	155.5634919	82.40688923	195.997718	123.4708253	1318.510228	0.5
203065_s_at	CAV1	184.9972114	73.41619198	277.182631	116.5409404	1661.21879	1.5
209291_at	ID4	155.5634919	55	207.6523488	123.4708253	987.7666025	1.5
200953_s_at	CCND2	184.9972114	220	130.8127827	155.5634919	1108.730524	0.75
201012_at	ANXA1	155.5634919	41.20344461	164.8137785	138.5913155	987.7666025	0.25
201909_at	RPS4Y1	184.9972114	164.8137785	123.4708253	1244.507935	195.997718	0.5
205542_at	STEAP1	233.0818808	65.40639133	233.0818808	233.0818808	1396.912926	1.5
213921_at	SST	116.5409404	195.997718	32.70319566	1244.507935	65.40639133	1.5
210095_s_at	IGFBP3	155.5634919	138.5913155	830.6093952	82.40688923	174.6141157	0.75
213791_at	PENK	116.5409404	87.30705786	110	123.4708253	1046.502261	0.25
201667_at	GJA1	155.5634919	97.998859	233.0818808	69.29565774	739.9888454	0.5
202410_x_at	IGF2 /// INS-IGF2	184.9972114	195.997718	174.6141157	830.6093952	30.86770633	2.5
218559_s_at	MAFB	116.5409404	103.8261744	123.4708253	24.49971475	659.2551138	0.5
202431_s_at	MYC	155.5634919	69.29565774	329.6275569	46.24930284	554.365262	0.75

210302_s_at	MAB21L2	116.5409404	554.365262	87.30705786	146.832384	46.24930284	0.25
206163_at	MAB21L1	155.5634919	440	391.995436	58.27047019	24.49971475	0.5
218831_s_at	FCGRT	184.9972114	155.5634919	207.6523488	55	783.990872	1.5
201596_x_at	KRT18	155.5634919	87.30705786	220	103.8261744	554.365262	1.5
213847_at	PRPH	184.9972114	659.2551138	97.998859	311.1269837	110	0.75
221011_s_at	LBH	155.5634919	138.5913155	164.8137785	138.5913155	554.365262	0.25
221728_x_at	XIST	184.9972114	69.29565774	415.3046976	77.78174593	440	0.5
209757_s_at	MYCN	233.0818808	739.9888454	92.49860568	554.365262	77.78174593	1.5
210839_s_at	ENPP2	184.9972114	369.9944227	92.49860568	415.3046976	48.9994295	1.5
212188_at	KCTD12	233.0818808	523.2511306	440	38.89087297	116.5409404	0.75
218353_at	RGS5	184.9972114	369.9944227	622.2539674	87.30705786	65.40639133	0.25
202409_at	IGF2 /// INS-IGF2	233.0818808	261.6255653	207.6523488	698.4564629	110	0.5
211959_at	IGFBP5	277.182631	659.2551138	440	92.49860568	164.8137785	1.5
205113_at	NEFM	138.5913155	123.4708253	146.832384	440	12.24985737	1.5
207076_s_at	ASS1	184.9972114	123.4708253	19.44543648	391.995436	277.182631	0.75
215076_s_at	COL3A1	138.5913155	130.8127827	293.6647679	23.12465142	138.5913155	0.25
201852_x_at	COL3A1	184.9972114	184.9972114	369.9944227	15.43385316	174.6141157	0.5
204086_at	PRAME	233.0818808	440	415.3046976	123.4708253	130.8127827	2.5
219449_s_at	TMEM70	92.49860568	87.30705786	92.49860568	87.30705786	293.6647679	0.5
201590_x_at	ANXA2	103.8261744	41.20344461	220	46.24930284	277.182631	0.75
209392_at	ENPP2	82.40688923	155.5634919	43.65352893	164.8137785	13.75	0.25
201105_at	LGALS1	103.8261744	69.29565774	233.0818808	36.70809599	146.832384	0.5
202465_at	PCOLCE	123.4708253	123.4708253	261.6255653	123.4708253	65.40639133	2.5
203423_at	RBP1	184.9972114	17.32391444	293.6647679	391.995436	110	0.5
209071_s_at	RGS5	207.6523488	415.3046976	554.365262	97.998859	61.73541266	0.75
209560_s_at	DLK1	164.8137785	123.4708253	207.6523488	587.3295358	36.70809599	0.25
210427_x_at	ANXA2	207.6523488	97.998859	440	87.30705786	554.365262	0.5
213503_x_at	ANXA2	246.9416506	116.5409404	466.1637615	77.78174593	622.2539674	2.5
221916_at	NEFL	184.9972114	207.6523488	155.5634919	349.2282314	20.60172231	0.5
204260_at	CHGB	103.8261744	146.832384	65.40639133	195.997718	10.30086115	0.75
209070_s_at	RGS5	82.40688923	174.6141157	233.0818808	38.89087297	9.722718241	0.25
209987_s_at	ASCL1	103.8261744	164.8137785	146.832384	69.29565774	8.661957218	0.5
212552_at	HPCAL1	138.5913155	110	174.6141157	311.1269837	77.78174593	1.5
204035_at	SCG2	92.49860568	130.8127827	61.73541266	130.8127827	19.44543648	1.5
204338_s_at	RGS4	123.4708253	138.5913155	184.9972114	103.8261744	20.60172231	0.75

209988_s_at	ASCL1	92.49860568	174.6141157	130.8127827	61.73541266	16.35159783	0.25
217869_at	HSD17B12	123.4708253	21.82676446	92.49860568	155.5634919	184.9972114	0.5
204339_s_at	RGS4	155.5634919	164.8137785	233.0818808	130.8127827	18.35404799	2.5
204540_at	EEF1A2	92.49860568	110	82.40688923	130.8127827	16.35159783	0.5
209841_s_at	LRRN3	103.8261744	174.6141157	130.8127827	82.40688923	20.60172231	0.75
210950_s_at	FDFT1	82.40688923	77.78174593	77.78174593	82.40688923	164.8137785	0.25
203000_at	STMN2	103.8261744	77.78174593	164.8137785	130.8127827	12.24985737	0.5
203001_s_at	STMN2	123.4708253	87.30705786	207.6523488	164.8137785	5.456691116	2.5
203130_s_at	KIF5C	184.9972114	246.9416506	146.832384	207.6523488	36.70809599	0.5
205311_at	DDC	207.6523488	207.6523488	207.6523488	349.2282314	10.30086115	0.75
209840_s_at	LRRN3	164.8137785	233.0818808	207.6523488	123.4708253	23.12465142	0.25
210221_at	CHRNA3	207.6523488	261.6255653	195.997718	207.6523488	32.70319566	0.5
210547_x_at	ICA1	246.9416506	233.0818808	261.6255653	246.9416506	20.60172231	2.5
220138_at	HAND1	92.49860568	130.8127827	87.30705786	92.49860568	24.49971475	0.5
204697_s_at	CHGA	103.8261744	97.998859	103.8261744	164.8137785	11.56232571	0.75
219791_s_at	NBLA00301	82.40688923	138.5913155	116.5409404	55	3.062464344	0.25
204337_at	RGS4	103.8261744	116.5409404	130.8127827	92.49860568	13.75	0.5
204914_s_at	SOX11	138.5913155	207.6523488	164.8137785	116.5409404	92.49860568	1.5
212233_at	MAP1B	92.49860568	116.5409404	82.40688923	97.998859	36.70809599	1.5
201426_s_at	VIM	123.4708253	155.5634919	174.6141157	97.998859	87.30705786	0.75
207009_at	PHOX2B	92.49860568	130.8127827	87.30705786	92.49860568	5.456691116	0.25
209604_s_at	GATA3	123.4708253	146.832384	138.5913155	110	24.49971475	0.5
200790_at	ODC1	155.5634919	164.8137785	146.832384	329.6275569	146.832384	0.75
201241_at	DDX1	123.4708253	261.6255653	73.41619198	207.6523488	73.41619198	0.25
209771_x_at	CD24	155.5634919	164.8137785	155.5634919	146.832384	58.27047019	0.5
205967_at	HIST1H4A /// HIST1H4B /// HIST1H4C /// HIST1H4D /// HIST1H4E /// HIST1H4F /// HIST1H4H /// HIST1H4I /// HIST1H4J /// HIST1H4K /// HIST1H4L ///	184.9972114	164.8137785	123.4708253	277.182631	195.997718	0.75

	HIST2H4A /// HIST2H4B /// HIST4H4						
212085_at	SLC25A6	155.5634919	73.41619198	155.5634919	155.5634919	155.5634919	0.25
216379_x_at	CD24	184.9972114	207.6523488	184.9972114	164.8137785	58.27047019	0.5
207783_x_at	HUWE1	246.9416506	261.6255653	246.9416506	155.5634919	233.0818808	4

Supplementary Table S8.

Indiviual results from the interview from Figure 7. 23 Individuals were asked to identify the outlier in Figure 1A ("Diagram Top 192"), Figure 3A ("Diagram EFT specific"), and Figure 6 ("Diagram Song of Joy"). Thereafter, the same individuals were asked to identify the outlier among the corresponding melodies ("Sound Top 192","Sound EFT specific", "Diagram Song of Joy". For technical reasons the individuals were interviewed in two independent groups (exp1 and exp2). Correct answers were indicated in bold face.

No.	Group	Diagram Top 192	Sound Top 192	Diagram EFT specific	Sound EFT specific	Diagram Song of Joy	Sound Song of Joy
1	exp1	**SK-N-MC**	CHP-126	**SK-N-MC**	**SK-N-MC**	SiMa	**SK-N-MC**
2	exp1	SH-SY5Y	**SK-N-MC**	**SK-N-MC**	SiMa	CHP-126	SiMa
3	exp1	**SK-N-MC**	CHP-126	SiMa	**SK-N-MC**	**SK-N-MC**	**SK-N-MC**
4	exp1	**SK-N-MC**	**SK-N-MC**	SH-SY5Y	**SK-N-MC**	**SK-N-MC**	**SK-N-MC**
5	exp1	CHP-126	**SK-N-MC**	**SK-N-MC**	**SK-N-MC**	**SK-N-MC**	**SK-N-MC**
6	exp1	SH-SY5Y	**SK-N-MC**	SH-SY5Y	**SK-N-MC**	**SK-N-MC**	**SK-N-MC**
7	exp1	**SK-N-MC**	SiMa	**SK-N-MC**	**SK-N-MC**	**SK-N-MC**	**SK-N-MC**
8	exp1	**SK-N-MC**	SH-SY5Y	**SK-N-MC**	SiMa	**SK-N-MC**	SiMa
9	exp1	**SK-N-MC**	**SK-N-MC**	**SK-N-MC**	**SK-N-MC**	**SK-N-MC**	**SK-N-MC**
10	exp1	**SK-N-MC**	**SK-N-MC**	**SK-N-MC**	**SK-N-MC**	**SK-N-MC**	no answer
11	exp1	**SK-N-MC**	SiMa	SH-SY5Y	SiMa	**SK-N-MC**	no answer
12	exp1	**SK-N-MC**	**SK-N-MC**	**SK-N-MC**	**SK-N-MC**	**SK-N-MC**	**SK-N-MC**
13	exp1	**SK-N-MC**	SH-SY5Y	**SK-N-MC**	**SK-N-MC**	**SK-N-MC**	**SK-N-MC**
14	exp1	**SK-N-MC**	no answer	**SK-N-MC**	no answer	**SK-N-MC**	**SK-N-MC**
15	exp2	**SK-N-MC**	SiMa	**SK-N-MC**	**SK-N-MC**	**SK-N-MC**	**SK-N-MC**
16	exp2	SH-SY5Y	**SK-N-MC**	**SK-N-MC**	SH-SY5Y	**SK-N-MC**	**SK-N-MC**
17	exp2	**SK-N-MC**	SH-SY5Y	**SK-N-MC**	**SK-N-MC**	**SK-N-MC**	**SK-N-MC**
18	exp2	**SK-N-MC**	**SK-N-MC**	**SK-N-MC**	**SK-N-MC**	**SK-N-MC**	**SK-N-MC**
19	exp2	SH-SY5Y	**SK-N-MC**	SiMa	**SK-N-MC**	**SK-N-MC**	**SK-N-MC**
20	exp2	**SK-N-MC**	SiMa	**SK-N-MC**	SiMa	**SK-N-MC**	**SK-N-MC**
21	exp2	**SK-N-MC**	**SK-N-MC**	**SK-N-MC**	**SK-N-MC**	**SK-N-MC**	**SK-N-MC**
22	exp2	**SK-N-MC**	no answer	**SK-N-MC**	**SK-N-MC**	**SK-N-MC**	**SK-N-MC**
23	exp2	**SK-N-MC**	**SK-N-MC**	**SK-N-MC**	**SK-N-MC**	**SK-N-MC**	**SK-N-MC**

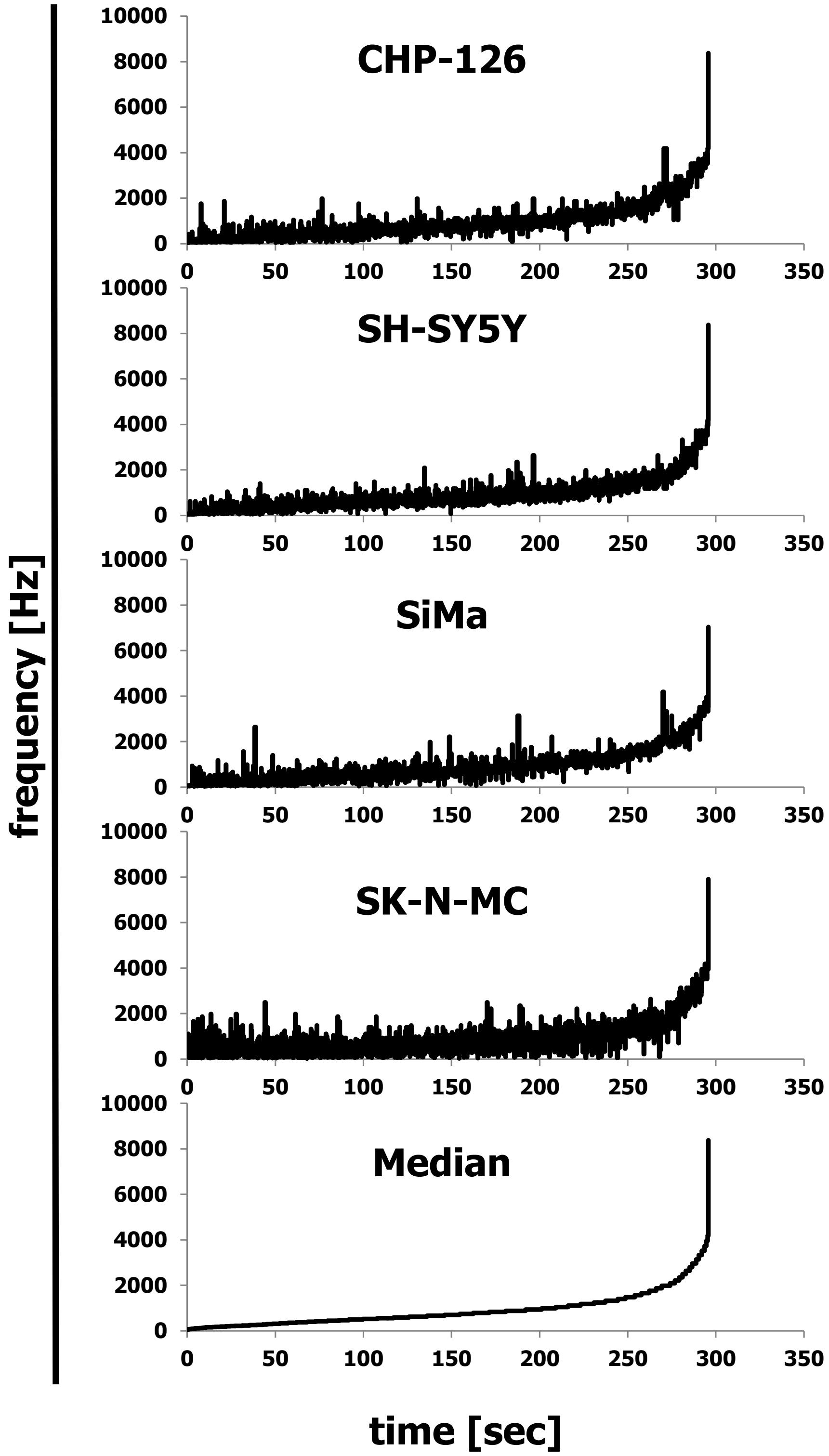

Supplementary Figure S1: Frequency courses for the musically interpreted microarray data from "neuroblastoma" cell lines. DNA microarray data from 4 cell lines that were initially established as neuroblastoma cell lines (GSE1824) were transformed into melodies as described in the Methods section by using the following parameters: minimal frequency: 27.5; number of different frequencies (keys): 88; number of tone steps per octave: 12; minimal duration: 1/8; number of tones: 2,228. Presented are the frequencies of the individual cell lines and the frequency of the median signal intensity as a function of time.

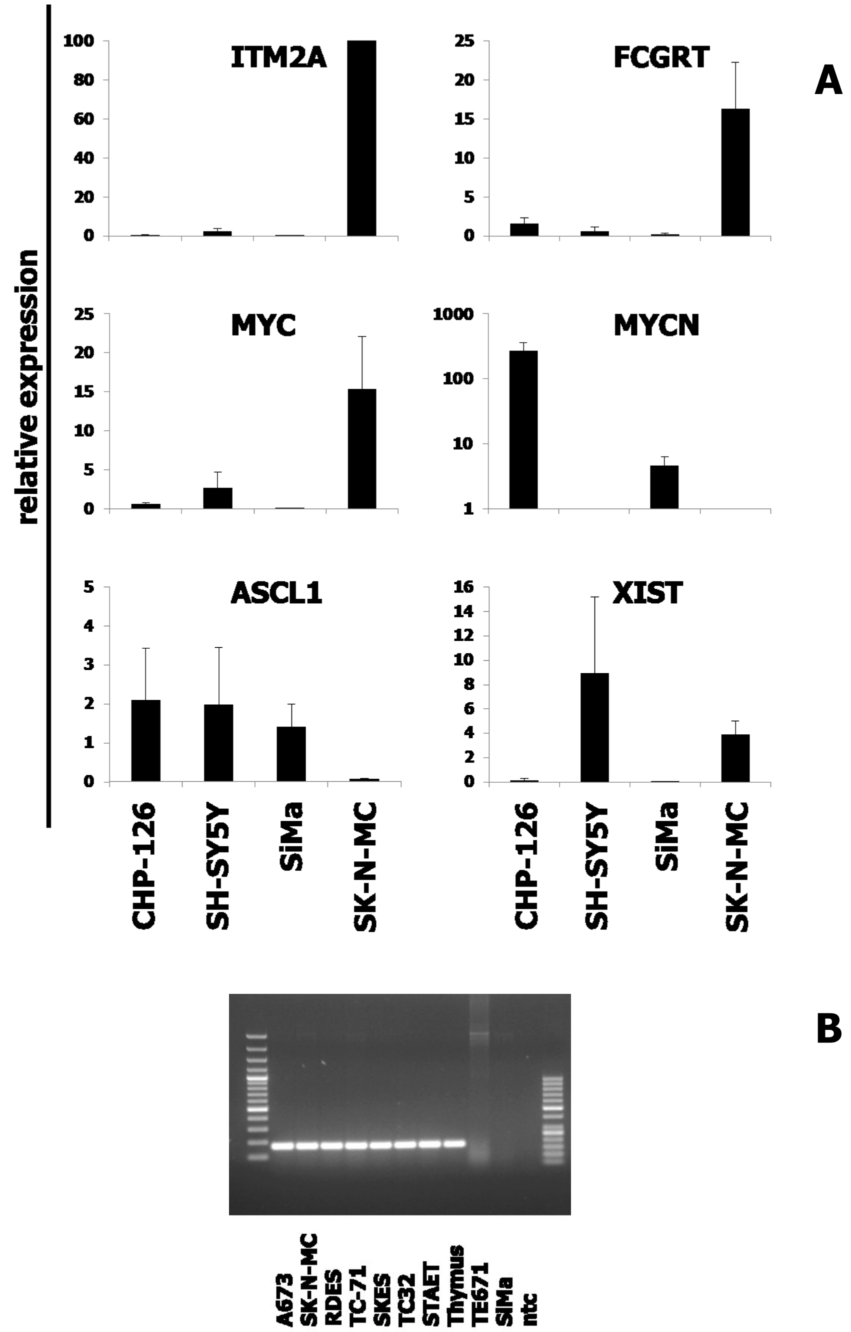

Supplementary Figure S2: Validation of differentially expressed genes from the "neuroblastoma" data. A) Expression of the indicated genes in the different cell lines was assessed by qRT-PCR. Presented are means and standard deviations from three experiments. For comparative analysis, TBP was used as house-keeping control and the median of all Ct values from a given primer combination was set as 1. **B)** Expression of ITM2A in the indicated cell lines and normal thymus was assessed by conventional PCR. All Ewing sarcoma cell lines expressed ITM2A whereas ITM2A was undetectable in TE671 cells (rhabdomyosarcoma) or SiMa cells (neuroblastoma). Thymus served as positive control.

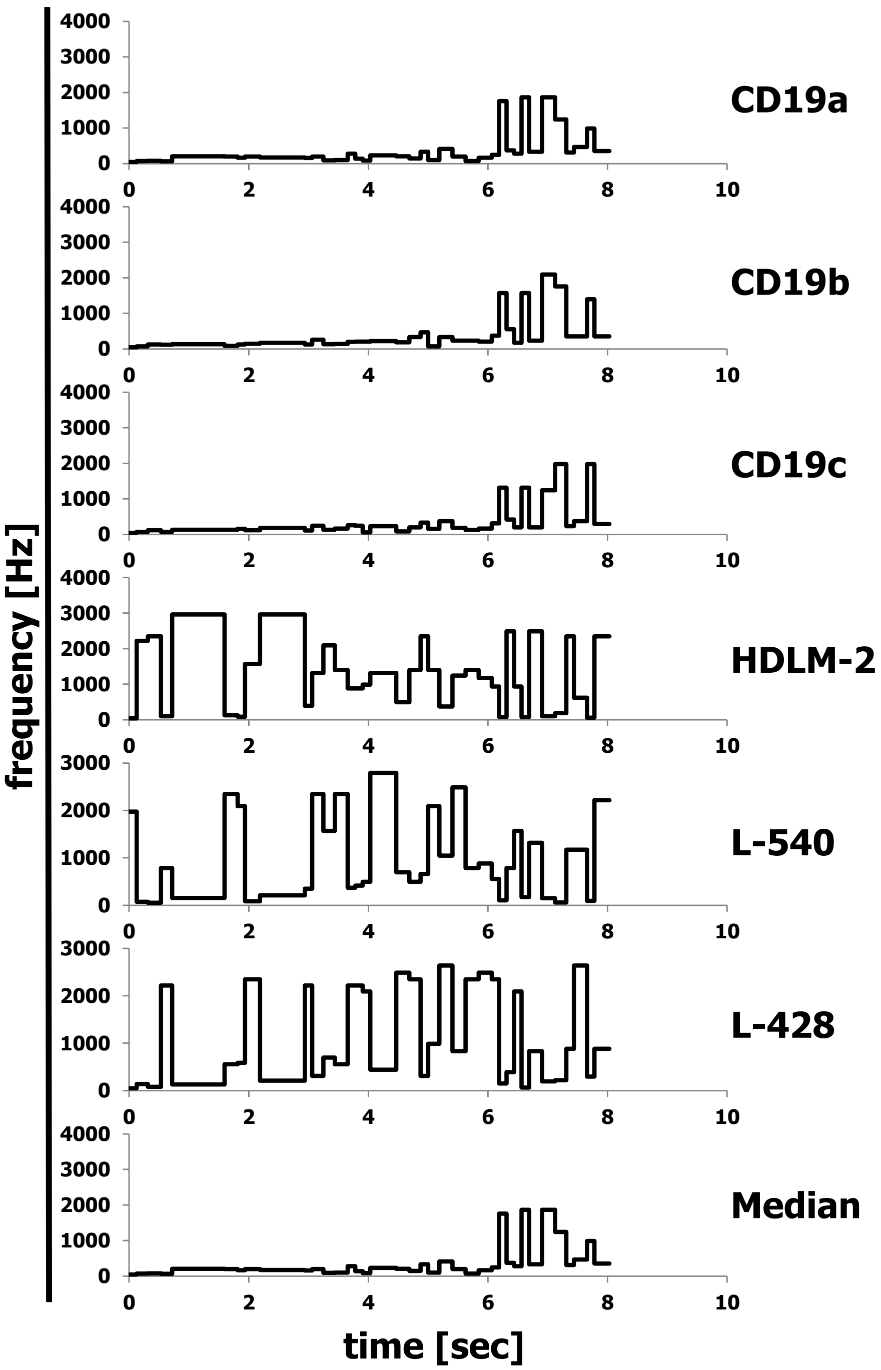

Supplementary Figure 3: Musical interpretation of differentially expressed probe sets from Hodgkin's lymphoma cell lines and normal B cells.
Affymetrix Human Exon 1.0ST microarray data (extended exon level) from 3 Hodgkin's lymphoma cell lines and three CD19 positive B cell samples (from GSE20200) were transformed into melodies by using the following parameters: minimal frequency: 27.5; number of different frequencies: 88; number of tone steps per octave: 12; minimal duration: 1/8; number of tones: 288. Presented are the frequencies of the individual cell lines and the frequency of the median signal intensity as a function of time. Only the first 8 seconds are displayed.

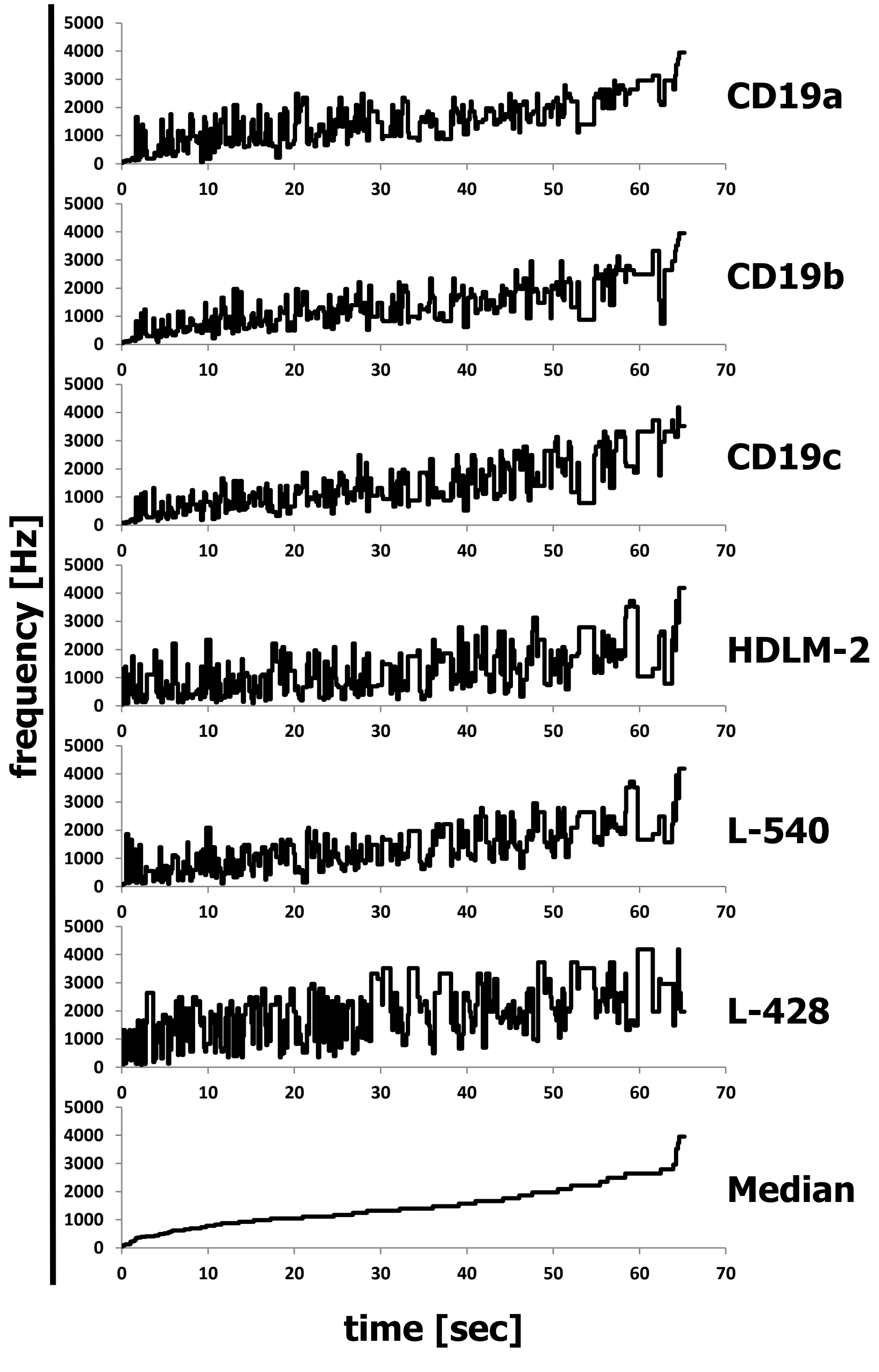

Supplementary Figure 4: Musical interpretation of differentially expressed probe sets from Hodgkin's lymphoma cell lines and normal B cells. Affymetrix Human Exon 1.0ST microarray data (core gene level) from 3 Hodgkin's lymphoma cell lines and three CD19-positive B cell samples (from GSE GSE20200) were transformed into melodies by using the following parameters: minimal frequency: 27.5; number of different frequencies: 88; number of tone steps per octave: 12; minimal duration: 1/8; number of tones: 288. Presented are the frequencies of the individual cell lines and the frequency of the median signal intensity as a function of time.

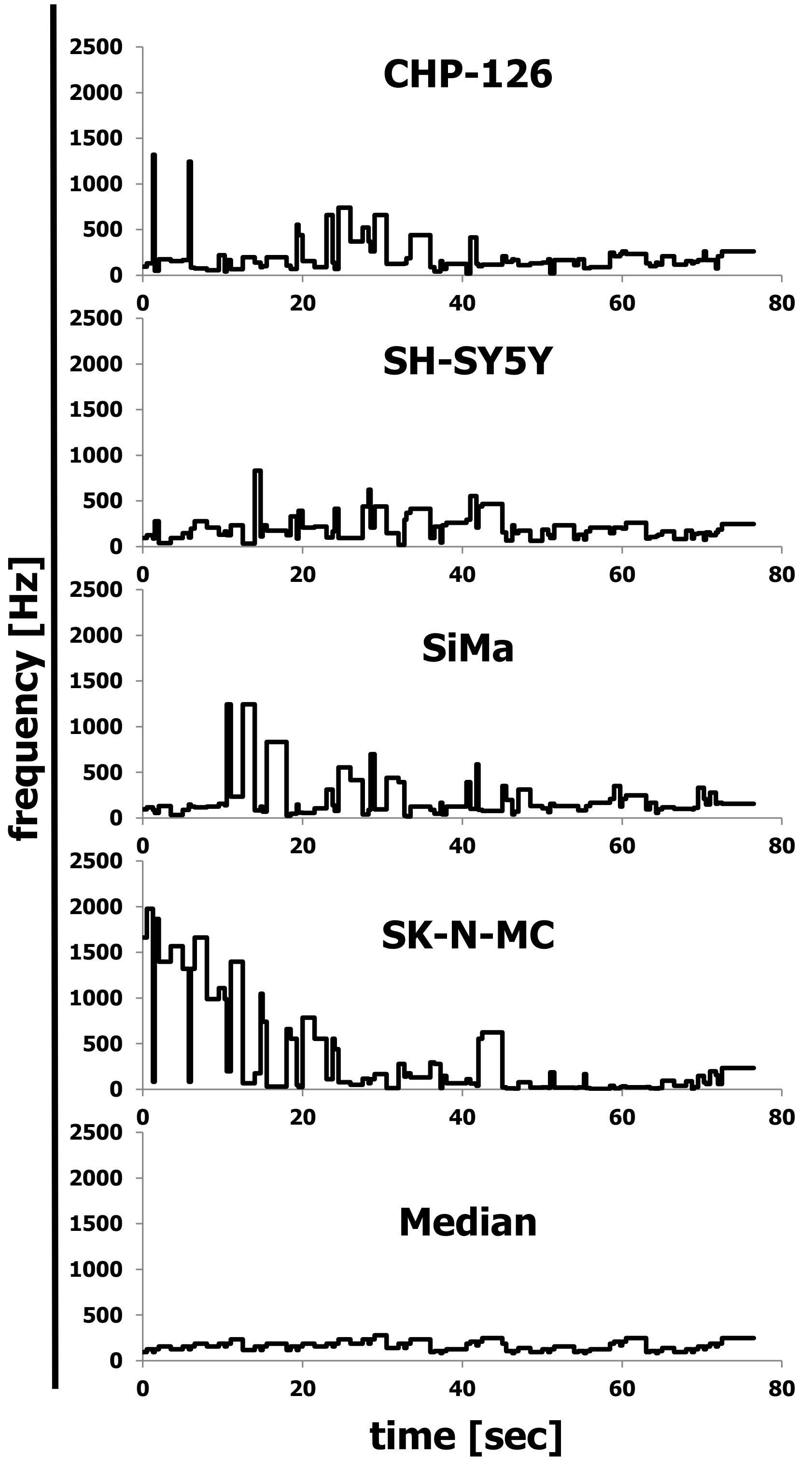

Supplementary Figure 5: Musical interpretation of top differentially expressed probe sets from "neuroblastoma" cell lines (Ride of the Valkyries). DNA microarray data from 4 cell lines that were initially established as neuroblastoma cell lines were transformed into melodies by using the following parameters: minimal frequency: 27.5; number of different frequencies: 88; number of tone steps per octave: 12; minimal duration: 1/8; number of tones: 86. Probe sets were sorted ascending according to the calculated frequencies from the median signal intensities. Thereafter, Wagner's "Ride of the Valkyries" was used for re-calibration of the frequencies. Presented are the frequencies of the individual cell lines and the frequency of the median signal intensity as a function of time.

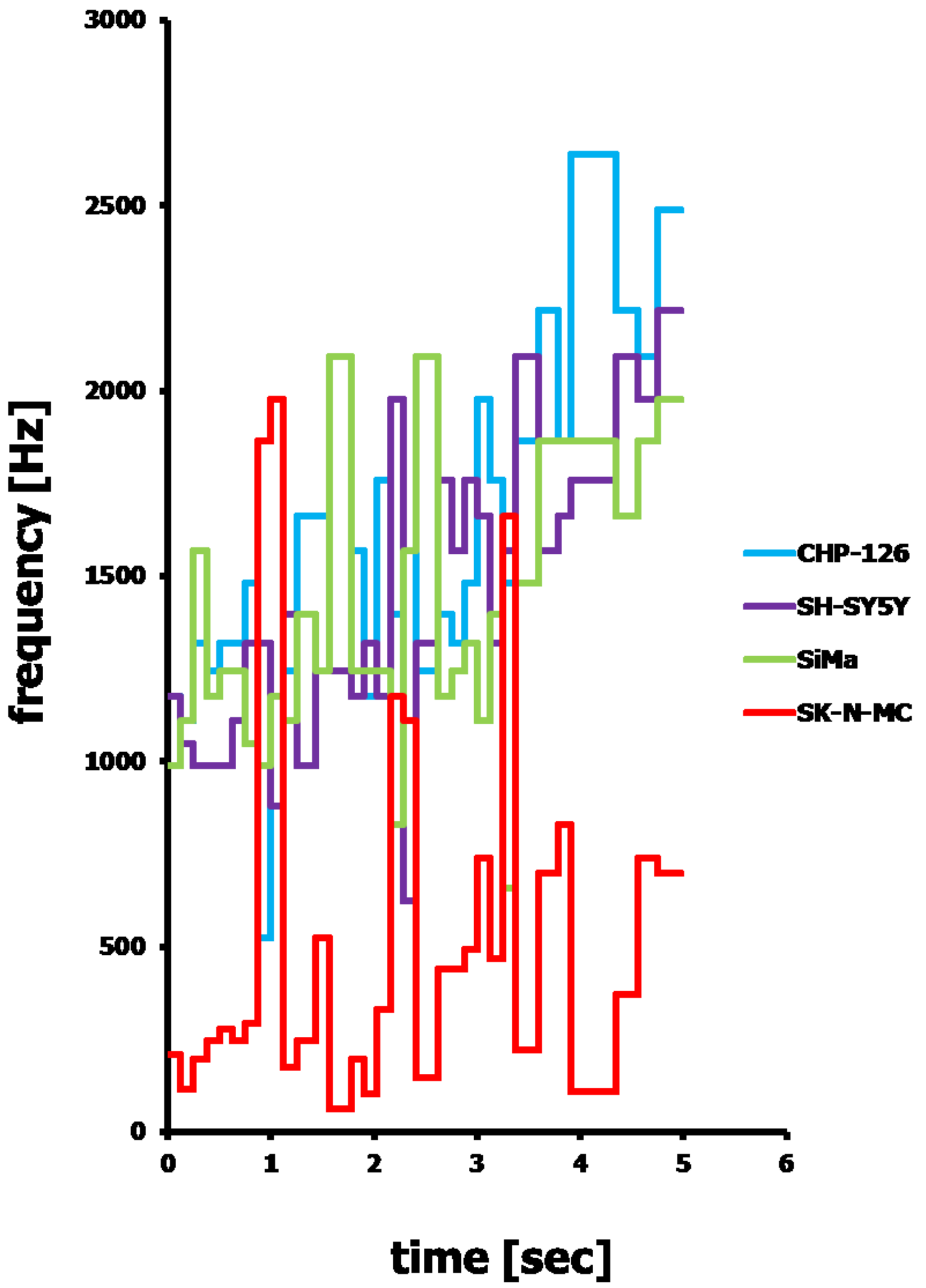

Supplementary Figure 6: Identification of neuroblastoma specific probe sets in "neuroblastoma" cell lines. DNA microarray data from 4 cell lines that were initially established as neuroblastoma cell lines (GSE1824) were transformed into melodies as described in the Methods section by using the following parameters: minimal frequency: 27.5; number of different frequencies: 88; number of tone steps per octave: 12; minimal duration: 1/8; number of tones: 192. Thereafter, probe sets were filtered for frequencies above 987 Hz in exactly 3 samples. All other probe sets were deleted.

microarray data

↓

(optional:) pre-filtering of the data
(*e.g.*, for disease associated probe sets)

↓

setting of the parameters:
- minimal frequency
- number of different frequencies ("keys")
- number of tone steps per octave
- number of notes to generate (*N*)

↓

calculation of the notes:
- calculation of the median signal intensities
- calculation of the frequencies for all samples and the median
- calculation of the variances of the frequencies
- calculation of the durations
- selection of *N* probe sets with highest variances of the frequencies

↓

sorting of the selected probe sets according to the calculated frequencies of the median

↓

(optional:) re-calibration of the frequencies and tone durations using a model melody

↓

transformation of calculated frequencies and durations into sound

Supplementary Figure 7: The Gene Expression Music Algorithm (GEMusicA). DNA microarray data were transformed into sounds in the following steps: 1. Pre-filtering (optional); 2. Parameter setting; 3. Calculation of frequencies and durations; 4. Selection of N probe sets with highest variance; 5.Sorting of selected probe sets according to the median; 5. Re-calibration (only if a model melody is used); 6. Generation of sounds (*e.g.* by using the scripts on pages 126-146 of this Supplement.

Supplementary Information

A. Pre-filtered probe sets used in Figure 3

Probe set ID form Affymetrix HG:U133A microarrays: 200715_x_at, 200853_at, 200951_s_at, 200953_s_at, 201028_s_at, 201029_s_at, 201074_at, 201075_s_at, 201260_s_at, 201291_s_at, 201292_at, 201331_s_at, 201397_at, 201562_s_at, 201563_at, 201565_s_at, 201566_x_at, 201648_at, 201666_at, 201890_at, 201976_s_at, 201981_at, 201982_s_at, 202060_at, 202145_at, 202419_at, 202431_s_at, 202503_s_at, 202554_s_at, 202709_at, 202720_at, 202746_at, 202747_s_at, 202758_s_at, 202790_at, 202870_s_at, 203065_s_at, 203213_at, 203240_at, 203304_at, 203324_s_at, 203418_at, 203554_x_at, 203625_x_at, 203646_at, 203757_s_at, 203764_at, 203790_s_at, 203824_at, 203832_at, 203856_at, 203914_x_at, 204023_at, 204197_s_at, 204198_s_at, 204201_s_at, 204229_at, 204230_s_at, 204244_s_at, 204386_s_at, 204393_s_at, 204424_s_at, 204456_s_at, 204457_s_at, 204603_at, 204686_at, 204712_at, 204779_s_at, 204822_at, 204860_s_at, 204867_at, 204887_s_at, 204917_s_at, 204951_at, 205046_at, 205066_s_at, 205097_at, 205103_at, 205131_x_at, 205145_s_at, 205157_s_at, 205176_s_at, 205199_at, 205227_at, 205306_x_at, 205307_s_at, 205345_at, 205375_at, 205395_s_at, 205440_s_at, 205473_at, 205478_at, 205542_at, 205552_s_at, 205597_at, 205604_at, 205694_at, 205752_s_at, 205827_at, 205828_at, 205872_x_at, 205888_s_at, 205932_s_at, 205962_at, 205990_s_at, 206002_at, 206025_s_at, 206026_s_at, 206110_at, 206114_at, 206149_at, 206159_at, 206164_at, 206231_at, 206309_at, 206316_s_at, 206326_at, 206456_at, 206481_s_at, 206558_at, 206565_x_at, 206581_at, 206626_x_at, 206644_at, 206645_s_at, 206743_s_at, 206745_at, 206812_at, 206866_at, 206906_at, 206915_at, 206935_at, 207016_s_at, 207059_at, 207069_s_at, 207118_s_at, 207165_at, 207172_s_at, 207173_x_at, 207373_at, 207395_at, 207397_s_at, 207398_at, 207400_at, 207493_x_at, 207563_s_at, 207587_at, 207666_x_at, 207670_at, 207675_x_at, 207736_s_at, 207772_s_at, 207869_s_at, 207957_s_at, 208060_at, 208214_at, 208237_x_at, 208242_at, 208262_x_at, 208286_x_at, 208312_s_at, 208334_at, 208345_s_at, 208465_at, 208470_s_at, 208510_s_at, 208565_at, 208771_s_at, 208796_s_at, 208950_s_at, 208951_at, 209129_at, 209270_at, 209294_x_at, 209340_at, 209628_at, 209629_s_at, 209655_s_at, 209656_s_at, 209685_s_at, 209714_s_at, 209734_at, 209752_at, 209773_s_at, 209791_at, 209844_at, 209885_at, 209979_at, 210107_at, 210233_at, 210237_at, 210374_x_at, 210375_at, 210394_x_at, 210475_at, 210497_x_at, 210673_x_at, 210727_at, 210770_s_at, 210783_x_at, 210831_s_at, 210911_at, 210931_at, 210952_at, 210956_at, 210960_at, 210961_s_at, 210963_s_at, 210964_s_at, 211032_at, 211042_x_at, 211128_at, 211138_s_at, 211253_x_at, 211307_s_at, 211419_s_at, 211425_x_at, 211657_at, 211709_s_at, 211802_x_at, 211953_s_at, 211954_s_at, 211955_at, 212045_at, 212097_at, 212236_x_at, 212278_x_at, 212307_s_at, 212402_at, 212415_at, 212501_at, 212612_at,

212621_at, 212713_at, 212739_s_at, 212909_at, 212920_at, 213173_at, 213385_at, 213395_at, 213425_at, 213524_s_at, 213552_at, 213589_s_at, 213714_at, 213793_s_at, 213811_x_at, 213825_at, 213931_at, 214028_x_at, 214290_s_at, 214307_at, 214362_at, 214444_s_at, 214460_at, 214498_at, 214584_x_at, 214604_at, 214611_at, 214636_at, 214730_s_at, 214929_s_at, 214933_at, 215006_at, 215043_s_at, 215071_s_at, 215094_at, 215161_at, 215300_s_at, 215322_at, 215365_at, 215509_s_at, 215510_at, 215559_at, 215652_at, 215695_s_at, 215774_s_at, 215876_at, 215931_s_at, 215962_at, 216000_at, 216052_x_at, 216222_s_at, 216228_s_at, 216471_x_at, 216624_s_at, 216818_s_at, 217013_at, 217026_at, 217303_s_at, 217316_at, 217521_at, 217549_at, 217569_x_at, 217647_at, 217698_at, 218078_s_at, 218182_s_at, 218280_x_at, 218313_s_at, 218663_at, 218665_at, 218782_s_at, 218831_s_at, 218847_at, 218883_s_at, 218959_at, 219090_at, 219102_at, 219104_at, 219306_at, 219343_at, 219360_s_at, 219427_at, 219528_s_at, 219555_s_at, 219572_at, 219597_s_at, 219663_s_at, 219686_at, 219703_at, 219766_at, 219825_at, 219837_s_at, 219887_at, 219908_at, 219918_s_at, 219937_at, 219976_at, 220047_at, 220218_at, 220227_at, 220315_at, 220333_at, 220362_at, 220588_at, 220654_at, 220672_at, 220756_s_at, 220780_at, 220786_s_at, 220820_at, 220828_s_at, 220900_at, 220906_at, 220942_x_at, 220948_s_at, 221011_s_at, 221022_s_at, 221076_at, 221129_at, 221139_s_at, 221185_s_at, 221215_s_at, 221308_at, 221552_at, 221606_s_at, 221679_s_at, 221691_x_at, 221703_at, 221854_at, 221923_s_at, 221928_at, 222025_s_at, 222067_x_at, 222209_s_at, 222254_at, 222312_s_at, 222340_at, 222379_at, 43427_at, 45288_at, 49452_at, 57588_at, 61734_at, AFFX-r2-Hs18SrRNA-M_x_at

B. Gene expression analysis

RNA from cell lines was isolated using TriFast reagent (peqlab, Erlangen, Germany) following the manufacturer's protocol. 2 µg of the RNA were transcribed into cDNA and polymerase chain reaction (RT-PCR) was performed with 5 µL Green Go Taq Buffer (Promega, Mannheim, Germany), 16.8 µL water, 0.5 µL 10mM dNTPs (Fermentas, St. Leon Roth, Germany), 0.25 µL of each primer, 2 µL cDNA and 0.2 µL Go Taq polymerase (Promega). Real-time quantitative RT-PCR (qRT-PCR) was performed using the Maxima™ SYBR Green qPCR Master Mix (Fermentas, St. Leon Roth, Germany). The reaction was performed with 10 µL Maxima™ SYBR Green qPCR Master Mix, 6 µL water, 1 µL of each primer and 2 µL cDNA using the following conditions: 94°C, 30s; 60°C, 30s; 72°C, 45s (40 cycles). The following primer combinations were used[18-20]: achaete-scute complex homolog 1 (ASCL1): 5′-tcg cac aac ctg cat ctt at-3′, 5′-ctt ttg cac aca agc tgc at-3′; Fc fragment of IgG, receptor, transporter, alpha (FCGRT): 5′-cct ggc ttt tcc gtg ctt ac-3′, 5′-ttt gac tgt tag tga cga cga g-3′; integral membrane protein 2 A (ITM2A): 5′- aat gac tgc tta cct gga ctt g-3′,

5′-tcc aca gca act agg tct tct-3′; TATA box binding protein (TBP): 5′-cca ctc aca gac tct cac aac-3′, 5′-ctg cgg tac aat ccc aga act-3′; v-myc avian myelocytomatosis viral oncogene homolog (MYC): 5′-ggc tcc tgg caa aag gtc a-3′, 5′-ctg cgt agt tgt gct gat gt-3′; v-myc avian myelocytomatosis viral oncogene neuroblastoma derived homolog (MYCN): 5′-tga tcc tca aac gat gcc ttc-3′, 5′-gga cgc ctc gct ctt tat ct-3′; X inactive specific transcript (XIST): 5′-ctc cag gcc aat gag aag aa-3′, 5′-tgg cac agt cca cca aat ta-3′. Determination of gene expression was performed using the $2^{-\Delta\Delta Ct}$ method[21].

B. GEMusicAR: R script for GEMusicA

```
# GEMusicAR
# Version 1.6.1
# Friday, September 11, 2015

# This R script transforms DNA microarray data into music files
# as described in reference [1].
# The following files will be generated:
# 1) Audio files (.wav).
# 2) Text files (.txt) that can be transformed into sounds by
#       the PERL script ArrayMusic.pl .
# 3) TeX template files (.tex) for the generation of music scores.

# Usage:
# 1.    Install R on your Windows system
#               (e.g. from http://cran.r-project.org).
# 1.1.  Optionally install RStudio (http://www.rstudio.com/ide)
# 2.    Start R and install the required packages from the
#               Bioconductor homepage:

#                       source("http://bioconductor.org/biocLite.R")
#                       biocLite("affy")
#                       biocLite("limma")
#                       biocLite("genefilter")
#                       biocLite("GEOquery")
#                       biocLite("oligo")
#                       biocLite("hgu133a.db")

#                       biocLite("huex10stprobeset.db")
#                       biocLITE("pd.huex.1.0.st.v2")

#       These packages are required for the analysis of Affymetrix
#       HG_U133A and Human_Exon1.0ST microarrays. For other arrays
#       the corresponding packages have to be installed.

# 3.    Install the audio package

#                       install.packages("audio")

# 4.    Create a new R project and run part A of this GEMusicAR
#               script (lines 1-1100) in your R console. Part A contains
#               the required functions and model data.
#               Part B includes examples for the usage of the functions.
#               The script can be used for automatic download of .cel files
```

```
#		from the Gene Expression Omnibus (GEO) dataset GSE1824 [2].
#		The script uses the MAS5 algorithm for primary analysis of the
#		files and the script generates diverse melodies as described
#		in reference [1] (and additional melodies based on additional
#		models).
#		The script can also be used for automatic download of
#		individual .cel	files from the Gene Expression Omnibus
#		database. The example .cel files from Human_Exon_1.0ST arrays
#		are from references [3] and [4]. In this case, the script
#		uses the RMA algorithm for primary analysis of the files.
#		The script can also be used for the generation of music files
#		on the basis of pre-processed data (e.g. exported files from
#		the Affymetrix Expression Console. These data have to be placed
#		in the "GEMusicAR TXT Input" directory.

#		Running part B of the script will produce a series of directories,
#		.txt files, .tex files, and .wav files in the GEMusicAR directory.
#		Each .txt file will contain the necessary information for
#		generating a single melody as described in reference [1]. Each
#		.tex file contains the information for printing music scores from
#		the data. The generated audio (.wav) files can be directly played
#		or manipulated with appropriate audio software. For generation of
#		stereo version, the usage of Acid Xpress (Sony Creative Software
#		Inc., Mittleton, WI; http://www.acidplanet.com/downloads/xpress/)
#		is suggested.

# 5.	For generation of music scores, install a TeX/LaTeX system
#		(e.g. MiKTeX; http://miktex.org/2.9/setup) and an TeX writing
#		environment (e.g. TeXstudie; http://texstudio.sourceforge.net/).
#		Load the generated .tex files and build the scores.

# 7.	For manual transformation of .txt files into audio files,
#		install a PERL compiler on your system (e.g. ActivePerl;
#		http://www.activestate.com/activeperl/downloads),
#		safe the ArrayMusic.pl PERL script from the Supplementary Material
#		from reference [1] in the PERL directory and
#		execute the PERL script for each individual .txt file.

# References:
# [1]	Staege MS (2015) A short treatise concerning a musical approach
#			for the interpretation of gene expression data (submitted).
# [2]	Staege MS, Hutter C, Neumann I, Foja S, Hattenhorst UE, Hansen G,
#			Afar D, Burdach S (2015) DNA microarrays reveal relationship of
#			Ewing family tumors to both endothelial and fetal neural creast-
#			derived cells and define novel targets. Cancer Res. 64:8213-21.
# [3]	Kewitz S, Staege MS (2013) Expression and Regulation of the
#			Endogenous Retrovirus 3 in Hodgkin's Lymphoma Cells.
#			Front Oncol. 3:179.
# [4]	Nikitin PA, Yan CM, Forte E, Bocedi A, Tourigny JP, White RE,
#			Allday MJ, Patel A, Dave SS, Kim W, Hu K, Guo J, Tainter D,
#			Rusyn E, Luftig MA (2010) An ATM/Chk2-mediated DNA damage-
#			responsive signaling pathway suppresses Epstein-Barr virus
#			transformation of primary human B cells.
#			Cell Host Microbe. 8:510-22.
```

```
#-----------------------------------------------------------------------
# Part A (functions and model data)
#-----------------------------------------------------------------------
# load libraries
library(affy)
library(limma)
library(genefilter)
library(hgu133a.db)
library(GEOquery)
library(audio)
library(oligo)
library(huex10stprobeset.db)
library(pd.huex.1.0.st.v2)

# GEMusicA function
GEMusicA<-function(notes,signals){
# calculate frequencies
	print(paste("calculate frequencies for",nrow(signals),"probe sets"))
	cnames<-colnames(signals)
	signals<-data.frame(cbind(signals,NA,NA,NA))
	colnames(signals)<-c(cnames,"Med","Variability","counter")
	cnames<-colnames(signals)
	signals$Med<-apply(signals,1,median,na.rm=TRUE)
	co<-ncol(signals)
	sigmax<-apply(signals,2,max)
	for(k in 1:co){
		signals[,k]<-ceiling((signals[,k]/sigmax[k]*numkeys))
	}
	signals<-data.frame((((2^((signals-1)/tonesteps)*minfreq)*(signals>0))
	+(minfreq*(signals<1))))
	colnames(signals)<-cnames

# select probe sets with highest variability
	print(paste("select",notes,"probe sets with highest variability"))
	ro<-nrow(signals)
	co<-ncol(signals)-3
	s1<-data.frame(signals)
	s1$Med<-NULL
	signals$Variability<-apply(s1,1,var,na.rm=TRUE)
	signals[,"counter"]<-1:nrow(signals)
	signals<-signals[order(signals[,"Variability"]),]
	selection<-tail(signals,(notes+1))
	maxvar<-selection[nrow(selection),"Variability"]
	minvar<-selection[1,"Variability"]
	selection<-tail(signals,notes)
	selection<-selection[order(selection[,"Med"],selection[,
		"counter"]),]

# calculate durations
	print(paste("calculate duration of",notes,"tones"))
	sy<-log2(mindur)+1
	st<-(maxvar-minvar)/sy
	ndots<-maxNdots+1
	mdots<-maxNdots-1
	st3<-st/ndots
	selection<-data.frame(cbind(selection,0,0,0,0,0))
	colnames(selection)<-c(cnames,"val","dot","dot2","dot3","Duration")
	selection$val<-2^((ceiling(((selection$Variability)-minvar)/st))-1)
```

```
        selection$dot<-(((((ceiling((selection$Variability-minvar)/st3))))
                +mdots)/ndots)==(ceiling((((((ceiling((selection$Variability-
                minvar)/st3)))+mdots)/ndots))))
        selection$dot2<-(((((ceiling((selection$Variability-minvar)/
                st3))))+(mdots-1))/ndots)==(ceiling((((((ceiling(
                (selection$Variability-minvar)/st3)))+(mdots-1))/ndots))))
        selection$dot3<-(((((ceiling((selection$Variability-minvar)/st3))))
                +(mdots-2))/ndots)==(ceiling((((((ceiling((selection$Variability-
                minvar)/st3)))+(mdots-2))/ndots))))
        if(maxNdots==0){
                selection$dot<-0
                selection$dot2<-0
                selection$dot3<-0
        }
        if(maxNdots==1){
                        selection$dot2<-0
                        selection$dot3<-0
        }
        if(maxNdots==2){
                selection$dot3<-0
        }
        selection$Duration<-(selection$val+(0.5*selection$val*selection$dot)+
                (0.75*selection$val*selection$dot2)+0.875*selection$val*
                selection$dot3)/mindur
        selection
}

# Generation of .txt files, .tex files and .wav files
#       (N probe sets)
GenerateNtones<-function(Dataset,N,PSfilter=""){
        vals<-GEMusicA(notes=N,signals=signalslog2)
        colnames(vals)[ncol(signalslog2)+1]<-samp[length(samp)]
        setwd(HostDir)
        setwd(paste("GEMusicAR/",Dataset,"Results",sep=""))
        write.csv(vals,file=paste(Dataset,N,PSfilter,".txt",sep=""),quote=F)
        template<-data.frame(nrow=nrow(vals),ncol=2)
        nrowtex=nrow(vals)+11
        textemplate<-data.frame(nrow=nrowtex,ncol=2)
        textemplate2voices<-data.frame(nrow=nrowtex,ncol=2)
        ro<-nrow(vals)
        co<-ncol(signalslog2)+1
        for(k in 1:co){
                mel<-NULL
                print(paste("generating 4 files for",colnames(vals)[k]))
                for (i in 1:ro) {
                        template[i,1]<-vals[i,k]
                        template[i,2]<-vals[i,"Duration"]
                        if(i/5==round(i/5)){
                                bar<-paste("\\","bar",sep="")
                        } else {
                                bar<-""
                        }
                        if(symbulFT[paste("",vals[i,"Duration"]/TeXscalefactor,
                                sep="")]=="T"){
                                sul<-symbul[paste("",round(vals[i,k],1),sep="")]
                                sulmed<-symbul[paste("",round(vals[i,ncol(signalslog2)+1],
                                1),sep="")]
                        } else {
```

```
            sul<-""
            sulmed<-""
        }
        if(symbd[paste("",vals[i,"Duration"]/TeXscalefactor,sep="")]!=""){
            sbd<-paste(symbd[paste("",vals[i,"Duration"]/
                TeXscalefactor,sep="")],"{",symbh[paste("",
                round(vals[i,k],1),sep="")],"}\\",sep="")
            sbdmed<-paste(symbd[paste("",vals[i,"Duration"]/
                TeXscalefactor,sep="")],"{",symbh[paste("",round(vals[i,
                ncol(signalslog2)+1],1),sep="")],"}\\",sep="")
        } else {
            sbd<-""
            sbdmed<-""
        }
        textemplate[i+8,1]<-paste("\\",symbfont[paste("",vals[i,"Duration"]/
            TeXscalefactor,sep="")],"\\transpose=",transp[paste("",
            round(vals[i,k],1),sep="")],"\\",sbd,symbl[paste("",
            vals[i,"Duration"]/TeXscalefactor,sep="")],sul,"{",symbh[paste("",
            round(vals[i,k],1),sep="")],"}\\en",bar,sep="")
        textemplate2voices[i+8,1]<-paste("\\",symbfont[paste("",vals[i,
            "Duration"]/TeXscalefactor,sep="")],"\\transpose=",transp[paste("",
            round(vals[i,ncol(signalslog2)+1],1),sep="")],"\\",sbdmed,
            symbl[paste("",vals[i,"Duration"]/TeXscalefactor,sep="")],sulmed,
            "{",symbh[paste("",round(vals[i,ncol(signalslog2)+1],1),sep="")],
            "}&\\transpose=",transp[paste("",round(vals[i,k],1),sep="")],"\\",
            sbd,symbl[paste("",vals[i,"Duration"]/TeXscalefactor,sep="")],
            sul,"{",symbh[paste("",round(vals[i,k],1),sep="")],"}\\en",
            bar,sep="")
        tone=sin(pi*template[i,1]*seq(0,4*template[i,2],
            length.out=(44100*template[i,2])))
        mel<-c(mel,tone)
    }
    mel<-mel*vol
    mel[mel < -1] <- -1
    mel[mel > 1] <- 1
    textemplate[1,1]<-"\\documentclass{scrbook}"
    textemplate[2,1]<-"\\usepackage{musixtex}"
    textemplate[3,1]<-"\\begin{document}"
    textemplate[4,1]<-"Accidentals only apply to the immediate note\\\\"
    textemplate[5,1]<-paste(colnames(vals)[k],"\\\\\\\\",sep="")
    textemplate[6,1]<-"\\begin{music}"
    textemplate[7,1]<-"\\instrumentnumber1"
    textemplate[8,1]<-"\\startpiece"
    textemplate[nrowtex-2,1]<-"\\endpiece"
    textemplate[nrowtex-1,1]<-"\\end{music}"
    textemplate[nrowtex,1]<-"\\end{document}"
    textemplate[,2]<-NULL
    textemplate2voices[1,1]<-"\\documentclass{scrbook}"
    textemplate2voices[2,1]<-"\\usepackage{musixtex}"
    textemplate2voices[3,1]<-"\\begin{document}"
    textemplate2voices[4,1]<-paste("Accidentals only apply to the immediate note\\\\",
        colnames(vals)[k],"\\\\\\\\",sep="")
    textemplate2voices[5,1]<-"\\begin{music}"
    textemplate2voices[6,1]<-"\\instrumentnumber2\\interstaff{30}"
    textemplate2voices[7,1]<-"\\setname1{Median}"
    textemplate2voices[8,1]<-"\\startpiece"
    textemplate2voices[nrowtex-2,1]<-"\\endpiece"
    textemplate2voices[nrowtex-1,1]<-"\\end{music}"
```

```
		textemplate2voices[nrowtex,1]<-"\\end{document}"
		textemplate2voices[,2]<-NULL
		write.table(template,file=paste(colnames(vals)[k],
			PSfilter,N,".txt",sep=""),row.names=FALSE,
			col.names=FALSE,sep="\t")
		write.table(textemplate,file=paste(colnames(vals)[k],
			PSfilter,N,"TeX.tex",sep=""),row.names=FALSE,
			col.names=FALSE,quote=FALSE)
		write.table(textemplate2voices,file=paste(colnames(vals)[k],
			PSfilter,N,"stTeX.tex",sep=""),row.names=FALSE,
			col.names=FALSE,quote=FALSE)
		aud<-audioSample(mel,rate=44100,bits=16)
		save.wave(aud,paste(colnames(vals)[k],PSfilter,N,".wav",sep=""))
	}
	setwd(HostDir)
}

# Generation of .txt files, .tex files, and .wav files
#	(based on a list of pre-filtered probe sets)
GeneratePrefiltered<-function(Dataset,filteredPS){
	setwd(HostDir)
	setwd(paste("GEMusicAR/GEMusicAR Filter",sep=""))
	PSlistprim<-read.table(file=filteredPS)
	PSlistpost<-matrix(unlist(PSlistprim),ncol=(ncol(PSlistprim)))
	ID<-rownames(signalslog2)
	psnames<-cbind(signalslog2,0)
	colnames(psnames)<-c(colnames(signalslog2),"ps")
	ro<-nrow(psnames)
	for (i in 1:ro){
		psnames[i,"ps"]<-ID[i]
	}
	counter<-0
	rnames<-NULL
	prefiltered<-matrix(nrow=length(PSlistpost),ncol=ncol(signalslog2))
	colnames(prefiltered)<-colnames(signalslog2)
	ro<-nrow(psnames)
	co<-ncol(signalslog2)
	for (i in 1:ro){
		if (psnames[i,"ps"] %in% PSlistpost){
			counter<-counter+1
			for(k in 1:co){
				prefiltered[counter,k]<-signalslog2[i,k]
			}
			rnames<-c(rnames, psnames[i,"ps"])
		}
	}
	rownames(prefiltered)<-rnames
	assign("signalslog2",prefiltered,envir=globalenv())
	setwd(HostDir)
}

# Generation of .txt files for ArrayMusic.pl and .wav files
#	(based on a model melody)
ExtractModel<-function(Dataset,Model){
	Modeldur<-Model$dur
	Modelkeys<-Model$keys
	Modelname<-Model[1,3]
	Metronome<-Model[1,4]
```

```
notes<-length(Modeldur)
vals<-GEMusicA(notes=notes,signals=signalslog2)
valsnew<-matrix(ncol=ncol(vals),nrow=nrow(vals))
beats<-50/Metronome
colnames(valsnew)<-colnames(vals)
rownames(valsnew)<-rownames(vals)
ro<-nrow(valsnew)
co<-ncol(signalslog2)
for(i in 1:ro){
	valsnew[i,ncol(signalslog2)+1]<-(((2^((Modelkeys[i]-1)/tonesteps)*
		minfreq)*(Modelkeys[i]>0))+(minfreq*(Modelkeys[i]<1)))
	valsnew[i,"Duration"]<-Modeldur[i]
	for(k in 1:co){
		valsnew[i,k]<-vals[i,k]*valsnew[i,ncol(signalslog2)+1]/
		vals[i,ncol(signalslog2)+1]
	}
}
setwd(HostDir)
setwd(paste("GEMusicAR/",Dataset,"Results",sep=""))
colnames(valsnew)[ncol(signalslog2)+1]<-samp[length(samp)]
write.csv(valsnew,file=paste(Dataset,Modelname,".txt",sep=""),quote=F)
template<-data.frame(nrow=nrow(valsnew),ncol=2)
nrowtex=nrow(valsnew)+11
textemplate<-data.frame(nrow=nrowtex,ncol=2)
textemplate2voices<-data.frame(nrow=nrowtex,ncol=2)
ro<-nrow(valsnew)
co<-ncol(signalslog2)+1
for(k in 1:co){
	mel<-NULL
	print(paste("generating 4 files for",colnames(vals)[k]))
	for(i in 1:ro){
		template[i,1]<-valsnew[i,k]
		template[i,2]<-valsnew[i,"Duration"]
		if(i/5==round(i/5)){
			bar<-paste("\\","bar",sep="")
		} else {
			bar<-""
		}
		if(paste("",valsnew[i,"Duration"]/TeXscalefactor,sep="")
			%in% c("2.5","1.25","0.625")){
			first<-splits[paste("",valsnew[i,"Duration"]/TeXscalefactor,
				"1",sep="")]
			second<-splits[paste("",valsnew[i,"Duration"]/TeXscalefactor,
				"2",sep="")]
			if(symbulFT[paste("",first,sep="")]=="T"){
				sul<-symbul[paste("",round(valsnew[i,k],1),sep="")]
				sulmed<-symbul[paste("",round(valsnew[i,
					ncol(signalslog2)+1],1),sep="")]
			} else {
				sul<-""
				sulmed<-""
			}
			textemplate[i+8,1]<-paste("\\",symbfont[paste("",first,
				sep="")],"\\","transpose=",
				transp[paste("",round(valsnew[i,k],1),sep="")],"\\itied0{",
				symbh[paste("",round(valsnew[i,k],1),sep="")],"}\\",
				symbl[paste("",first,sep="")],
				sul,"{",symbh[paste("",round(valsnew[i,k],1),sep="")],
```

```
                "}\\en\\",symbfont[paste("",second,sep="")],"\\",
                "transpose=",transp[paste("",round(valsnew[i,k],1),
                sep="")],"\\ttie0\\",symbl[paste("",second,sep="")],
                sul,"{",symbh[paste("",round(valsnew[i,k],1),sep="")],
                "}\\en",bar,sep="")
        textemplate2voices[i+8,1]<-paste("\\",symbfont[paste("",first,
                sep="")],"\\transpose=",transp[paste("",round(valsnew[i,
                ncol(signalslog2)+1],1),sep="")],"\\itied1{",
                symbh[paste("",round(valsnew[i,ncol(signalslog2)+1],1),
                sep="")],"}\\",symbl[paste("",first,sep="")],sulmed,"{",
                symbh[paste("",round(valsnew[i,ncol(signalslog2)+1],1),
                sep="")],"}&\\transpose=",transp[paste("",round(valsnew[i,
                k],1),sep="")],"\\itied2{",symbh[paste("",round(valsnew[i,
                k],1),sep="")],"}\\",symbl[paste("",first,sep="")],sul,"{",
                symbh[paste("",round(valsnew[i,k],1),sep="")],"}\\en\\",
                symbfont[paste("",second,sep="")],"\\transpose=",
                transp[paste("",round(valsnew[i,ncol(signalslog2)+1],1),
                sep="")],"\\ttie1\\",symbl[paste("",second,sep="")],
                sulmed,"{",symbh[paste("",round(valsnew[i,
                ncol(signalslog2)+1],1),sep="")],"}&\\transpose=",
                transp[paste("",round(valsnew[i,k],1),sep="")],
                "\\ttie2\\",symbl[paste("",second,sep="")],sul,"{",
                symbh[paste("",round(valsnew[i,k],1),sep="")],"}\\en",
                bar,sep="")
} else {
        if(symbulFT[paste("",valsnew[i,"Duration"]/TeXscalefactor,
                sep="")]=="T"){
                sul<-symbul[paste("",round(valsnew[i,k],1),sep="")]
                sulmed<-symbul[paste("",round(valsnew[i,
                        ncol(signalslog2)+1],1),sep="")]
        } else {
                sul<-""
                sulmed<-""
        }
        if(symbd[paste("",valsnew[i,"Duration"]/TeXscalefactor,
                sep="")]!=""){
                sbd<-paste(symbd[paste("",valsnew[i,"Duration"]/
                TeXscalefactor,sep="")],"{",symbh[paste("",
                round(valsnew[i,k],1),sep="")],"}\\",sep="")
                sbdmed<-paste(symbd[paste("",valsnew[i,"Duration"]/
                        TeXscalefactor,sep="")],"{",symbh[paste("",
                        round(valsnew[i,ncol(signalslog2)+1],1),sep="")],
                        "}\\",sep="")
        } else {
                sbd<-""
                sbdmed<-""
        }
        textemplate[i+8,1]<-paste("\\",symbfont[paste("",valsnew[i,
                "Duration"]/TeXscalefactor,sep="")],"\\","transpose=",
                transp[paste("",round(valsnew[i,k],1),sep="")],"\\",sbd,
                symbl[paste("",valsnew[i,"Duration"]/TeXscalefactor,sep="")],
                sul,"{",symbh[paste("",round(valsnew[i,k],1),sep="")],"}\\en",
                bar,sep="")
        textemplate2voices[i+8,1]<-paste("\\",symbfont[paste("",valsnew[i,
                "Duration"]/TeXscalefactor,sep="")],"\\transpose=",
                transp[paste("",round(valsnew[i,ncol(signalslog2)+1],1),
                sep="")],"\\",sbdmed,symbl[paste("",valsnew[i,"Duration"]/
                TeXscalefactor,sep="")],sulmed,"{",symbh[paste("",
```

```
                                        round(valsnew[i,ncol(signalslog2)+1],1),sep="")],
                                        "}&\\transpose=",transp[paste("",round(valsnew[i,k],1),
                                        sep="")],"\\",sbd,symbl[paste("",valsnew[i,"Duration"]/
                                        TeXscalefactor,sep="")],sul,"{",symbh[paste("",
                                        round(valsnew[i,k],1),sep="")],"}\\en",bar,sep="")
                        }
                        tone=sin(pi*template[i,1]*seq(0,4*beats*template[i,2],
                                length.out=(44100*beats*template[i,2])))
                        mel<-c(mel,tone)
                }
                mel<-mel*vol
                mel[mel < -1] <- -1
                mel[mel > 1] <- 1
                textemplate[1,1]<-"\\documentclass{scrbook}"
                textemplate[2,1]<-"\\usepackage{musixtex}"
                textemplate[3,1]<-"\\begin{document}"
                textemplate[4,1]<-"Accidentals only apply to the immediate note\\\\"
                textemplate[5,1]<-paste(colnames(vals)[k],"\\\\\\\\",sep="")
                textemplate[6,1]<-"\\begin{music}"
                textemplate[7,1]<-"\\instrumentnumber1"
                textemplate[8,1]<-"\\startpiece"
                textemplate[nrowtex-2,1]<-"\\endpiece"
                textemplate[nrowtex-1,1]<-"\\end{music}"
                textemplate[nrowtex,1]<-"\\end{document}"
                textemplate[,2]<-NULL
                textemplate2voices[1,1]<-"\\documentclass{scrbook}"
                textemplate2voices[2,1]<-"\\usepackage{musixtex}"
                textemplate2voices[3,1]<-"\\begin{document}"
                textemplate2voices[4,1]<-paste("Accidentals only apply to the immediate note\\\\",
                        colnames(vals)[k],"\\\\\\\\",sep="")
                textemplate2voices[5,1]<-"\\begin{music}"
                textemplate2voices[6,1]<-"\\instrumentnumber2\\interstaff{30}"
                textemplate2voices[7,1]<-"\\setname1{Median}"
                textemplate2voices[8,1]<-"\\startpiece"
                textemplate2voices[nrowtex-2,1]<-"\\endpiece"
                textemplate2voices[nrowtex-1,1]<-"\\end{music}"
                textemplate2voices[nrowtex,1]<-"\\end{document}"
                textemplate2voices[,2]<-NULL
                write.table(template, file=paste(colnames(valsnew)[k],
                        Modelname,".txt",sep=""),row.names=FALSE,col.names=FALSE,sep="\t")
                write.table(textemplate,file=paste(colnames(valsnew)[k],
                        Modelname,"TeX.tex",sep=""),row.names=FALSE,
                        col.names=FALSE,quote=FALSE)
                write.table(textemplate2voices,file=paste(colnames(vals)[k],Modelname,
                        "stTeX.tex",sep=""),row.names=FALSE,col.names=FALSE,quote=FALSE)
                aud<-audioSample(mel,rate=44100,bits=16)
                save.wave(aud,paste(colnames(valsnew)[k],Modelname,".wav",sep=""))
        }
        setwd(HostDir)
}

# Preparation of data based on a .txt file with pre-processed data
preproc<-function(file){
        setwd(HostDir)
        setwd("GEMusicAR")
        setwd("GEMusicAR TXT Input")
        signalsprim<-read.csv(file=file, row.names=1)
        signalspost<-matrix(unlist(signalsprim), ncol=(ncol(signalsprim)))
```

```
	colnames(signalspost)<-c(colnames(signalsprim))
	rownames(signalspost)<-c(rownames(signalsprim))
	assign("signalslog2",signalspost,envir=globalenv())
	setwd(HostDir)
}

processed_data<-function(Dataset,N,newnames=FALSE){
	setwd(HostDir)
	setwd("GEMusicAR")
	subdout<-paste(Dataset,"Results",sep="")
	dir.create(subdout,showWarnings = FALSE)
	preproc(paste(Dataset,".txt",sep=""))
	if(newnames==TRUE){
		signalslog2<-replacenames(signalslog2,samp)
	}
	colnames(signalslog2)<-gsub("[[:punct:]]"," ",colnames(signalslog2))
	GenerateNtones(Dataset=Dataset,N=N)
	setwd(HostDir)
}

# Transformation table for frequencies
symbh<-c("1.7"="A","1.8"="^A","1.9"="B","2.0"="C","2.2"="^C","2.3"="D",
	"2.4"="^D-35","2.6"="E","2.7"="F","2.9"="^F","3.1"="G","3.2"="^G",
	"3.4"="A","3.6"="^A","3.9"="B","4.1"="C","4.3"="^C",
	"4.6"="D","4.9"="^D","5.2"="E","5.5"="F","5.8"="^F","6.1"="G",
	"6.5"="^G","6.9"="A","7.3"="^A","7.7"="B","8.2"="C","8.7"="^C",
	"9.2"="D","9.7"="^D","10.3"="E","10.9"="F","11.6"="^F","12.2"="G",
	"13"="^G","13.7"="A","13.8"="A","14.6"="^A","15.4"="B","16.4"="C",
	"17.3"="^C","18.4"="D","19.4"="^D","20.6"="E","21.8"="F",
	"23.1"="^F","24.5"="G","26"="^G","27.5"="A","29.1"="^A",
	"30.9"="B","32.7"="C","34.6"="^C","36.7"="D","38.9"="^D",
	"41.2"="E","43.7"="F","46.2"="^F","49"="G","51.9"="^G","55"="A",
	"58.3"="^A","61.7"="B","65.4"="C","69.3"="^C","73.4"="D",
	"77.8"="^D","82.4"="E","87.3"="F","92.5"="^F","98"="G",
	"103.8"="^G","110"="A","116.5"="^A","123.5"="B","130.8"="C",
	"138.6"="^C","146.8"="D","155.6"="^D","164.8"="E","174.6"="F",
	"185"="^F","196"="G","207.7"="^G","220"="a","233.1"="^a",
	"246.9"="b","261.6"="c","277.2"="^c","293.7"="d","311.1"="^d",
	"329.6"="e","349.2"="f","370"="^f","392"="g","415.3"="^g",
	"440"="a","466.2"="^a","493.9"="b","523.3"="c","554.4"="^c",
	"587.3"="d","622.3"="^d","659.3"="e","698.5"="f","740"="^f",
	"784"="g","830.6"="^g","880"="a","932.3"="^a","987.8"="b",
	"1046.5"="c","1108.7"="^c","1174.7"="d","1244.5"="^d",
	"1318.5"="e","1396.9"="f","1480"="^f","1568"="g","1661.2"="^g",
	"1760"="a","1864.7"="^a","1975.5"="b","2093"="c","2217.5"="^c",
	"2349.3"="d","2489"="^d","2637"="e","2793.8"="f","2960"="^f",
	"3136"="g","3322.4"="^g","3520"="a","3729.3"="^a","3951.1"="b",
	"4186"="c","4434.9"="^c","4698.6"="d","4978"="^d","5274"="e",
	"5587.7"="f","5919.9"="^f","6271.9"="g","6644.9"="^g","7040"="a",
	"7458.6"="^a","7902.1"="b","8372"="c","8869.8"="^c",
	"9397.3"="d","9956.1"="^d","10548.1"="e","11175.3"="f",
	"11839.8"="^f","12543.9"="g","13289.8"="^g","14080"="a")

transp<-c("1.7"="-35","1.8"="-35","1.9"="-35","2.0"="-35","2.2"="-35","2.3"="-35",
	"2.4"="-35","2.6"="-35","2.7"="-35","2.9"="-35","3.1"="-35","3.2"="-35",
	"3.4"="-28","3.6"="-28","3.9"="-28","4.1"="-28","4.3"="-28",
	"4.6"="-28","4.9"="-28","5.2"="-28","5.5"="-28","5.8"="-28",
	"6.1"="-28","6.5"="-28","6.9"="-21","7.3"="-21","7.7"="-21",
```

```
"8.2"="-21","8.7"="-21","9.2"="-21","9.7"="-21","10.3"="-21",
"10.9"="-21","11.6"="-21","12.2"="-21","13"="-21","13.7"="-14","13.8"="-14",
"14.6"="-14","15.4"="-14","16.4"="-14","17.3"="-14","18.4"="-14",
"19.4"="-14","20.6"="-14","21.8"="-14","23.1"="-14","24.5"="-14",
"26"="-14","27.5"="-7","29.1"="-7","30.9"="-7","32.7"="-7",
"34.6"="-7","36.7"="-7","38.9"="-7","41.2"="-7","43.7"="-7",
"46.2"="-7","49"="-7","51.9"="-7","55"="0","58.3"="0","61.7"="0",
"65.4"="0","69.3"="0","73.4"="0","77.8"="0","82.4"="0","87.3"="0",
"92.5"="0","98"="0","103.8"="0","110"="7","116.5"="7","123.5"="7",
"130.8"="7","138.6"="7","146.8"="7","155.6"="7","164.8"="7",
"174.6"="7","185"="7","196"="7","207.7"="7","220"="0","233.1"="0",
"246.9"="0","261.6"="0","277.2"="0","293.7"="0","311.1"="0",
"329.6"="0","349.2"="0","370"="0","392"="0","415.3"="0","440"="7",
"466.2"="7","493.9"="7","523.3"="7","554.4"="7","587.3"="7",
"622.3"="7","659.3"="7","698.5"="7","740"="7","784"="7","830.6"="7",
"880"="14","932.3"="14","987.8"="14","1046.5"="14","1108.7"="14",
"1174.7"="14","1244.5"="14","1318.5"="14","1396.9"="14","1480"="14",
"1568"="14","1661.2"="14","1760"="21","1864.7"="21","1975.5"="21",
"2093"="21","2217.5"="21","2349.3"="21","2489"="21","2637"="21",
"2793.8"="21","2960"="21","3136"="21","3322.4"="21","3520"="28",
"3729.3"="28","3951.1"="28","4186"="28","4434.9"="28","4698.6"="28",
"4978"="28","5274"="28","5587.7"="28","5919.9"="28","6271.9"="28",
"6644.9"="28","7040"="35","7458.6"="35","7902.1"="35","8372"="35",
"8869.8"="35","9397.3"="35","9956.1"="35","10548.1"="35","11175.3"="35",
"11839.8"="35","12543.9"="35","13289.8"="35","14080"="42")

symbul<-c("1.7"="u","1.8"="u","1.9"="u","2.0"="u","2.2"="u","2.3"="u",
"2.4"="u","2.6"="u","2.7"="u","2.9"="u","3.1"="u","3.2"="u","3.4"="u",
"3.6"="u","3.9"="u","4.1"="u","4.3"="u","4.6"="u","4.9"="u","5.2"="u",
"5.5"="u","5.8"="u","6.1"="u","6.5"="u","6.9"="u","7.3"="u","7.7"="u",
"8.2"="u","8.7"="u","9.2"="u","9.7"="u","10.3"="u",
"10.9"="u","11.6"="u","12.2"="u","13"="u","13.7"="u","13.8"="u",
"14.6"="u","15.4"="u","16.4"="u","17.3"="u","18.4"="u",
"19.4"="u","20.6"="u","21.8"="u","23.1"="u","24.5"="u",
"26"="u","27.5"="u","29.1"="u","30.9"="u","32.7"="u",
"34.6"="u","36.7"="u","38.9"="u","41.2"="u","43.7"="u",
"46.2"="u","49"="u","51.9"="u","55"="u","58.3"="u","61.7"="u",
"65.4"="u","69.3"="u","73.4"="u","77.8"="u","82.4"="u","87.3"="u",
"92.5"="u","98"="u","103.8"="u","110"="u","116.5"="u","123.5"="u",
"130.8"="u","138.6"="u","146.8"="u","155.6"="u","164.8"="u",
"174.6"="u","185"="u","196"="u","207.7"="u","220"="u","233.1"="u",
"246.9"="u","261.6"="u","277.2"="u","293.7"="u","311.1"="u",
"329.6"="u","349.2"="u","370"="u","392"="u","415.3"="u","440"="l",
"466.2"="l","493.9"="l","523.3"="l","554.4"="l","587.3"="l",
"622.3"="l","659.3"="l","698.5"="l","740"="l","784"="l","830.6"="l",
"880"="l","932.3"="l","987.8"="l","1046.5"="l","1108.7"="l",
"1174.7"="l","1244.5"="l","1318.5"="l","1396.9"="l","1480"="l",
"1568"="l","1661.2"="l","1760"="l","1864.7"="l","1975.5"="l",
"2093"="l","2217.5"="l","2349.3"="l","2489"="l","2637"="l",
"2793.8"="l","2960"="l","3136"="l","3322.4"="l","3520"="l",
"3729.3"="l","3951.1"="l","4186"="l","4434.9"="l","4698.6"="l",
"4978"="l","5274"="l","5587.7"="l","5919.9"="l","6271.9"="l",
"6644.9"="l","7040"="l","7458.6"="l","7902.1"="l","8372"="l",
"8869.8"="l","9397.3"="l","9956.1"="l","10548.1"="l","11175.3"="l",
"11839.8"="l","12543.9"="l","13289.8"="l","14080"="l")

# Transformation table for durations
symbl<-c("2"="breve","4"="longa","3"="breve","6"="longa",
```

```
	"3.5"="breve","7"="longa","3.75"="breve","7.5"="longa",
	"1"="wh","0.5"="h","0.25"="q","0.125"="c","0.0625"="cc",
	"0.03125"="ccc","0.015625"="cccc","0.0078125"="ccccc","1.5"="wh",
	"0.75"="h","0.375"="q","0.1875"="c","0.09375"="cc",
	"0.046875"="ccc","0.0234375"="cccc","0.01171875"="ccccc",
	"1.75"="wh","0.875"="h","0.4375"="q","0.21875"="c","0.109375"="cc",
	"0.0546875"="ccc","0.02734375"="cccc","0.013671875"="ccccc",
	"1.875"="wh","0.9375"="h","0.46875"="q","0.234375"="c","0.1171875"="cc",
	"0.05859375"="ccc","0.029296875"="cccc","0.014648438"="ccccc")

symbulFT<-c("2"="F","4"="F","3"="F","6"="F","3.5"="F","7"="F",
	"3.75"="F","7.5"="F","1"="F","0.5"="T","0.25"="T","0.125"="T",
	"0.0625"="T","0.03125"="T","0.015625"="T","0.0078125"="T",
	"1.5"="F","0.75"="T","0.375"="T","0.1875"="T","0.09375"="T",
	"0.046875"="T","0.0234375"="T","0.01171875"="T",
	"1.75"="F","0.875"="T","0.4375"="T","0.21875"="T","0.109375"="T",
	"0.0546875"="T","0.02734375"="T","0.013671875"="T","1.875"="F",
	"0.9375"="T","0.46875"="T","0.234375"="T","0.1171875"="T",
	"0.05859375"="T","0.029296875"="T","0.014648438"="T")

symbd<-c("2"="","4"="","3"="pt","6"="pt","3.5"="ppt","7"="ppt",
	"3.75"="pppt","7.5"="pppt","1"="","0.5"="","0.25"="","0.125"="","0.0625"="",
	"0.03125"="","0.015625"="","0.0078125"="","1.5"="pt","0.75"="pt",
	"0.375"="pt","0.1875"="pt","0.09375"="pt","0.046875"="pt","0.0234375"="pt",
	"0.01171875"="pt","1.75"="ppt","0.875"="ppt","0.4375"="ppt","0.21875"="ppt",
	"0.109375"="ppt","0.0546875"="ppt","0.02734375"="ppt","0.013671875"="ppt",
	"1.875"="pppt","0.9375"="pppt","0.46875"="pppt","0.234375"="pppt",
	"0.1171875"="pppt","0.05859375"="pppt","0.029296875"="pppt","0.014648438"="pppt")

symbfont<-c("2"="NOTEs","4"="NOTEs","3"="NOTEs","6"="NOTEs","3.5"="NOTEs",
	"7"="NOTEs","3.75"="NOTEs","7.5"="NOTEs","1"="NOTEs","0.5"="NOTes",
	"0.25"="NOtes","0.125"="Notes","0.0625"="notes","0.03125"="notes",
	"0.015625"="notes","0.0078125"="notes","1.5"="NOTEs","0.75"="NOTesp",
	"0.375"="NOtesp","0.1875"="Notesp","0.09375"="notesp","0.046875"="notes",
	"0.0234375"="notes","0.01171875"="notes","1.75"="NOTEs","0.875"="NOTEs",
	"0.4375"="NOTes","0.21875"="NOtes","0.109375"="notesp","0.0546875"="notes",
	"0.02734375"="notes","0.013671875"="notes","1.875"="NOTEs","0.9375"="NOTEs",
	"0.46875"="NOTes","0.234375"="NOtes","0.1171875"="notesp","0.05859375"="notes",
	"0.029296875"="notes","0.014648438"="notes")

# Splitting of non-standard length
splits<-c("2.5"=c("2","0.5"),"1.25"=c("1","0.25"),"0.625"=c("0.5","0.125"))

# Download files from GEO data base
LoadGSE<-function(Dataset,Directory="NewExperiment"){
	setwd(HostDir)
	setwd("GEMusicAR")
	CelDir<-paste("Cel Files",Dataset,sep="")
	getGEOSuppFiles(Dataset)
	untar((paste(Dataset,"/",Dataset,"_RAW.tar",sep="")),exdir=CelDir)
	cels<-list.files(CelDir,pattern="[gz]")
	sapply(paste(CelDir,cels,sep="/"),gunzip)
	setwd(HostDir)
}

LoadGSM<-function(Dataset,Directory="NewExperiment"){
	setwd(HostDir)
	setwd("GEMusicAR")
```

```
	CelDir<-paste("Cel Files",Directory,sep="")
	dir.create(CelDir,showWarnings=FALSE)
	setwd(CelDir)
	getGEOSuppFiles(Dataset,makeDirectory=FALSE)
	setwd(HostDir)
}

PrepareCels<-function(Directory="NewExperiment"){
	setwd(HostDir)
	setwd("GEMusicAR")
	CelDir<-paste("Cel Files",Directory,sep="")
	cels<-list.files(CelDir,pattern="[gz]")
	sapply(paste(CelDir,cels,sep="/"),gunzip)
	setwd(HostDir)
}

# Apply MAS5
ProcessCelMAS<-function(Dataset,out=Dataset){
	setwd(HostDir)
	setwd(paste("GEMusicAR/Cel Files",Dataset,sep=""))
	abatch<-ReadAffy()
	eset<-mas5(abatch)
	SI<-log2(exprs(eset))
	setwd(HostDir)
	setwd(paste("GEMusicAR/Signal Intensities",sep=""))
	write.table(SI,paste(out,"SIlog2",sep=""),sep="\t")
	setwd(HostDir)
}

# Apply RMA
ProcessCelRMA<-function(Dataset,level="core",out=Dataset){
	setwd(HostDir)
	setwd(paste("GEMusicAR/Cel Files",Dataset,sep=""))
	cFiles<-list.celfiles(paste(HostDir,"/GEMusicAR/Cel Files",
		Dataset,sep=""))
	celFiles<-read.celfiles(cFiles)
	SIrma<-rma(celFiles,target=level)
	SI<-(exprs(SIrma))
	setwd(HostDir)
	setwd(paste("GEMusicAR/Signal Intensities",sep=""))
	write.table(SI,paste(out,"SIlog2",sep=""),sep="\t")
	setwd(HostDir)
}

# Modify sample names
replacenames<-function(signalslog2,samp){
	last<-length(samp)-1
	for(i in 1:last){
		colnames(signalslog2)[i]<-samp[i]
	}
	signalslog2
}

# Set parameters
SetPar<-function(minfreq=27.5,tonesteps=12,numkeys=88,
	mindur=8,vol=1,TeXscalefactor=1,maxNdots=2){
#	minimal frequency
	assign("minfreq",minfreq,envir=globalenv())
```

```
#	number of tone steps per octave
	assign("tonesteps",tonesteps,envir=globalenv())
#	number of different tone frequencies on the "instrument"
	assign("numkeys",numkeys,envir=globalenv())
#	1/minimal tone length
	assign("mindur",mindur,envir=globalenv())
#	volume
   assign("vol",vol,envir=globalenv())
#	scale factor for TeX
	assign("TeXscalefactor",TeXscalefactor,envir=globalenv())
#	maximal number of dots
	assign("maxNdots",maxNdots,envir=globalenv())
}

# Load data
LoadSIforGEMusicA<-function(Dataset,newnames=FALSE){
	setwd(HostDir)
	setwd(paste("GEMusicAR",sep=""))
	subdout<-paste(Dataset,"Results",sep="")
	dir.create(subdout,showWarnings = FALSE)
	setwd(HostDir)
	setwd("GEMusicAR/Signal Intensities")
	signalslog2<-read.table(paste(Dataset,"SIlog2",sep=""),sep="\t")
	if(newnames==TRUE){
		signalslog2<-replacenames(signalslog2,samp)
	}
	colnames(signalslog2)<-gsub("[[:punct:]]","",colnames(signalslog2))
	assign("signalslog2",signalslog2,envir=globalenv())
	setwd(HostDir)
}

# Pre-filtered probe sets
EFTvsNB<-c("200715_x_at","200853_at","200951_s_at","200953_s_at","201028_s_at",
	"201029_s_at","201074_at","201075_s_at","201260_s_at","201291_s_at","201292_at",
	"201331_s_at","201397_at","201562_s_at","201563_at","201565_s_at","201566_x_at",
	"201648_at","201666_at","201890_at","201976_s_at","201981_at","201982_s_at",
	"202060_at","202145_at","202419_at","202431_s_at","202503_s_at","202554_s_at",
	"202709_at","202720_at","202746_at","202747_s_at","202758_s_at","202790_at",
	"202870_s_at","203065_s_at","203213_at","203240_at","203304_at","203324_s_at",
	"203418_at","203554_x_at","203625_x_at","203646_at","203757_s_at","203764_at",
	"203790_s_at","203824_at","203832_at","203856_at","203914_x_at","204023_at",
	"204197_s_at","204198_s_at","204201_s_at","204229_at","204230_s_at",
	"204244_s_at","204386_s_at","204393_s_at","204424_s_at","204456_s_at",
	"204457_s_at","204603_at","204686_at","204712_at","204779_s_at","204822_at",
	"204860_s_at","204867_at","204887_s_at","204917_s_at","204951_at","205046_at",
	"205066_s_at","205097_at","205103_at","205131_x_at","205145_s_at","205157_s_at",
	"205176_s_at","205199_at","205227_at","205306_x_at","205307_s_at","205345_at",
	"205375_at","205395_s_at","205440_s_at","205473_at","205478_at","205542_at",
	"205552_s_at","205597_at","205604_at","205694_at","205752_s_at","205827_at",
	"205828_at","205872_x_at","205888_s_at","205932_s_at","205962_at","205990_s_at",
	"206002_at","206025_s_at","206026_s_at","206110_at","206114_at","206149_at",
	"206159_at","206164_at","206231_at","206309_at","206316_s_at","206326_at","206456_at",
	"206481_s_at","206558_at","206565_x_at","206581_at","206626_x_at","206644_at",
	"206645_s_at","206743_s_at","206745_at","206812_at","206866_at","206906_at",
	"206915_at","206935_at","207016_s_at","207059_at","207069_s_at","207118_s_at",
	"207165_at","207172_s_at","207173_x_at","207373_at","207395_at","207397_s_at",
	"207398_at","207400_at","207493_x_at","207563_s_at","207587_at","207666_x_at",
	"207670_at","207675_x_at","207736_s_at","207772_s_at","207869_s_at","207957_s_at",
```

```
        "208060_at","208214_at","208237_x_at","208242_at","208262_x_at","208286_x_at",
        "208312_s_at","208334_at","208345_s_at","208465_at","208470_s_at","208510_s_at",
        "208565_at","208771_s_at","208796_s_at","208950_s_at","208951_at","209129_at",
        "209270_at","209294_x_at","209340_at","209628_at","209629_s_at","209655_s_at",
        "209656_s_at","209685_s_at","209714_s_at","209734_at","209752_at","209773_s_at",
        "209791_at","209844_at","209885_at","209979_at","210107_at","210233_at","210237_at",
        "210374_x_at","210375_at","210394_x_at","210475_at","210497_x_at","210673_x_at",
        "210727_at","210770_s_at","210783_x_at","210831_s_at","210911_at","210931_at",
        "210952_at","210956_at","210960_at","210961_s_at","210963_s_at","210964_s_at",
        "211032_at","211042_x_at","211128_at","211138_s_at","211253_x_at","211307_s_at",
        "211419_s_at","211425_x_at","211657_at","211709_s_at","211802_x_at","211953_s_at",
        "211954_s_at","211955_at","212045_at","212097_at","212236_x_at","212278_x_at",
        "212307_s_at","212402_at","212415_at","212501_at","212612_at","212621_at","212713_at",
        "212739_s_at","212909_at","212920_at","213173_at","213385_at","213395_at","213425_at",
        "213524_s_at","213552_at","213589_s_at","213714_at","213793_s_at","213811_x_at",
        "213825_at","213931_at","214028_x_at","214290_s_at","214307_at","214362_at",
        "214444_s_at","214460_at","214498_at","214584_x_at","214604_at","214611_at",
        "214636_at","214730_s_at","214929_s_at","214933_at","215006_at","215043_s_at",
        "215071_s_at","215094_at","215161_at","215300_s_at","215322_at","215365_at",
        "215509_s_at","215510_at","215559_at","215652_at","215695_s_at","215774_s_at",
        "215876_at","215931_s_at","215962_at","216000_at","216052_x_at","216222_s_at",
        "216228_s_at","216471_x_at","216624_s_at","216818_s_at","217013_at","217026_at",
        "217303_s_at","217316_at","217521_at","217549_at","217569_x_at","217647_at",
        "217698_at","218078_s_at","218182_s_at","218280_x_at","218313_s_at","218663_at",
        "218665_at","218782_s_at","218831_s_at","218847_at","218883_s_at","218959_at",
        "219090_at","219102_at","219104_at","219306_at","219343_at","219360_s_at",
        "219427_at","219528_s_at","219555_s_at","219572_at","219597_s_at","219663_s_at",
        "219686_at","219703_at","219766_at","219825_at","219837_s_at","219887_at",
        "219908_at","219918_s_at","219937_at","219976_at","220047_at","220218_at",
        "220227_at","220315_at","220333_at","220362_at","220588_at","220654_at","220672_at",
        "220756_s_at","220780_at","220786_s_at","220820_at","220828_s_at","220900_at",
        "220906_at","220942_x_at","220948_s_at","221011_s_at","221022_s_at","221076_at",
        "221129_at","221139_s_at","221185_s_at","221215_s_at","221308_at","221552_at",
        "221606_s_at","221679_s_at","221691_x_at","221703_at","221854_at","221923_s_at",
        "221928_at","222025_s_at","222067_x_at","222209_s_at","222254_at","222312_s_at",
        "222340_at","222379_at","43427_at","45288_at","49452_at","57588_at","61734_at",
        "AFFX-r2-Hs18SrRNA-M_x_at")

# Model melody data
# Song of Joy (L. v. Beethoven, Symphony No.9; source: transcription
# for piano solo by F. Liszt, Kalmus K09228 edition, Belwin Mills
# Publishing Corp., Miami, FL, USA, page 196, bars 5-20).
SoJ<-data.frame(
        dur=c(1,1,1,1,1,1,1,1,1,1,1,1,1.5,0.5,2,1,1,1,1,1,1,1,1,1,1,1,
                1,1,1.5,0.5,2,1,1,1,1,1,0.5,0.5,1,1,1,0.5,0.5,1,1,1,1,1,2,1,
                1,1,1,1,1,0.5,0.5,1,1,1,1,1.5,0.5,2),
        keys<-c(34,34,35,37,37,35,34,32,30,30,32,34,34,32,32,34,34,
                35,37,37,35,34,32,30,30,32,34,32,30,30,32,32,34,30,32,34,35,
                34,30,32,34,35,34,32,30,32,25,34,34,35,37,37,35,34,35,32,30,
                30,32,34,32,30,30),
        Name="SoJ",
        Metro=100)

# Ride of the Valkyries (R. Wagner, Die Walküre; source: trombone
# part of the Philharmonia pocket score edition No. 123, Wiener
# Philharmonischer Verlag, Vienna, Austria, pages 25-34, bars 58-75)
Valkyries<-data.frame(
        dur=c(0.5,0.75,0.25,0.5,1.5,1.5,0.75,0.25,0.5,1.5,1.5,0.75,
```

```
            0.25,0.5,1.5,1.5,0.75,0.25,0.5,2.5,0.5,0.75,0.25,0.5,1.5,
            1.5,0.75,0.25,0.5,1.5,1.5,0.75,0.25,0.5,1.5,1.5,0.75,0.25,
            0.5,2.5,0.5,0.75,0.25,0.5,2.5,0.5,0.75,0.25,0.5,2.5,0.5,
            0.75,0.25,0.5,1.5,1.5,0.75,0.25,0.5,2.5,0.5,0.75,0.25,0.5,
            2.5,0.5,0.75,0.25,0.5,2.5,0.5,0.75,0.25,0.5,1.5,1.5,0.75,
            0.25,0.5,0.75,0.25,0.5,0.75,0.25,0.5,4),
      keys=c(22,27,22,27,31,27,31,27,31,34,31,34,31,34,38,26,31,
            26,31,34,26,31,26,31,34,31,34,31,34,38,34,38,34,38,41,29,
            34,29,34,38,22,24,20,24,27,34,36,32,36,39,34,24,20,24,29,
            22,27,22,27,31,22,24,20,24,27,34,36,32,36,39,22,24,20,
            24,29,22,27,22,27,31,27,31,34,31,34,39),
      Name="Valkyrie",
      Metro=100)

# Landmann (R. Schumann, Fröhlicher Landmann, von der Arbeit
# zurückkehrend; source: Robert Schumann's Werke, Serie VII,
# No. 67, 43 Clavierstücke für die Jugend (Clara Schumann, Ed.),
# 1887, Breitkopf & Härtel, Leipzig, Germany, page 8)
Landmann<-data.frame(
      dur=c(0.5,1.5,0.5,1.5,0.5,0.5,0.5,0.5,0.5,1.5,0.5,0.5,
            0.5,0.5,0.5,0.5,0.5,0.5,0.5,1,1,1.5,0.5,1.5,0.5,1.5,0.5,
            0.5,0.5,0.5,0.5,1.5,0.5,0.5,0.5,0.5,0.5,0.5,0.5,0.5,0.5,
            1,1,1.5,0.5,1.5,0.5,1.5,0.5,0.5,0.5,0.5,0.5,1.5,0.5,1.5,0.5,
            1.5,0.5,0.5,0.5,0.5,0.5,1.5,0.5,0.5,0.5,0.5,0.5,0.5,0.5,0.5,
            0.5,1,1,1.5,0.5,1.5,0.5,1.5,0.5,0.5,0.5,0.5,0.5,1.5,0.5,1.5,
            0.5,1.5,0.5,0.5,0.5,0.5,0.5,1.5,0.5,0.5,0.5,0.5,0.5,0.5,0.5,
            0.5,0.5,1,1,1.5),
      keys=c(16,21,25,28,21,26,30,33,30,28,25,26,23,16,26,25,21,
            16,25,20,18,16,16,21,25,28,21,26,30,33,30,28,25,26,23,16,
            26,25,21,16,25,20,18,16,16,23,21,20,16,23,21,20,18,20,16,
            21,25,28,21,26,30,33,30,28,25,26,23,16,26,25,21,16,25,20,18,
            21,16,23,21,20,16,23,21,20,18,20,16,21,25,28,21,26,30,33,30,
            28,25,26,23,16,26,25,21,16,25,20,18,21),
      Name="Landmann",
      Metro=100)

# Choral No. 1. (Joseph Klug, Geistliche Lieder, Wittenberg, 1535;
# source: Choralbuch für den Consistorialbezirk Cassel, 2nd Edition
# 1897, E. Röttger, Kassel, Germany, page 30).
Choral1<-data.frame(
      dur=c(1,1,1,1,1,1,2,2,1,1,1,1,2,3,1,1,1,1,1,1,1,2,2,1,1,
            1,1,2,3,1,1,1,1,1,1,1,3,1,1,1,1,1,1,2,2,1,1,1,1,2,4),
      keys=c(35,35,39,37,35,37,37,39,35,39,40,42,39,37,35,35,35,
            39,37,35,37,37,39,35,39,40,42,39,37,35,39,39,37,35,34,35,
            32,30,30,35,35,35,30,35,37,39,35,39,40,42,39,37,35),
      Name="ChoralNo1",
      Metro=120)

# Choral No. 2. (Medieval melody; source: Choralbuch für den
# Consistorialbezirk Cassel, 2nd Edition 1897, E. Röttger, Kassel,
# Germany, page 11).
Choral2<-data.frame(
      dur=c(2,1,1,1,1,2,1,1,1,1,1,1,1.5,0.5,1,1,1,1,1,1,2,1,1,1,
            1,1,1,1,2,1,2,4,1,1,1,1,1,1,1.5,0.5,1,1,1,1,1,1,2,1,1,1,1,
            1,1,1.5,0.5,1,1,1,1,1,1,1,2,1,2,4,2,1,1,1,1,2,2,1,1,1,1,2,
            2,1,1,1,1,1.5,0.5,1,1,1,1,1,1,2,1,1,1,1,1,1,1,2,1,2,4),
      keys<-c(37,35,37,40,42,37,37,35,37,33,32,33,30,30,35,35,
            30,28,33,35,37,37,35,37,33,32,33,30,32,29,30,30,37,37,35,
```

```
            37,40,42,37,37,37,35,37,33,32,33,30,35,35,30,28,33,35,37,
            37,37,35,37,33,32,33,30,32,29,30,30,37,35,33,35,38,37,
            40,37,40,37,35,33,37,33,30,32,33,30,30,35,35,30,28,33,
            35,37,37,35,37,33,32,33,30,32,29,30,30),
      Name="ChoralNo2",
      Metro=120)

# Melody (source: original).
Melody<-data.frame(
      dur=c(1.5,0.5,1,1,1.5,0.5,0.5,0.5,1,1.5,0.5,0.5,0.5,1,1.5,
            0.5,0.5,0.5,1,1.5,0.5,1,1,1.5,0.5,1,1,0.5,0.5,3,1.5,0.5,1,
            1,1.5,0.5,1,1,1.5,0.5,0.5,0.5,1,1.5,0.5,0.5,0.5,1,2,1,1,
            2,1,1,1.5,0.5,1,1,0.5,0.5,3),
      keys<-c(16,23,16,18,16,23,22,20,28,28,16,16,20,22,21,24,
            19,20,21,21,18,20,21,18,16,20,24,22,16,21,22,20,24,16,
            23,16,20,18,28,16,24,17,20,16,23,22,20,28,23,24,21,20,
            21,22,18,16,20,24,22,16,21),
      Name="Melody",
      Metro=120)

# Chromatic scale.
ChromoScale1<-data.frame(
      dur=c(rep(1,25)),
      keys=c(25:49),
      Name="ChromaticScale",
      Metro=60)

# Chromatic scale 2.
ChromoScale2<-data.frame(
      dur=c(1.5,0.75,0.375,0.1875,0.09375,0.046875,
            0.0234375,0.01171875,1.75,0.875,0.4375,0.21875,0.109375,
            0.0546875,0.02734375,0.013671875,rep(c(1,0.5,0.25,0.125,0.0625,
            0.03125,0.015625,0.0078125,1.5,0.75,0.375,0.1875,0.09375,
            0.046875,0.0234375,0.01171875,1.75,0.875,0.4375,0.21875,0.109375,
            0.0546875,0.02734375,0.013671875),3)),
      keys=c(1:88),
      Name="ChromaticScale2",
      Metro=100)

# Long notes.
Longdur<-data.frame(
      dur=c(rep(7.5,88)),
      keys=c(1:88),
      Name="LongNotes",
      Metro=100)

# Create working directories
HostDir<-getwd()
dir.create("GEMusicAR",showWarnings=FALSE)
setwd("GEMusicAR")
dir.create("Signal Intensities",showWarnings=FALSE)
dir.create("GEMusicAR Filter",showWarnings=FALSE)
dir.create("GEMusicAR TXT Input",showWarnings=FALSE)
setwd("GEMusicAR Filter")
write.table(EFTvsNB,"EFTspec.txt",quote=FALSE,row.names=FALSE,col.names=FALSE)
setwd(HostDir)
```

```
#----------------------------------------------------------------------
# Part B (Examples)
#----------------------------------------------------------------------

# Data set 1:
# Neuroblastoma/Ewing sarcoma cell lines
# GEO data set GSE1824

# Load Data
setwd(HostDir)
setwd(paste("GEMusicAR",sep=""))
Experiment1=("GSE1824")
LoadGSE(Experiment1)
ProcessCelMAS(Dataset=Experiment1)
setwd(HostDir)

# The following examples are discussed in the paper:
# Martin S. Staege (2015) "A short treatise concerning a
# musical approach for the interpretation of gene expression data"

# Example 1: 2228 probe sets
# WARNING: generation of .wav files will take few minutes!!
samp<-c("4SKNMC","3SIMA","1CHP","2SHSY","5MedSRBC")
LoadSIforGEMusicA(Dataset=Experiment1,newnames=TRUE)
SetPar(minfreq=27.5,tonesteps=12,numkeys=88,
        mindur=8,vol=4,TeXscalefactor=1,maxNdots=2)
GenerateNtones(Dataset="GSE1824",N=2228)
setwd(HostDir)

# Example 2: 192 probe sets
samp<-c("13SKNMC","12SIMA","10CHP","11SHSY","14MedSRBC")
LoadSIforGEMusicA(Dataset=Experiment1,newnames=TRUE)
SetPar(minfreq=27.5,tonesteps=12,numkeys=88,
        mindur=8,vol=4,TeXscalefactor=1,maxNdots=2)
GenerateNtones(Dataset="GSE1824",N=192)
setwd(HostDir)

# Example 3: Pre-filtered probe sets
samp<-c("22SKNMC","21SIMA","19CHP","20SHSY","23MedSRBC")
LoadSIforGEMusicA(Dataset=Experiment1,newnames=TRUE)
GeneratePrefiltered(Dataset="GSE1824",filteredPS="EFTspec.txt")
SetPar(minfreq=27.5,tonesteps=12,numkeys=88,
        mindur=8,vol=4,TeXscalefactor=1,maxNdots=2)
GenerateNtones(Dataset="GSE1824",N=192,PSfilter="EFTspec")
setwd(HostDir)

# Example 4: Model melodies
# A: Song of Joy
samp<-c("57SKNMC","56SIMA","54CHP","55SHSY","58Med")
LoadSIforGEMusicA(Dataset=Experiment1,newnames=TRUE)
SetPar(minfreq=27.5,tonesteps=12,numkeys=88,
        mindur=8,vol=4,TeXscalefactor=4,maxNdots=2)
ExtractModel(Dataset="GSE1824",Model=SoJ)
setwd(HostDir)

# B: Ride of the Valkyries
samp<-c("66SKNMC","65SIMA","63CHP","64SHSY","67Med")
LoadSIforGEMusicA(Dataset=Experiment1,newnames=TRUE)
```

```
SetPar(minfreq=27.5,tonesteps=12,numkeys=88,
        mindur=8,vol=4,TeXscalefactor=4,maxNdots=2)
ExtractModel(Dataset="GSE1824",Model=Valkyries)
setwd(HostDir)

# Additional models
# 1. Landmann
samp<-c("SKNMC","SIMA","CHP","SHSY","Med")
LoadSIforGEMusicA(Dataset=Experiment1,newnames=TRUE)
SetPar(minfreq=110,tonesteps=12,numkeys=42,
        mindur=8,vol=4,TeXscalefactor=4,maxNdots=2)
ExtractModel(Dataset="GSE1824",Model=Landmann)
setwd(HostDir)

# 2. Choral No. 1
samp<-c("SKNMC","SIMA","CHP","SHSY","Med")
LoadSIforGEMusicA(Dataset=Experiment1,newnames=TRUE)
SetPar(minfreq=55,tonesteps=12,numkeys=54,
        mindur=8,vol=4,TeXscalefactor=4,maxNdots=2)
ExtractModel(Dataset="GSE1824",Model=Choral1)
setwd(HostDir)

# 3. Choral No. 2
samp<-c("SKNMC","SIMA","CHP","SHSY","Med")
LoadSIforGEMusicA(Dataset=Experiment1,newnames=TRUE)
SetPar(minfreq=55,tonesteps=12,numkeys=54,
        mindur=8,vol=4,TeXscalefactor=4,maxNdots=2)
ExtractModel(Dataset="GSE1824",Model=Choral2)
setwd(HostDir)

# 4. Original Melody
samp<-c("SKNMC","SIMA","CHP","SHSY","Med")
LoadSIforGEMusicA(Dataset=Experiment1,newnames=TRUE)
SetPar(minfreq=220,tonesteps=12,numkeys=30,
        mindur=8,vol=4,TeXscalefactor=4,maxNdots=2)
ExtractModel(Dataset="GSE1824",Model=Melody)
setwd(HostDir)

# 5. Chromatic scale No. 1
samp<-c("SKNMC","SIMA","CHP","SHSY","Med")
LoadSIforGEMusicA(Dataset=Experiment1,newnames=TRUE)
SetPar(minfreq=55,tonesteps=12,numkeys=54,
        mindur=8,vol=4,TeXscalefactor=4,maxNdots=2)
ExtractModel(Dataset="GSE1824",Model=ChromoScale1)
setwd(HostDir)

# 6. Chromatic scale No. 2
samp<-c("SKNMC","SIMA","CHP","SHSY","Med")
LoadSIforGEMusicA(Dataset=Experiment1,newnames=TRUE)
SetPar(minfreq=27.5,tonesteps=12,numkeys=88,
        mindur=8,vol=4,TeXscalefactor=1,maxNdots=2)
ExtractModel(Dataset="GSE1824",Model=ChromoScale2)
setwd(HostDir)

# 7. Long duration
samp<-c("SKNMC","SIMA","CHP","SHSY","Med")
LoadSIforGEMusicA(Dataset=Experiment1,newnames=TRUE)
SetPar(minfreq=27.5,tonesteps=12,numkeys=88,
```

```
        mindur=8,vol=4,TeXscalefactor=4,maxNdots=2)
ExtractModel(Dataset="GSE1824",Model=Longdur)
setwd(HostDir)

#-----------------------------------------------------------------------

# Data set 2:
# Hodgkin's lymphoma/B cell data
# GEO data sets GSE47686 and GSE20200 (partial)

# Load Data
setwd(HostDir)
setwd("GEMusicAR")
Experiment2<-"HLvsB"
LoadGSM(Dataset="GSM1154791",Directory=Experiment2)
LoadGSM(Dataset="GSM1154792",Directory=Experiment2)
LoadGSM(Dataset="GSM1154793",Directory=Experiment2)
LoadGSM(Dataset="GSM506714",Directory=Experiment2)
LoadGSM(Dataset="GSM506715",Directory=Experiment2)
LoadGSM(Dataset="GSM506716",Directory=Experiment2)
PrepareCels(Directory=Experiment2)
setwd(HostDir)

# Example 5: Probeset level
Experiment2A<-"HLvsBEx"
ProcessCelRMA(Dataset=Experiment2,level="probeset",out=Experiment2A)
samp<-c("32L540Ex","33L428Ex","31HDLM2Ex","28CD19aEx",
        "29CD19bEx","30CD19cEx","34MedEx")
LoadSIforGEMusicA(Dataset=Experiment2A,newnames=TRUE)
SetPar(minfreq=27.5,tonesteps=12,numkeys=88,
        mindur=8,vol=4,TeXscalefactor=1,maxNdots=2)
GenerateNtones(Dataset=Experiment2A,N=288)
setwd(HostDir)

# Example 6: Core Gene level
Experiment2B<-"HLvsBGe"
ProcessCelRMA(Dataset=Experiment2,level="core",out=Experiment2B)
samp<-c("45L540Ge","46L428Ge","44HDLM2Ge","41CD19aGe",
        "42CD19bGe","43CD19cGe","47MedGe")
LoadSIforGEMusicA(Dataset=Experiment2B,newnames=TRUE)
SetPar(minfreq=27.5,tonesteps=12,numkeys=88,
        mindur=8,vol=4,TeXscalefactor=1,maxNdots=2)
GenerateNtones(Dataset=Experiment2B,N=288)
setwd(HostDir)
```

C. ArrayMusic: PERL script used for generation of wavesound files

The following PERL script generates a wavesound file that approximates the expected melodies from the GEMusicA. The script is essentially based on common Win32::Sound scripts, *e.g.* http://cpansearch.perl.org/src/JDB/Win32-Sound-0.49/Sound.pm. The script expects a text file that contains two columns: the first column should contain the frequencies, the second column should contain the durations of the tones. The R script GEMusicAR (see above) generates these files automatically. The PERL script can be saved as text file ArrayMusic.pl and used

together with PERL compilers, *e.g.* ActivePerl (ActiveState Software Inc., Vancouver, BC, Canada). The script has to be started with the command *ArrayMusic.pl x.txt* where *x.txt* is the name of the text file with the data that will be transformed into sounds. The script generates a new file *melody.wav* which can be processed with any multimedia or music program, e.g. Acid Xpress (Sony Creative Software Inc., Mittleton, WI).

```
use 5.016;
use Win32::Sound;
open(IN, $ARGV[0]);
my @data= <IN>;
close(IN);
my @values=();
my @durations=();
foreach my $l (@data){
    chomp($l);
    my @tmp= split("\t", $l);
    push(@values, $tmp[0]);
    push(@durations, $tmp[1]);
}
my $wav = new Win32::Sound::WaveOut(88200, 16, 2);
my $dat = "";
my $index = 0;
foreach my $i (@values){
    $dat .= tone($i, $durations[$index]);
    $index ++;
}
$wav->Load($dat);
$wav->Write();
1 while(! $wav->Status());
$wav->Save("melody.wav");
$wav->Unload();
sub tone{
    my ($freq, $duration)= @_;
    my $data = "";
    my $counter = 0;
    my $incr = $freq/44100;
    for my $i(1 .. $duration*88200){
        my $v = sin($counter/2*3.141592653589793238462643383279) * 128 + 128;
        $data .= pack("cc", $v, $v);
        $counter += $incr;
    }
    return($data);
}
```

D. Index of Supplementary audio files

The following sound examples are available as MP3 files (IMSLP, “Tumor music”):

Microarray data	Files	Comments
Neuroblastoma/<br>Ewing sarcoma	1CHP2228<br>2SHSY5Y2228<br>3SIMA2228<br>4SKNMC2228<br>5MedSRBCT2228	Generated with ArrayMusic.pl. 2228 probe sets with highest variance in the neuroblastoma/ Ewing sarcoma data set.
Neuroblastoma/<br>Ewing sarcoma	6CHP2228st<br>7SHSY5Y2228st<br>8SIMA2228st<br>9SKNMC2228st	Generated with ArrayMusic.pl and Acid Xpress. 2228 probe sets with highest variance in the neuroblastoma/Ewing sarcoma data set. For each cell line, the individual melody is displayed together with the melody of the median (two channels; stereo required).
Neuroblastoma/<br>Ewing sarcoma	10CHP192<br>11SHSY5Y192<br>12SIMA192<br>13SKNMC192<br>14MedSRBCT192	Generated with ArrayMusic.pl. 192 probe sets with highest variance in the neuroblastoma/Ewing sarcoma data set.
Neuroblastoma/<br>Ewing sarcoma	15CHP192st<br>16SHSY5Y192st<br>17SIMA192st<br>18SKNMC192st	Generated with ArrayMusic.pl and Acid Xpress. 192 probe sets with highest variance in the neuroblastoma/Ewing sarcoma data set. For each cell line, the individual melody is displayed together with the melody of the median (two channels; stereo required).
Neuroblastoma/<br>Ewing sarcoma	19CHPEFTspec192<br>20SHSY5YEFTspec192<br>21SIMAEFTspec192<br>22SKNMCEFTspec192<br>23MedEFTspec192	Generated with ArrayMusic.pl. 192 probe sets with highest variance in the neuroblastoma/Ewing sarcoma data set. Probe sets were pre-filtered for EFT specificity by using MAFilter. The probe sets are presented on page 124-5 of this Supplement.
Neuroblastoma/<br>Ewing sarcoma	24CHP126EFTspec192st<br>25SHSY5YEFTspec192st<br>26SIMAEFTspec192st<br>27SKNMCEFTspec192st	Generated with ArrayMusic.pl and Acid Xpress. 192 probe sets with highest variance in the neuroblastoma/Ewing sarcoma data set. Probe sets were pre-filtered for EFT-specificity. For each cell line, the individual

		melody is displayed together with the melody of the median (two channels, stereo required).
Hodgkin's lymphoma/<br>B cells;<br><br>exon level	28CD19aEx288<br>29CD19bEx288<br>30CD19cEx288<br>31HDLM2Ex288<br>32L540Ex288<br>33L428Ex288<br>34MedHLEx288	Generated with ArrayMusic.pl. 288 probe sets with highest variance in the in the Hodgkin's lymphoma/ B cell data set (exon level analysis).
Hodgkin's lymphoma/<br>B cells;<br><br>exon level	35CD19aEx288st<br>36CD19bEx288st<br>37CD19cEx288st<br>38HDLM2Ex288st<br>39L428Ex28st<br>40L540Ex288st	Generated with ArrayMusic.pl and ACID Xpress. 288 probe sets with highest variance in the Hodgkin's lymphoma/B cell data set (exon level analysis). For each cell line, the individual melody is displayed together with the melody of the median (two channels; stereo required).
Hodgkin's lymphoma/<br>B cells;<br><br>gene level	41CD19aGe288<br>42CD19bGe288<br>43CD19cGen288<br>44HDLM2Ge288<br>45L540Ge288<br>46L428Ge288<br>47MedHLGe288	Generated with ArrayMusic.pl. 288 probe sets with highest variance in the in the Hodgkin's lymphoma/ B cell data set (gene level analysis).
Hodgkin's lymphoma/<br>B cells;<br><br>gene level	48CD19aGe288st<br>49CD19bGe288st<br>50CD19cGe288st<br>51HDLM2Ge288st<br>52L428Ge288st<br>53L540 Ge288st	Generated with ArrayMusic.pl and ACID Xpress. 288 probe sets with highest variance in the Hodgkin's lymphoma/B cell data set (gene level analysis). For each cell line, the individual melody is displayed together with the melody of the median (two channels; stereo required).
Neuroblastoma/<br>Ewing sarcoma	54CHPSoJ<br>55SHSY5YSoJ<br>56SIMASoJ<br>57SKNMCSoJ<br>58MedSRBCTSoJ	Generated with ArrayMusic.pl. 63 probe sets with highest variance in the neuroblastoma/Ewing sarcoma data set. Frequencies were recalibrated on the basis of L. v. Beethoven's "Song of Joy". The median was adjusted to the original melody.
Neuroblastoma/<br>Ewing sarcoma	59CHPSoJst<br>60SHSY5YSoJst<br>61SIMASoJst<br>62SKNMCSoJst	Generated with ArrayMusic.pl and ACID Xpress. 63 probe sets with highest variance in the neuroblastoma/Ewing sarcoma data set. Frequencies were

		recalibrated on the basis of L. v. Beethoven's "Song of Joy". The median was adjusted to the original melody. For each cell line, the individual melody is displayed together with the melody of the median (two channels; stereo required).
Neuroblastoma/ Ewing sarcoma	63CHPVakyrie<br>64SHSY5YValkyrie<br>65SIMAValkyrie<br>66SKNMCValkyrie<br>67MedSRBCTValkyrie	Generated with ArrayMusic.pl. 86 probe sets with highest variance in the neuroblastoma/Ewing sarcoma data set. Frequencies were recalibrated on the basis of R. Wagner's "Ride of the Valkyries". The median was adjusted to the original melody.
Neuroblastoma/ Ewing sarcoma	68CHPVakyriest<br>69SHSY5YValkyriest<br>70SIMAValkyriest<br>71SKNMCValkyriest	Generated with ArrayMusic.pl and ACID Xpress. 86 probe sets with highest variance in the neuroblastoma/Ewing sarcoma data set. Frequencies were recalibrated on the basis of R. Wagner's "Ride of the Valkyries". The median was adjusted to the original melody. For each cell line, the individual melody is displayed together with the melody of the median (two channels; stereo required).
